# Lessons Learned from Crowdsourcing Complex Engineering Tasks

**DOI:** 10.1371/journal.pone.0134978

**Published:** 2015-09-18

**Authors:** Matthew Staffelbach, Peter Sempolinski, Tracy Kijewski-Correa, Douglas Thain, Daniel Wei, Ahsan Kareem, Gregory Madey

**Affiliations:** 1 Department of Computer Science and Engineering, University of Notre Dame, Notre Dame, Indiana, United States of America; 2 Department of Civil & Environmental Engineering & Earth Sciences, University of Notre Dame, Notre Dame, Indiana, United States of America; Northwestern University, UNITED STATES

## Abstract

**Crowdsourcing:**

Crowdsourcing is the practice of obtaining needed ideas, services, or content by requesting contributions from a large group of people. Amazon Mechanical Turk is a web marketplace for crowdsourcing microtasks, such as answering surveys and image tagging. We explored the limits of crowdsourcing by using Mechanical Turk for a more complicated task: analysis and creation of wind simulations.

**Harnessing Crowdworkers for Engineering:**

Our investigation examined the feasibility of using crowdsourcing for complex, highly technical tasks. This was done to determine if the benefits of crowdsourcing could be harnessed to accurately and effectively contribute to solving complex real world engineering problems. Of course, untrained crowds cannot be used as a mere substitute for trained expertise. Rather, we sought to understand how crowd workers can be used as a large pool of labor for a preliminary analysis of complex data.

**Virtual Wind Tunnel:**

We compared the skill of the anonymous crowd workers from Amazon Mechanical Turk with that of civil engineering graduate students, making a first pass at analyzing wind simulation data. For the first phase, we posted analysis questions to Amazon crowd workers and to two groups of civil engineering graduate students. A second phase of our experiment instructed crowd workers and students to create simulations on our Virtual Wind Tunnel website to solve a more complex task.

**Conclusions:**

With a sufficiently comprehensive tutorial and compensation similar to typical crowd-sourcing wages, we were able to enlist crowd workers to effectively complete longer, more complex tasks with competence comparable to that of graduate students with more comprehensive, expert-level knowledge. Furthermore, more complex tasks require increased communication with the workers. As tasks become more complex, the employment relationship begins to become more akin to outsourcing than crowdsourcing. Through this investigation, we were able to stretch and explore the limits of crowdsourcing as a tool for solving complex problems.

## Introduction

### Citizen Engineering

Crowdsourcing, in recent years, has become one way to increase the supply of labor for problem solving tasks. The basic idea of crowdsourcing is that a lengthy task is divided into manageable pieces and these pieces are delivered to a large group of people to work on. These crowd workers might be compensated or act as volunteers. If the pool of workers is large enough, a crowdsourced task can be accomplished fairly quickly. However, there can be concerns over the quality of the subsequent result, since the workers are basically a large group of strangers. Often this is accounted for by making sure that the task is not too difficult and by having many workers independently work on the same thing, in order to verify results by consensus.

In this investigation, however, we were interested in exploring the limits of crowdsourcing as a means of accomplishing larger, more difficult technical tasks such as those found in civil engineering. While the United States is a highly developed country in its infrastructure and services, in recent years, there is has been some concern regarding the age of various buildings. According to ASCE’s (American Society of Civil Engineers) *2013 report card for America’s infrastructure* [1], the average grade for US civil infrastructure was a D+, with a 3.6 trillion dollar estimated investment needed by 2020 or else the US infrastructure will be aging faster than it is being replaced. Harnessing a large group of "Citizen Engineers" to perform at least some of the intellectual work on this, and other engineering problems, could be a means to expand the limited formal workplace available to respond to such grand challenges.

This work is part of a broader project, "Open-Sourcing the Design of Civil Infrastructure" (OSD-CI) supported by the NSF [2]. One of our motivations in this overall project was to explore the possibilities of using the "wisdom of the crowds" in civil engineering work for specific situations, such as in rebuilding after some natural disaster. The engineering, both physical and intellectual, after a large-scale calamity is significant. Furthermore, a general public, even worldwide, would be highly motivated to assist is such a scenario. The challenge lies in finding the sort of tasks that can be delegated to crowds. This particular study is motivated to stretch the limits of crowdsourcing, in an effort to begin to explore this area.

Civil Engineering, however, is a complex field, requiring specialized training and certification. Therefore, one of the main concerns with Citizen Engineering is insuring the accuracy of any result used. This is a significant concern when dealing with civil infrastructure for obvious reasons: flawed data could lead to loss of lives. Therefore, we could not under any circumstances minimize the need for trained expertise as the primary guarantor of safe and accurate results. Rather than allowing crowd workers to replace expert labor, which would be dangerous, we envision the role of crowd workers as an assistant to expert labor, since the time and effort of expert labor is limited and expensive.

Our goal was to determine if crowd workers on Mechanical Turk (a common crowdsourcing broker) were willing participants capable of learning new task types which were more complex than a typical crowdsourcing task. These tasks were a first pass at checking and evaluating wind simulation results. Simulations are cheap and plentiful, but ensuring useful simulations requires thoughtful analysis. If a group of crowd workers can filter out simulation results that are obviously wrong or produce results that are more likely to be useful, this can save an expert's time for more specialized analysis. Performing this filtering, however, required crowd workers to read comprehensive tutorials, give quality responses to engineering questions and use specialized software tools. After such training, however, crowdworkers were able to perform this task, with more than sufficient accuracy. In fact, on one occasion, the input of the crowd even caused our expert to reconsider their assessment of one simulation.

### Citizen Science

Crowdsourcing has been effectively applied in the sciences, even prior to the internet. For example, the Audubon Society has been harnessing the power of the crowds in order to effectively plot the location of hundreds of bird species in the United States for over 115 years. Thousands of Audubon members would mail in information stating the number species and locations of birds. Today, the Audubon society and the Cornell Lab of Ornithology run a real-time, online checklist program called eBird [3]. Some other famous instances of effective Citizen Science include Galaxy Zoo [4], a galaxy classifying website and Phylo [5] a game that allows crowds to help align related DNA sequences.

### Amazon Mechanical Turk

We used Amazon Mechanical Turk as our crowdsourcing engine. Mechanical Turk is a useful tool for conducting many types of research including behavioral research [6][7], survey research [8][9], economic game experiments [10], collective behavior experiments [11], exploring public knowledge gaps [12], and psychonomic research [13]. Others have shown that “Turkers” (online workers from Amazon Mechanical Turk) can be used to ensure text translation quality [14] and twitter has used crowd workers to categorize trending tweets [15]. Many of the typical uses for this website are image tagging, surveys, and audio transcription. In this paper, Mechanical Turk was our primary means of recruiting and engaging crowd workers.

### Virtual Wind Tunnel

**Fig 1:**
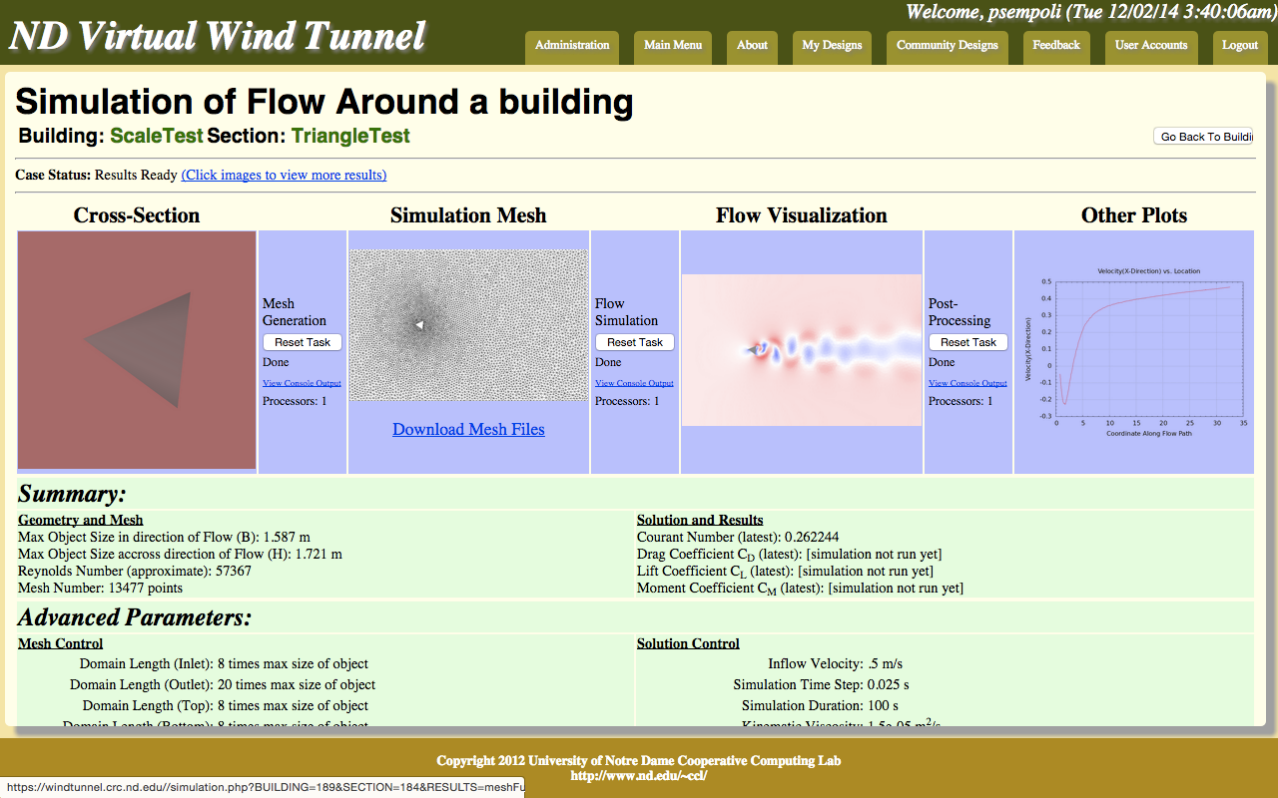
Screenshot of the Virtual Wind Tunnel Website. A screenshot of a page of the Virtual Wind Tunnel website that contains the tools for creating the simulations and was used by the crowdworkers in phase two of the experiment. This page shows various figures which are generated in each simulation. This simulation result shows a cross-section, simulation mesh, flow visualization and a velocity plot. Users can click on these images to see larger versions and get access to more results and images. There is also a summary of the simulation provided below the figures followed by a listing of the parameters used.

We designed a Virtual Wind Tunnel to enable non-technical users to access powerful simulation tools running in a cloud facility. The internals of this system, as well as other uses for which it was employed, are described in [16][17]. The basic idea is that this system can act as a web portal within which users can describe or upload building geometries and designs. Such uploaded designs can then be analyzed to determine wind loads acting on the structure. The analysis is facilitated by an underlying Computational Fluid Dynamics (CFD) program, OpenFOAM, which is an open-source CFD toolkit. Furthermore, this Virtual Wind Tunnel is sufficiently customizable, allowing the design of interfaces specifically for this study. Fig 1 shows a screenshot of the portal visualizing a single simulation.

We used wind load simulation as a representative complex Citizen Engineering task, because it provided a broad range of potential crowd tasks that would be unfamiliar to crowd workers and would require learning. For example, we could examine tasks that could not be broken into a series of simple tasks, i.e., "microtasks". Some other techniques for crowdsourcing complex tasks [18][19] would not be applicable, given that they concentrate on splitting the tasks into smaller non-complex tasks that can be taught to novices. However, training was an essential element of our tasks. Every volunteer for this study was required to read and comprehend a four to five page tutorial in order to begin to grasp the concepts necessary to effectively participate.

In this study, we explored the feasibility and limitations of recruiting crowd workers to complete complex engineering tasks. Our experience allows us to compile a list of useful suggestions for researchers who plan on using Amazon Mechanical Turk, or any similar engine, especially for more complex tasks. Given a well designed experiment, we found that such tasks can be accomplished in a crowd setting. However, specific effort must be made to train the crowd workers for the tasks and to maintain active lines of communication with the crowd workers. Increased task complexity will also result in a decrease in the number of crowd workers willing to perform the task.

## Methods

### Overview

We compared the skill of anonymous crowd workers of Amazon Mechanical Turk with the skill of trained graduate students in evaluating Virtual Wind Tunnel data. These graduate students were enrolled in departments of civil engineering, specializing in the study of civil infrastructure and related fields. To gain some insight into the crowd workers, we included a small number of survey questions in our Amazon HITs (Human Intelligence Tasks). For the first part of this study, we posted five survey questions and 102 graph analysis questions to groups of crowd workers with various levels of qualification: crowd workers with the masters qualification (established by Amazon), crowd workers who had completed at least 10,000 or more tasks within Mechanical Turk (known as HITs) with a 98% approval rating or higher, and crowd workers who have completed at least 1000 HITs with a 95% approval rating. We also had the aforementioned groups of civil engineering graduate students from two universities (a U.S. based institution and a second institution in China) complete our tasks.

The graduate students served as a control group for this study since they could be a skilled crowd for comparison. These engineering graduate students were not familiar with this particular task, but had a broad underlying knowledge of civil engineering. The responses of both crowds were compared to expert analyses of the same data sets by researchers with considerable expertise in CFD modeling and simulation. This expert analysis served as the "ground truth" against which we analyzed the participants' responses.

### Ethics Statement

This project was approved by the University of Notre Dame's Internal Review Board as protocol ID 14-06-1869. Crowdworkers were informed of the purpose of this project and their task as well as the compensation for the task before beginning. Crowdworkers were asked to give consent via a check-box if they understood the conditions of the task and wished to participate. This form of consent was approved by Notre Dame's Internal Review Board. This was the only form of consent which was given. Crowdworkers were asked to not participate if they did not agree with or understand the terms and conditions. After crowdworkers completed of the task, Mechanical Turk would return the results of the task to the researchers, and if the crowdworkers had checked the consent check-box then their data was used in the study. The consent of the crowdworkers was recorded with the rest of the data. Written or verbal consent was not required given that the crowdworkers were always kept anonymous and given that they completed all of the work over the internet. The crowdworkers were kept anonymous. Deception was not used in this study-1489666918. Our use of Amazon's Mechanical Turk for research purposes is consistent with the service's conditions-of-use as described in the Participation Agreement [20].

### Phase 1

**Fig 2:**
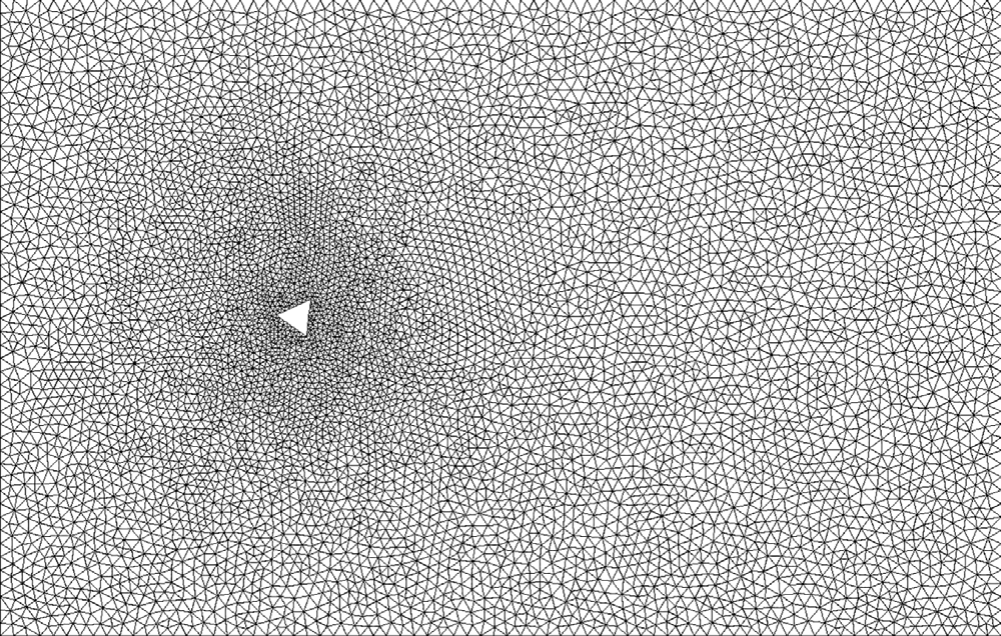
Mesh view of a simulation containing a triangular cross-section. This figure shows a mesh representing the area around a building's cross-section. The triangle in the center of the image represents the cross-section. The many smaller triangles represent “data points” or “pixels” in the image, each one will be a point at which velocity and pressure will be computed at each step of the simulation.

For the first part of the study, we asked crowd workers to evaluate various graphs produced by 2D Virtual Wind Tunnel simulations of the wind flow field due to a specified oncoming wind around buildings with various geometries. We ran all the CFD simulations in advance with a wide variety of parameters. One issue with CFD simulations is that it is relatively easy to produce a simulation that has no value for analysis either because the simulation failed to run or because it ran and produced results that do not correspond with reality. One major reason for this is how CFD models the fluid flow. CFD discretize the simulation domain as very small cells, to form the simulation mesh. The size and arrangement of these mesh cells affects the quality of the simulation. Our Virtual Wind Tunnel includes a tool that automatically constructs an unstructured mesh around the shape being analyzed, according to inputted parameters regarding cell size. Properly tuning these parameters is difficult. Cell size must be coordinated with the fluid flow and the simulation time step to produce accurate results. An example of such a mesh is in Fig 2. If this not done correctly, the results of the simulation will not be physically meaningful. Furthermore, the simulation must be run for a sufficient length of time for the flow to develop and for the results to be useful.

A CFD simulation generates information about the velocity and pressure of the fluid at each point in the simulation mesh. This can be visualized in various ways. We selected these results: two graphs and one image, which we used as the basis for the crowd tasks in this part of our study. These were an image of the wind velocity field, a graph of the wind velocity directly downstream of the building, and a graph of the lift coefficient over time. The key assessment requested from the crowd workers was whether the simulation that produced the presented graphs and image was either valid or invalid. An invalid simulation was one whose results should not be kept, i.e. would not warrant further consideration based on the indicators present in a tutorial. Similarly, valid simulations are at least potentially correct, given basic indicators, and warrant further consideration. This tutorial, given to the crowd workers, was designed to give a broad and basic understanding of the Virtual Wind Tunnel data as a whole as well as how to recognize faulty simulations. The indicators included in the tutorial advised crowd workers on what to look for in each graph and image to affirm validity.

### The Wind Velocity Field

**Fig 3:**
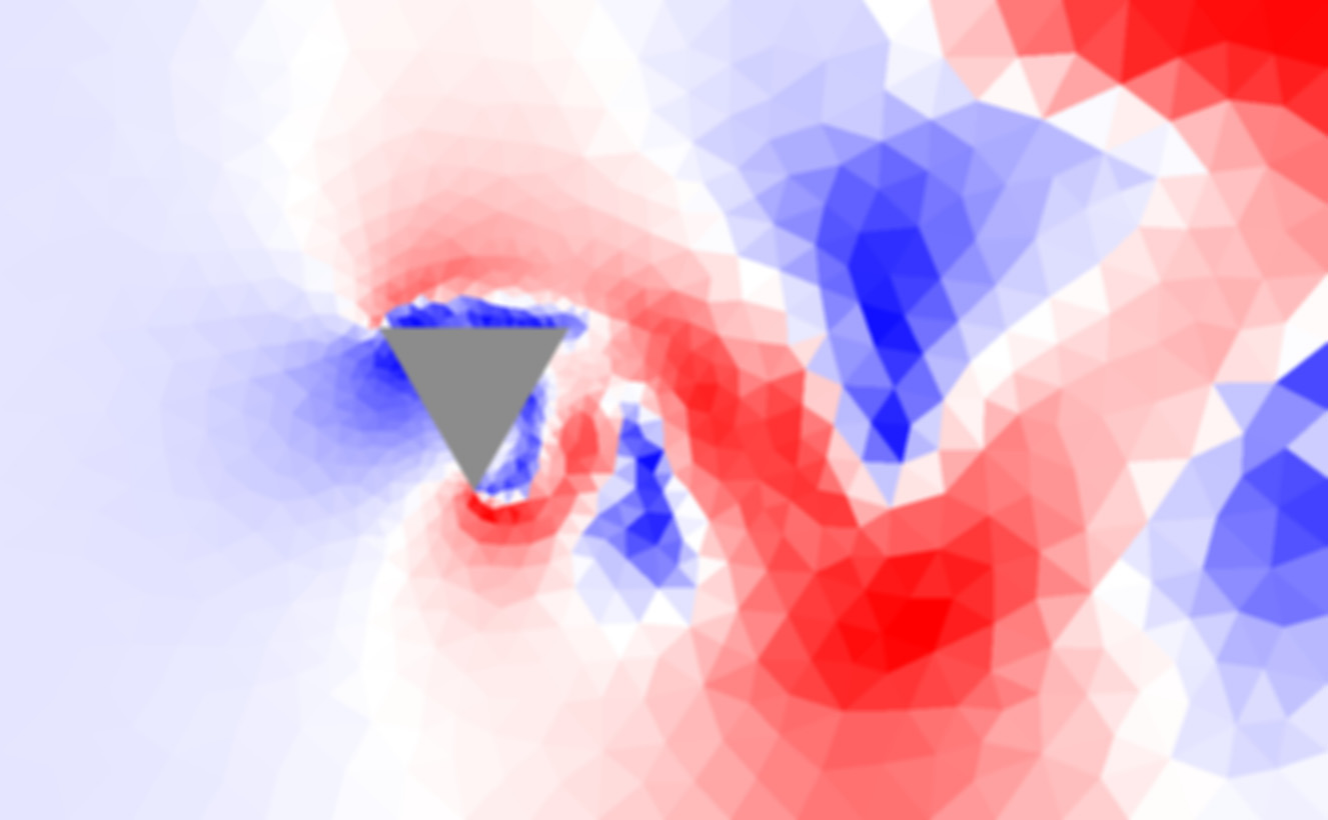
A flow visualization view of a simulation containing a triangular cross-section. This figure shows a colored flow visualization of the fluid velocity. The colors vary from red through white to blue. The red represents fast air, blue represents slow air, and white represents median speed air. This graph is meant to allow the reader to see the varying wind speeds around a structure.

The first of the three CFD outputs that the crowd workers assessed was an image of the Wind Velocity Field. Fig 3 shows a typical example of a Wind Velocity Field around a triangular building. The red areas show places where the wind is moving at high velocity. The blue areas show places where the velocity is low. The tutorial shows a Wind Velocity Field with super-imposed labels, with an explanation of the key elements in this image. In addition to these explanations, the tutorial presented indicators that reflected the validity of the simulation, e.g., if there is no oscillation of the flow (vortex shedding) in the region downwind of the building then the simulation is certainly faulty.

**Fig 4:**
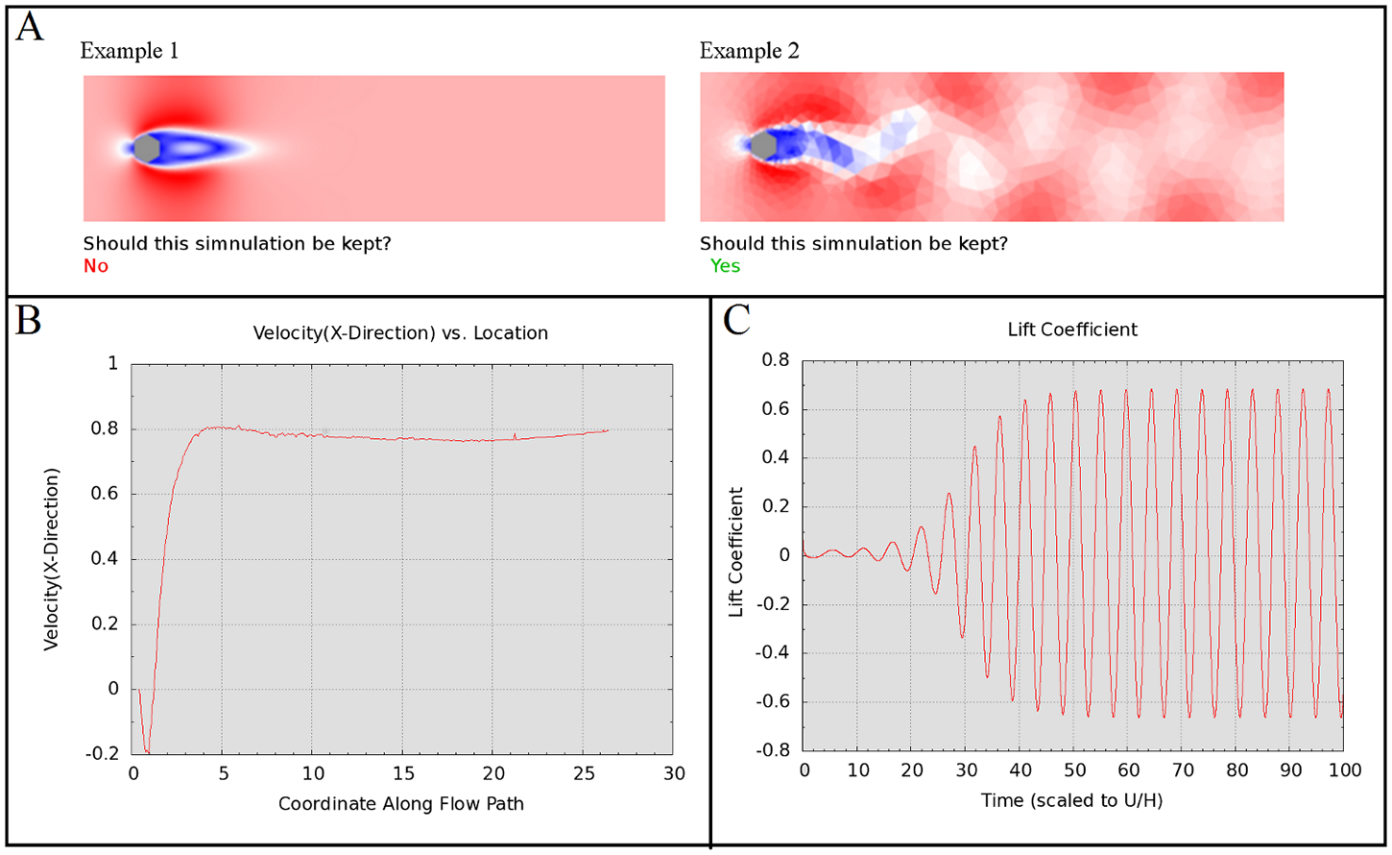
Four different graphs displaying wind tunnel data. A. Two flow view simulations contrasting a potentially useful, grainy simulation to a unhelpful, pristine simulation. B. A Velocity in the x-component versus location graph. C. A lift coefficient as a function of time graph. Fig 4A shows two sample graphs which were in the tutorial of the task. This is a prime example of how some simulations may be more visually appealing but do not show useful data. The first simulation shows a hexagonal cross-section building with a non-oscillating wake. The second simulation shows a hexagonal cross-section building with an oscillating wake. The first image is stated to be not worth keeping whereas the second is said to be worth keeping.

The tutorial ensures that crowd workers are trained to seek these indicators and are not biased by the coarseness of the resolution chosen. To achieve this goal, an example is presented where a high resolution simulation, producing a smooth wind velocity field, but lacking the oscillatory pattern is defined as "invalid". This is presented alongside low resolution results with a comparatively coarser wind velocity field displaying the desired oscillatory pattern and is thus classified as valid. The tutorial goes on to explain why the former case should not be accepted but the latter should be. These two examples are shown in Fig 4A.

### The Mean Velocity as a Function of Position Downstream Graphs

The next graph we presented shows the mean velocity (or more precisely, the x-component thereof). The image shows the velocity of the wind (on the y-axis) in units of meters per second (m/s). A greater x-value on the graph denotes a position further downstream of the building, in the "wake" of the building. The x-axis indicates the position where this velocity was simulated in meters. A circular building used in some simulations was superimposed at the bottom of the graph to demonstrate this more clearly for the tutorial example. Fig 4B shows a typical Mean Velocity graph. It is a simplified way to determine how large the wind velocity is in the wake region and how that velocity changes as you move further away from the building.

Fig 4B shows a Mean Velocity as a Function of Position Downstream graph. The x-axis shows the location of the wind along the flow path. The y-axis in this image shows the velocity of the wind in units of meters per second (m/s). And all numbers in x-axis are in meters.

The crowd workers were told to keep the simulation if the Mean Velocity curve decreases toward a negative value and then increases until it eventually “plateaus” or becomes nearly flat (constant). This nearly flat region could have some slight variations, but should not show significant increases or decreases once it reaches this plateau. The crowd workers were informed that only simulations where the Mean Velocity achieved a constant plateau at some point downstream should be kept.

### The Lift Coefficient as a Function of Time

The final graph described in the tutorial was the Lift Coefficient plotted as a function of time. Fig 4C shows a typical Lift Coefficient graph. The Lift Coefficient is one measure of the wind force on the building. The graph shows the Lift Coefficient on the y-axis (the coefficient has no units). The x-axis indicates the time during the simulation when this value is measured. This time is scaled according to the velocity of the wind and the size of the building. In this case, one unit of time is equal to the amount of time that wind, at inlet velocity, takes to travel the full width of the object.

Fig 4C is a Lift Coefficient as a Function of Time graph. The lift coefficient is one measure of the wind force on the structure. The image shows the lift coefficient on the y-axis (the coefficient is dimensionless). The x-axis indicates the time at which this lift coefficient is measured rescaled according to the velocity and size of the figure. For example, the x-axis coordinate at 10 corresponds to the time it takes for wind, unimpeded from the inlet, to travel ten times the width of the figure.

The crowd workers were informed that the graph was acceptable if the Lift Coefficient increases until the peaks of the oscillating pattern have nearly the same positive or negative value. That is, the oscillation should, as the simulation runs, eventually reach a consistent state. Since the velocity of the oncoming wind in the simulation is constant, the Lift Coefficient should tend to increase and stabilize: its peaks remaining near the same positive and negative values all the way to the right edge of the plot. They were told that if the Lift Coefficient’s peaks do not stabilize to approximately the same constant positive and negative values then the simulation should not be kept.

### HIT Design

**Fig 5:**
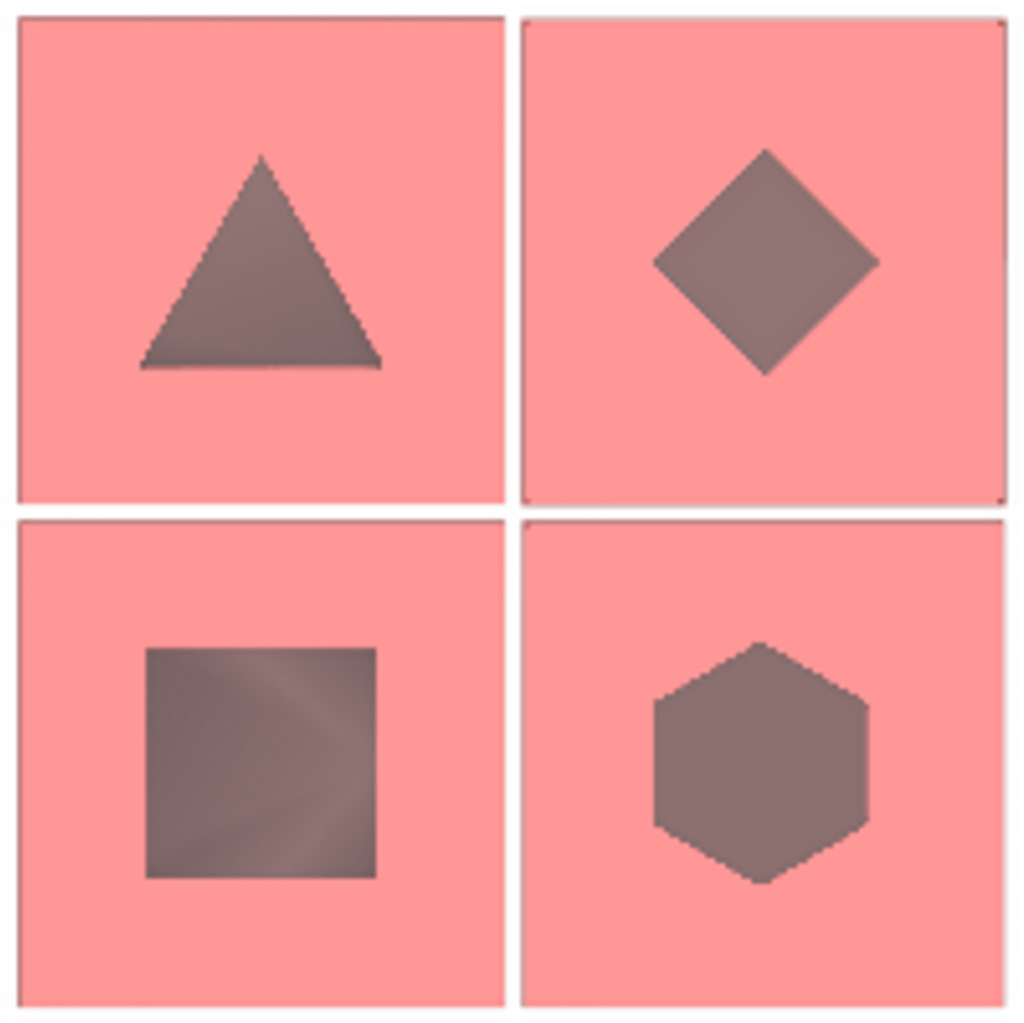
Four different cross-sections of buildings. The four images in this figure are shapes of buildings when viewed from the above. The cross-sections included are four different building shapes: a triangle, a diamond, a square, and a hexagon. This was shown in the tutorial and is called the plan view.

In Mechanical Turk, individual tasks are known as Human Intelligence Tasks, or HITs, and our first part of the study was divided into several HITs. This first HIT contained the aforementioned tutorial, a short survey and nine questions regarding the trio of output graphs and images described above. The subsequent HITs contained the same trio of outputs from thirty-six additional simulations. The tutorial explained the job of the crowd workers: to identify which simulations should be kept, based on the indicators in the three outputs. This allows the crowd workers to assist the expert by making a first pass through the data and rejecting simulations that are clearly unreliable. The tutorial also explained that buildings are represented in the 2D simulations from the top down view, known as the plan view. The four example "buildings" represented from the plan view can be seen in Fig 5. By simulating the wind flow around these cross sections, experts can develop a better understanding of how wind "pushes" on these buildings. In real life, the knowledge of this sort of wind interaction, which tends to be gained by studying scaled models in a wind tunnel, forms the basis of the loading scenarios that the building must be designed to resist.

### Number of Recruits for Simulation Analysis

Sixty-six "Master" (according to Amazon's Mechanical Turk's classification) workers completed our first HIT; 59 of them were qualified to move on the next HIT; 36 finished all the available HITs. Fifty-one non-Master crowd workers completed the first HIT, 27 were qualified to move on to the other HITs, and 9 finished all the HITs. These results were gathered in June 2013. In 2014, we re-released the same HITs and an extended survey. This time around 60 Master crowd workers and 150 crowd workers with the "10,000 HITs or more and 98% qualification" completed the first HIT.

### The Survey and Questions

**Fig 6:**
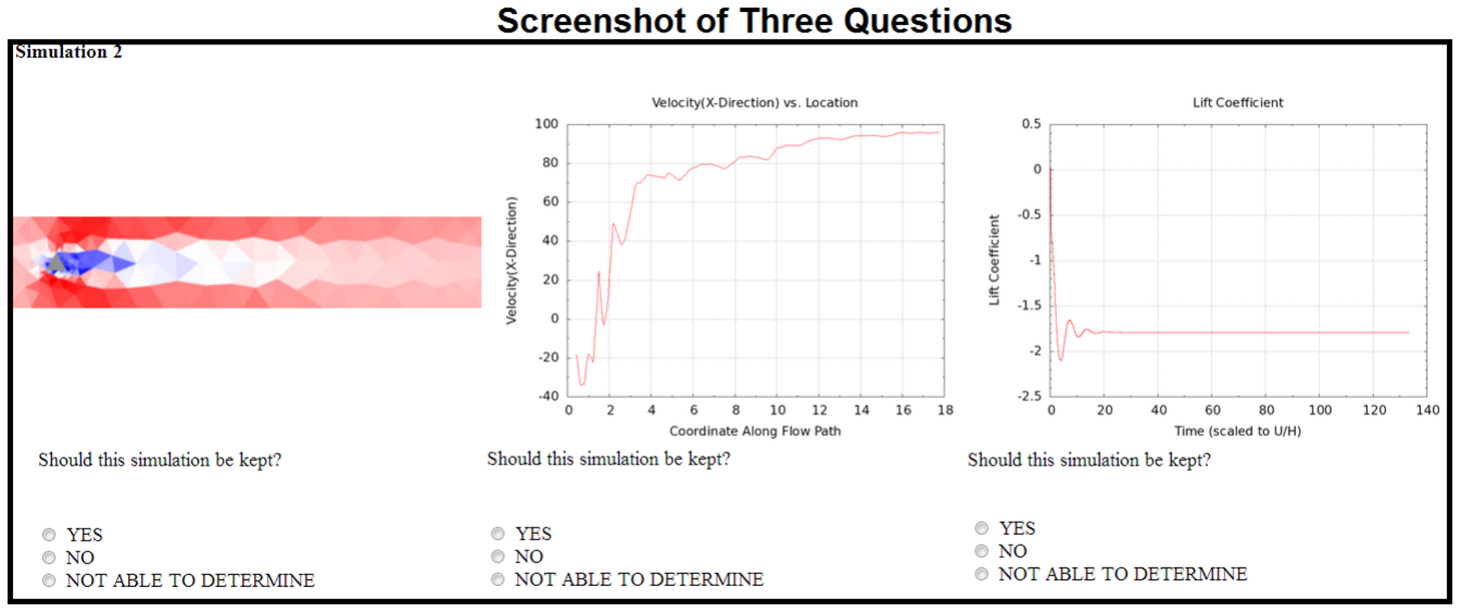
A Screenshot of three questions all pertaining to different aspects of one simulation. This image is a screenshot of three questions which the crowdworkers would be asked. Each of the questions pertains to different parts of the same simulation. This particular image was shown at the beginning of the tutorial which educated the crowdworkers on how to complete the task.

After the tutorial, crowd workers were presented with a five question survey, which asked questions regarding their gender, age country, education level, and motivation for doing this work. The trio of images from one simulation's results were presented following the survey. Crowd workers were asked three specific questions about the images. For each of the result's graphs/images, the worker could select "yes", "no" or "unable to determine" in response to the question regarding whether the simulation should be kept. If a worker selected "unable to determine," we included a text box where they could explain why, or give any other feedback they wished. An example without this text box is shown in Fig 6.

### HIT Payments and Posting Time

The reward for the first Masters HIT was $2.50, and the reward for the first non-Masters HIT was $2.00. The later twelve HITs paid Masters $0.25 and non-Masters $0.20. This decrease in price from $2.50 and $2.00 to $0.25 and $0.20 was chosen because the later twelve HITs only contained nine quick yes or no questions, whereas the first HIT contained a long tutorial, a survey and the nine yes or no questions. The estimated maximum time allotted per assignment for the first HIT was three hours and the time allotted per assignment for the later HITs was one hour. Each HIT expired after fifteen days if it was not completed. The Masters HITs were published on Amazon Mechanical Turk from June 12, 2013 to June 27, 2013. This HIT was left open for any crowd workers with the Masters qualification to complete. Fifty non-Masters HITs with the aforementioned qualifications were posted. Thirty were posted June 25, 2013 and twenty were posted on June 28, 2013.

### The Graduate Students

Eleven graduate students studying civil Engineering at University One (a U.S. based institution) were also asked to complete all the HITs. Eight Graduate students from a University Two (a University in China) also participated in the experiment. Amazon Mechanical Turk did not allow the students from University Two to work from the website, for reasons they did not state, but seemed to be due to legal issues. The project was then transcribed into SurveyMonkey for these students. The students were given all the same questions and were given access to the same introduction, tutorial, and survey. The only difference was the platform used (Mechanical Turk vs. SurveyMonkey). For further details on the submissions of the graduate students, see S1 and S2 Datasets.

### Communication with the Crowd Workers

We communicated with the crowd workers in many ways. First, the crowd workers had the opportunity to send messages via the comment boxes in the HITs. Mechanical Turk also allows crowd workers to send Personal Messages (PMs) to requester. These were received via email. We also set up accounts on Turkernation and CloudMeBaby, which are forum websites designed for communication among crowd workers and employers. We posted our HIT’s information and introduced ourselves on these sites. Many crowd workers try very hard to keep their approval ratings high for qualifications, so working for a new requester can be a risk, because some new requesters reject a lot of HITs not understanding and/or caring about the damage it can do to crowd workers' ratings. Therefore, introducing ourselves and welcoming open communication made more crowd workers comfortable with considering and working on our HITs. If crowd workers completed our HITs, they were invited on to later HITs. These invitations were distributed via $0.01 bonuses with messages attached. This was done both to compensate them for the time to read all of our messages, but also because Amazon does not provide a simple way to communicate with crowd workers other than through tasks or in response to communication which was initiated by the crowd worker.

### How Many HITs Were Completed and How Fast?

In 2013, both of the non-Masters HITs attracted their full allotment of workers within three and a half hours of publishing. Sixty-six "Masters qualified" crowd workers completed the first HIT. Fifty-eight of the sixty six master crowd workers were invited on to the next five HITs. Forty-nine Masters crowd workers went on to complete the next five HITs. All of the crowd workers who completed the first six HITs, and participated in good faith, were invited to complete the final seven HITs. Thirty-two Master crowd workers completed all thirteen HITs. Fifty non-Masters crowd workers completed the first HIT. Twenty-seven of these crowd workers were invited to the next five HITs. Sixteen of these non-Masters crowd workers went on to complete the next five HITs. All of these crowd workers were invited to complete the next seven HITs. Nine of the non-Masters crowd workers, who were invited, completed all thirteen HITs.

In 2014, both of the HITs attracted their full allotment of workers in under 18 hours. Both 2014 HITs were released at approximately 20:00 GMT. One hundred eleven non-Masters out of 150 returned to complete the other work, and 44 out of 60 Masters returned to complete the other work. All the extended work was left open for crowd workers to complete for nine days after the initial HIT was released. After crowd workers completed the first HIT, they were invited to complete all the remaining HITs. For further details on the submissions of the crowdworkers, see S3, S4 and S5 Datasets.

### Demographics

**Fig 7:**
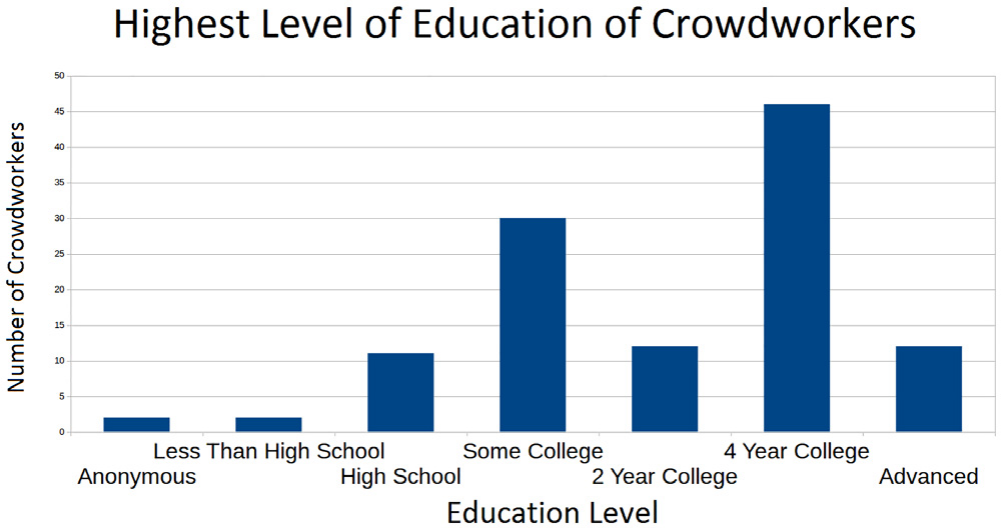
Histogram showing the highest level of education of crowdworkers. This image is a histogram showing the differing levels of education which the crowdworkers reported to have. This information includes the crowdworkers from the 2013 and 2014 test groups who completed the first HIT; this is 336 crowdworkers.

Based on collected survey data, the average weekly income for our crowd workers from Mechanical Turk was just under $200 and the average crowd worker total income from all sources was approximately $26,000 a year. From this we can deduce that the average crowd worker who worked for us gained more than 1/3 of their income that week from Mechanical Turk. The majority of our crowd workers said that their main reason for participating in the work was for the income it provided. In addition, over 80% of our crowd workers were from the United States. As can be seen in Fig 7, despite the low average income of crowd worker, over 85% of our crowd workers participated in at least some college and over half earned 4-year college degrees. These statistics show us both that the crowd workers we attracted were, in general, well-educated and relied on Mechanical Turk as a source of income. Mechanical Turk contains formally educated workers willing to crowdwork diligently for relatively modest compensation but nevertheless a strong monetary motivation. For further details on the data, see S6 Dataset.

### Percent Agreement with Ground Truth and Majority Consensus

**Fig 8:**
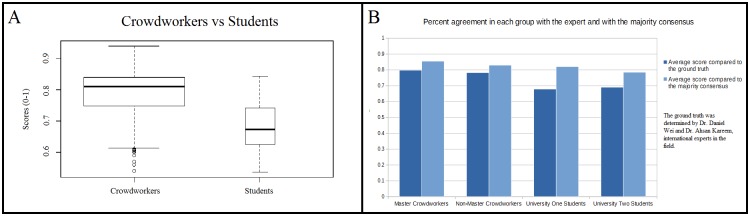
A. Histogram showing the percent agreement in each group in comparison to the majority consensus and with the expert. B. Box and whisker plots comparing 215 Crowdworkers to 19 Students in performance. Fig 8A is a histogram which compares each group's answers with the majority consensus and with the expert. Only crowdworkers who completed all thirteen HITs with twelve or fewer missing answers were included in these calculations. The most common answers of each group was calculated, this set of answers was called the majority consensus. A higher majority consensus indicates a higher group consistency. In Fig 8B, all crowdworkers who completed all thirteen HITs with twelve or fewer missing answers were included in these calculations. Performance was measured as the score of each participant as compared to the expert. The widths represent the number of people in each group. The y-axis represents the percentage of agreement with the expert “ground truth” on whether to keep a given graph. The average crowdworker's “score” (~79%) is higher than that of the graduate student “score” (~68%).

In order to determine ground truth, an expert in CFD performed analysis on the same collection of simulation results. We compared these to the assessments of the crowd workers to determine the crowd worker's score. Only the answers of crowd workers who completed all thirteen HITs were considered in these comparisons. The average score of the Master crowd workers was 79.79% agreement with ground truth. The average score of the non-Master crowd workers was 78.25%. The average score of the University Two Graduate students was 69.04%. The average score of the University One students was 67.91%. Each group’s majority consensus was then calculated. Majority consensus is the median response of the group. The Master crowd workers, University Two Graduate Students, University One Graduate students, and non-master crowd workers each had their majority consensuses calculated individually. The Master crowd workers, University Two Graduate Students, University One Graduate students, and non-Master crowd workers scores when compared to their respective majority consensuses were 85.46%, 78.56%, 82.06%, and 82.97%. These statistics are all represented in Fig 8A. Given the small number of graduate students at the two schools who finished all the HITs, we combined the crowd workers (Master and non-Master) into one group of 215 participants and the graduate students (University One and Two) into one group of 19 participants. A Welch two sample t-test shows a statistical difference (with a p-value of 2.339e-05) between the two groups. See Fig 8B.

### Comparing the “Generosity” of Each Group

We calculated how often each group said that a simulation should be kept. We refer to this as the generosity of the group. The more often a group stated that the simulation should be kept, the more generous the group. The ground truth ratio of yes to no was 3.00 to 1. The University Two students' ratio was 1.89 to 1. The University One students' ratio was 2.36 to 1. The Masters ratio was 2.70 to 1. The non-Masters ratio was 2.44 to 1. All of the generosity calculations for the crowd workers were made only using the answers from the crowd workers who completed all the HITs and supplied ten or less "Not Able to Determine" answers or non-responses.

## Discussion

The crowd workers performed very well, even surpassing the expert crowd of graduate students. This is especially impressive given their relatively small amount of training. This performance was again determined by comparing crowd inputs to ground truth, as determined by an expert post-doc and expert faculty member. The differing “generosities” show that the students were more “biased” toward answering no than the crowdworkers and as a result matched the expert's data less than the crowdworkers. This may be because the students had a preconceived idea for what constituted “data worth keeping”, and the crowdworkers, not bringing any preconceptions, more closely matched the expert's idea from the tutorial. Although the crowd workers' average score was higher than that of the formally trained graduate students by a statistically significant amount, the small sample size of graduate students cautions us against over-interpreting the results. However, the purpose of the study was to test if crowd workers are a viable resource for completing engineering tasks with efficiency and reliability, and this was confirmed.

One difference between the two groups of crowd workers (the volunteers from Mechanical Turk and the graduate students) should be acknowledged. The Mechanical Turk workers were allowed to quit if they were uninterested in the task. The graduate students were assigned the work by their professor. This increased care and enthusiasm on the part of the volunteers might have offset the greater training of the graduate students. That is, the graduate students were formally trained, but possibly less motivated to do well. Meanwhile, the crowd workers that persisted were not formally trained, but were possibly more interested. Of course, as we said before, one of our experts reexamined the data following the crowd responses and changed his mind about one simulation's validity. In effect, a large set of careful, though not expert, eyes double-checked his work and enabled the identification of his own mistake.

The ability of crowd workers to quit if they wanted to could have “filtered out” all of the uninterested, less careful crowd workers. This “filtering” is one of the great advantages of crowdsourcing. However, in our case, due to the relative complexity of the task, this was also a disadvantage for us, as it limited the pool of workers completing all the tasks, which had a significant impact in the second stage of this study and the statistical inferences we could draw.

## Masters versus Non-Master Crowd Workers

In 2013, the length of time it took for the first HIT to attract their full allotment of fifty non-Masters was three hours compared to the Masters who completed the first HIT fifty times in nine days (it took fifteen days to complete all sixty-six). This is most likely due to the relatively small number of Master crowd workers available on Mechanical Turk. Also, the fact that Master crowd workers have access to more HITs would result in a decreased likelihood for them to choose to complete our HITs. In 2014, it took approximately eighteen hours for both 60 Masters crowd workers to complete the first HIT and for 150 highly qualified crowd workers to complete the first HIT.

## Phase 2

To follow up the first phase of this experiment, we held a follow-up experiment which involved 32 participants from the previous experiment. While Phase 1, previously described, explored the ability of crowd workers to recognize if a CFD simulation was generating plausible results, Phase 2 explored the ability of crowd workers to generate good simulations. For Phase 2, we created an interface to our Virtual Wind Tunnel application, specifically simplified for this experiment. Crowd workers were given a building to analyze. Specifically, they were instructed to perform the best simulation they could upon it. Crowd workers were allowed to alter some of the CFD simulation parameters and were expected to use the skills they developed in Phase 1 to generate high-quality results through trial and error. Crowd workers were paid as usual, a sum based upon how long we expected the task to take: $20.00. In addition, within each group (crowd workers and expert students) a prize was offered for the best simulation: $100.00.

In order to help crowd workers gain some understanding of how specific parameter tweaks affected the results, three simplified exercises (optional) were provided that isolated particular parameters. Users could manipulate: the size of the simulation domain, the density of the mesh and the simulation time-step and duration. The building being analyzed, as well as the velocity and direction of the wind were kept constant. Crowd workers were allowed to manipulate all of these parameters, running as many simulations as they would like. Crowd workers were then instructed to mark one of these simulations as their best, for submission.

## Discussion

This follow-up experiment significantly pushed the limits of what "crowdsourcing" is typically expected to do. Most crowdsourcing relies upon the relative simplicity of the proposed task or the ability to break a task into small pieces. We were asking crowd workers to perform a task that required experimentation with simulation parameters and repeated analysis. This had two immediate consequences. First, the participation rate was dramatically lower than for the previous stage. This had consequences in limiting statistical analysis. Second, there was a fairly wide variation on the amount of time which the various workers were willing to spend interacting with our system. Some only made a few tries to get a good simulation, while others spent a long time trying many different possibilities. We suspect that some of the crowd workers were even taking the opportunity to enjoy using our tool to have some fun.

Between the three main groups of workers, the anonymous crowd workers and the graduate students from the two universities, there were slight variations in the visible effort made, measured by the amount of time spent logged into the Virtual Wind Tunnel and the number of simulations run to completion. Overall, all crowd workers submitting reasonable results averaged 21.3 completed simulations. (Not counting simulations for the simplified optional exercises.) The crowd workers ran a few more, averaging 28.9, and the two groups of graduate students ran a few less, at 12.2 and 18.2, respectively. However, there were many outliers, with some workers running as many as 130 simulations to completion. Overall, the median was 8, which appears to be typical.

The time spent logged in was similarly variable. We note, however, that even if a crowd worker was logged in, that does not mean they were actually working. They might have been doing other things while waiting for a finished simulation. Further, crowd workers were allowed to logout and come back later, and simulations would continue to run. On average, all workers spent about 9.4 hours logged in, though again, with significant outliers, with one person logged in for over 2 days. Again, the median was more typical, at 5 hours of login time. Overall, a typical crowd worker spent about 5 hours logged in, and ran 8–10 simulations, usually after some time doing the exploratory exercises. However, many crowd workers spent much more time, running many dozens of simulations.

In the previous phase we could easily quantify each participant's performance with a "score" for comparison. In this situation, however, it is not nearly as straightforward to compare the performance of each group. To made a useful comparison, we can classify the submissions of each user into one of three categories, based upon the three images used in Phase 1, which workers could see for their simulations in Phase 2. We describe "good" simulations as those for which the three images obviously and closely conform to the described parameters. If one of the three images are similar to what it should be but differs sightly we will call the submission "fair". Simulations for which at least one of the three images is significantly and clearly wrong are called "poor".

In this phase, we see a similar result as in phase 1. The crowd workers, drawn from the willing public, performed similarly to the graduate students. Among the 17 graduate student submissions, 8 were "good" (47%), 4 were "fair" (24%) and 5 were "poor" (29%). The authors' home institution however, did somewhat better, with 5 "good" (62%), 3 "fair" (38%) and 0 "poor". We suspect that the direct proximity of their advisor, who assigning them the task, might have had a motivating effect. Among the crowd workers, out of 14 submissions, there were 8 "good" (57%), 3 "fair" (21%) and 3 "poor" (21%). For further details on the submissions of the crowdworkers in phase2, see S7 and S8 Datasets.

**Fig 9:**
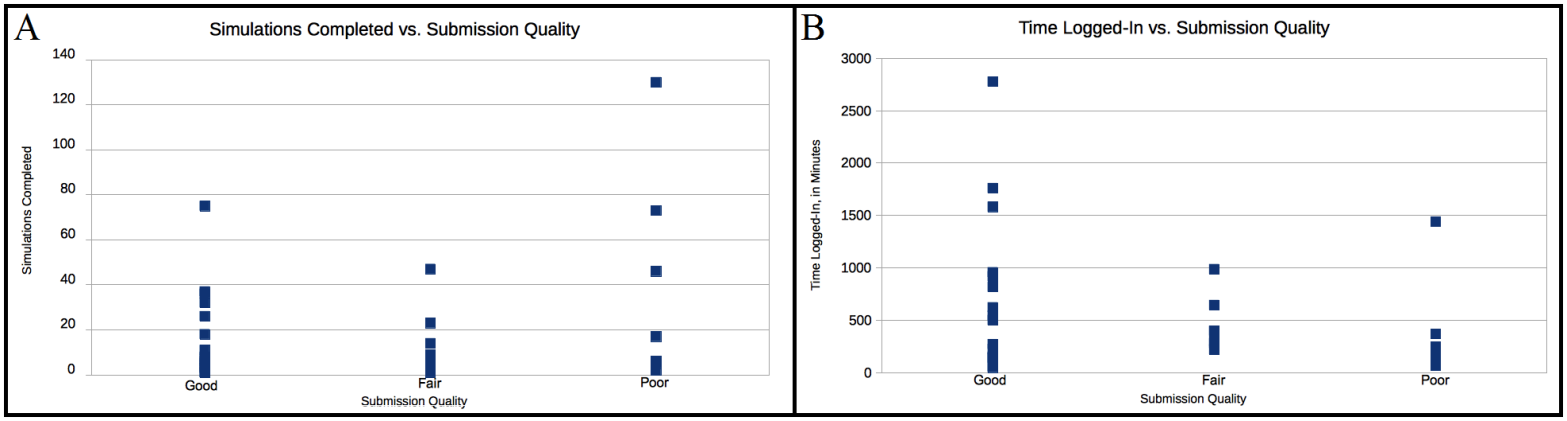
A. Phase 2 submission quality vs. the number of simulations run by participant. B. Phase 2 time logged vs. submission quality. In Fig 9A we show the number of simulations which a participant ran before submitting one, compared with the quality of their submission. The quality was subjectively determined as “good”, “fair” or “poor” according to the images and results in a manner similar to Phase 1. A look at the graph shows that better results seem to weakly coincide with more simulation attempts, but not without significant outliers. In Fig 9B we show the amount of time for which a user was logged-in, compared with the quality of their submission. The quality was subjectively determined as “good”, “fair” or “poor” according to the images and results in a manner similar to Phase 1. The time is measured in minutes. A look at the graph shows that better results seem to weakly coincide with more time spent, but not without significant outliers.

We also considered the relationship between the quality of submission with the time spent logged in and the number of simulations run to completion. It is intuitive to expect that better submissions will be submitted by persons expending more time and performing more simulations. In Fig 9A, we show the relationship between submission quality and the number of simulations completed. In Fig 9B, we show the relationship between submission quality and the time spent logged in. A visual inspection of both these graphs shows some evidence to the expected trend: The "poor" submissions have a bunch of results with few simulations and low time spent. However, this overall picture is clouded by many outliers. As such, this is not a direct relationship. This fact underlies why this problem is difficult for both automation and traditional crowd techniques. More, in the form of brute-force attempts, is not always better. However, more attempts, combined with careful thought, seems to help.

As the project progressed from Phase 1 to Phase 2 the amount of personal interaction between the crowdsourcer and the crowdworkers increased significantly, as crowd workers began asking more questions and contacting us more frequently. This could potentially be expected with any experiment where the complexity is great. As we progressed into Phase 2 of this study, our interactions were more akin to outsourcing than crowdsourcing. The main difference between these two modes of work is that outsourcing is much more like a typical occupation, with frequent communication and updates between the employer and worker. This back-and-fourth is a very different dynamic than the crowdsourcing dynamic, in which a task is given, then returned. This outsourcing is possible in the Mechanical Turk marketplace, but the engine is not optimized for such activity and as a result most personal interactions take place via email.

### Suggestions and Lesson Learned

We spent over 60 hours reading through Mechanical Turk Forum and Turkernation threads, reviews, and general discussions. We also spent many hours on Turkernation’s chat room. Crowd workers used the chat room mostly for sharing news about new HITs and discussing different requesters. We gleaned a few notable ideas through this correspondence and from personal messages sent to us by crowd workers. One is that there is a general distaste among the Mechanical Turk community for the use of the Master qualification, partly because many of them cannot figure out exactly how to become “qualified,” as Amazon does not share how the process works. Another lesson is many crowd workers see requesters who use the Masters qualification as “foolish” given their belief that the Masters qualification is costly without tangible benefit. More experienced crowd workers often become upset with newer crowd workers who will work for low paying requesters or those who are poor communicators. We found that many crowd workers were suspicious of new requesters and high paying HITs. Turkopticon is a favored requester rating site for crowd workers. This site helps show what crowd workers believe is fair pay, how much communication is acceptable, the regular HIT acceptance speed, and the fairness of rejection rates.

If a researcher is going to use Mechanical Turk, we suggest a few things:

1. Test your HIT by releasing a version where the description of the HIT is “test this HIT” and just ask about 5 crowd workers to check your HIT for typos, and fill out all the answer boxes. Then just check the data you get back to make sure the code functions properly.
2. Use Qualification HITs. This will be the first HIT you release. Only allow crowd workers who complete this HIT to move on to later HITs. All qualification tasks should be approved. That is, the crowdsourcer should mark the crowdworker's work as "approved". But, only crowd workers you want should be allowed to continue. This allows you to avoid rejecting the work of crowd workers, which creates ill will, while still not having to retain low quality crowd workers. (Crowd workers are very suspicious of potential rejection, as they don't want it to negatively impact their stats.)
3. Using the Number of HITs qualification (>10,000 hits completed) combined with the HIT approval rate will give very high quality crowd workers. However, if you have a massive amount of HITs to be completed quickly you may have to lower the qualifications.
4. One option is to use the Masters qualification but only on the qualification HIT as in point 2. Then, after crowd workers are given your personalized qualification, you can take off the masters qualification knowing that all of your crowd workers are Masters and avoid paying the extra money that Mechanical Turk charges for using the Masters qualification. But be cautious, we observed a strong negative feeling in the Mechanical Turk community with regards to this qualification.
5. Use applicable social media, (such as Turkernation) to introduce yourself to workers and say that you will reward good workers and not reject sub-average workers (unless they are obviously not participating in good faith). Be watchful on such forums for questions from workers.

## Conclusions

Given a sufficiently comprehensive tutorial and compensation, under certain circumstances, crowd workers can and will complete long and sometimes complex engineering tasks with comparable competence to that of formally trained persons. This can be used to supply a significant group of trainable assistants for experts to use to prescreen or check their own work.

When giving such high difficulty tasks, one must be cognizant of the qualification limits placed on workers allowed to do these tasks. In Amazon Mechanical Turk, if one is using the Masters qualification, the time it takes for getting workers to complete your tasks is significantly increased since there are fewer available workers. However, Master crowd workers are significantly less likely to “spam” your HITs than non-Masters crowd workers. Master crowd workers also have a higher return rate than non-Masters.

The data in this experiment encourages the use of crowd workers as trainable assistants for complex engineering tasks. Tasks, such as surveys, and data analysis can be done quite easily on the Mechanical Turk with fast and accurate results if one is using the correct qualifications and has a sufficiently well-designed tutorial. If one asks crowd workers to use complicated, unfamiliar tools, this may result in significant increase in the need for communication between the crowdsourcer and the crowdworkers, but such tasks can be done.

### Future Work

Given the outcome of this experiment, we are interested in studying the effectiveness of crowd workers on other complex engineering tasks. One option is to consider pushing the complexity of the tasks even more, such as by asking crowd workers to design their own buildings using Google Sketchup or a similar program and then analyzing them. However, this runs the risk of further pushing the experiment into outsourcing, rather than crowdsourcing, and only gaining a limited pool of crowd workers, which would have to be trained even more and provides less of a basis for consensus.

If, instead, we wish to remain in crowdsourcing, we see three main avenues of exploration. We can explore other high-complexity tasks. For example, we can ask crowd workers to help in the processing and analysis of post-disaster reconnaissance images gathered by apps such as CyberEye [21]. We could also investigate the internal dynamics of crowdsourcing itself, by studying the effectiveness of introducing requesters on networks such as Turkernation, Mechanical Turk Forum, and CloudMeBaby.

One potentially interesting avenue of investigation is a hybrid model which includes automated computers, crowd workers and experts. In one particular design, we can have computers automatically perform many simulations, of fairly coarse quality over a range of parameters, on designs proposed by experts. Then, we can ask crowd workers to evaluate the quality of those simulations. Using the crowd results, we can further refine the simulations, producing another set of simulations of finer detail over the parameter ranges garnering crowd consensus. We can then repeat this until we have a simulation of the precision desired, which could be further refined by an expert.

We would argue that one of the most interesting aspects of this entire study is that we have begun to more greatly appreciate the considerable potential of crowdsourcing, both as a subject of study and as a means of accomplishing more and more complex tasks. It is our hope that, as we begin to further understand the potential of this tool, we can more effectively deploy it for some complex, real world, engineering problems.

## Supporting Information

S1 Dataset Response data for grad student group 1 to Phase 1 questions. Some questions are removed due to personally identifying data. 0 = Response is "reject simulation". 1 = Response is "to keep simulation". 2 = Response is "unable to determine".
(CSV)

S2 Dataset Response data for grad student group 2 to Phase 1 questions. As explained in the paper, this data is in a slightly different format than the previous dataset. But the image names can be used to correspond to the other data sets.
(CSV)

S3 Dataset Aggregate scores of all participants. This file contains the percentage "scores" (relative to ground truth) of all participants who completed all tasks in phase 1 (up to 12 "Unable to determine" or blanks allowed). Headings Legend: MT—Master Turker. NT—Non-master Turker. U1—Grad student group 1. U2—Grad student group 2.
(CSV)

S4 Dataset Response data for master turkers to Phase 1 questions. 0 = Response is "reject simulation". 1 = Response is "to keep simulation". 2 = Response is "unable to determine". There were two groups of master turkers surveyed, one in 2013, and one in 2014. The beginning of each group is marked.
(CSV)

S5 Dataset Response data for non-master turkers to Phase 1 questions. There were two groups of non-master turkers surveyed, one in 2013, and one in 2014. The beginning of each group is marked.
(CSV)

S6 Dataset Demographic and motivational data for crowd workers who completed an extended demographic survey. For survey questions, all column headings explain the questions asked. For survey questions, options to not answer or answer "other" were given. Some questions are removed due to personally identifying data. Also contained in this file are aggregate statistics for crowd workers from the 5 survey questions which were asked of all participants.
(CSV)

S7 Dataset Summary data for the submission quality for Phase 2. (CSV)

S8 Dataset Aggregate summary of the interactions of users with the Virtual Wind Tunnel during phase 2, for users who made a submission. (TXT)

S1 Images Folder Images for the users to evaluate in phase 1. Image names are the column headings for and pertain to data in S1, S2, S4 and S5 Datasets.
(ZIP)

S2 Images Folder Set of folders containing the key images, extracted from the simulations submitted by users for phase 2. The numbers for each folder corresponds to the user number in phase2reports.txt. The Virtual Wind Tunnel does not generate images unless a user asks for a particular image. On a few occasions, users did not even look at certain graphs, so those graphs were not generated. Such non-inspected graphs are not present here. (Note: the x-axis label in the wake stream velocity graph, in Phase 2, due to a typo, indicated a scaling factor that was not actually applied.)
(ZIP)
