# Supplementary figures and images for: Lessons Learned from Crowdsourcing Complex Engineering Tasks (part 2 of 3)
Source: PLoS One. 2015 Sep 18;10(9):e0134978. doi: 10.1371/journal.pone.0134978 (PMC4575153; doi:10.1371/journal.pone.0134978)

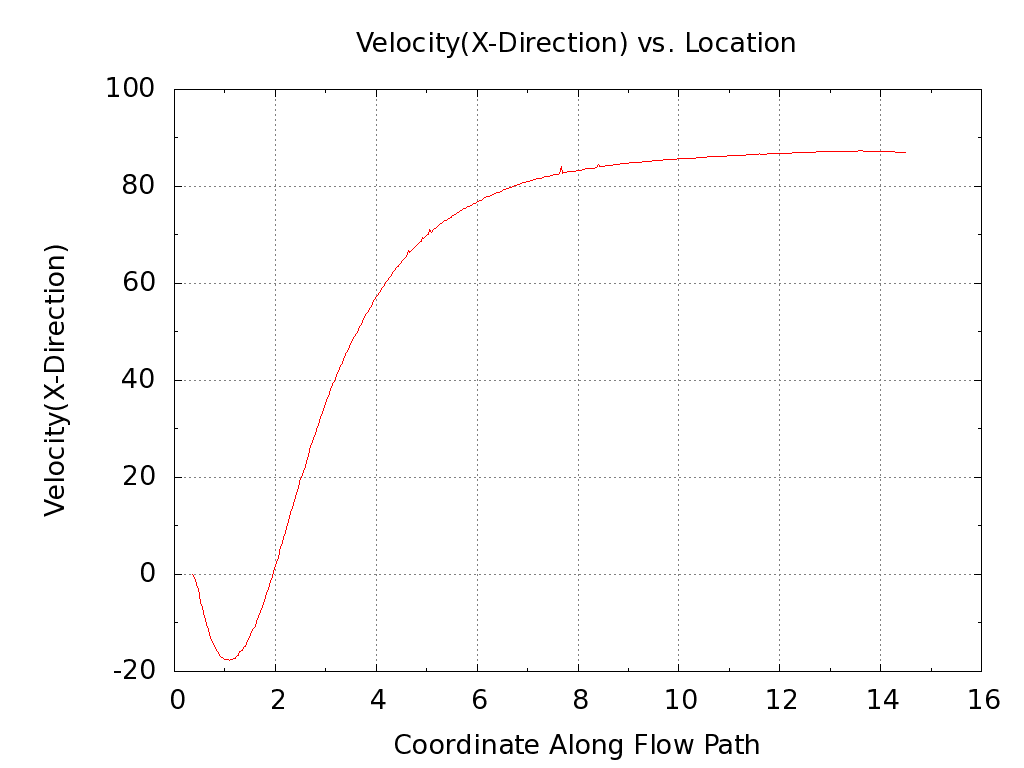

Supplement: S1 Images Folder — Image names are the column headings for and pertain to data in S1, S2, S4 and S5 Datasets. (ZIP) [file pone.0134978.s009.zip › S1_imagesfolder/Dur1_C.png]

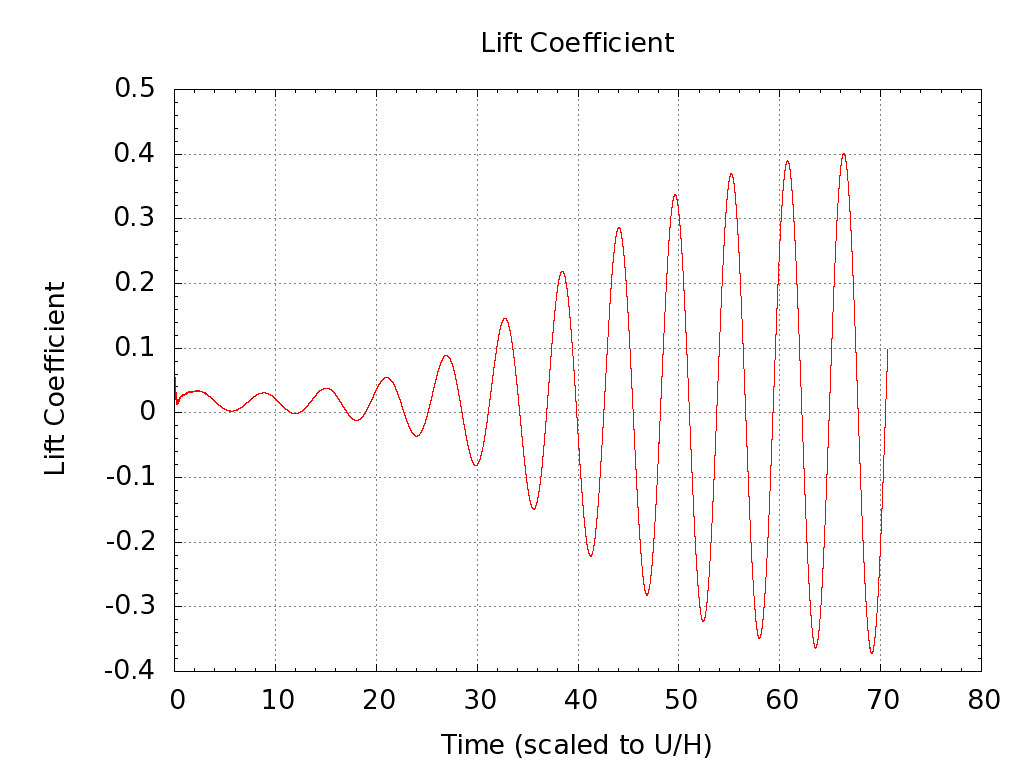

Supplement: S1 Images Folder — Image names are the column headings for and pertain to data in S1, S2, S4 and S5 Datasets. (ZIP) [file pone.0134978.s009.zip › S1_imagesfolder/Dur1_D.png]

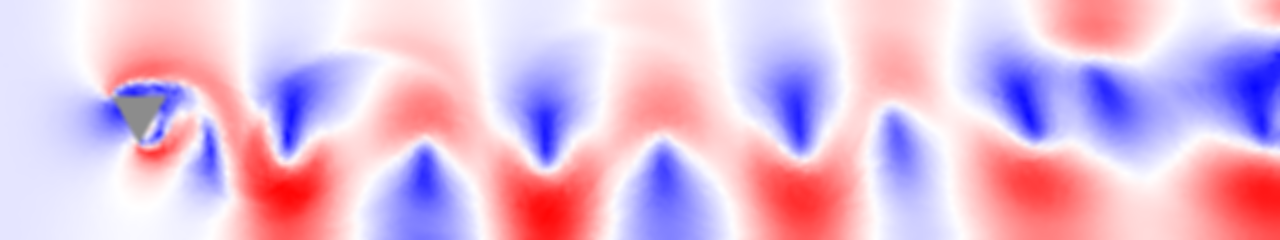

Supplement: S1 Images Folder — Image names are the column headings for and pertain to data in S1, S2, S4 and S5 Datasets. (ZIP) [file pone.0134978.s009.zip › S1_imagesfolder/Msh1_B.png]

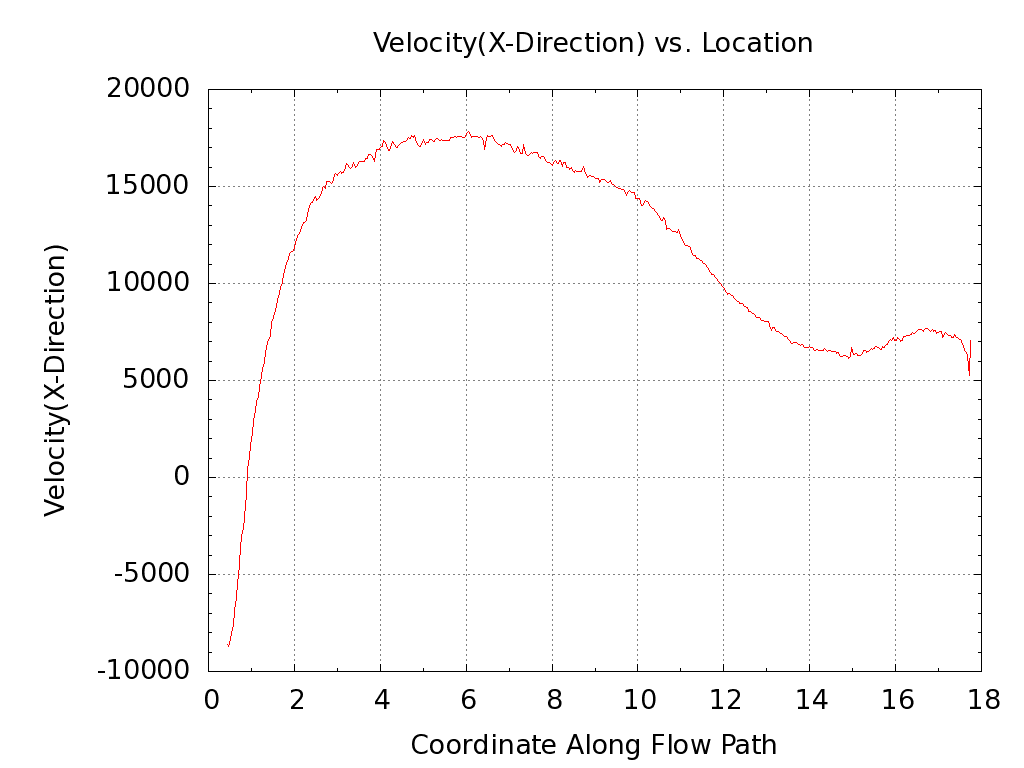

Supplement: S1 Images Folder — Image names are the column headings for and pertain to data in S1, S2, S4 and S5 Datasets. (ZIP) [file pone.0134978.s009.zip › S1_imagesfolder/Msh1_C.png]

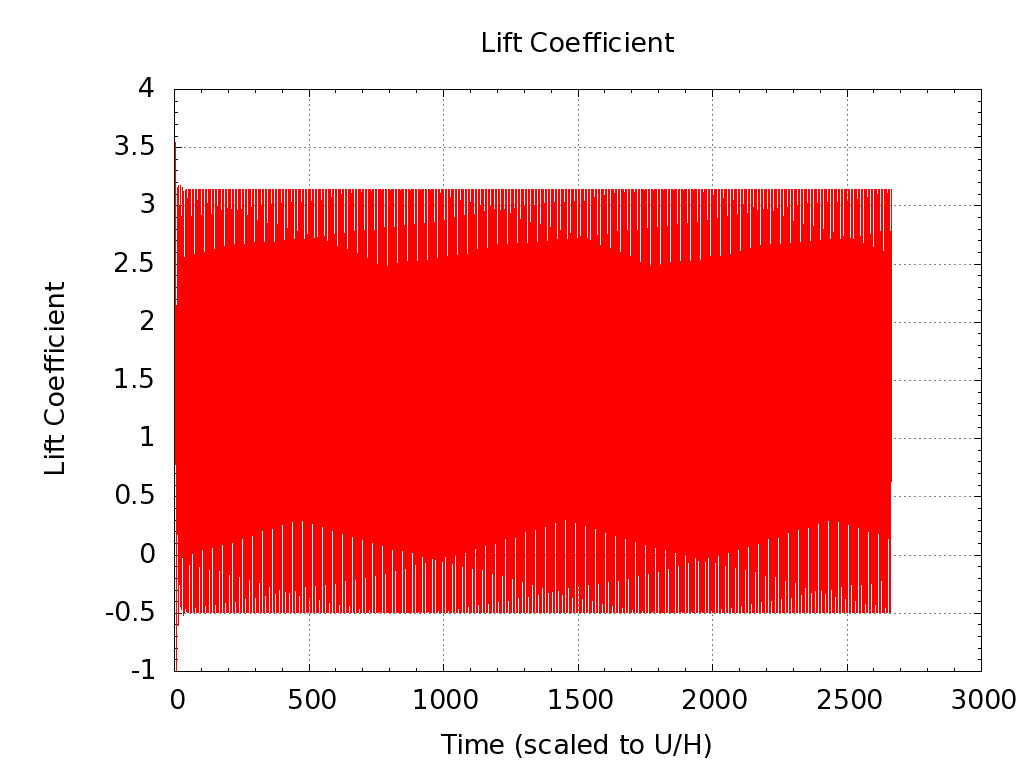

Supplement: S1 Images Folder — Image names are the column headings for and pertain to data in S1, S2, S4 and S5 Datasets. (ZIP) [file pone.0134978.s009.zip › S1_imagesfolder/Msh1_D.png]

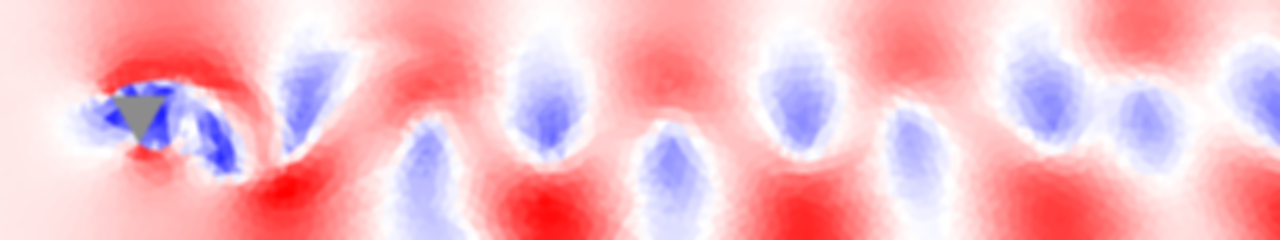

Supplement: S1 Images Folder — Image names are the column headings for and pertain to data in S1, S2, S4 and S5 Datasets. (ZIP) [file pone.0134978.s009.zip › S1_imagesfolder/Msh2_B.png]

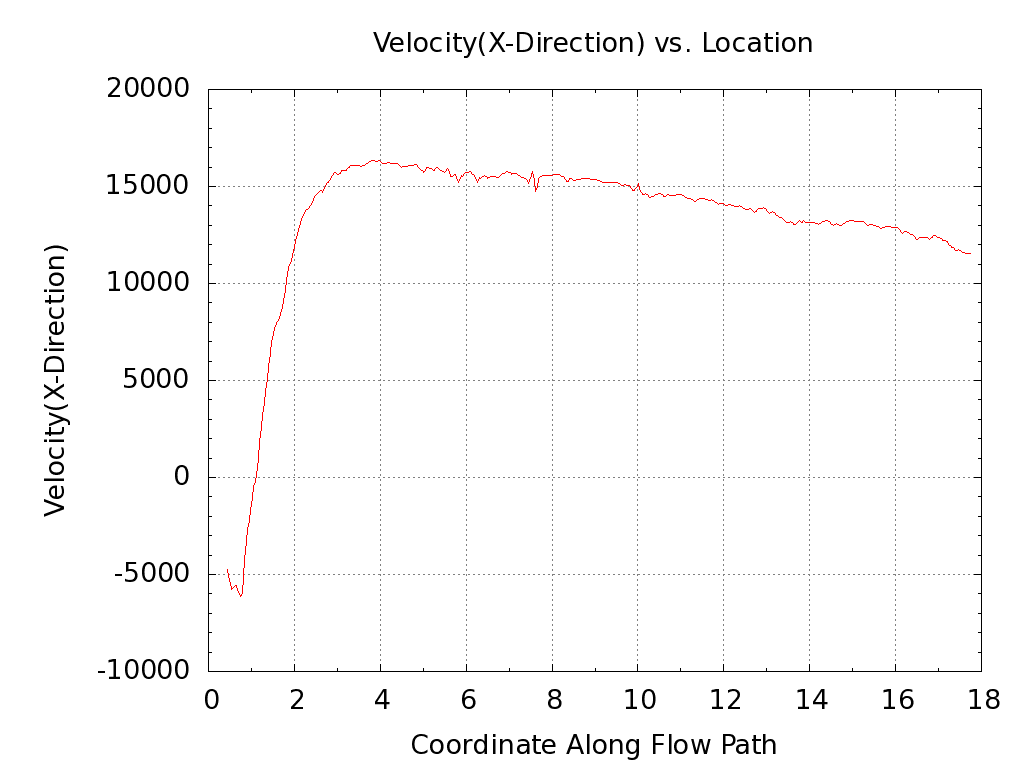

Supplement: S1 Images Folder — Image names are the column headings for and pertain to data in S1, S2, S4 and S5 Datasets. (ZIP) [file pone.0134978.s009.zip › S1_imagesfolder/Msh2_C.png]

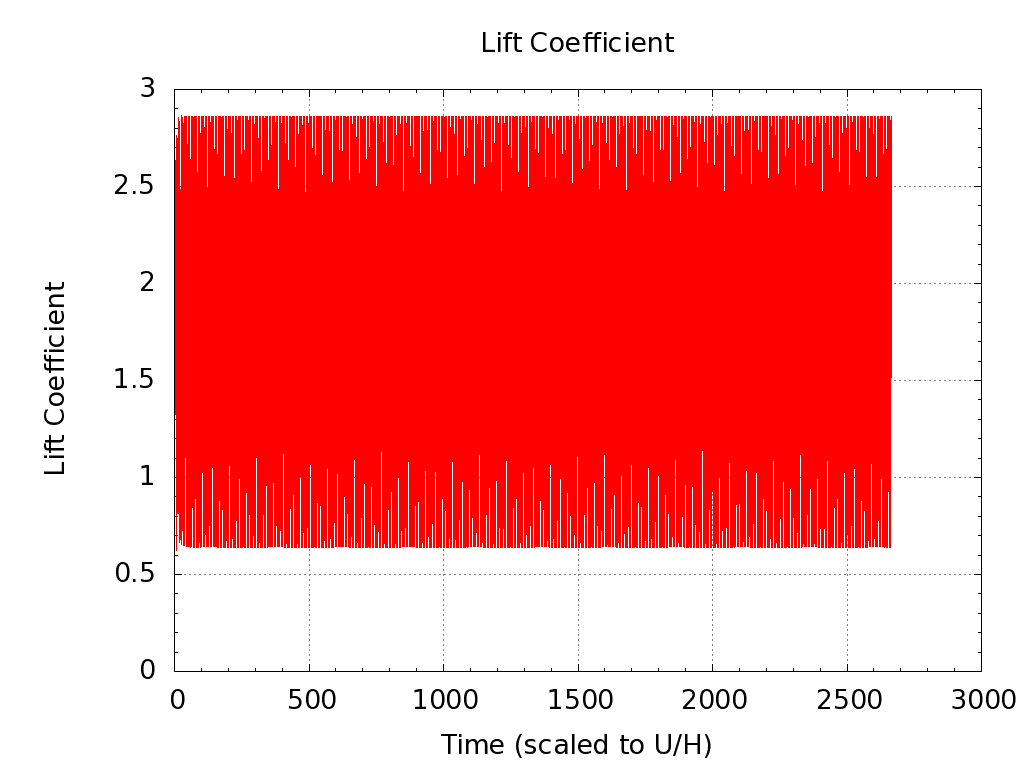

Supplement: S1 Images Folder — Image names are the column headings for and pertain to data in S1, S2, S4 and S5 Datasets. (ZIP) [file pone.0134978.s009.zip › S1_imagesfolder/Msh2_D.png]

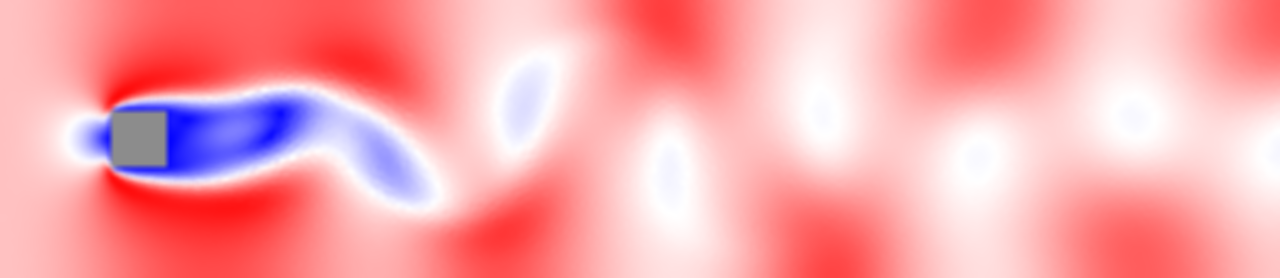

Supplement: S1 Images Folder — Image names are the column headings for and pertain to data in S1, S2, S4 and S5 Datasets. (ZIP) [file pone.0134978.s009.zip › S1_imagesfolder/Rot1_B.png]

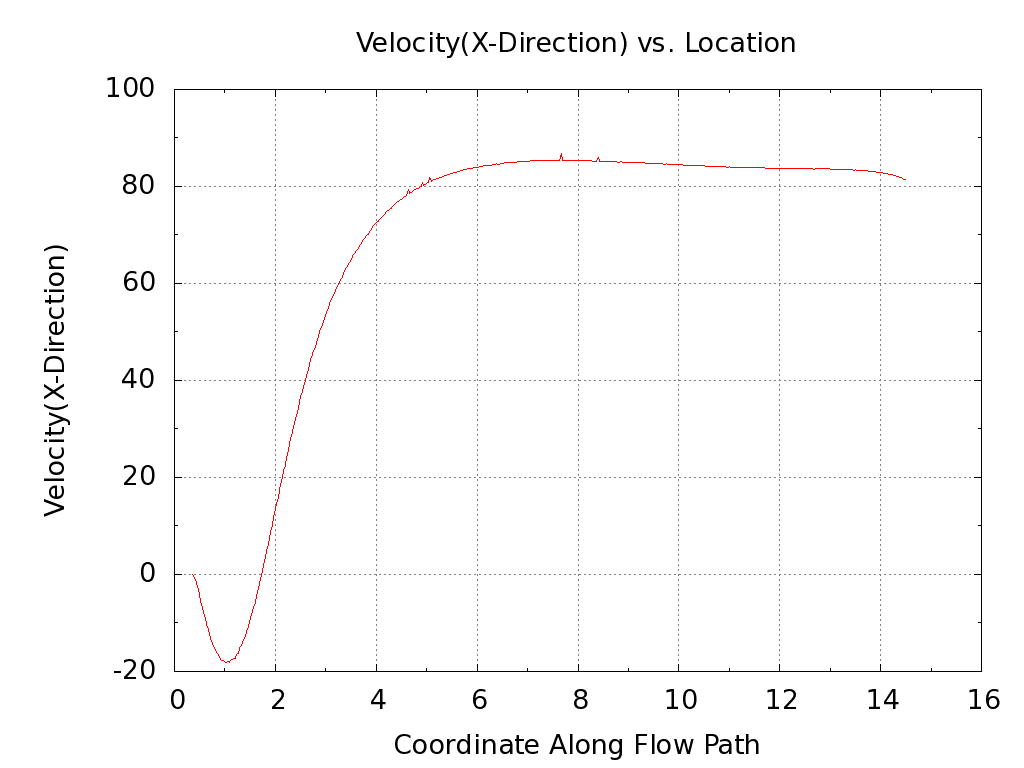

Supplement: S1 Images Folder — Image names are the column headings for and pertain to data in S1, S2, S4 and S5 Datasets. (ZIP) [file pone.0134978.s009.zip › S1_imagesfolder/Rot1_C.png]

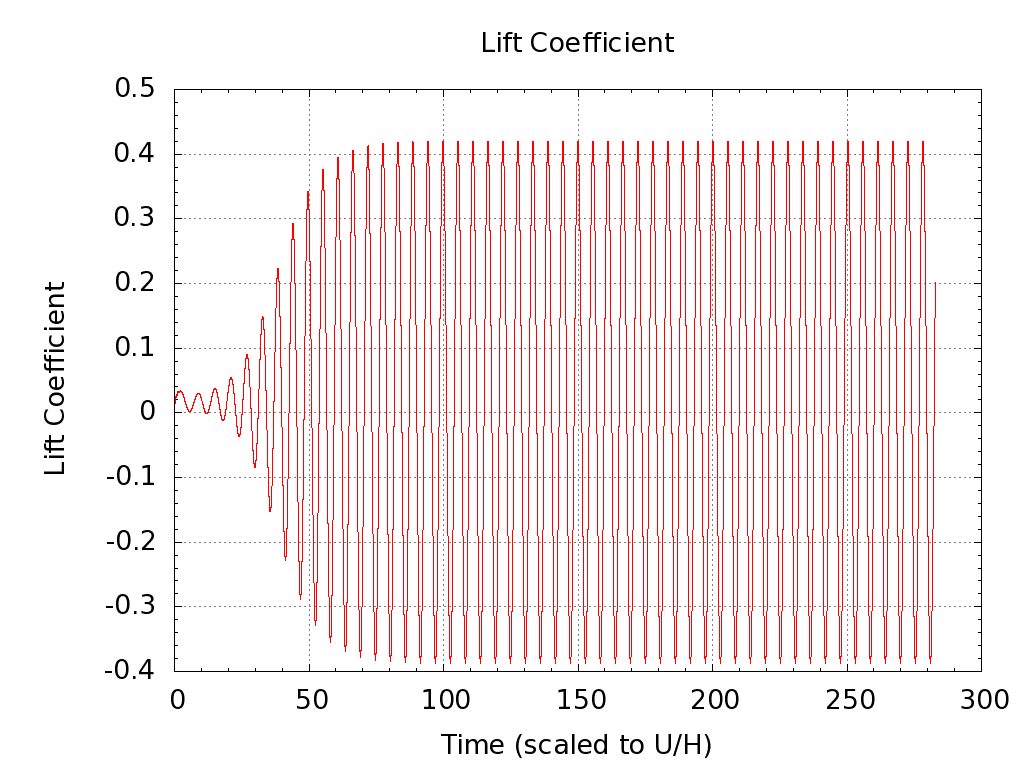

Supplement: S1 Images Folder — Image names are the column headings for and pertain to data in S1, S2, S4 and S5 Datasets. (ZIP) [file pone.0134978.s009.zip › S1_imagesfolder/Rot1_D.png]

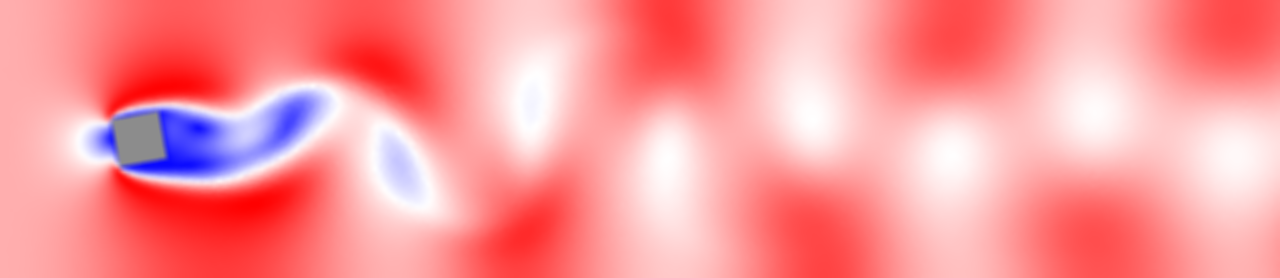

Supplement: S1 Images Folder — Image names are the column headings for and pertain to data in S1, S2, S4 and S5 Datasets. (ZIP) [file pone.0134978.s009.zip › S1_imagesfolder/Rot2_B.png]

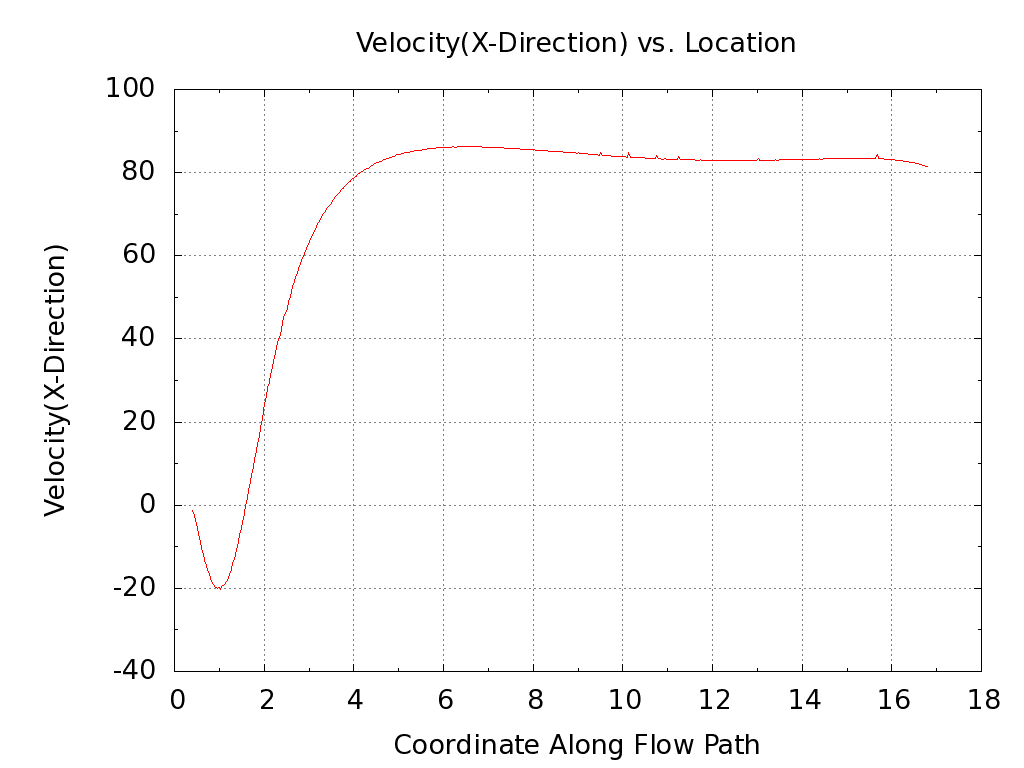

Supplement: S1 Images Folder — Image names are the column headings for and pertain to data in S1, S2, S4 and S5 Datasets. (ZIP) [file pone.0134978.s009.zip › S1_imagesfolder/Rot2_C.png]

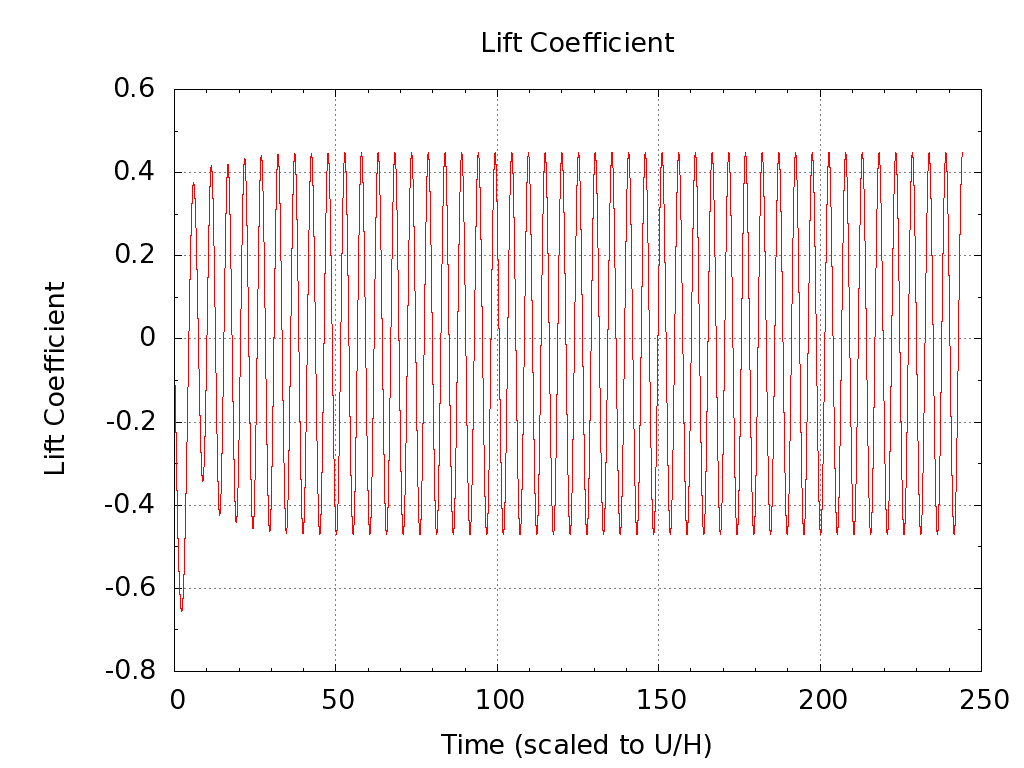

Supplement: S1 Images Folder — Image names are the column headings for and pertain to data in S1, S2, S4 and S5 Datasets. (ZIP) [file pone.0134978.s009.zip › S1_imagesfolder/Rot2_D.png]

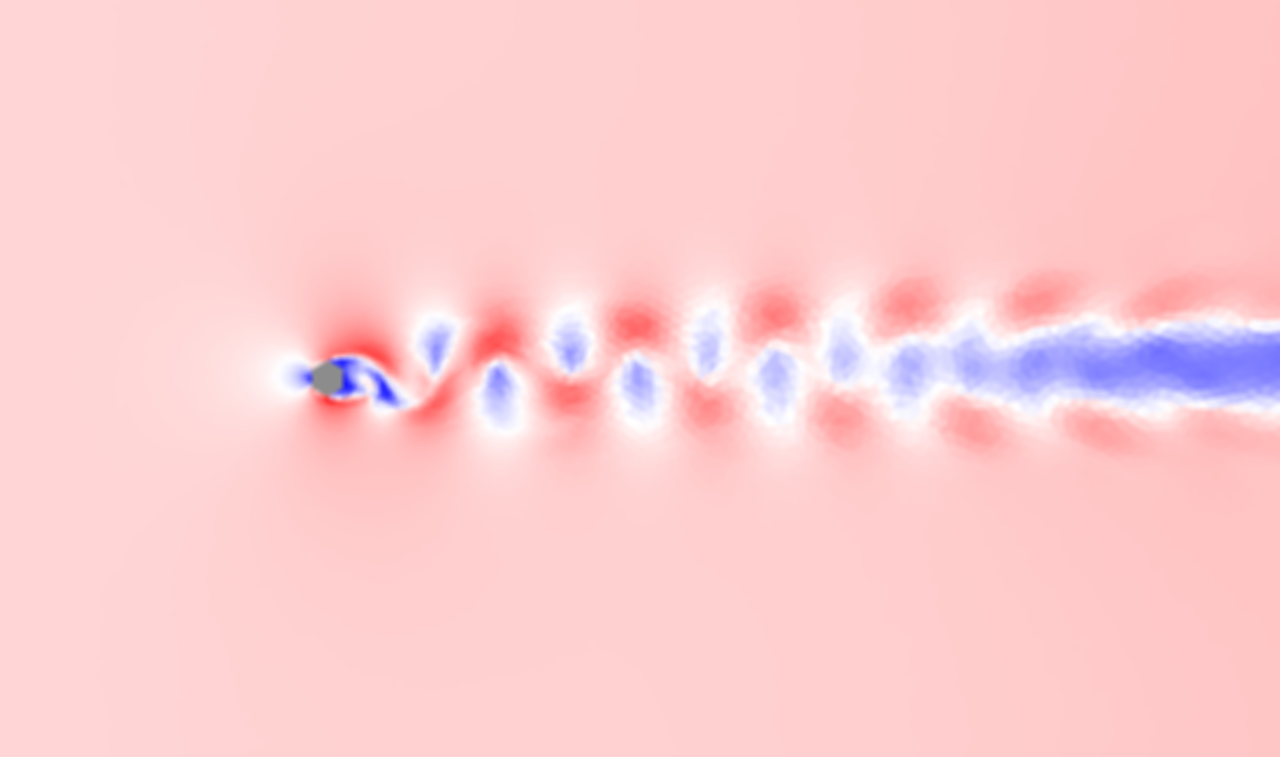

Supplement: S1 Images Folder — Image names are the column headings for and pertain to data in S1, S2, S4 and S5 Datasets. (ZIP) [file pone.0134978.s009.zip › S1_imagesfolder/Turb1_B.png]

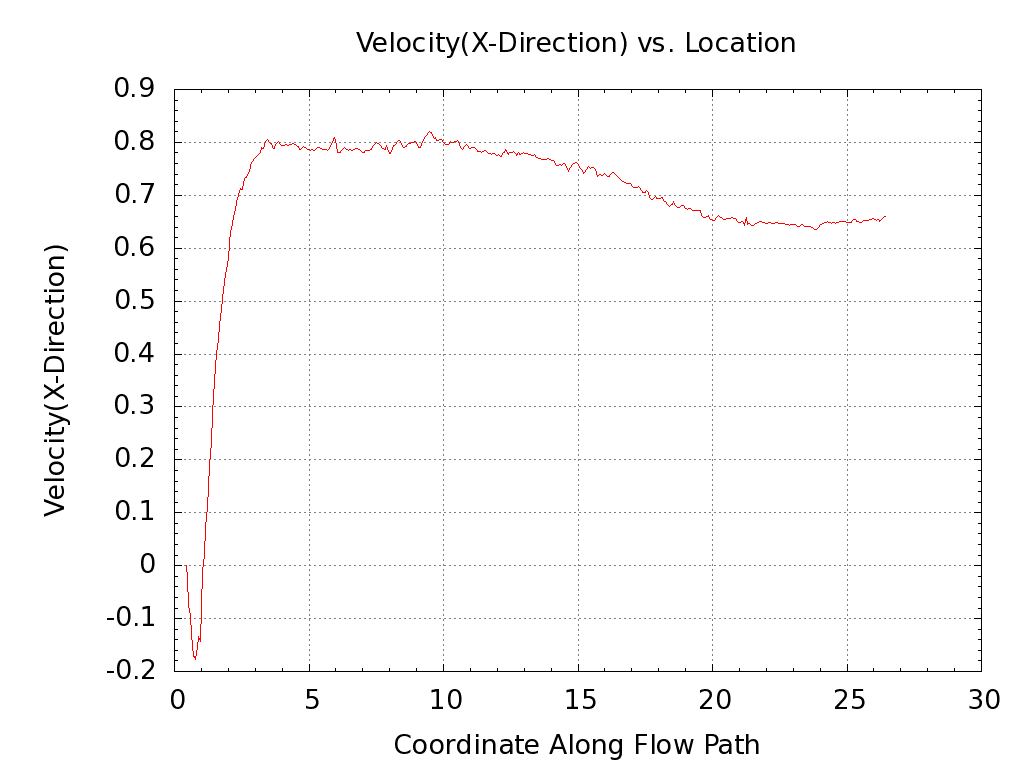

Supplement: S1 Images Folder — Image names are the column headings for and pertain to data in S1, S2, S4 and S5 Datasets. (ZIP) [file pone.0134978.s009.zip › S1_imagesfolder/Turb1_C.png]

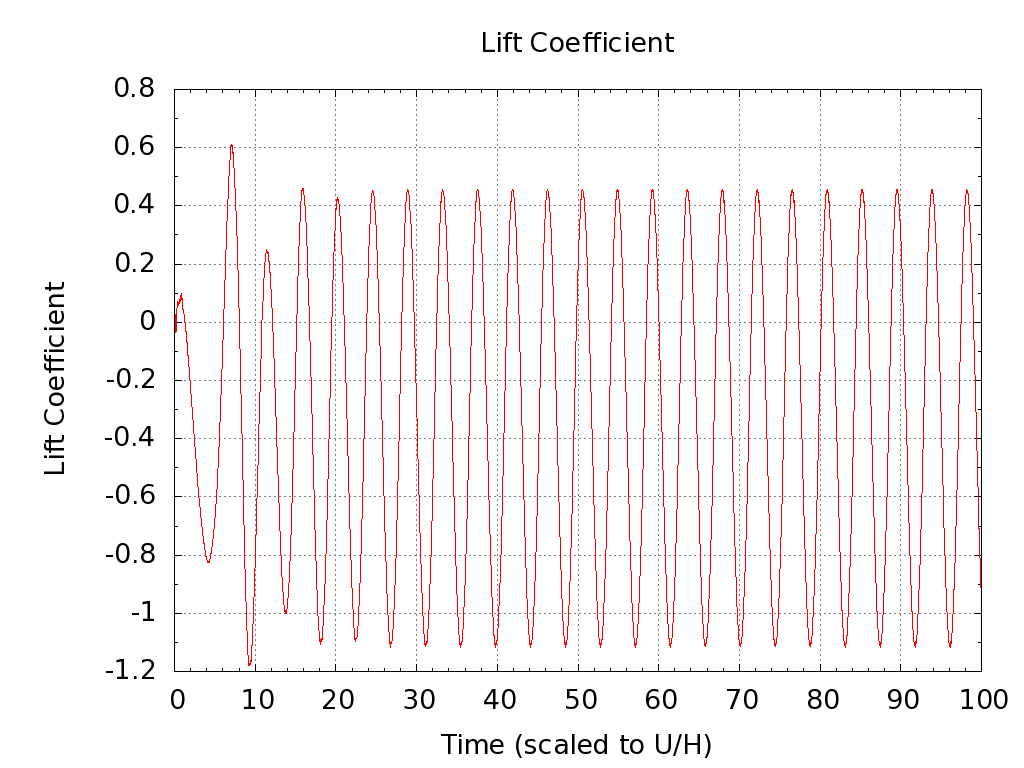

Supplement: S1 Images Folder — Image names are the column headings for and pertain to data in S1, S2, S4 and S5 Datasets. (ZIP) [file pone.0134978.s009.zip › S1_imagesfolder/Turb1_D.png]

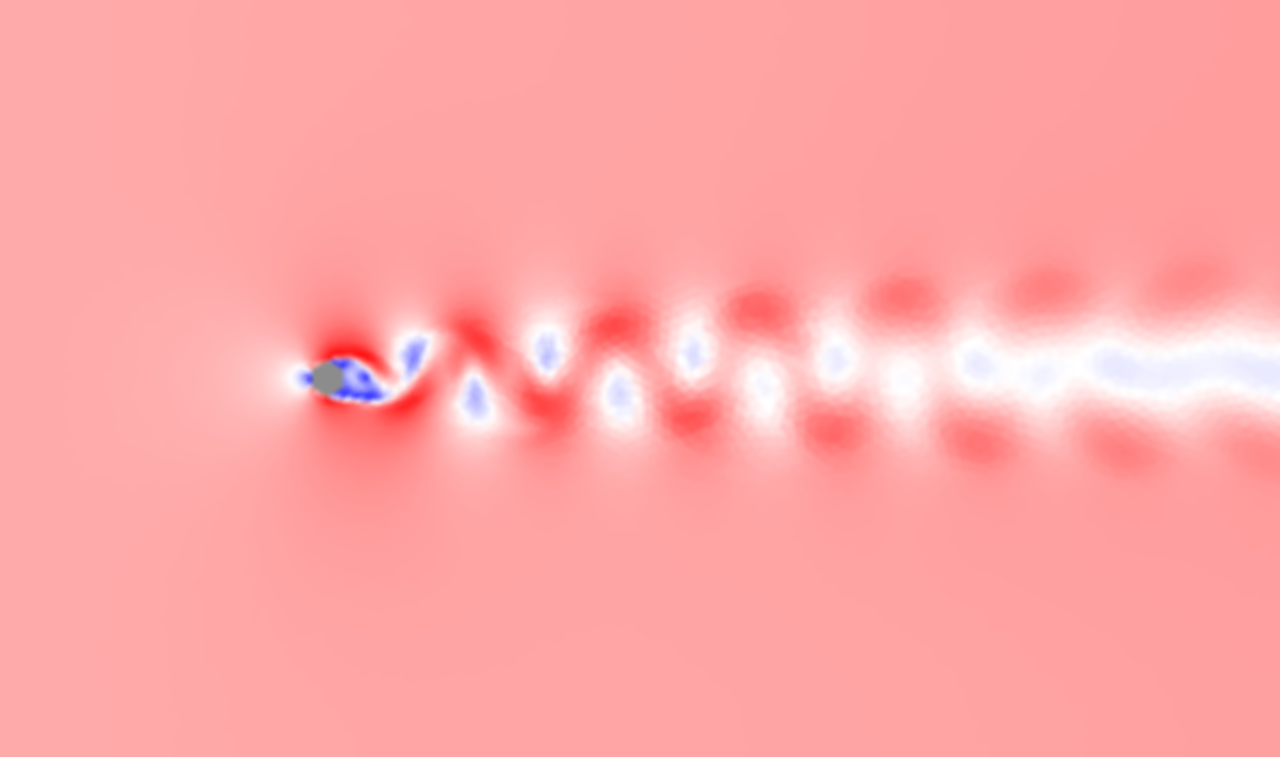

Supplement: S1 Images Folder — Image names are the column headings for and pertain to data in S1, S2, S4 and S5 Datasets. (ZIP) [file pone.0134978.s009.zip › S1_imagesfolder/Turb2_B.png]

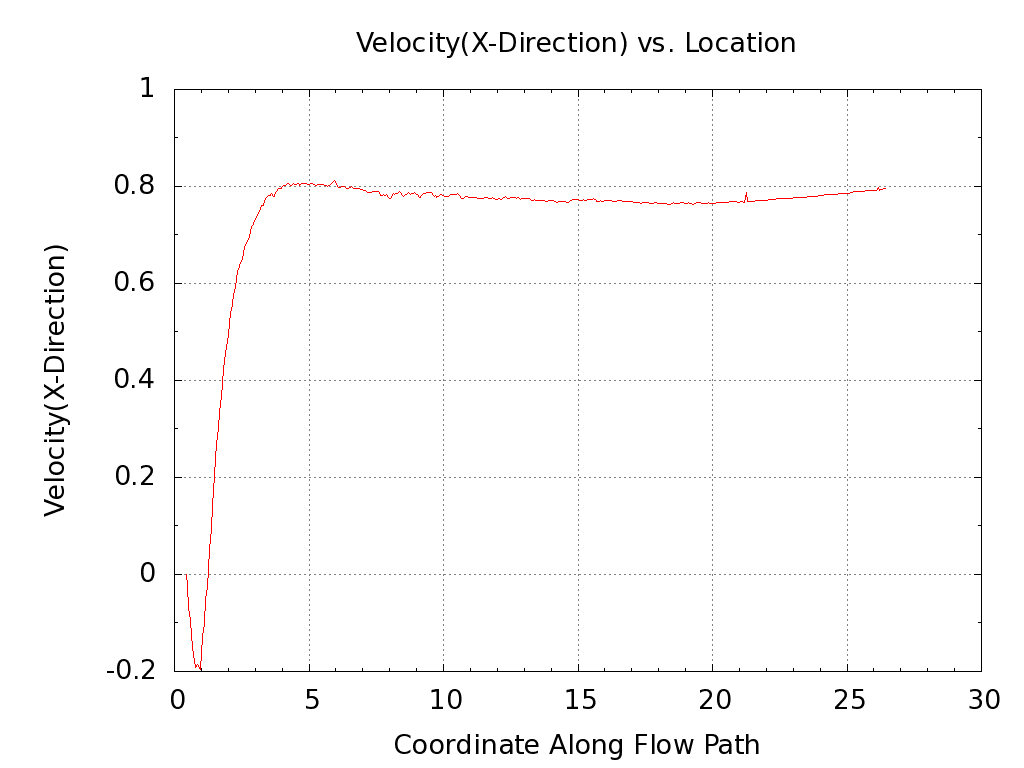

Supplement: S1 Images Folder — Image names are the column headings for and pertain to data in S1, S2, S4 and S5 Datasets. (ZIP) [file pone.0134978.s009.zip › S1_imagesfolder/Turb2_C.png]

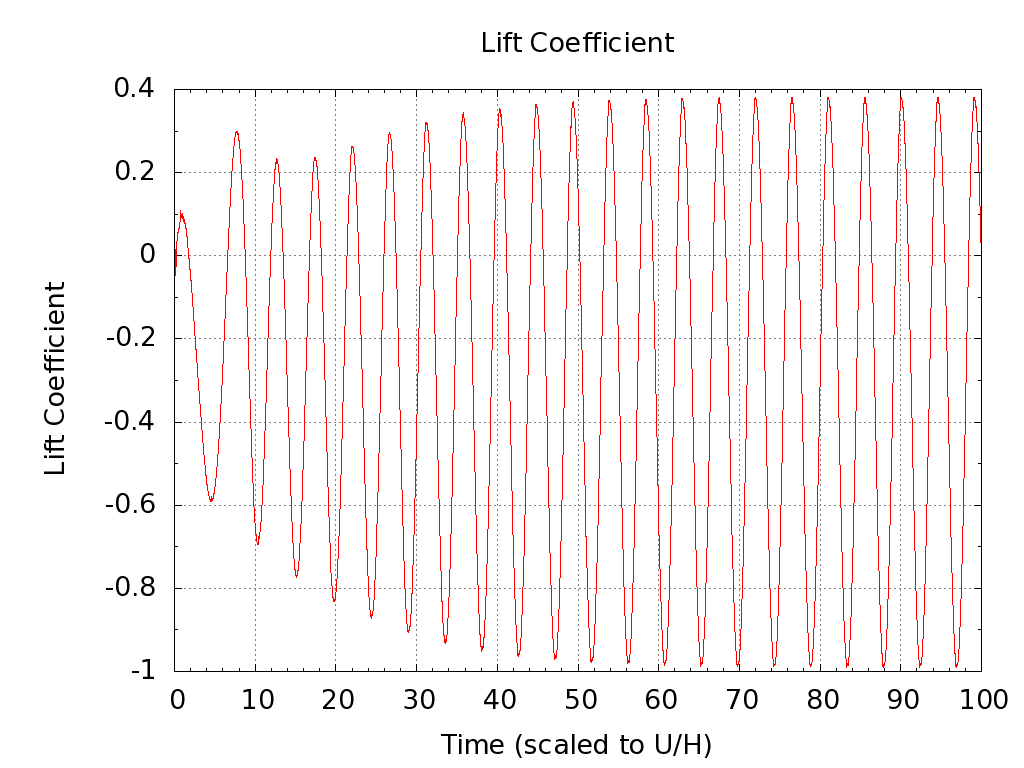

Supplement: S1 Images Folder — Image names are the column headings for and pertain to data in S1, S2, S4 and S5 Datasets. (ZIP) [file pone.0134978.s009.zip › S1_imagesfolder/Turb2_D.png]

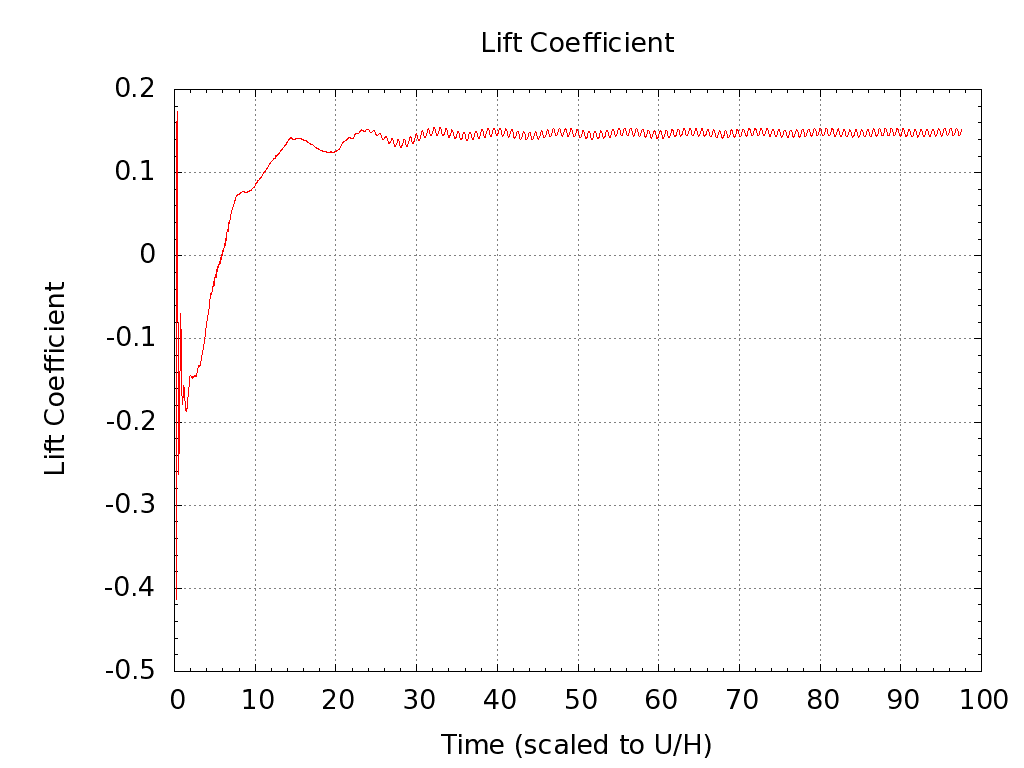

Supplement: S2 Images Folder — The numbers for each folder corresponds to the user number in phase2reports.txt. The Virtual Wind Tunnel does not generate images unless a user asks for a particular image. On a few occasions, users did not even look at certain graphs, so those graphs were not generated. Such non-inspected graphs are not present here. (Note: the x-axis label in the wake stream velocity graph, in Phase 2, due to a typo, indicated a scaling factor that was not actually applied.) (ZIP) [file pone.0134978.s010.zip › S2_imagesfolder/10/forceCoeffs.Cl.png]

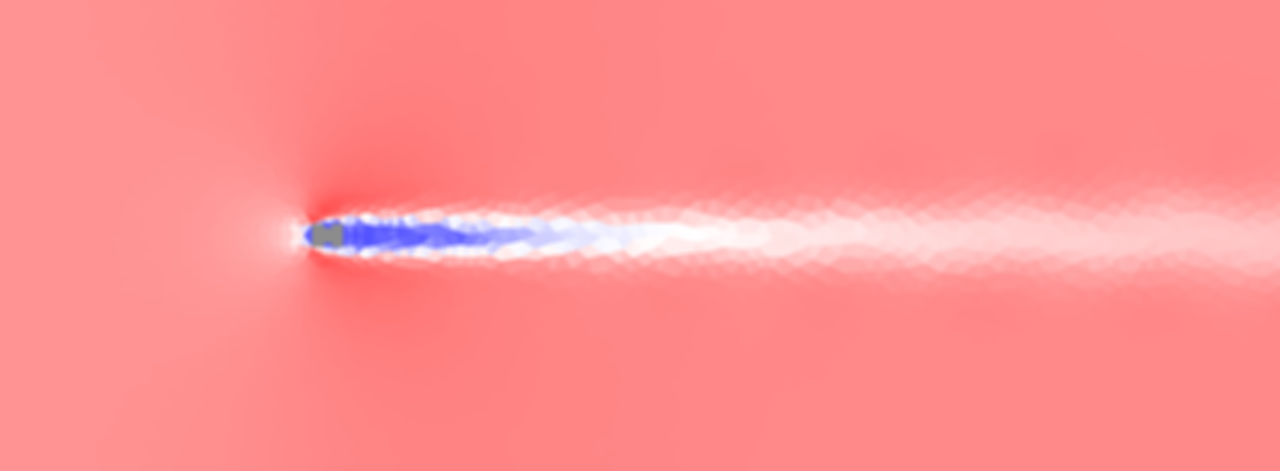

Supplement: S2 Images Folder — The numbers for each folder corresponds to the user number in phase2reports.txt. The Virtual Wind Tunnel does not generate images unless a user asks for a particular image. On a few occasions, users did not even look at certain graphs, so those graphs were not generated. Such non-inspected graphs are not present here. (Note: the x-axis label in the wake stream velocity graph, in Phase 2, due to a typo, indicated a scaling factor that was not actually applied.) (ZIP) [file pone.0134978.s010.zip › S2_imagesfolder/10/U.png]

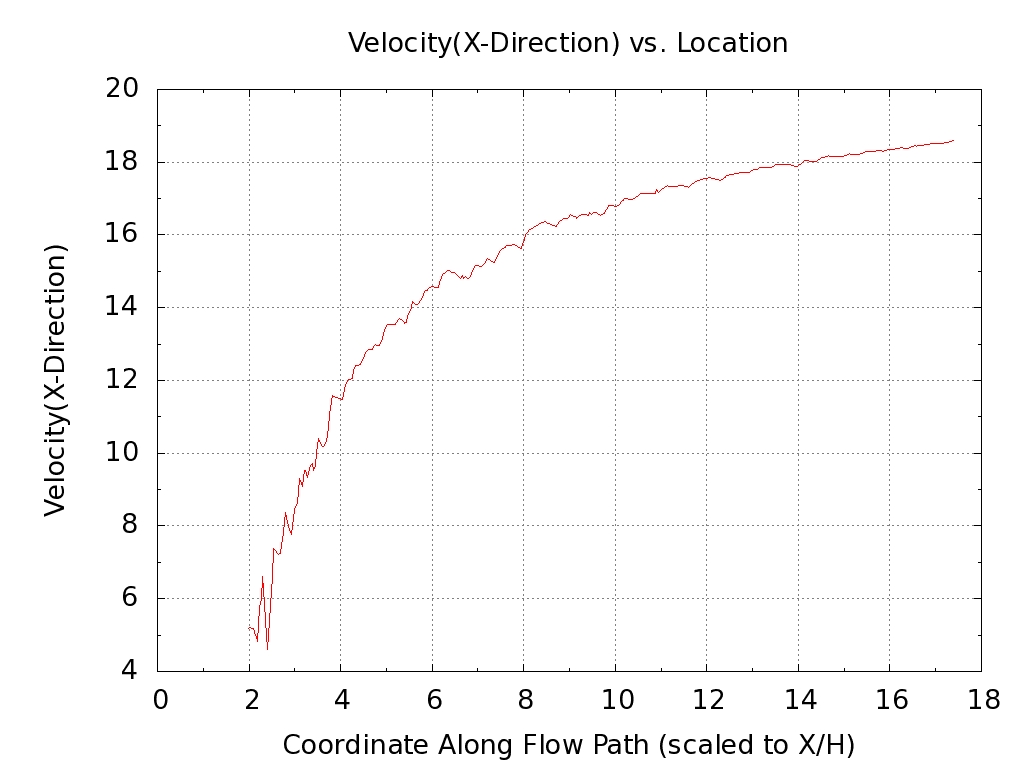

Supplement: S2 Images Folder — The numbers for each folder corresponds to the user number in phase2reports.txt. The Virtual Wind Tunnel does not generate images unless a user asks for a particular image. On a few occasions, users did not even look at certain graphs, so those graphs were not generated. Such non-inspected graphs are not present here. (Note: the x-axis label in the wake stream velocity graph, in Phase 2, due to a typo, indicated a scaling factor that was not actually applied.) (ZIP) [file pone.0134978.s010.zip › S2_imagesfolder/10/wakeCenter.UMean.X.png]

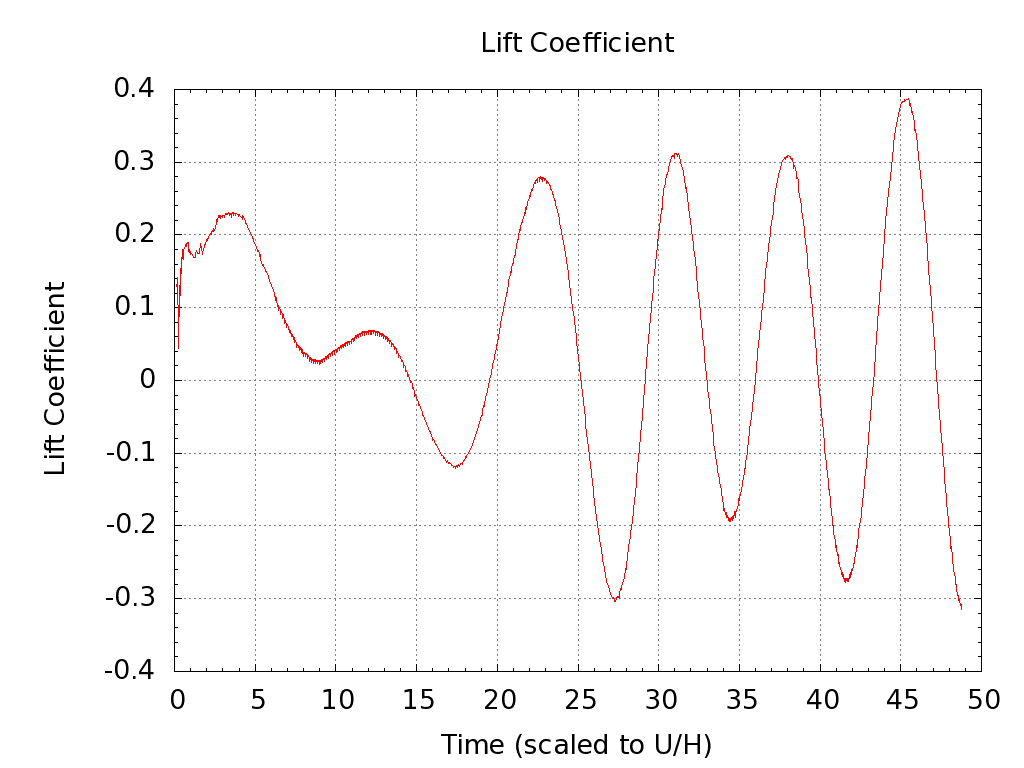

Supplement: S2 Images Folder — The numbers for each folder corresponds to the user number in phase2reports.txt. The Virtual Wind Tunnel does not generate images unless a user asks for a particular image. On a few occasions, users did not even look at certain graphs, so those graphs were not generated. Such non-inspected graphs are not present here. (Note: the x-axis label in the wake stream velocity graph, in Phase 2, due to a typo, indicated a scaling factor that was not actually applied.) (ZIP) [file pone.0134978.s010.zip › S2_imagesfolder/12/forceCoeffs.Cl.png]

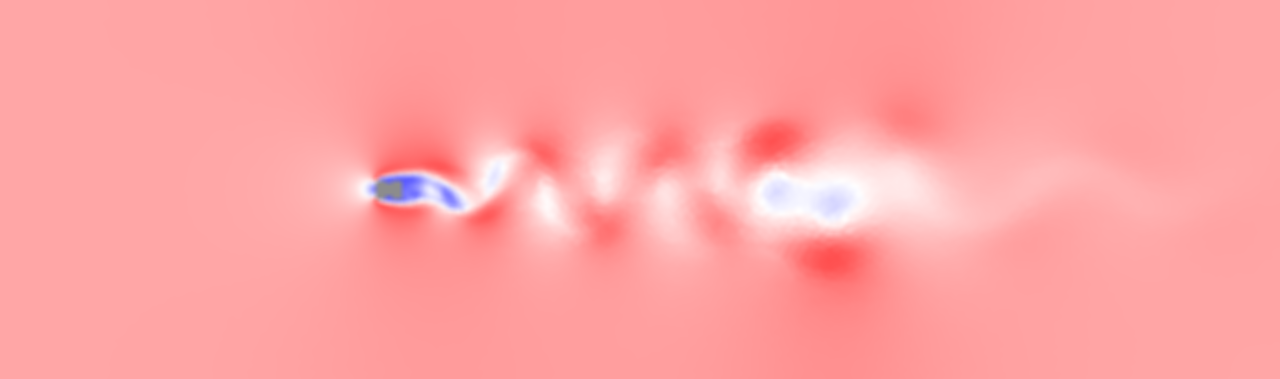

Supplement: S2 Images Folder — The numbers for each folder corresponds to the user number in phase2reports.txt. The Virtual Wind Tunnel does not generate images unless a user asks for a particular image. On a few occasions, users did not even look at certain graphs, so those graphs were not generated. Such non-inspected graphs are not present here. (Note: the x-axis label in the wake stream velocity graph, in Phase 2, due to a typo, indicated a scaling factor that was not actually applied.) (ZIP) [file pone.0134978.s010.zip › S2_imagesfolder/12/U.png]

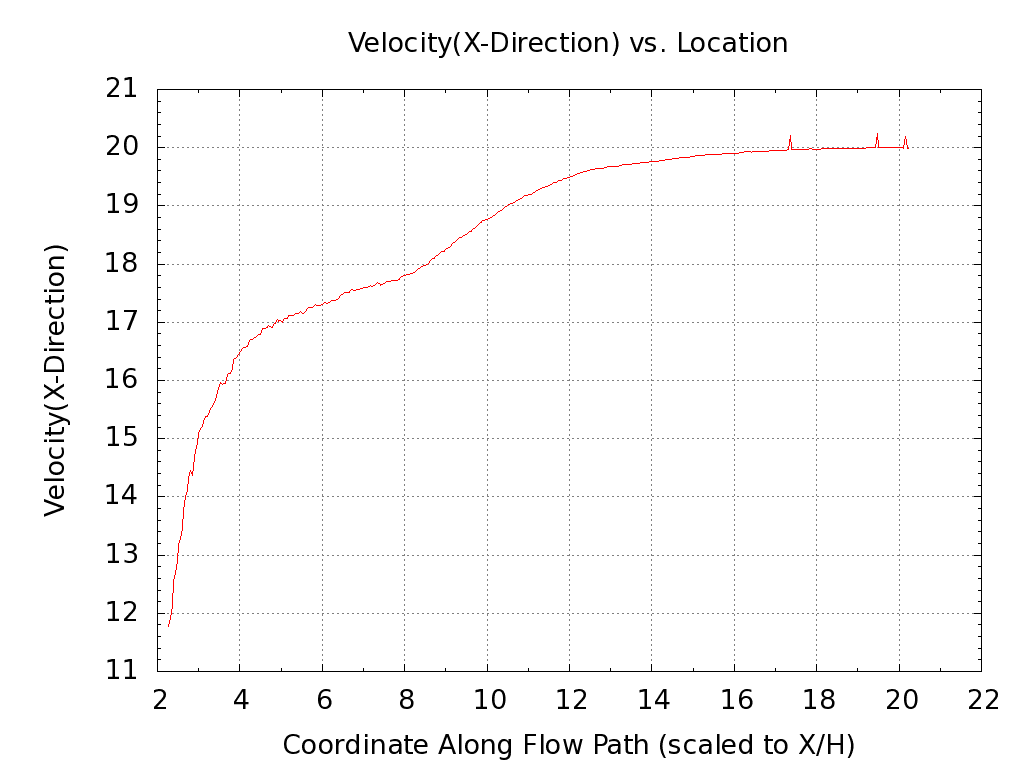

Supplement: S2 Images Folder — The numbers for each folder corresponds to the user number in phase2reports.txt. The Virtual Wind Tunnel does not generate images unless a user asks for a particular image. On a few occasions, users did not even look at certain graphs, so those graphs were not generated. Such non-inspected graphs are not present here. (Note: the x-axis label in the wake stream velocity graph, in Phase 2, due to a typo, indicated a scaling factor that was not actually applied.) (ZIP) [file pone.0134978.s010.zip › S2_imagesfolder/12/wakeCenter.UMean.X.png]

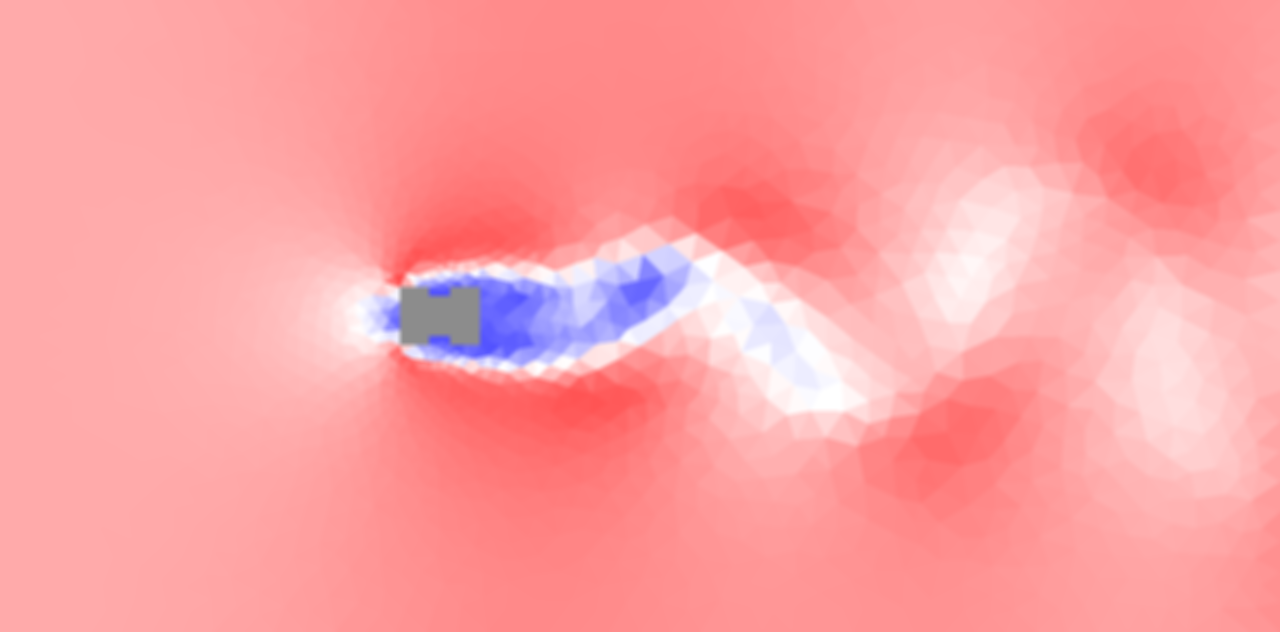

Supplement: S2 Images Folder — The numbers for each folder corresponds to the user number in phase2reports.txt. The Virtual Wind Tunnel does not generate images unless a user asks for a particular image. On a few occasions, users did not even look at certain graphs, so those graphs were not generated. Such non-inspected graphs are not present here. (Note: the x-axis label in the wake stream velocity graph, in Phase 2, due to a typo, indicated a scaling factor that was not actually applied.) (ZIP) [file pone.0134978.s010.zip › S2_imagesfolder/14/U.png]

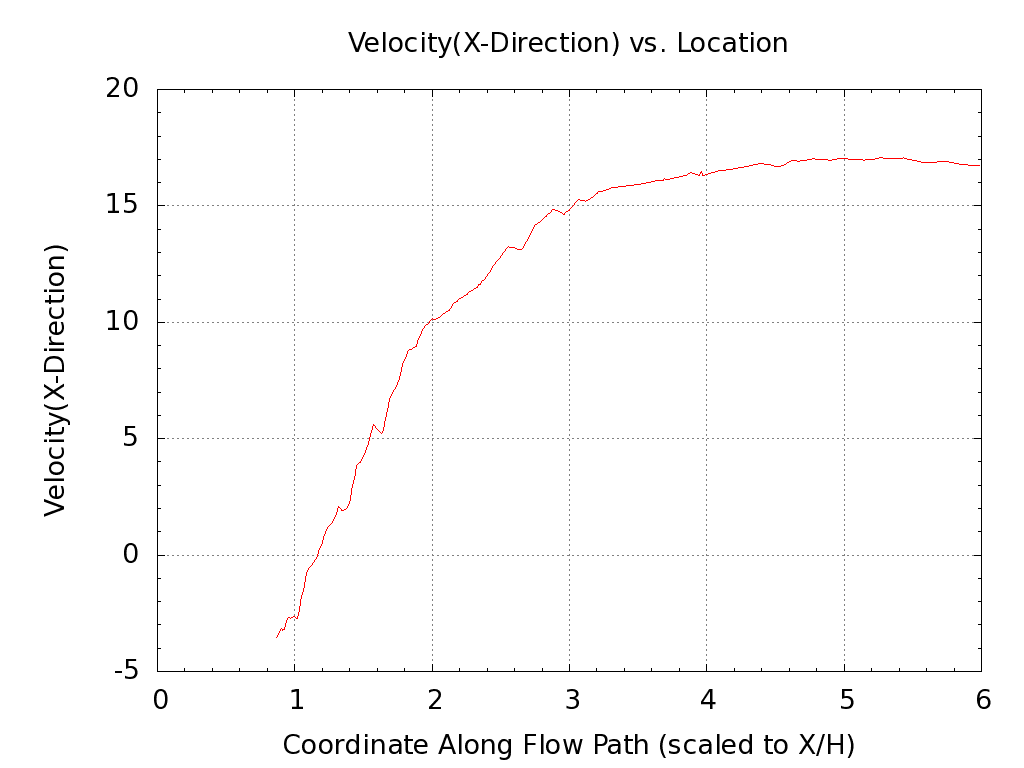

Supplement: S2 Images Folder — The numbers for each folder corresponds to the user number in phase2reports.txt. The Virtual Wind Tunnel does not generate images unless a user asks for a particular image. On a few occasions, users did not even look at certain graphs, so those graphs were not generated. Such non-inspected graphs are not present here. (Note: the x-axis label in the wake stream velocity graph, in Phase 2, due to a typo, indicated a scaling factor that was not actually applied.) (ZIP) [file pone.0134978.s010.zip › S2_imagesfolder/14/wakeCenter.UMean.X.png]

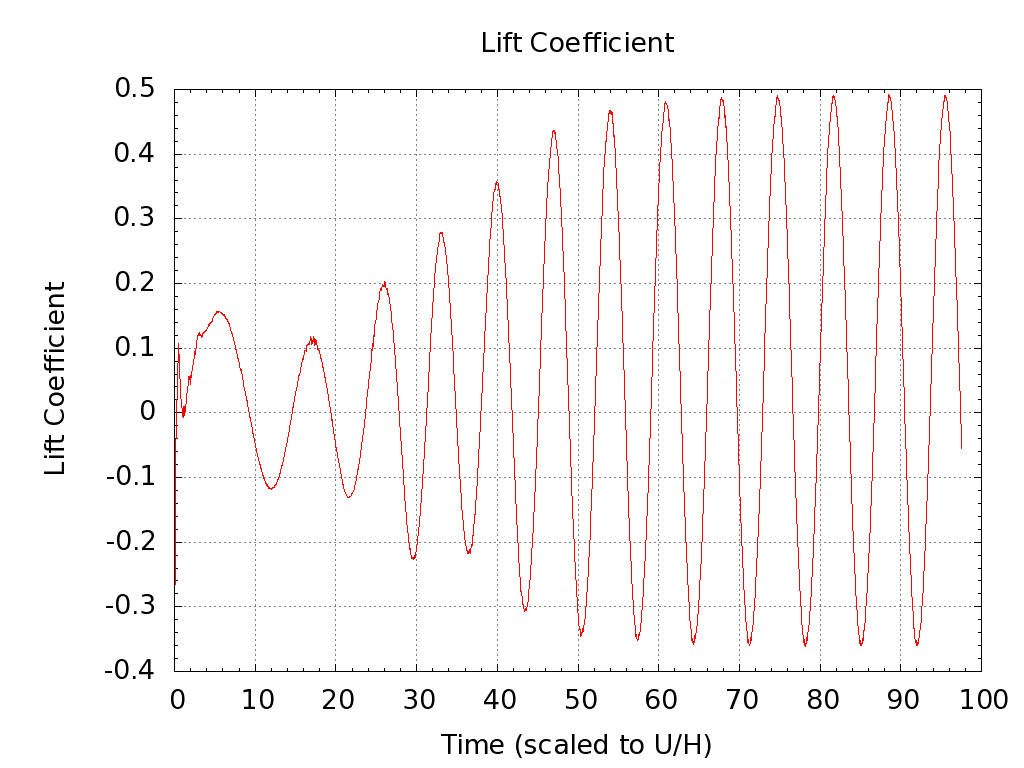

Supplement: S2 Images Folder — The numbers for each folder corresponds to the user number in phase2reports.txt. The Virtual Wind Tunnel does not generate images unless a user asks for a particular image. On a few occasions, users did not even look at certain graphs, so those graphs were not generated. Such non-inspected graphs are not present here. (Note: the x-axis label in the wake stream velocity graph, in Phase 2, due to a typo, indicated a scaling factor that was not actually applied.) (ZIP) [file pone.0134978.s010.zip › S2_imagesfolder/15/forceCoeffs.Cl.png]

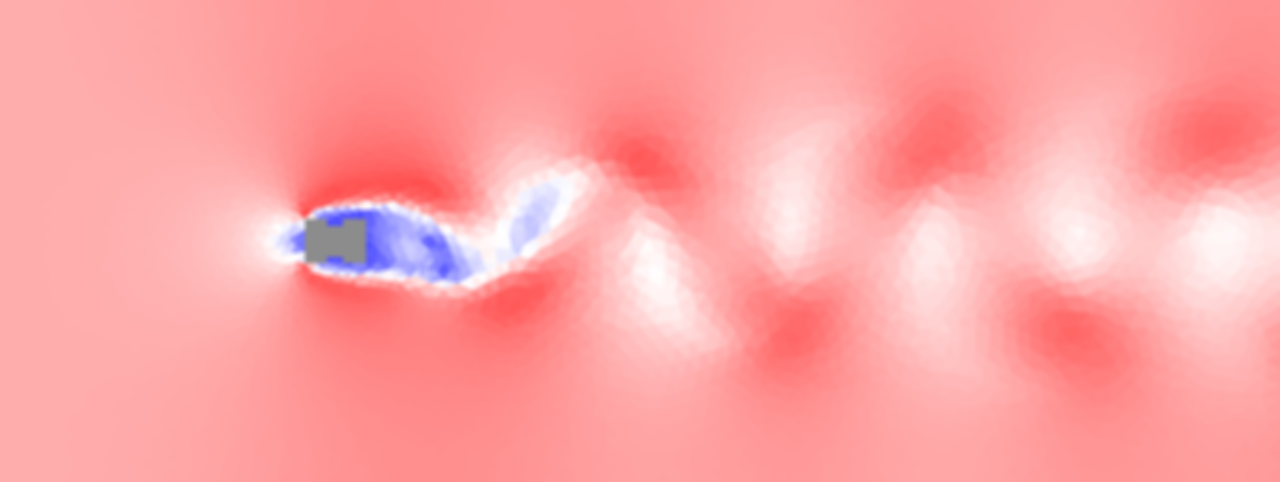

Supplement: S2 Images Folder — The numbers for each folder corresponds to the user number in phase2reports.txt. The Virtual Wind Tunnel does not generate images unless a user asks for a particular image. On a few occasions, users did not even look at certain graphs, so those graphs were not generated. Such non-inspected graphs are not present here. (Note: the x-axis label in the wake stream velocity graph, in Phase 2, due to a typo, indicated a scaling factor that was not actually applied.) (ZIP) [file pone.0134978.s010.zip › S2_imagesfolder/15/U.png]

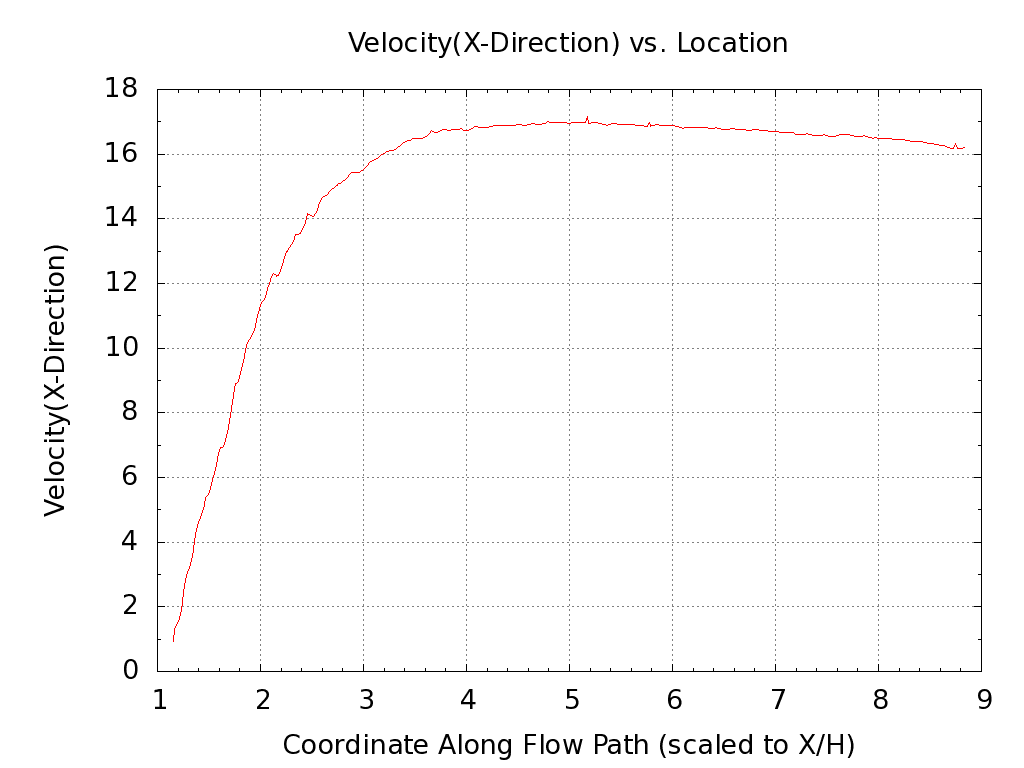

Supplement: S2 Images Folder — The numbers for each folder corresponds to the user number in phase2reports.txt. The Virtual Wind Tunnel does not generate images unless a user asks for a particular image. On a few occasions, users did not even look at certain graphs, so those graphs were not generated. Such non-inspected graphs are not present here. (Note: the x-axis label in the wake stream velocity graph, in Phase 2, due to a typo, indicated a scaling factor that was not actually applied.) (ZIP) [file pone.0134978.s010.zip › S2_imagesfolder/15/wakeCenter.UMean.X.png]

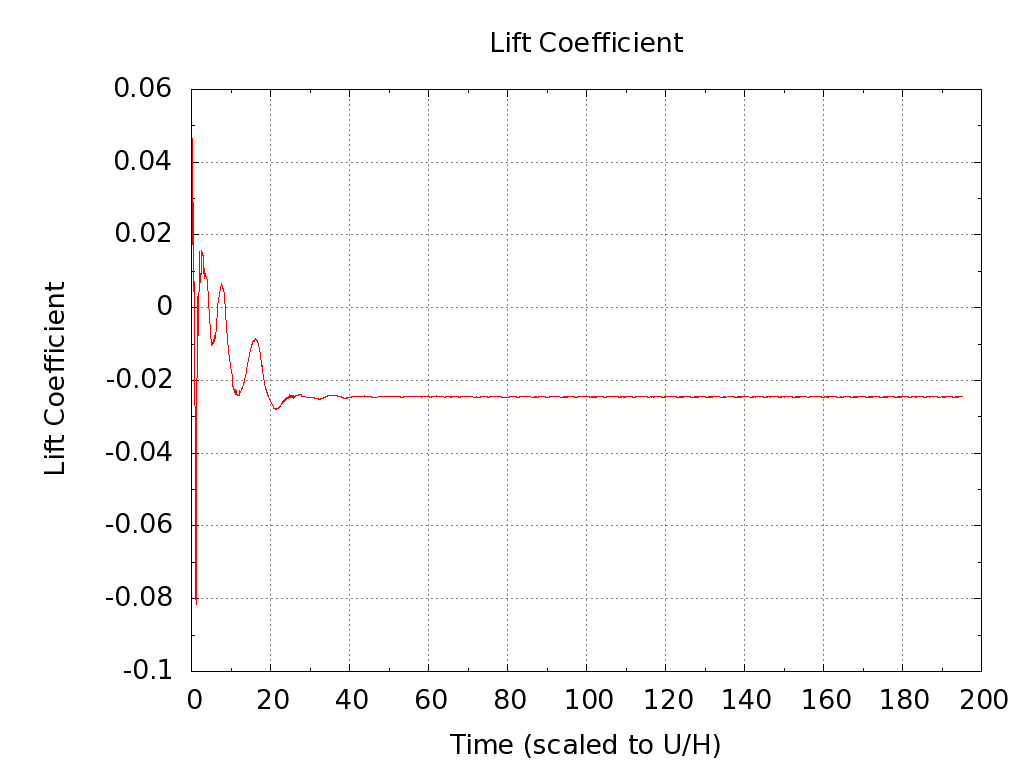

Supplement: S2 Images Folder — The numbers for each folder corresponds to the user number in phase2reports.txt. The Virtual Wind Tunnel does not generate images unless a user asks for a particular image. On a few occasions, users did not even look at certain graphs, so those graphs were not generated. Such non-inspected graphs are not present here. (Note: the x-axis label in the wake stream velocity graph, in Phase 2, due to a typo, indicated a scaling factor that was not actually applied.) (ZIP) [file pone.0134978.s010.zip › S2_imagesfolder/16/forceCoeffs.Cl.png]

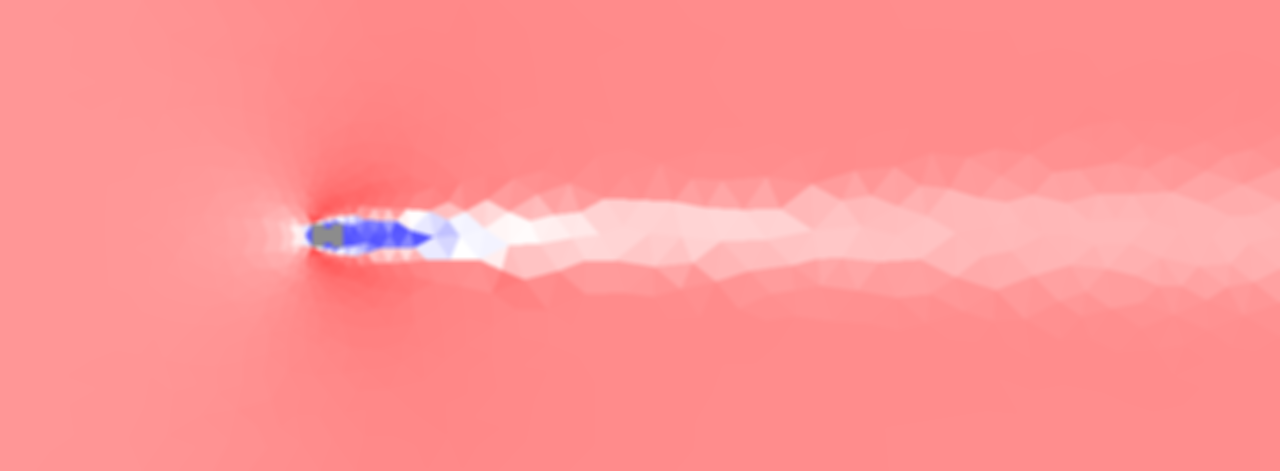

Supplement: S2 Images Folder — The numbers for each folder corresponds to the user number in phase2reports.txt. The Virtual Wind Tunnel does not generate images unless a user asks for a particular image. On a few occasions, users did not even look at certain graphs, so those graphs were not generated. Such non-inspected graphs are not present here. (Note: the x-axis label in the wake stream velocity graph, in Phase 2, due to a typo, indicated a scaling factor that was not actually applied.) (ZIP) [file pone.0134978.s010.zip › S2_imagesfolder/16/U.png]

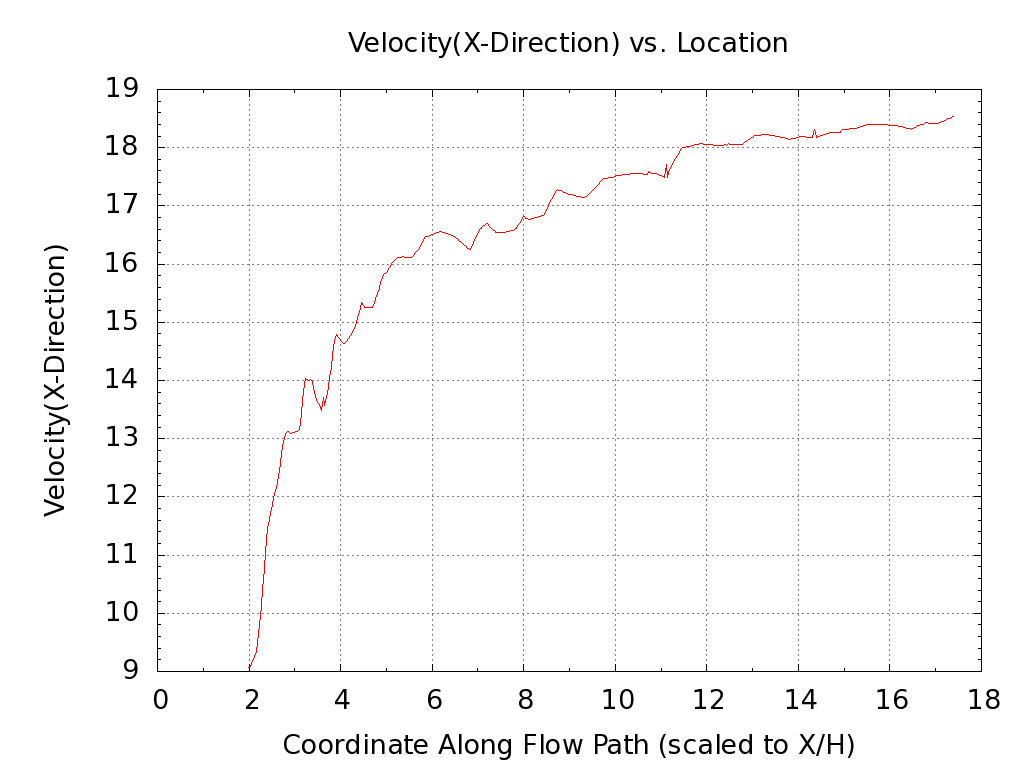

Supplement: S2 Images Folder — The numbers for each folder corresponds to the user number in phase2reports.txt. The Virtual Wind Tunnel does not generate images unless a user asks for a particular image. On a few occasions, users did not even look at certain graphs, so those graphs were not generated. Such non-inspected graphs are not present here. (Note: the x-axis label in the wake stream velocity graph, in Phase 2, due to a typo, indicated a scaling factor that was not actually applied.) (ZIP) [file pone.0134978.s010.zip › S2_imagesfolder/16/wakeCenter.UMean.X.png]

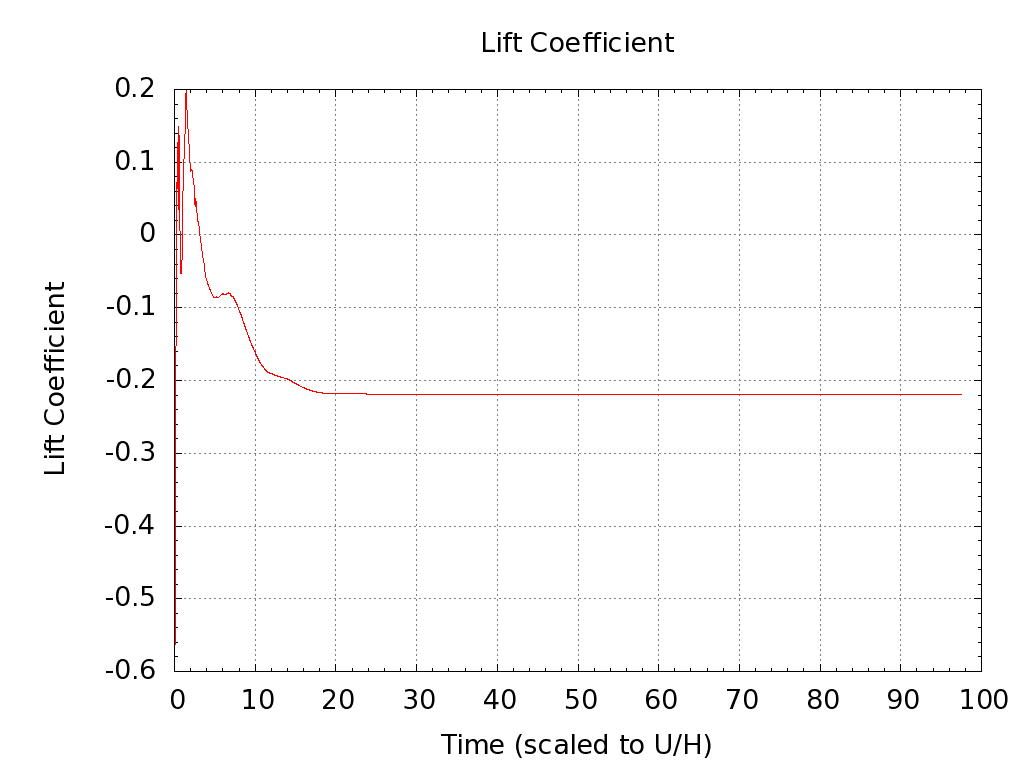

Supplement: S2 Images Folder — The numbers for each folder corresponds to the user number in phase2reports.txt. The Virtual Wind Tunnel does not generate images unless a user asks for a particular image. On a few occasions, users did not even look at certain graphs, so those graphs were not generated. Such non-inspected graphs are not present here. (Note: the x-axis label in the wake stream velocity graph, in Phase 2, due to a typo, indicated a scaling factor that was not actually applied.) (ZIP) [file pone.0134978.s010.zip › S2_imagesfolder/17/forceCoeffs.Cl.png]

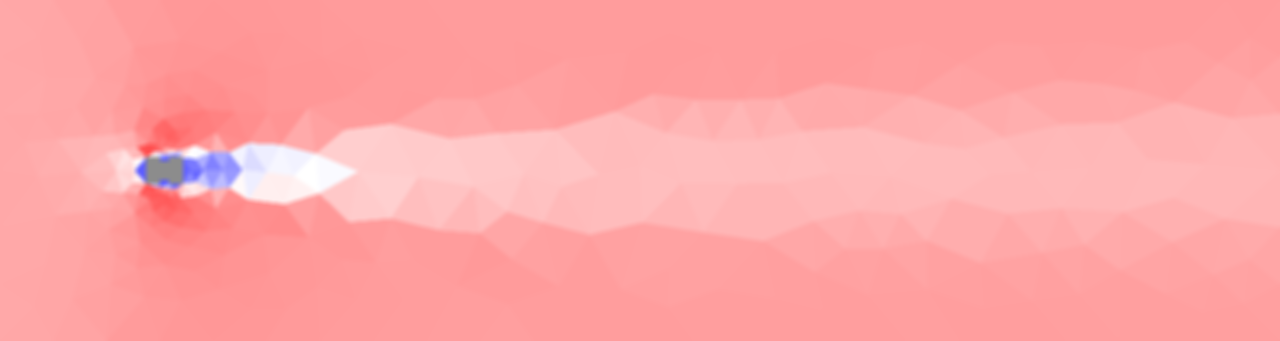

Supplement: S2 Images Folder — The numbers for each folder corresponds to the user number in phase2reports.txt. The Virtual Wind Tunnel does not generate images unless a user asks for a particular image. On a few occasions, users did not even look at certain graphs, so those graphs were not generated. Such non-inspected graphs are not present here. (Note: the x-axis label in the wake stream velocity graph, in Phase 2, due to a typo, indicated a scaling factor that was not actually applied.) (ZIP) [file pone.0134978.s010.zip › S2_imagesfolder/17/U.png]

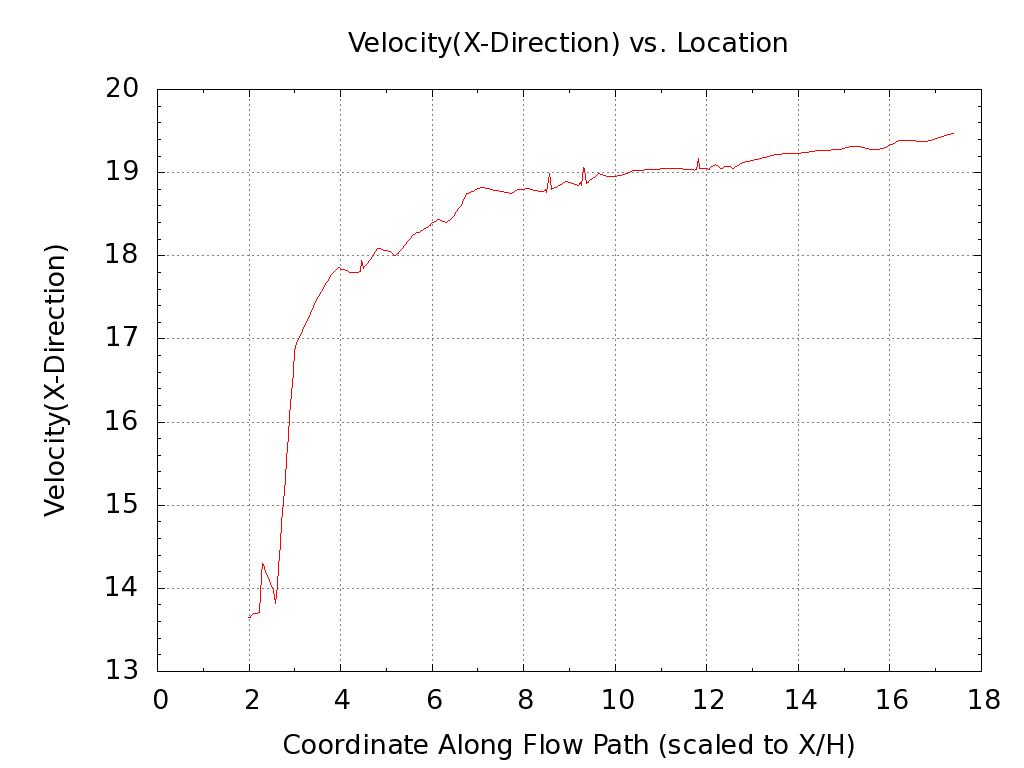

Supplement: S2 Images Folder — The numbers for each folder corresponds to the user number in phase2reports.txt. The Virtual Wind Tunnel does not generate images unless a user asks for a particular image. On a few occasions, users did not even look at certain graphs, so those graphs were not generated. Such non-inspected graphs are not present here. (Note: the x-axis label in the wake stream velocity graph, in Phase 2, due to a typo, indicated a scaling factor that was not actually applied.) (ZIP) [file pone.0134978.s010.zip › S2_imagesfolder/17/wakeCenter.UMean.X.png]

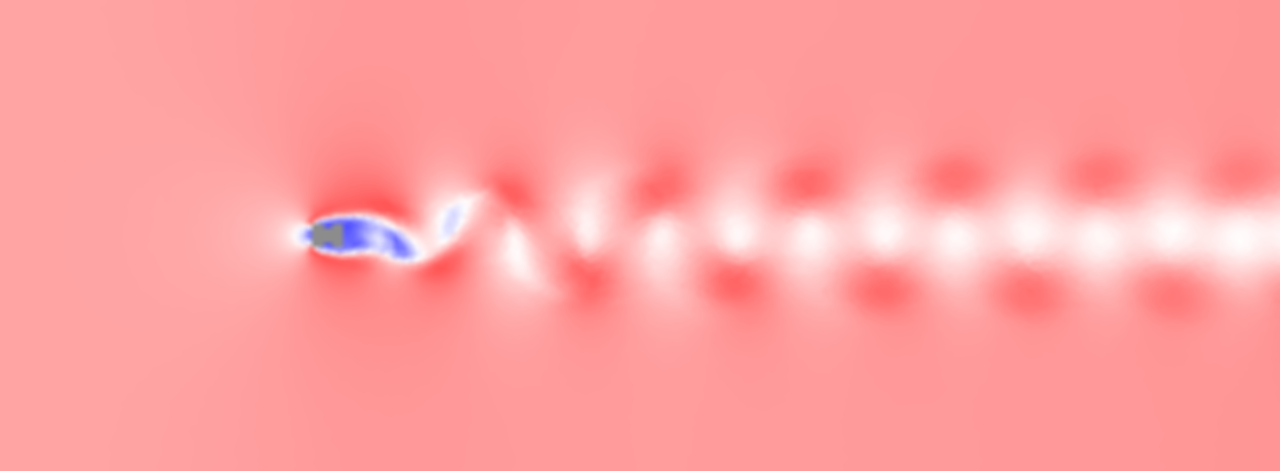

Supplement: S2 Images Folder — The numbers for each folder corresponds to the user number in phase2reports.txt. The Virtual Wind Tunnel does not generate images unless a user asks for a particular image. On a few occasions, users did not even look at certain graphs, so those graphs were not generated. Such non-inspected graphs are not present here. (Note: the x-axis label in the wake stream velocity graph, in Phase 2, due to a typo, indicated a scaling factor that was not actually applied.) (ZIP) [file pone.0134978.s010.zip › S2_imagesfolder/19/U.png]

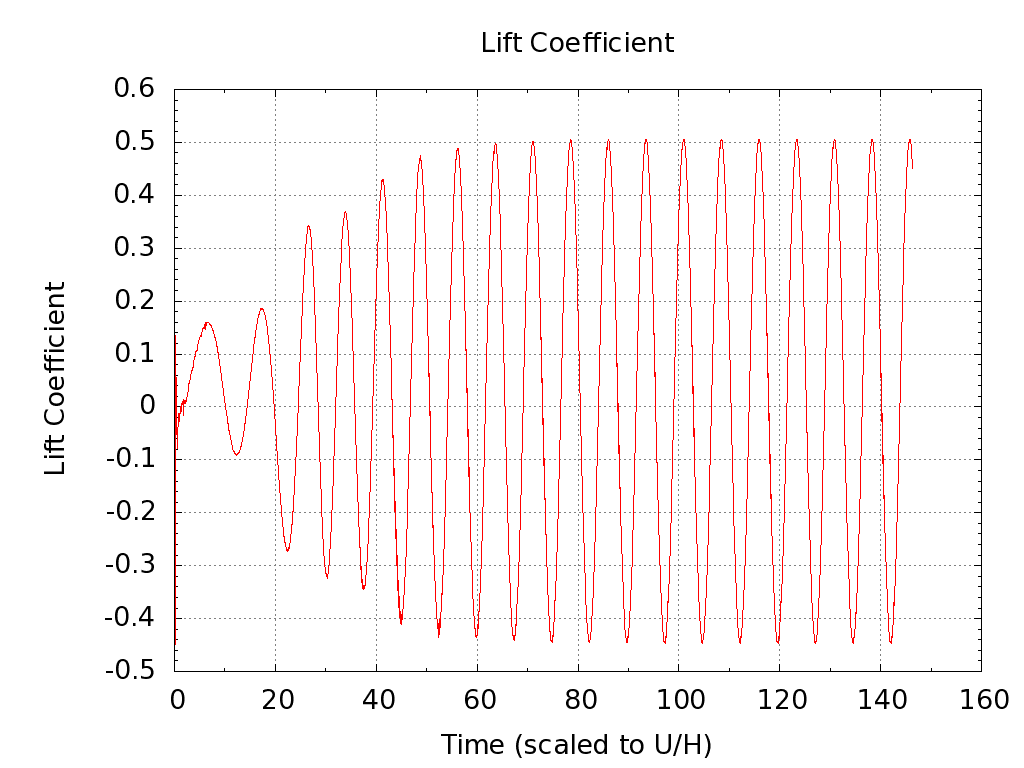

Supplement: S2 Images Folder — The numbers for each folder corresponds to the user number in phase2reports.txt. The Virtual Wind Tunnel does not generate images unless a user asks for a particular image. On a few occasions, users did not even look at certain graphs, so those graphs were not generated. Such non-inspected graphs are not present here. (Note: the x-axis label in the wake stream velocity graph, in Phase 2, due to a typo, indicated a scaling factor that was not actually applied.) (ZIP) [file pone.0134978.s010.zip › S2_imagesfolder/20/forceCoeffs.Cl.png]

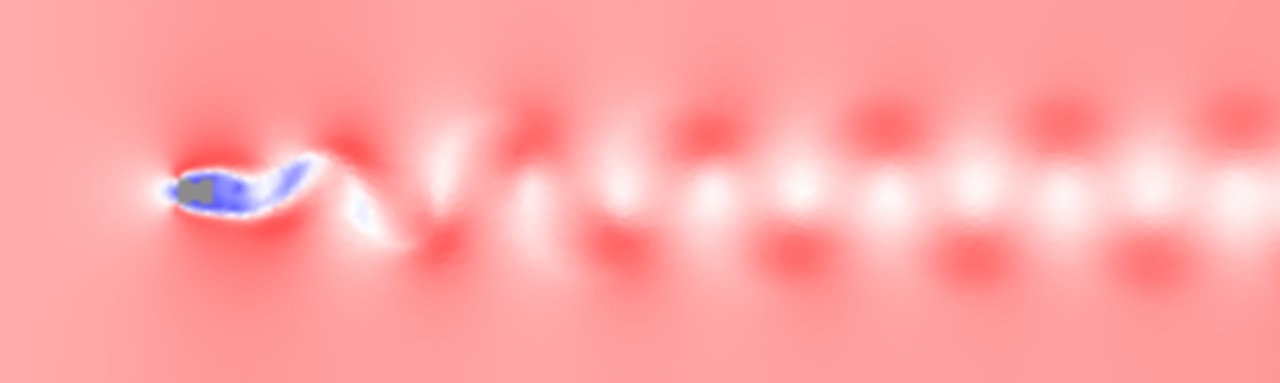

Supplement: S2 Images Folder — The numbers for each folder corresponds to the user number in phase2reports.txt. The Virtual Wind Tunnel does not generate images unless a user asks for a particular image. On a few occasions, users did not even look at certain graphs, so those graphs were not generated. Such non-inspected graphs are not present here. (Note: the x-axis label in the wake stream velocity graph, in Phase 2, due to a typo, indicated a scaling factor that was not actually applied.) (ZIP) [file pone.0134978.s010.zip › S2_imagesfolder/20/U.png]

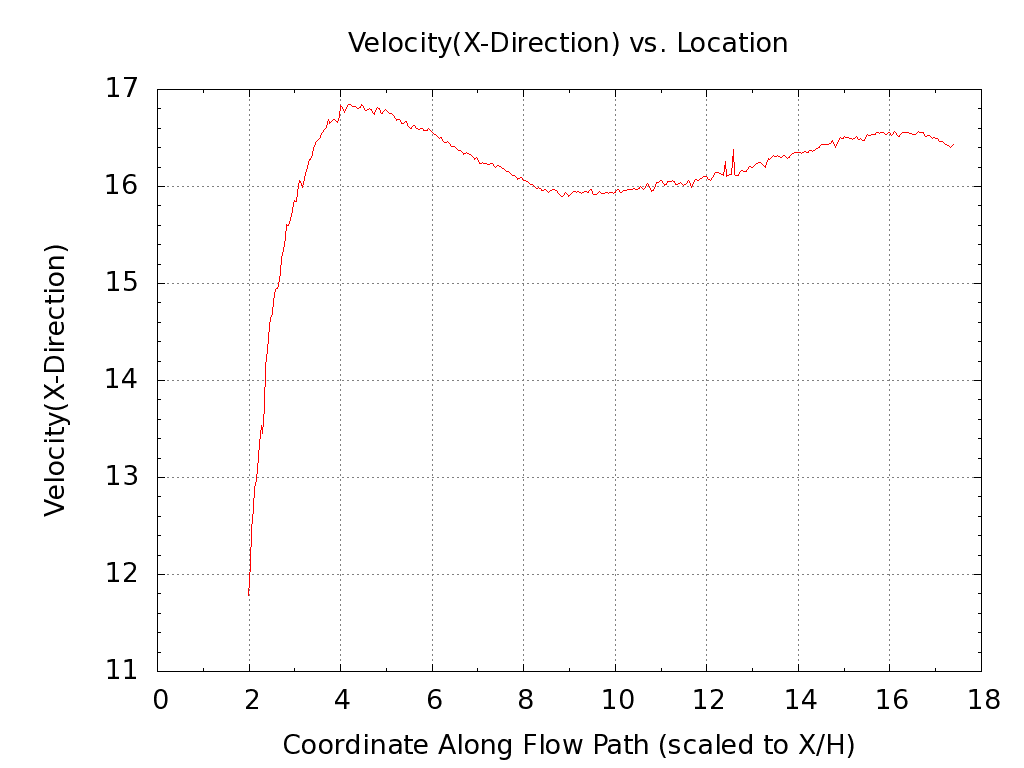

Supplement: S2 Images Folder — The numbers for each folder corresponds to the user number in phase2reports.txt. The Virtual Wind Tunnel does not generate images unless a user asks for a particular image. On a few occasions, users did not even look at certain graphs, so those graphs were not generated. Such non-inspected graphs are not present here. (Note: the x-axis label in the wake stream velocity graph, in Phase 2, due to a typo, indicated a scaling factor that was not actually applied.) (ZIP) [file pone.0134978.s010.zip › S2_imagesfolder/20/wakeCenter.UMean.X.png]

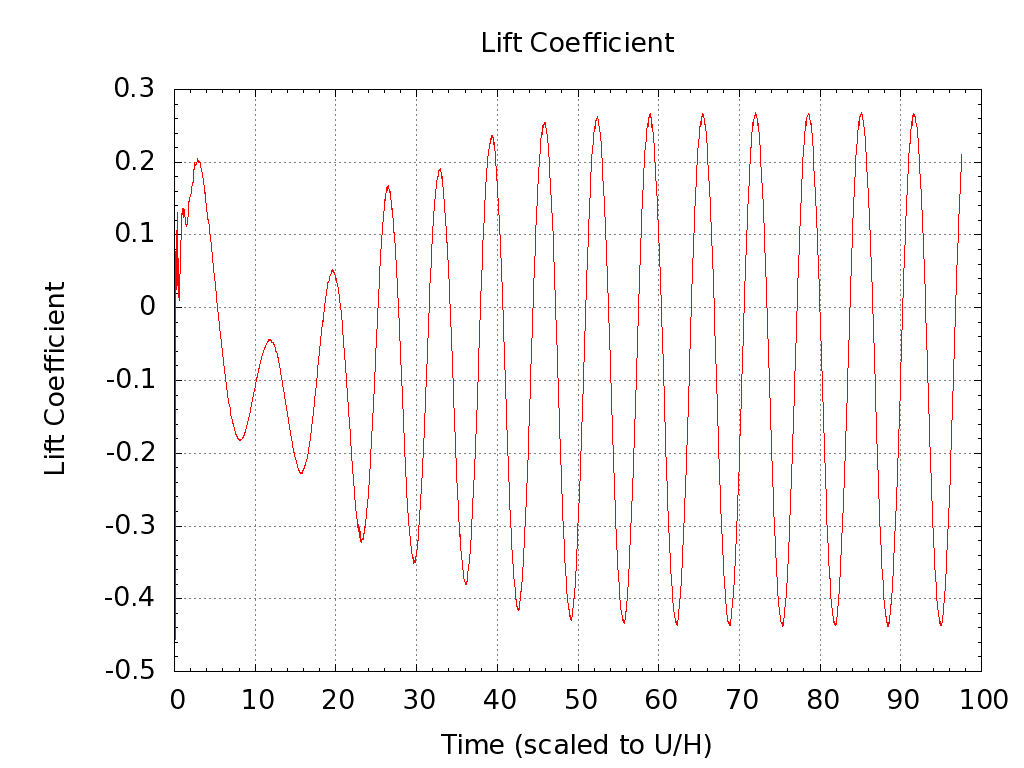

Supplement: S2 Images Folder — The numbers for each folder corresponds to the user number in phase2reports.txt. The Virtual Wind Tunnel does not generate images unless a user asks for a particular image. On a few occasions, users did not even look at certain graphs, so those graphs were not generated. Such non-inspected graphs are not present here. (Note: the x-axis label in the wake stream velocity graph, in Phase 2, due to a typo, indicated a scaling factor that was not actually applied.) (ZIP) [file pone.0134978.s010.zip › S2_imagesfolder/21/forceCoeffs.Cl.png]

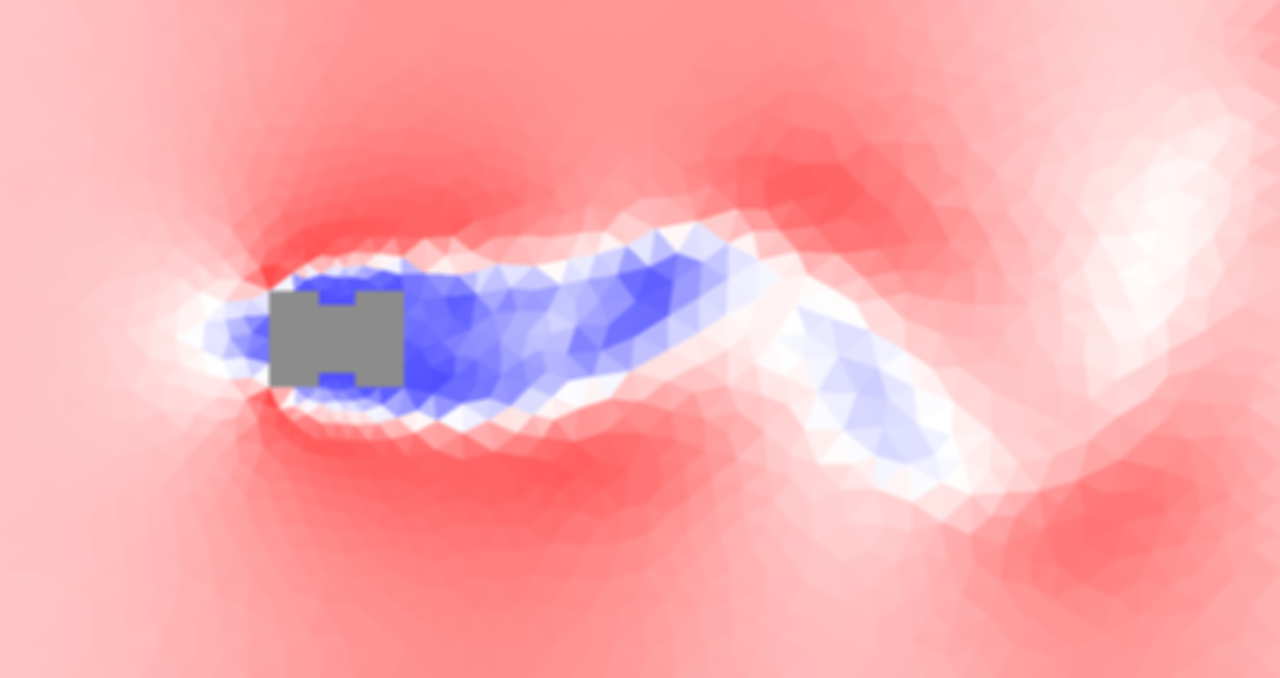

Supplement: S2 Images Folder — The numbers for each folder corresponds to the user number in phase2reports.txt. The Virtual Wind Tunnel does not generate images unless a user asks for a particular image. On a few occasions, users did not even look at certain graphs, so those graphs were not generated. Such non-inspected graphs are not present here. (Note: the x-axis label in the wake stream velocity graph, in Phase 2, due to a typo, indicated a scaling factor that was not actually applied.) (ZIP) [file pone.0134978.s010.zip › S2_imagesfolder/21/U.png]

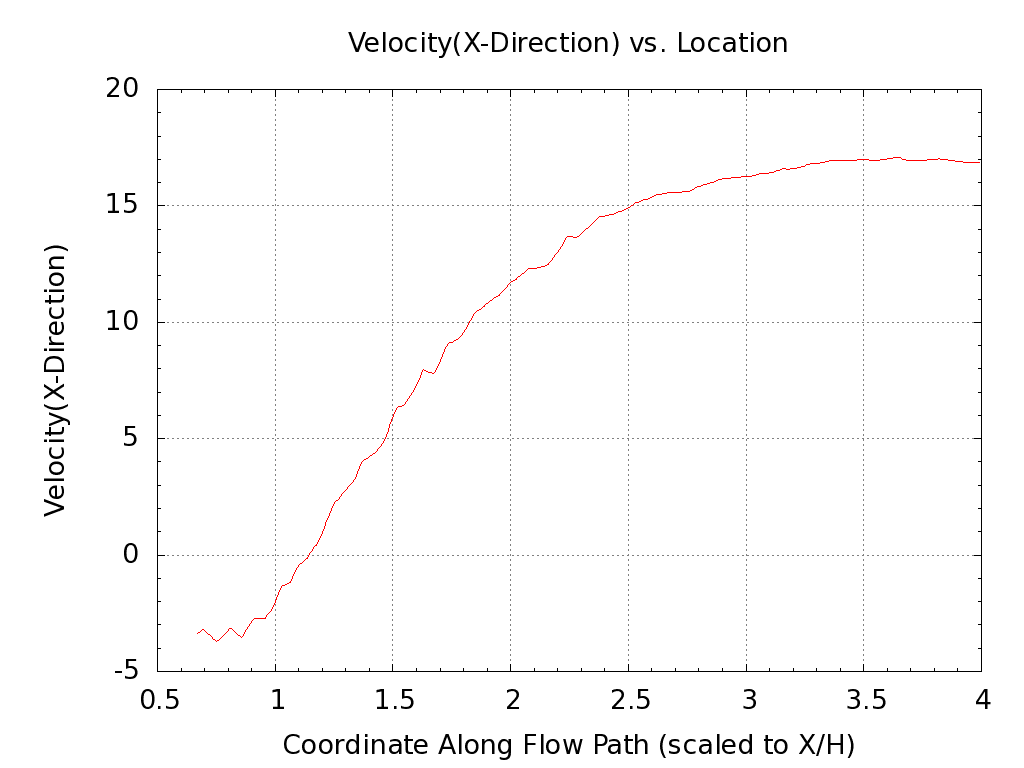

Supplement: S2 Images Folder — The numbers for each folder corresponds to the user number in phase2reports.txt. The Virtual Wind Tunnel does not generate images unless a user asks for a particular image. On a few occasions, users did not even look at certain graphs, so those graphs were not generated. Such non-inspected graphs are not present here. (Note: the x-axis label in the wake stream velocity graph, in Phase 2, due to a typo, indicated a scaling factor that was not actually applied.) (ZIP) [file pone.0134978.s010.zip › S2_imagesfolder/21/wakeCenter.UMean.X.png]

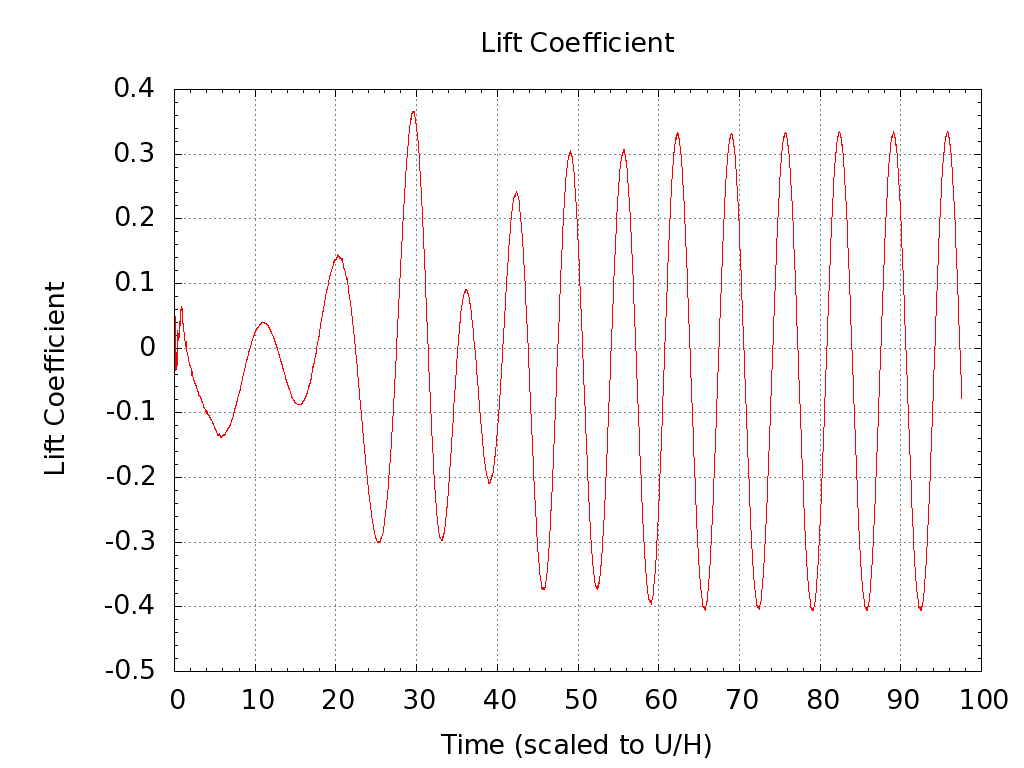

Supplement: S2 Images Folder — The numbers for each folder corresponds to the user number in phase2reports.txt. The Virtual Wind Tunnel does not generate images unless a user asks for a particular image. On a few occasions, users did not even look at certain graphs, so those graphs were not generated. Such non-inspected graphs are not present here. (Note: the x-axis label in the wake stream velocity graph, in Phase 2, due to a typo, indicated a scaling factor that was not actually applied.) (ZIP) [file pone.0134978.s010.zip › S2_imagesfolder/23/forceCoeffs.Cl.png]

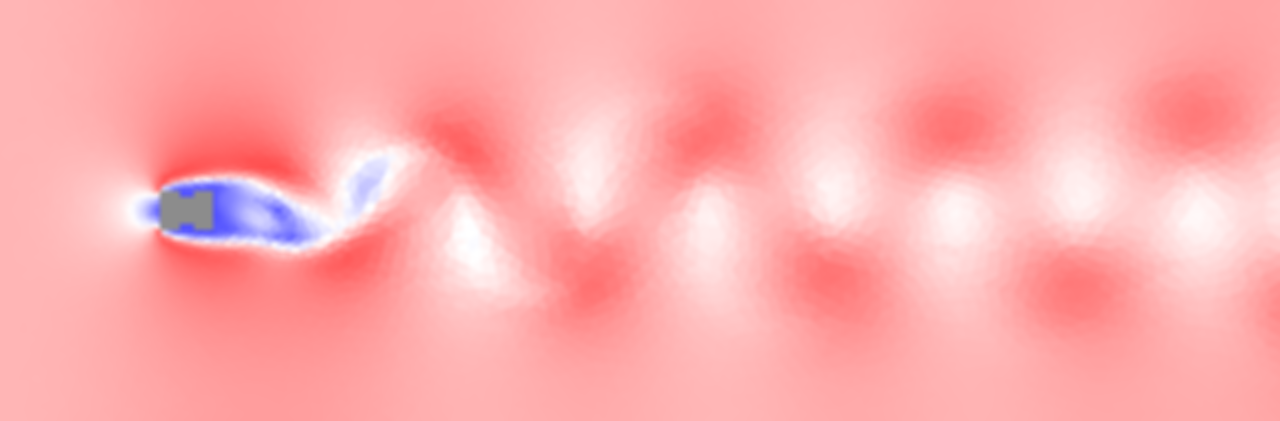

Supplement: S2 Images Folder — The numbers for each folder corresponds to the user number in phase2reports.txt. The Virtual Wind Tunnel does not generate images unless a user asks for a particular image. On a few occasions, users did not even look at certain graphs, so those graphs were not generated. Such non-inspected graphs are not present here. (Note: the x-axis label in the wake stream velocity graph, in Phase 2, due to a typo, indicated a scaling factor that was not actually applied.) (ZIP) [file pone.0134978.s010.zip › S2_imagesfolder/23/U.png]

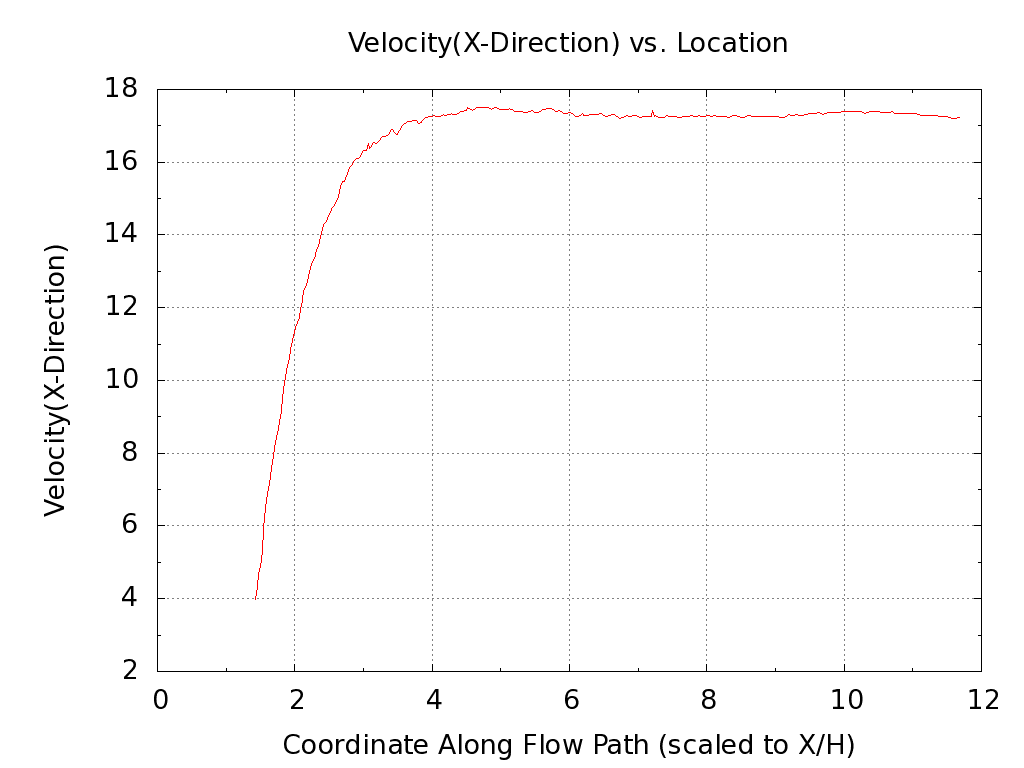

Supplement: S2 Images Folder — The numbers for each folder corresponds to the user number in phase2reports.txt. The Virtual Wind Tunnel does not generate images unless a user asks for a particular image. On a few occasions, users did not even look at certain graphs, so those graphs were not generated. Such non-inspected graphs are not present here. (Note: the x-axis label in the wake stream velocity graph, in Phase 2, due to a typo, indicated a scaling factor that was not actually applied.) (ZIP) [file pone.0134978.s010.zip › S2_imagesfolder/23/wakeCenter.UMean.X.png]

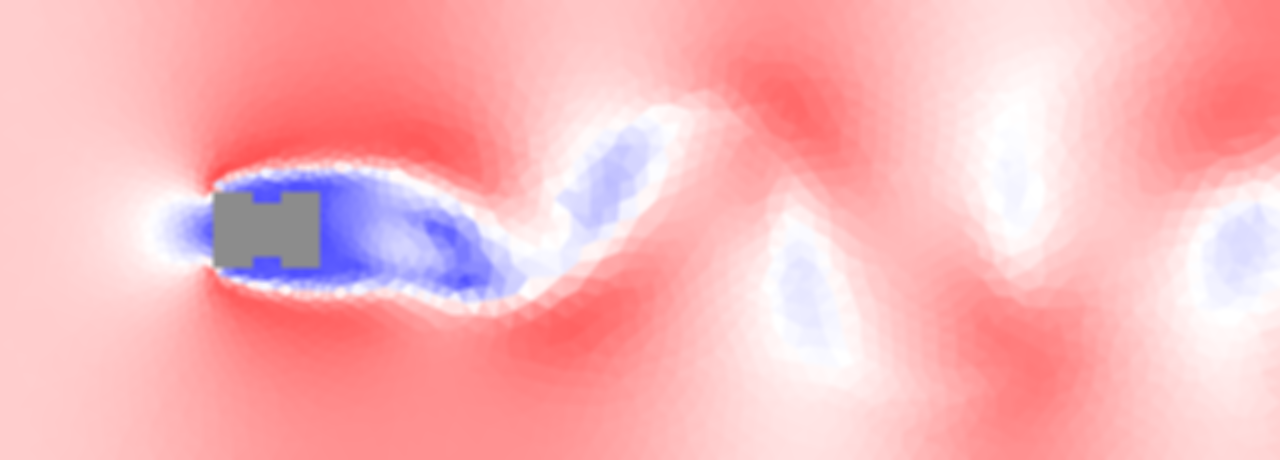

Supplement: S2 Images Folder — The numbers for each folder corresponds to the user number in phase2reports.txt. The Virtual Wind Tunnel does not generate images unless a user asks for a particular image. On a few occasions, users did not even look at certain graphs, so those graphs were not generated. Such non-inspected graphs are not present here. (Note: the x-axis label in the wake stream velocity graph, in Phase 2, due to a typo, indicated a scaling factor that was not actually applied.) (ZIP) [file pone.0134978.s010.zip › S2_imagesfolder/25/U.png]

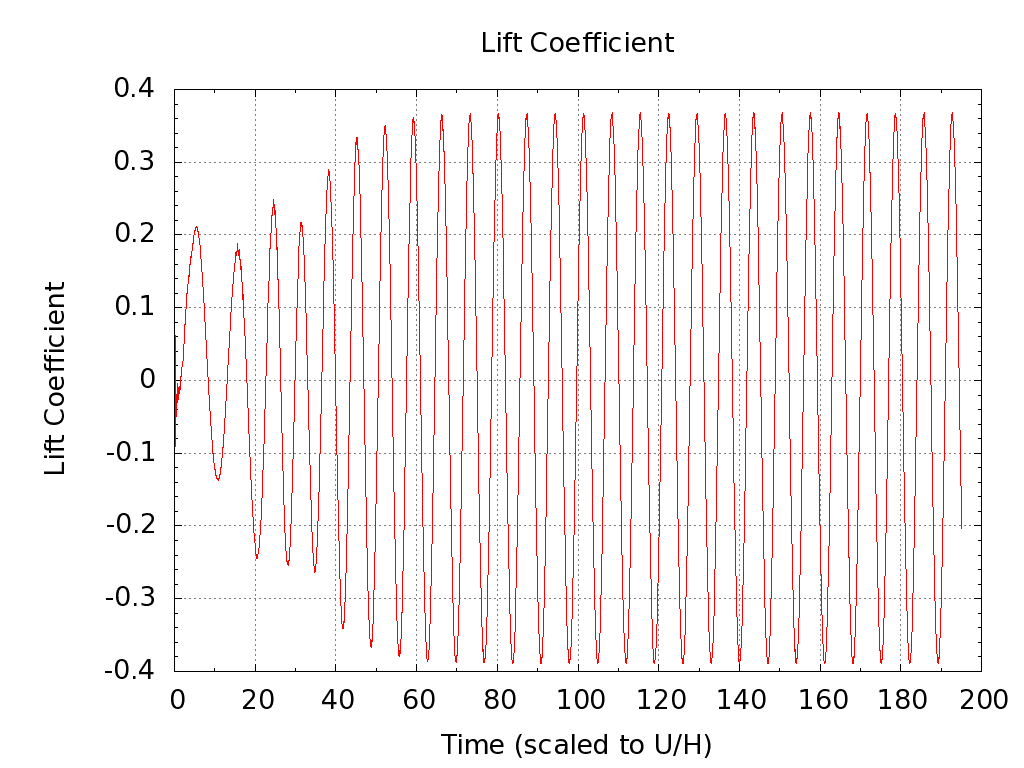

Supplement: S2 Images Folder — The numbers for each folder corresponds to the user number in phase2reports.txt. The Virtual Wind Tunnel does not generate images unless a user asks for a particular image. On a few occasions, users did not even look at certain graphs, so those graphs were not generated. Such non-inspected graphs are not present here. (Note: the x-axis label in the wake stream velocity graph, in Phase 2, due to a typo, indicated a scaling factor that was not actually applied.) (ZIP) [file pone.0134978.s010.zip › S2_imagesfolder/27/forceCoeffs.Cl.png]

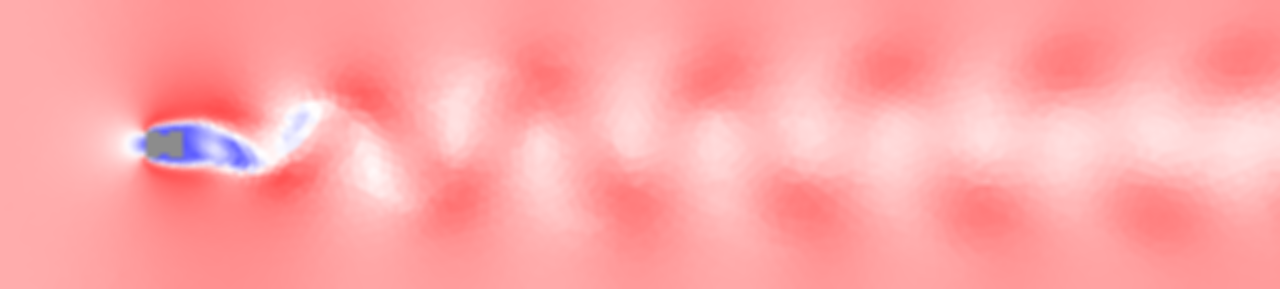

Supplement: S2 Images Folder — The numbers for each folder corresponds to the user number in phase2reports.txt. The Virtual Wind Tunnel does not generate images unless a user asks for a particular image. On a few occasions, users did not even look at certain graphs, so those graphs were not generated. Such non-inspected graphs are not present here. (Note: the x-axis label in the wake stream velocity graph, in Phase 2, due to a typo, indicated a scaling factor that was not actually applied.) (ZIP) [file pone.0134978.s010.zip › S2_imagesfolder/27/U.png]

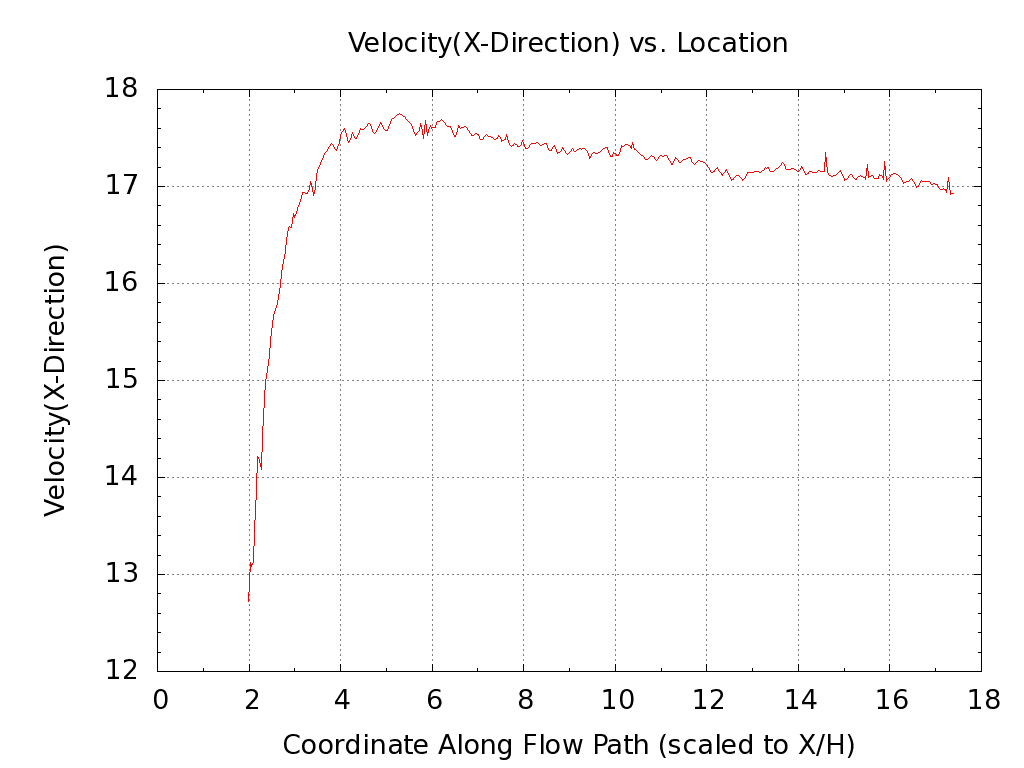

Supplement: S2 Images Folder — The numbers for each folder corresponds to the user number in phase2reports.txt. The Virtual Wind Tunnel does not generate images unless a user asks for a particular image. On a few occasions, users did not even look at certain graphs, so those graphs were not generated. Such non-inspected graphs are not present here. (Note: the x-axis label in the wake stream velocity graph, in Phase 2, due to a typo, indicated a scaling factor that was not actually applied.) (ZIP) [file pone.0134978.s010.zip › S2_imagesfolder/27/wakeCenter.UMean.X.png]

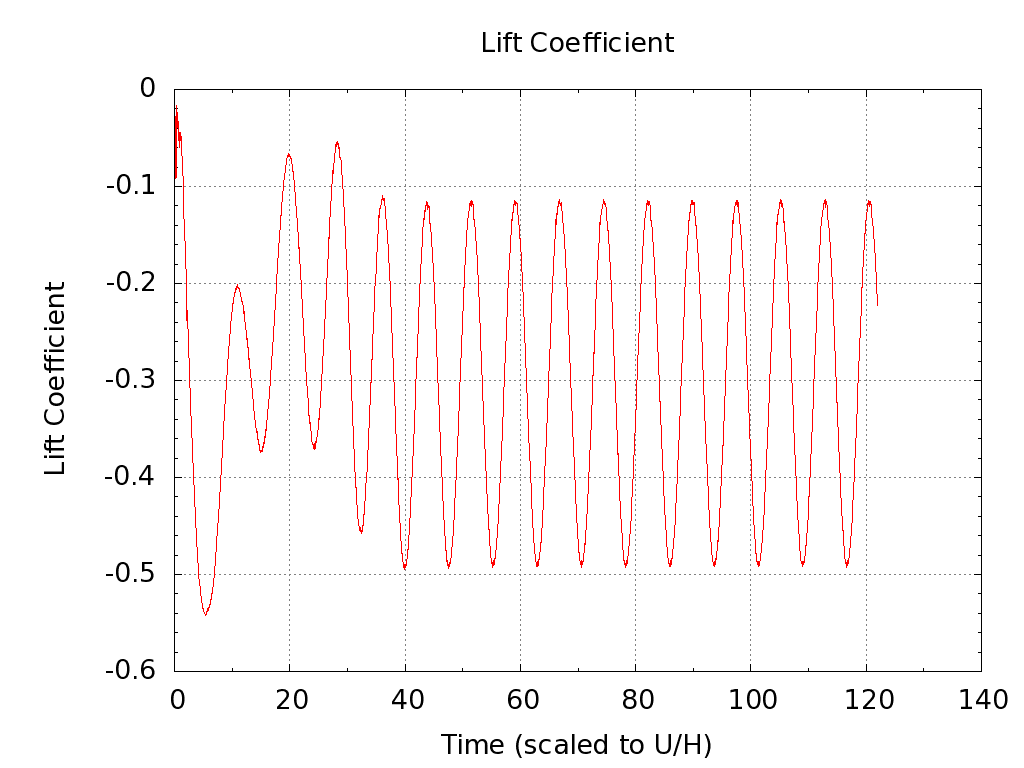

Supplement: S2 Images Folder — The numbers for each folder corresponds to the user number in phase2reports.txt. The Virtual Wind Tunnel does not generate images unless a user asks for a particular image. On a few occasions, users did not even look at certain graphs, so those graphs were not generated. Such non-inspected graphs are not present here. (Note: the x-axis label in the wake stream velocity graph, in Phase 2, due to a typo, indicated a scaling factor that was not actually applied.) (ZIP) [file pone.0134978.s010.zip › S2_imagesfolder/28/forceCoeffs.Cl.png]

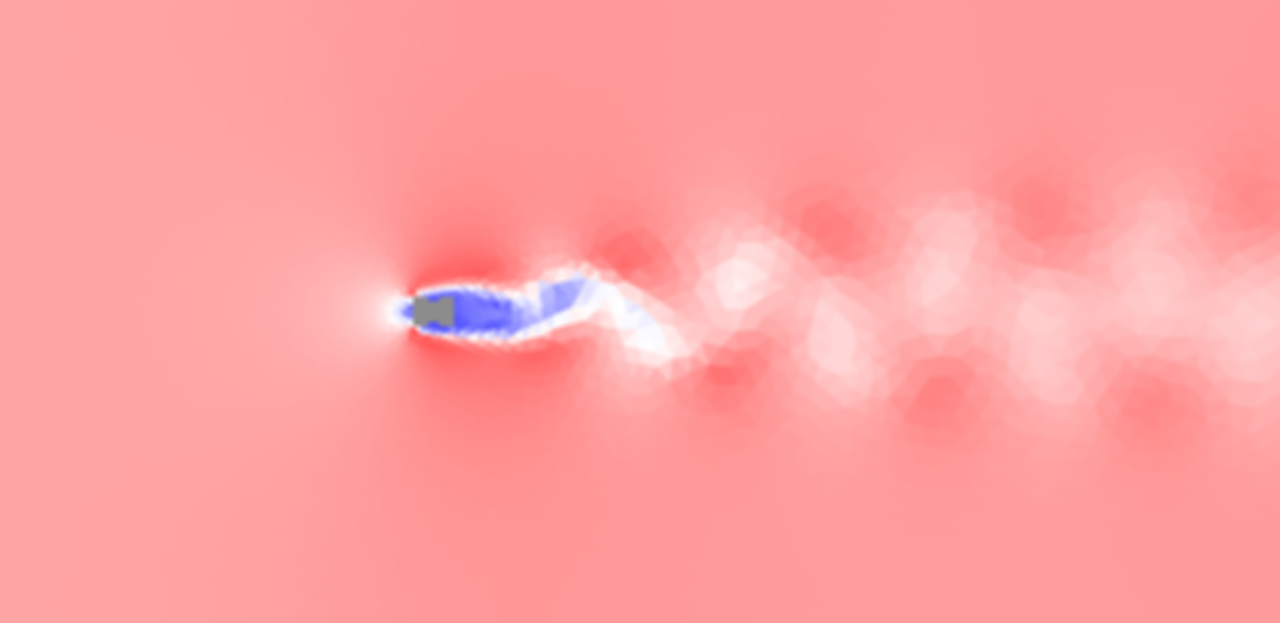

Supplement: S2 Images Folder — The numbers for each folder corresponds to the user number in phase2reports.txt. The Virtual Wind Tunnel does not generate images unless a user asks for a particular image. On a few occasions, users did not even look at certain graphs, so those graphs were not generated. Such non-inspected graphs are not present here. (Note: the x-axis label in the wake stream velocity graph, in Phase 2, due to a typo, indicated a scaling factor that was not actually applied.) (ZIP) [file pone.0134978.s010.zip › S2_imagesfolder/28/U.png]

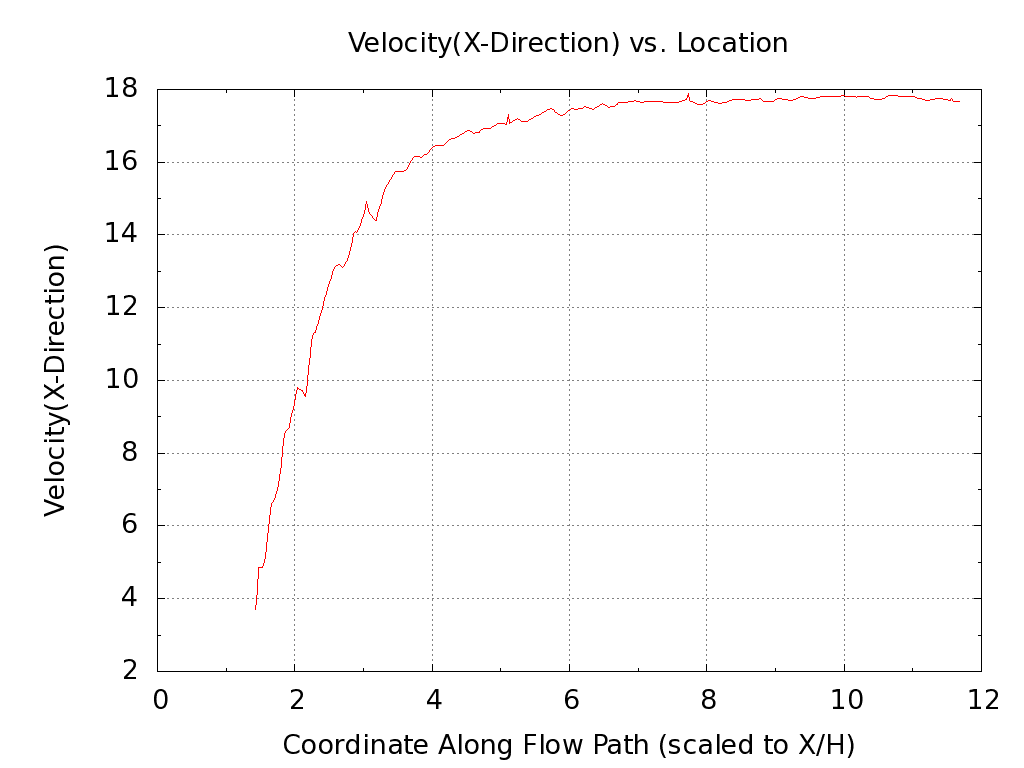

Supplement: S2 Images Folder — The numbers for each folder corresponds to the user number in phase2reports.txt. The Virtual Wind Tunnel does not generate images unless a user asks for a particular image. On a few occasions, users did not even look at certain graphs, so those graphs were not generated. Such non-inspected graphs are not present here. (Note: the x-axis label in the wake stream velocity graph, in Phase 2, due to a typo, indicated a scaling factor that was not actually applied.) (ZIP) [file pone.0134978.s010.zip › S2_imagesfolder/28/wakeCenter.UMean.X.png]

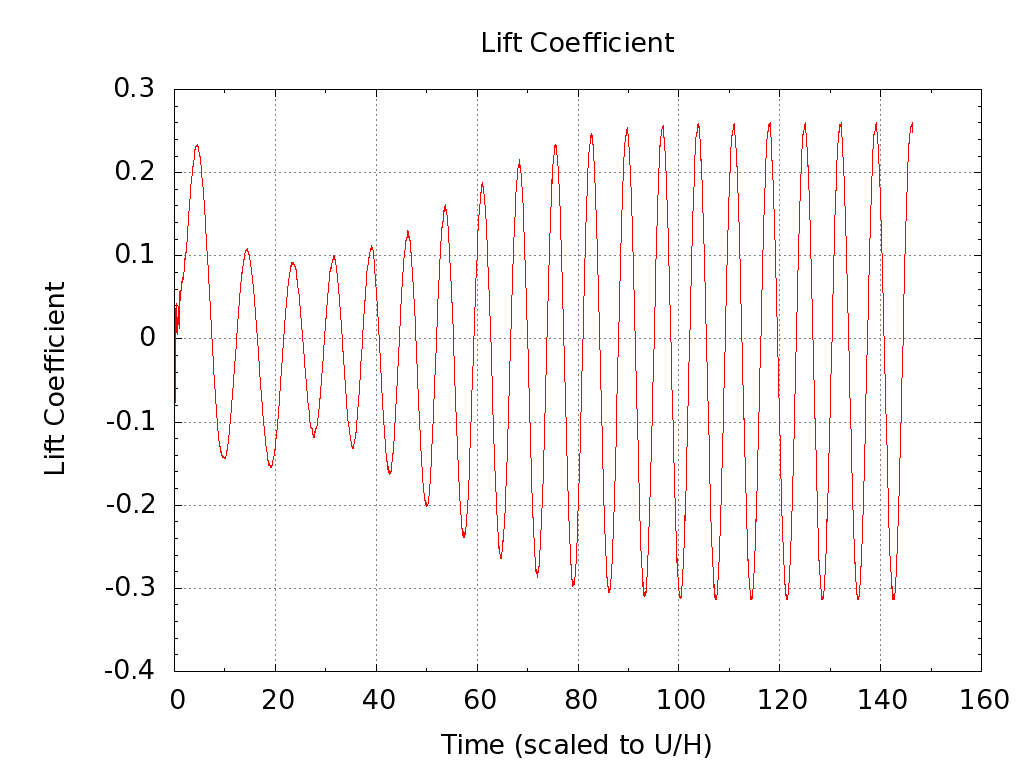

Supplement: S2 Images Folder — The numbers for each folder corresponds to the user number in phase2reports.txt. The Virtual Wind Tunnel does not generate images unless a user asks for a particular image. On a few occasions, users did not even look at certain graphs, so those graphs were not generated. Such non-inspected graphs are not present here. (Note: the x-axis label in the wake stream velocity graph, in Phase 2, due to a typo, indicated a scaling factor that was not actually applied.) (ZIP) [file pone.0134978.s010.zip › S2_imagesfolder/29/forceCoeffs.Cl.png]

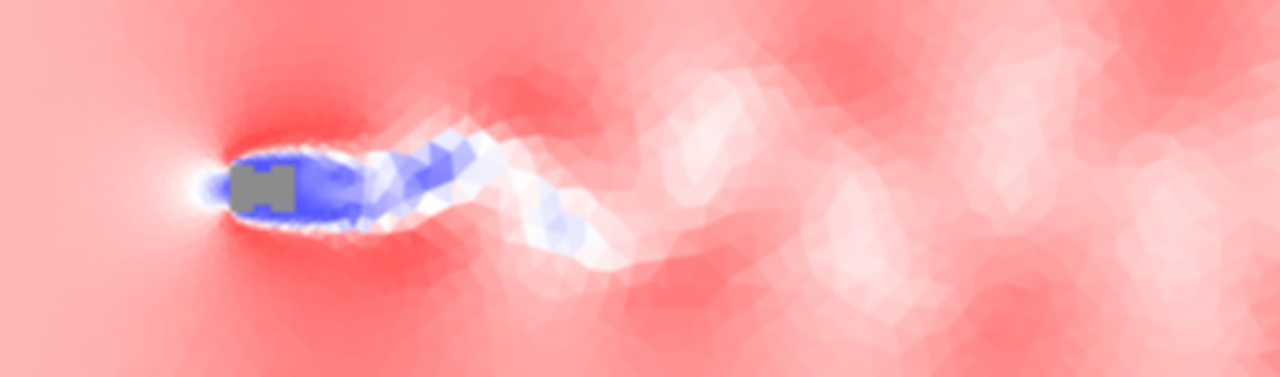

Supplement: S2 Images Folder — The numbers for each folder corresponds to the user number in phase2reports.txt. The Virtual Wind Tunnel does not generate images unless a user asks for a particular image. On a few occasions, users did not even look at certain graphs, so those graphs were not generated. Such non-inspected graphs are not present here. (Note: the x-axis label in the wake stream velocity graph, in Phase 2, due to a typo, indicated a scaling factor that was not actually applied.) (ZIP) [file pone.0134978.s010.zip › S2_imagesfolder/29/U.png]

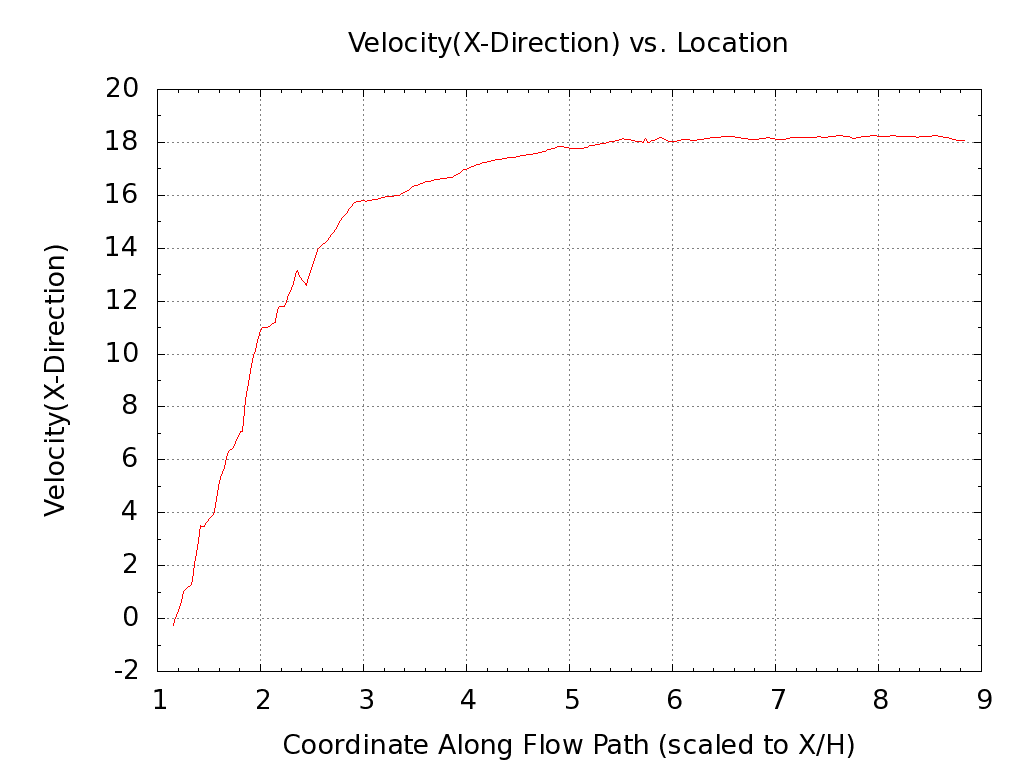

Supplement: S2 Images Folder — The numbers for each folder corresponds to the user number in phase2reports.txt. The Virtual Wind Tunnel does not generate images unless a user asks for a particular image. On a few occasions, users did not even look at certain graphs, so those graphs were not generated. Such non-inspected graphs are not present here. (Note: the x-axis label in the wake stream velocity graph, in Phase 2, due to a typo, indicated a scaling factor that was not actually applied.) (ZIP) [file pone.0134978.s010.zip › S2_imagesfolder/29/wakeCenter.UMean.X.png]

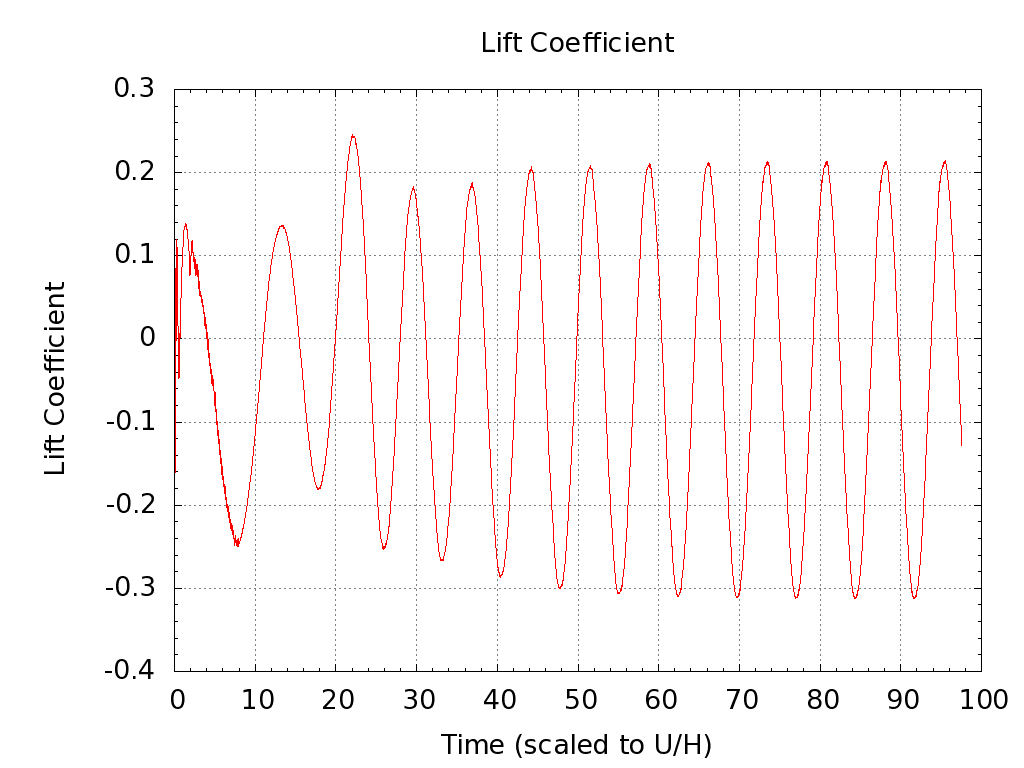

Supplement: S2 Images Folder — The numbers for each folder corresponds to the user number in phase2reports.txt. The Virtual Wind Tunnel does not generate images unless a user asks for a particular image. On a few occasions, users did not even look at certain graphs, so those graphs were not generated. Such non-inspected graphs are not present here. (Note: the x-axis label in the wake stream velocity graph, in Phase 2, due to a typo, indicated a scaling factor that was not actually applied.) (ZIP) [file pone.0134978.s010.zip › S2_imagesfolder/32/forceCoeffs.Cl.png]

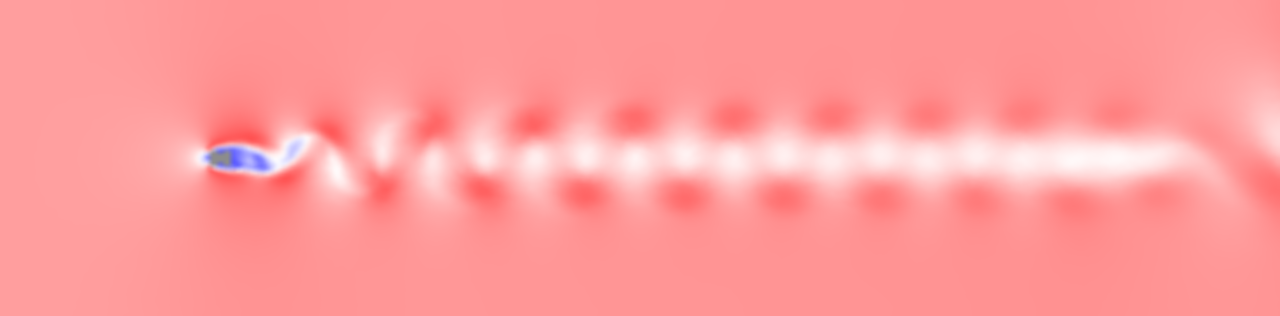

Supplement: S2 Images Folder — The numbers for each folder corresponds to the user number in phase2reports.txt. The Virtual Wind Tunnel does not generate images unless a user asks for a particular image. On a few occasions, users did not even look at certain graphs, so those graphs were not generated. Such non-inspected graphs are not present here. (Note: the x-axis label in the wake stream velocity graph, in Phase 2, due to a typo, indicated a scaling factor that was not actually applied.) (ZIP) [file pone.0134978.s010.zip › S2_imagesfolder/32/U.png]

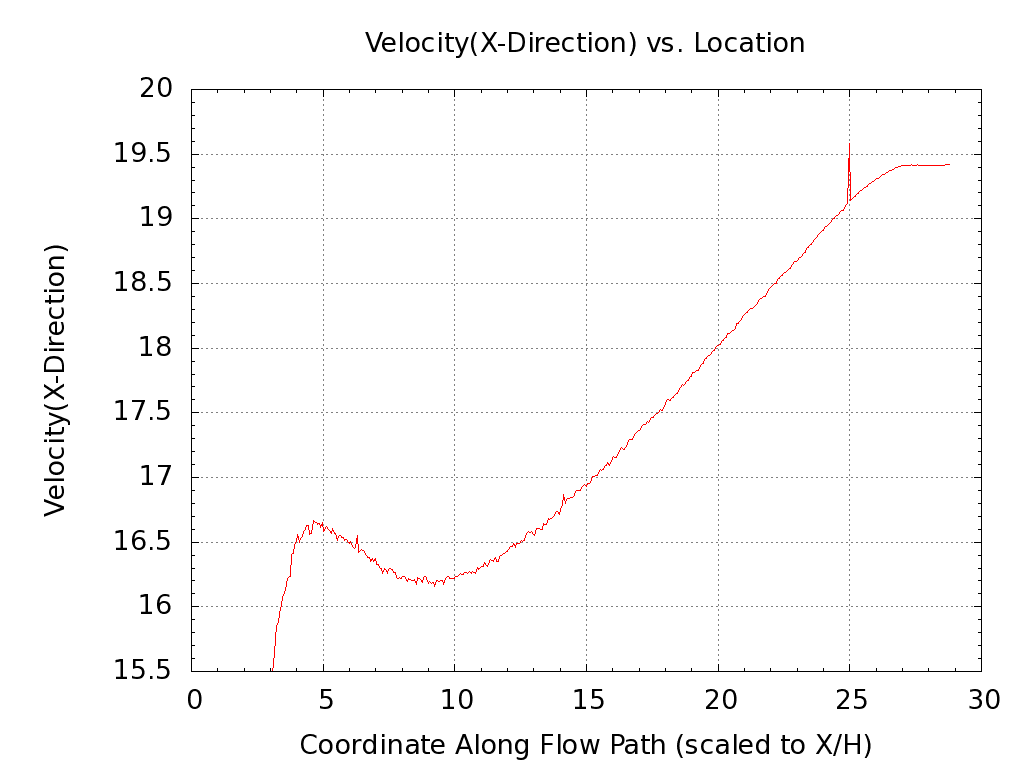

Supplement: S2 Images Folder — The numbers for each folder corresponds to the user number in phase2reports.txt. The Virtual Wind Tunnel does not generate images unless a user asks for a particular image. On a few occasions, users did not even look at certain graphs, so those graphs were not generated. Such non-inspected graphs are not present here. (Note: the x-axis label in the wake stream velocity graph, in Phase 2, due to a typo, indicated a scaling factor that was not actually applied.) (ZIP) [file pone.0134978.s010.zip › S2_imagesfolder/32/wakeCenter.UMean.X.png]

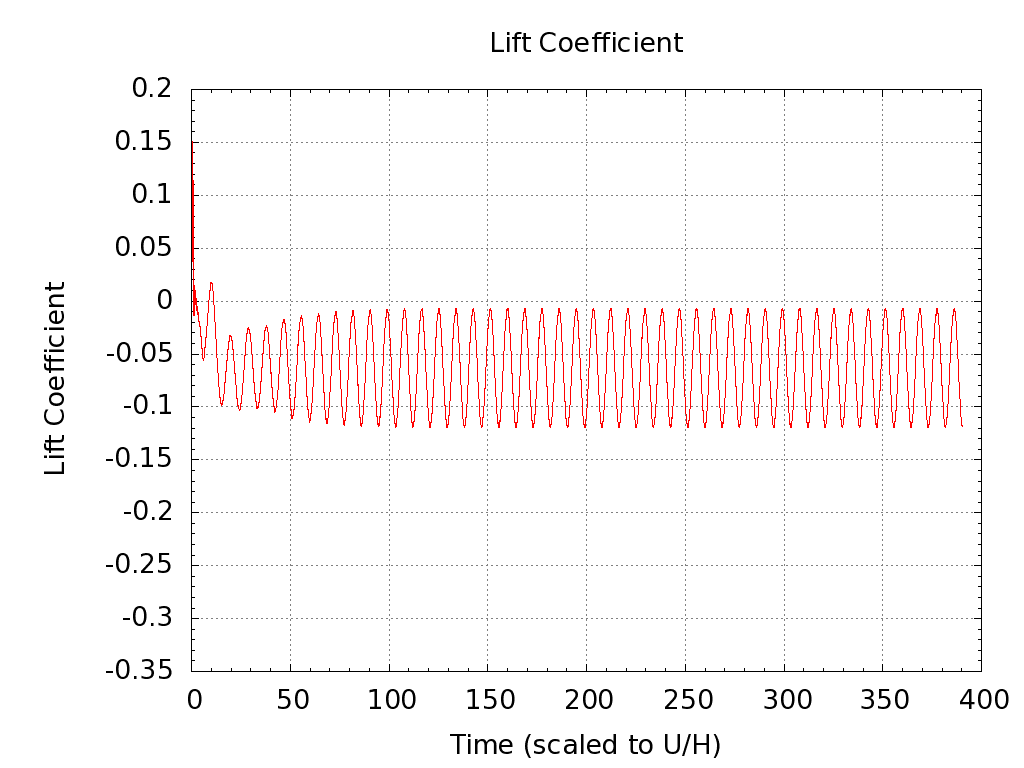

Supplement: S2 Images Folder — The numbers for each folder corresponds to the user number in phase2reports.txt. The Virtual Wind Tunnel does not generate images unless a user asks for a particular image. On a few occasions, users did not even look at certain graphs, so those graphs were not generated. Such non-inspected graphs are not present here. (Note: the x-axis label in the wake stream velocity graph, in Phase 2, due to a typo, indicated a scaling factor that was not actually applied.) (ZIP) [file pone.0134978.s010.zip › S2_imagesfolder/34/forceCoeffs.Cl.png]

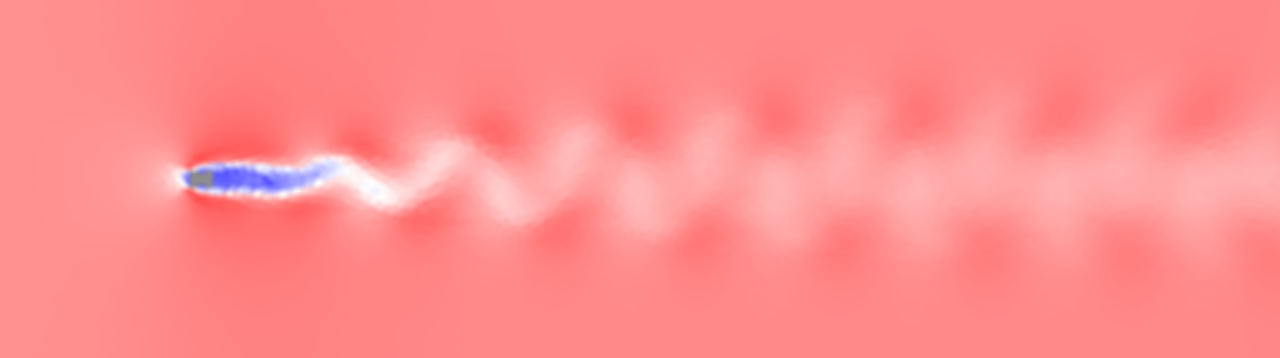

Supplement: S2 Images Folder — The numbers for each folder corresponds to the user number in phase2reports.txt. The Virtual Wind Tunnel does not generate images unless a user asks for a particular image. On a few occasions, users did not even look at certain graphs, so those graphs were not generated. Such non-inspected graphs are not present here. (Note: the x-axis label in the wake stream velocity graph, in Phase 2, due to a typo, indicated a scaling factor that was not actually applied.) (ZIP) [file pone.0134978.s010.zip › S2_imagesfolder/34/U.png]

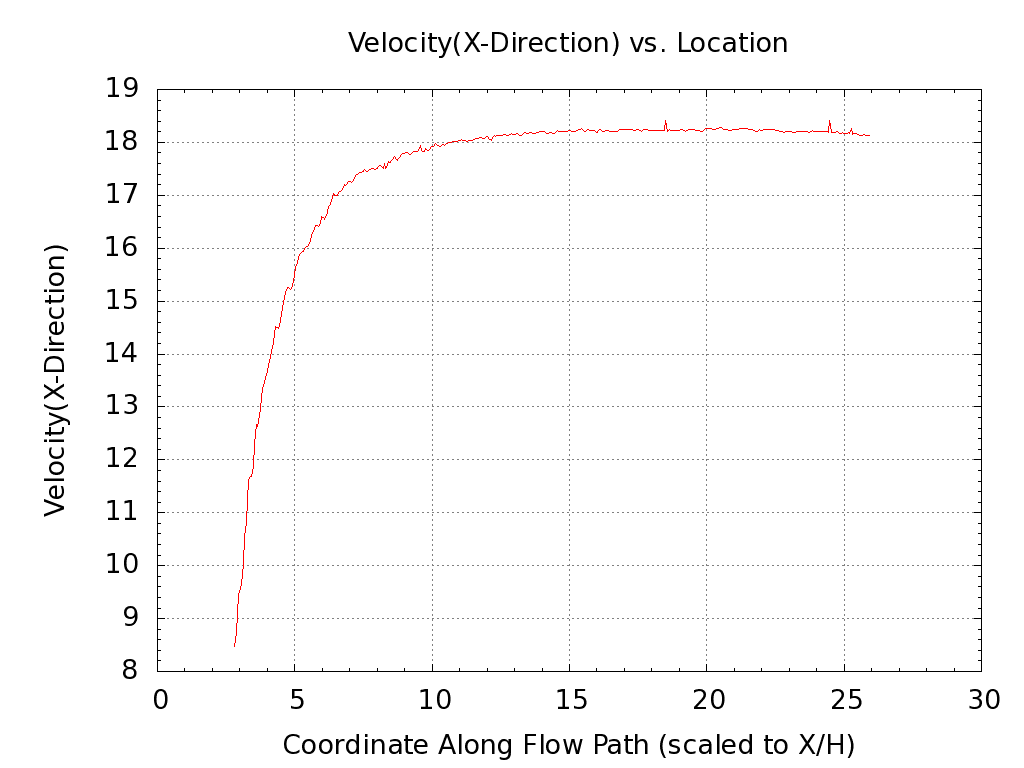

Supplement: S2 Images Folder — The numbers for each folder corresponds to the user number in phase2reports.txt. The Virtual Wind Tunnel does not generate images unless a user asks for a particular image. On a few occasions, users did not even look at certain graphs, so those graphs were not generated. Such non-inspected graphs are not present here. (Note: the x-axis label in the wake stream velocity graph, in Phase 2, due to a typo, indicated a scaling factor that was not actually applied.) (ZIP) [file pone.0134978.s010.zip › S2_imagesfolder/34/wakeCenter.UMean.X.png]

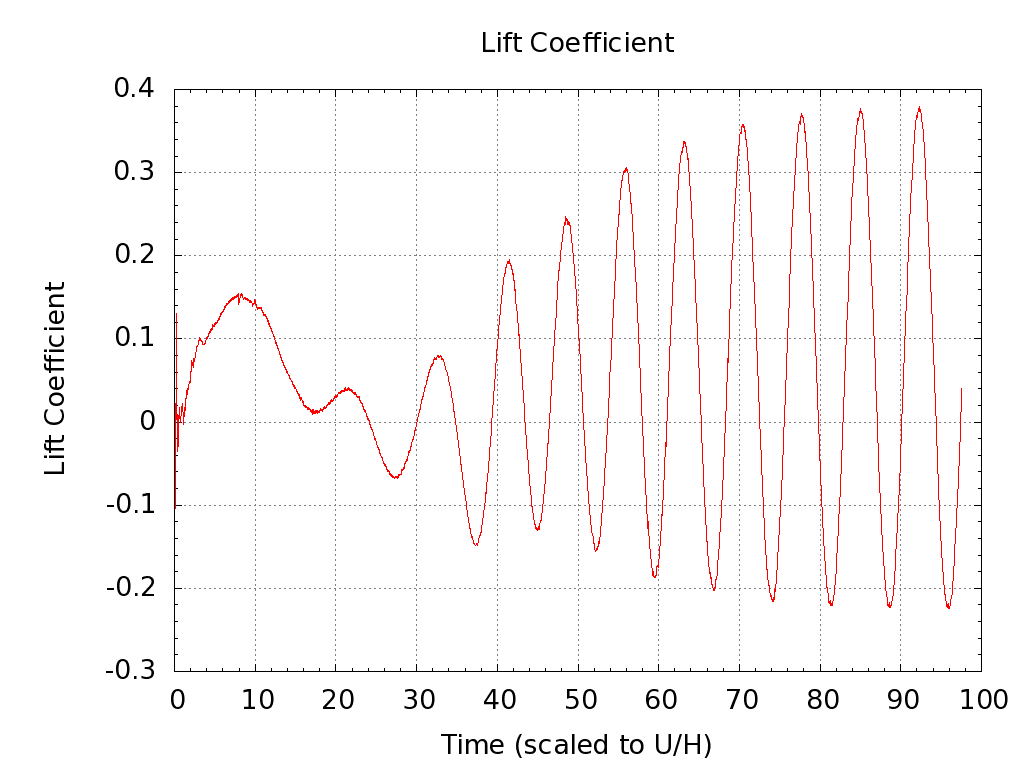

Supplement: S2 Images Folder — The numbers for each folder corresponds to the user number in phase2reports.txt. The Virtual Wind Tunnel does not generate images unless a user asks for a particular image. On a few occasions, users did not even look at certain graphs, so those graphs were not generated. Such non-inspected graphs are not present here. (Note: the x-axis label in the wake stream velocity graph, in Phase 2, due to a typo, indicated a scaling factor that was not actually applied.) (ZIP) [file pone.0134978.s010.zip › S2_imagesfolder/35/forceCoeffs.Cl.png]

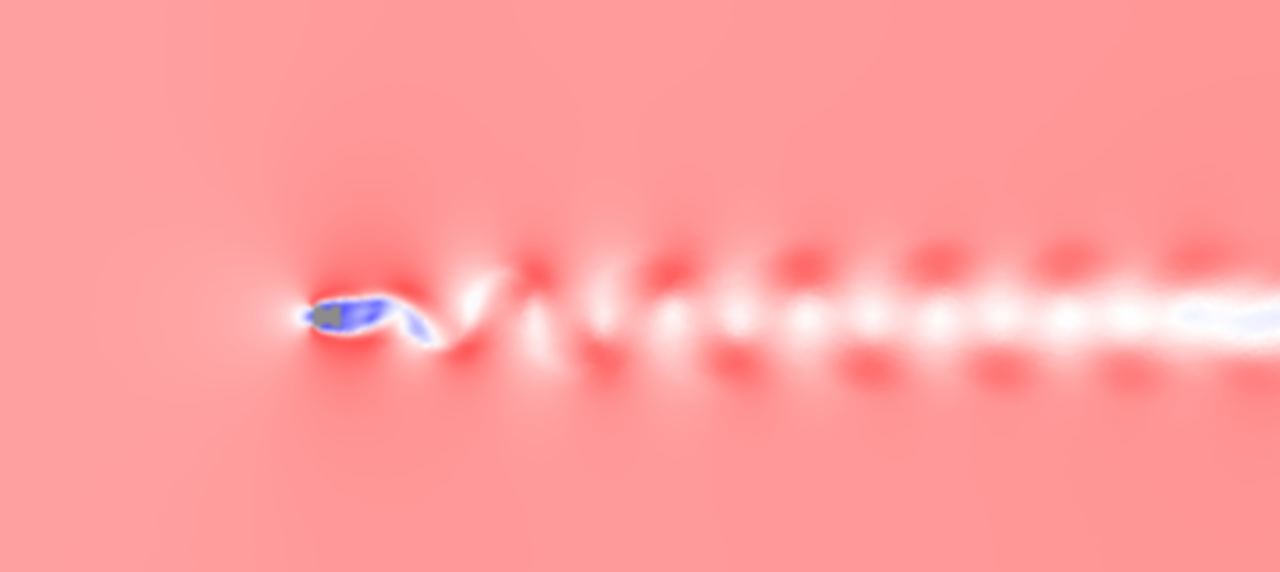

Supplement: S2 Images Folder — The numbers for each folder corresponds to the user number in phase2reports.txt. The Virtual Wind Tunnel does not generate images unless a user asks for a particular image. On a few occasions, users did not even look at certain graphs, so those graphs were not generated. Such non-inspected graphs are not present here. (Note: the x-axis label in the wake stream velocity graph, in Phase 2, due to a typo, indicated a scaling factor that was not actually applied.) (ZIP) [file pone.0134978.s010.zip › S2_imagesfolder/35/U.png]

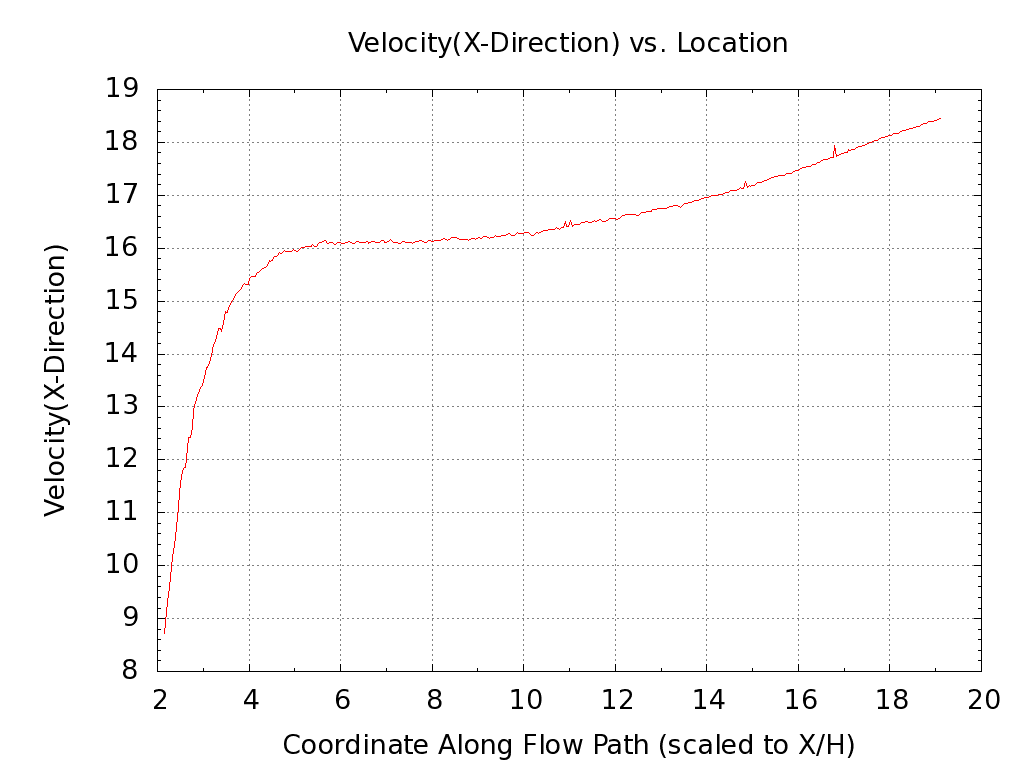

Supplement: S2 Images Folder — The numbers for each folder corresponds to the user number in phase2reports.txt. The Virtual Wind Tunnel does not generate images unless a user asks for a particular image. On a few occasions, users did not even look at certain graphs, so those graphs were not generated. Such non-inspected graphs are not present here. (Note: the x-axis label in the wake stream velocity graph, in Phase 2, due to a typo, indicated a scaling factor that was not actually applied.) (ZIP) [file pone.0134978.s010.zip › S2_imagesfolder/35/wakeCenter.UMean.X.png]

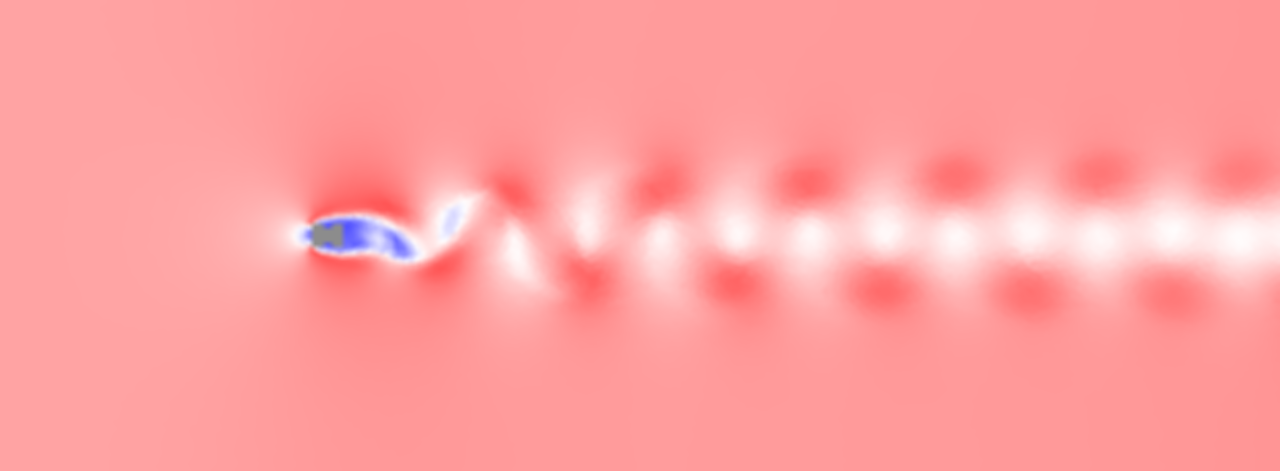

Supplement: S2 Images Folder — The numbers for each folder corresponds to the user number in phase2reports.txt. The Virtual Wind Tunnel does not generate images unless a user asks for a particular image. On a few occasions, users did not even look at certain graphs, so those graphs were not generated. Such non-inspected graphs are not present here. (Note: the x-axis label in the wake stream velocity graph, in Phase 2, due to a typo, indicated a scaling factor that was not actually applied.) (ZIP) [file pone.0134978.s010.zip › S2_imagesfolder/36/U.png]

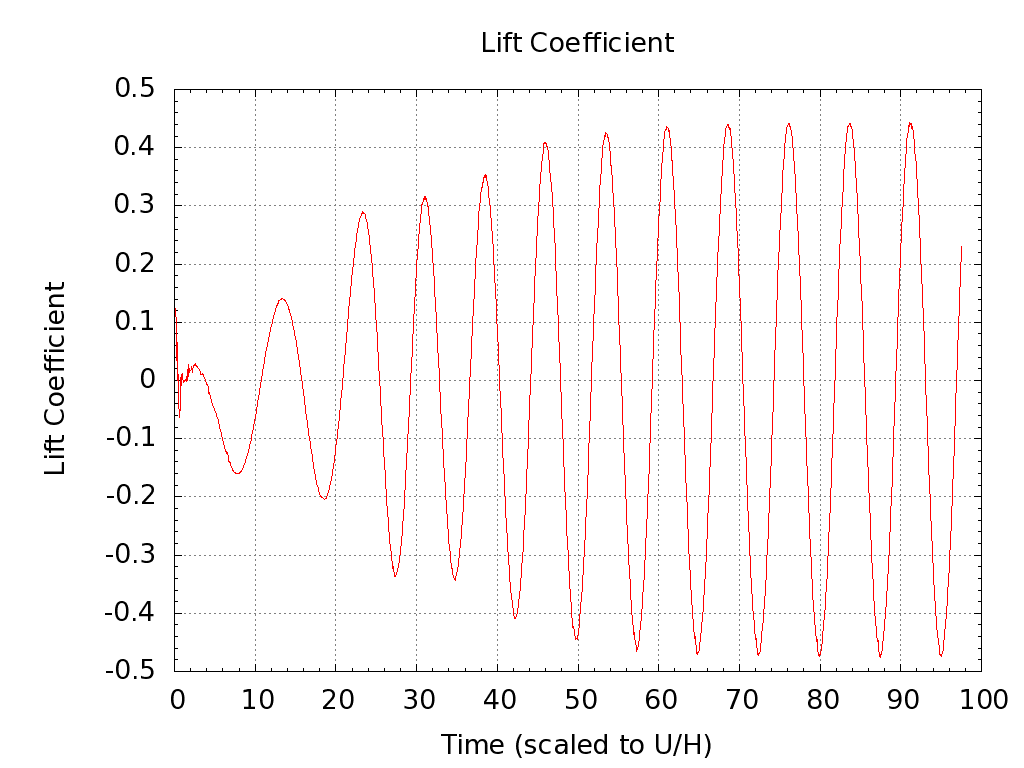

Supplement: S2 Images Folder — The numbers for each folder corresponds to the user number in phase2reports.txt. The Virtual Wind Tunnel does not generate images unless a user asks for a particular image. On a few occasions, users did not even look at certain graphs, so those graphs were not generated. Such non-inspected graphs are not present here. (Note: the x-axis label in the wake stream velocity graph, in Phase 2, due to a typo, indicated a scaling factor that was not actually applied.) (ZIP) [file pone.0134978.s010.zip › S2_imagesfolder/37/forceCoeffs.Cl.png]

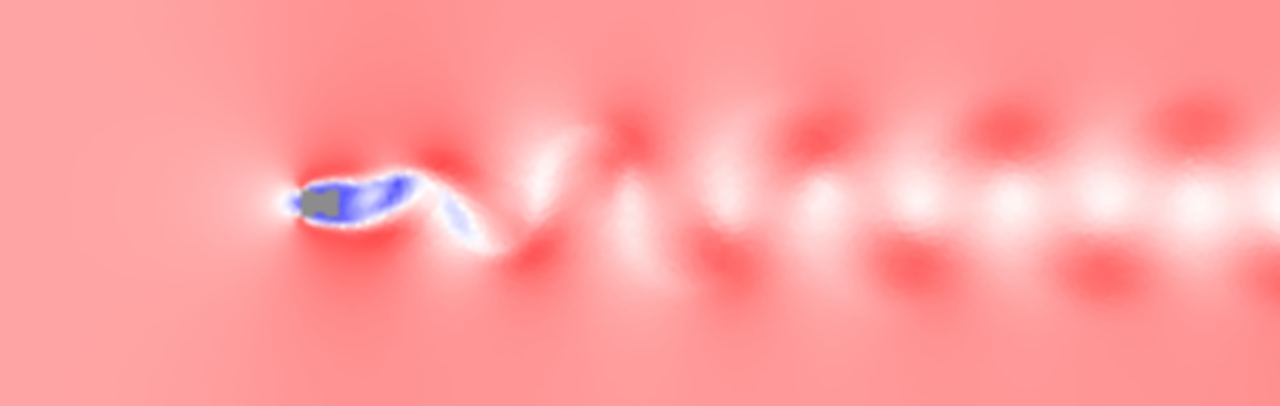

Supplement: S2 Images Folder — The numbers for each folder corresponds to the user number in phase2reports.txt. The Virtual Wind Tunnel does not generate images unless a user asks for a particular image. On a few occasions, users did not even look at certain graphs, so those graphs were not generated. Such non-inspected graphs are not present here. (Note: the x-axis label in the wake stream velocity graph, in Phase 2, due to a typo, indicated a scaling factor that was not actually applied.) (ZIP) [file pone.0134978.s010.zip › S2_imagesfolder/37/U.png]

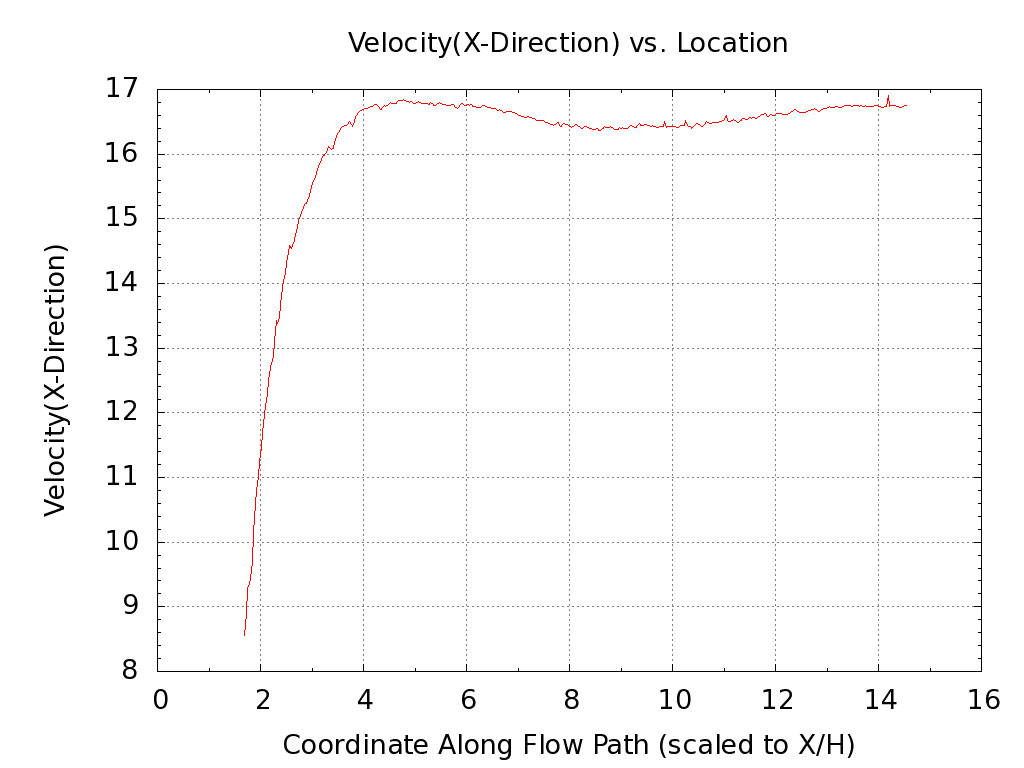

Supplement: S2 Images Folder — The numbers for each folder corresponds to the user number in phase2reports.txt. The Virtual Wind Tunnel does not generate images unless a user asks for a particular image. On a few occasions, users did not even look at certain graphs, so those graphs were not generated. Such non-inspected graphs are not present here. (Note: the x-axis label in the wake stream velocity graph, in Phase 2, due to a typo, indicated a scaling factor that was not actually applied.) (ZIP) [file pone.0134978.s010.zip › S2_imagesfolder/37/wakeCenter.UMean.X.png]

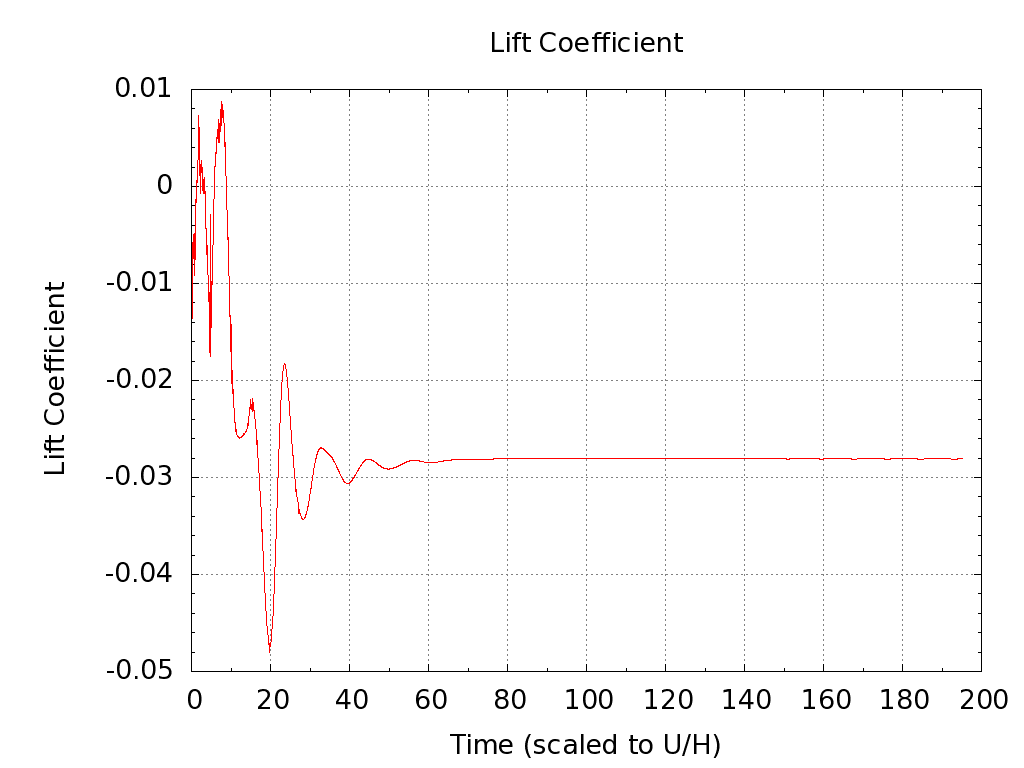

Supplement: S2 Images Folder — The numbers for each folder corresponds to the user number in phase2reports.txt. The Virtual Wind Tunnel does not generate images unless a user asks for a particular image. On a few occasions, users did not even look at certain graphs, so those graphs were not generated. Such non-inspected graphs are not present here. (Note: the x-axis label in the wake stream velocity graph, in Phase 2, due to a typo, indicated a scaling factor that was not actually applied.) (ZIP) [file pone.0134978.s010.zip › S2_imagesfolder/38/forceCoeffs.Cl.png]

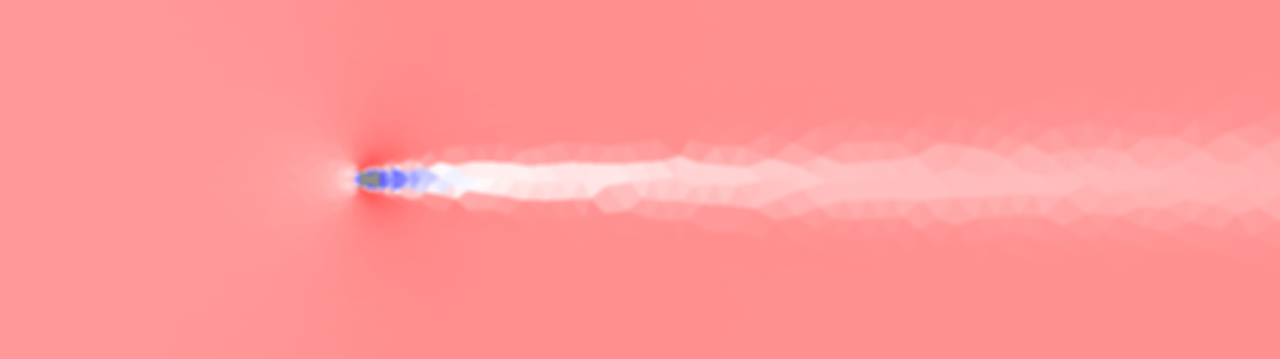

Supplement: S2 Images Folder — The numbers for each folder corresponds to the user number in phase2reports.txt. The Virtual Wind Tunnel does not generate images unless a user asks for a particular image. On a few occasions, users did not even look at certain graphs, so those graphs were not generated. Such non-inspected graphs are not present here. (Note: the x-axis label in the wake stream velocity graph, in Phase 2, due to a typo, indicated a scaling factor that was not actually applied.) (ZIP) [file pone.0134978.s010.zip › S2_imagesfolder/38/U.png]

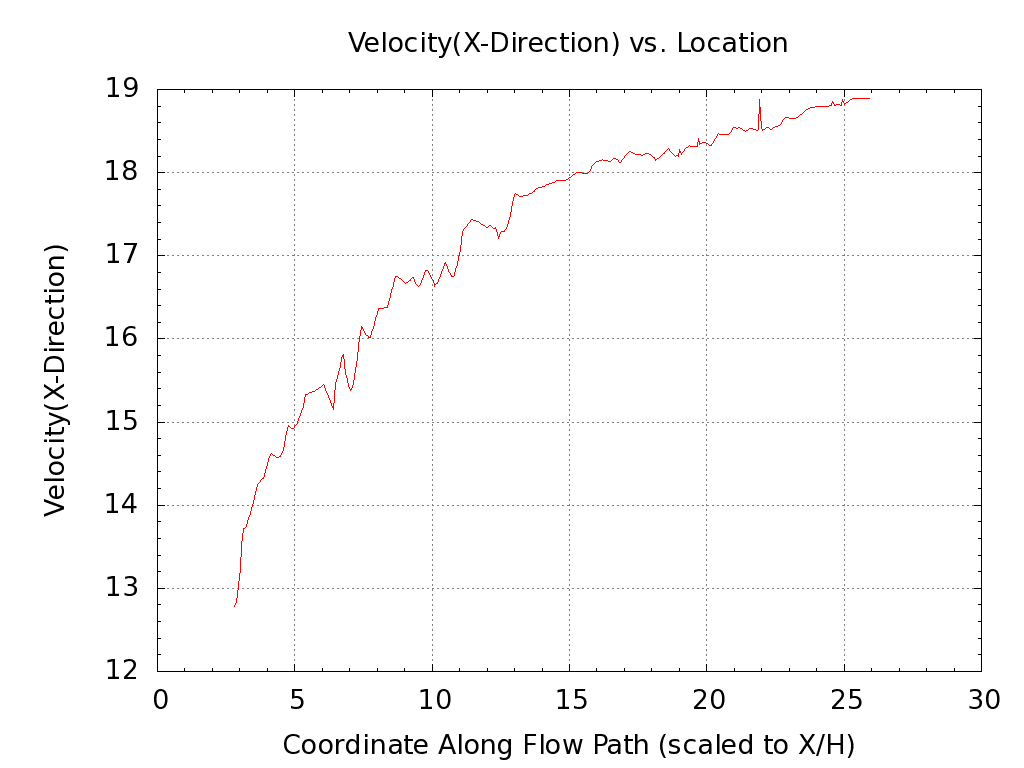

Supplement: S2 Images Folder — The numbers for each folder corresponds to the user number in phase2reports.txt. The Virtual Wind Tunnel does not generate images unless a user asks for a particular image. On a few occasions, users did not even look at certain graphs, so those graphs were not generated. Such non-inspected graphs are not present here. (Note: the x-axis label in the wake stream velocity graph, in Phase 2, due to a typo, indicated a scaling factor that was not actually applied.) (ZIP) [file pone.0134978.s010.zip › S2_imagesfolder/38/wakeCenter.UMean.X.png]

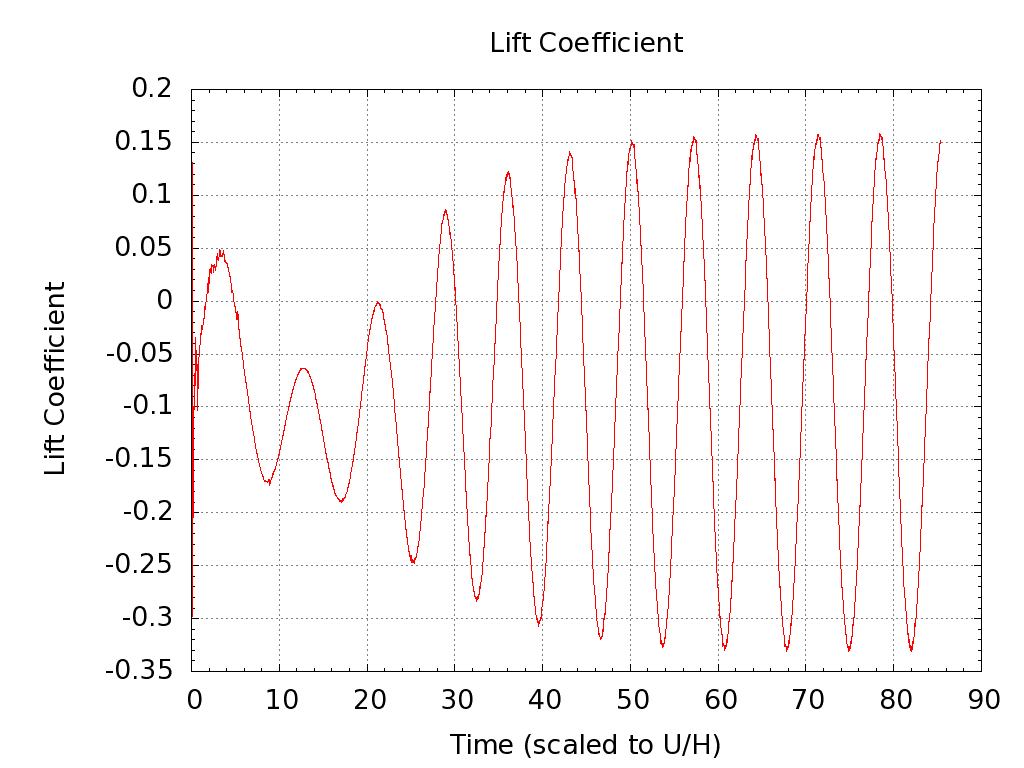

Supplement: S2 Images Folder — The numbers for each folder corresponds to the user number in phase2reports.txt. The Virtual Wind Tunnel does not generate images unless a user asks for a particular image. On a few occasions, users did not even look at certain graphs, so those graphs were not generated. Such non-inspected graphs are not present here. (Note: the x-axis label in the wake stream velocity graph, in Phase 2, due to a typo, indicated a scaling factor that was not actually applied.) (ZIP) [file pone.0134978.s010.zip › S2_imagesfolder/39/forceCoeffs.Cl.png]

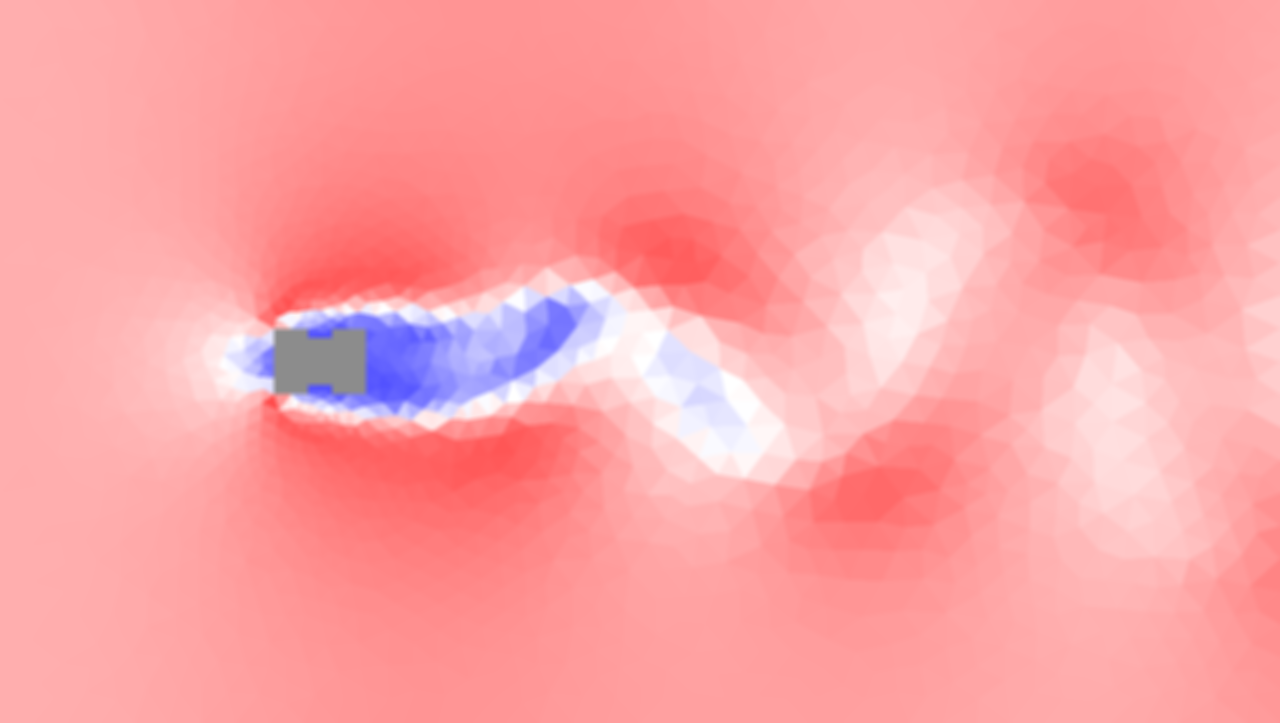

Supplement: S2 Images Folder — The numbers for each folder corresponds to the user number in phase2reports.txt. The Virtual Wind Tunnel does not generate images unless a user asks for a particular image. On a few occasions, users did not even look at certain graphs, so those graphs were not generated. Such non-inspected graphs are not present here. (Note: the x-axis label in the wake stream velocity graph, in Phase 2, due to a typo, indicated a scaling factor that was not actually applied.) (ZIP) [file pone.0134978.s010.zip › S2_imagesfolder/39/U.png]

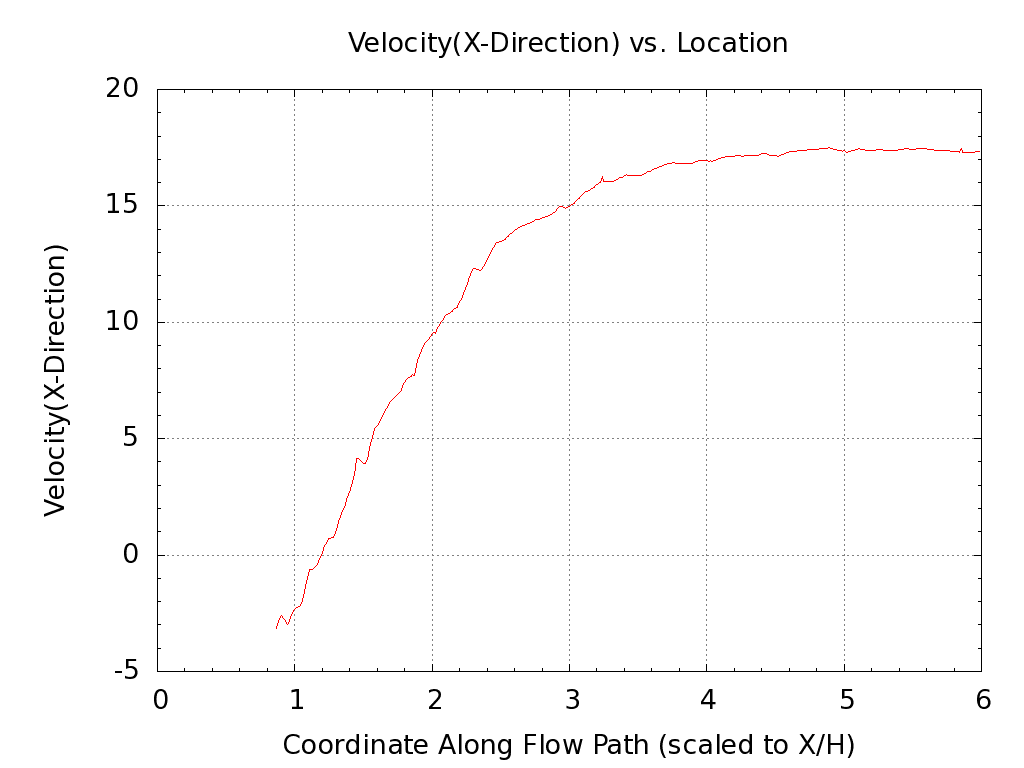

Supplement: S2 Images Folder — The numbers for each folder corresponds to the user number in phase2reports.txt. The Virtual Wind Tunnel does not generate images unless a user asks for a particular image. On a few occasions, users did not even look at certain graphs, so those graphs were not generated. Such non-inspected graphs are not present here. (Note: the x-axis label in the wake stream velocity graph, in Phase 2, due to a typo, indicated a scaling factor that was not actually applied.) (ZIP) [file pone.0134978.s010.zip › S2_imagesfolder/39/wakeCenter.UMean.X.png]

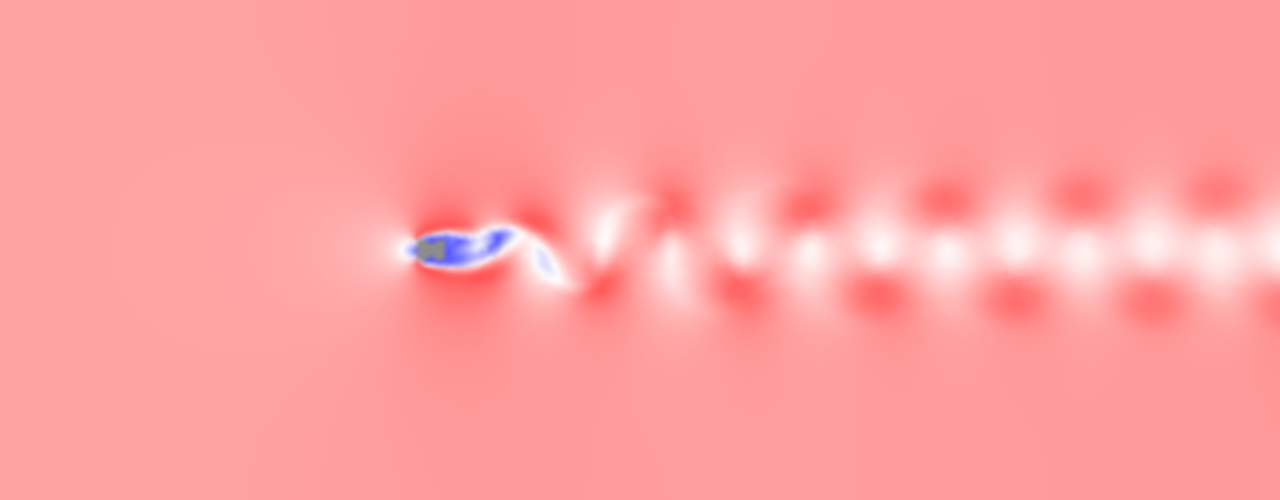

Supplement: S2 Images Folder — The numbers for each folder corresponds to the user number in phase2reports.txt. The Virtual Wind Tunnel does not generate images unless a user asks for a particular image. On a few occasions, users did not even look at certain graphs, so those graphs were not generated. Such non-inspected graphs are not present here. (Note: the x-axis label in the wake stream velocity graph, in Phase 2, due to a typo, indicated a scaling factor that was not actually applied.) (ZIP) [file pone.0134978.s010.zip › S2_imagesfolder/40/U.png]

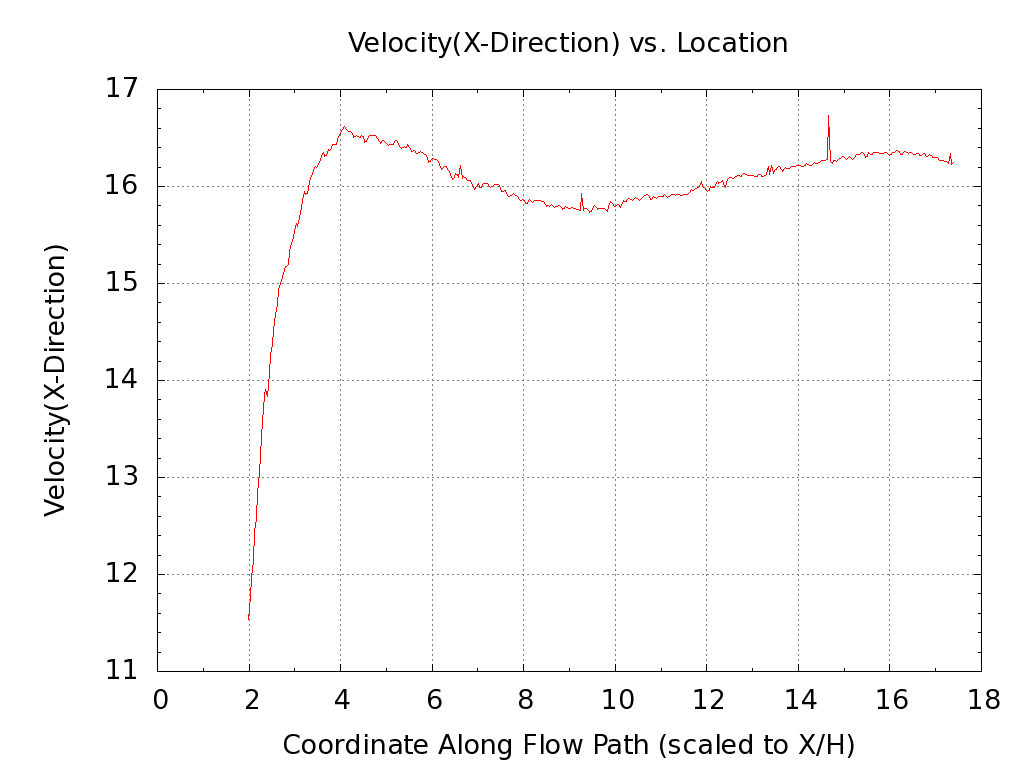

Supplement: S2 Images Folder — The numbers for each folder corresponds to the user number in phase2reports.txt. The Virtual Wind Tunnel does not generate images unless a user asks for a particular image. On a few occasions, users did not even look at certain graphs, so those graphs were not generated. Such non-inspected graphs are not present here. (Note: the x-axis label in the wake stream velocity graph, in Phase 2, due to a typo, indicated a scaling factor that was not actually applied.) (ZIP) [file pone.0134978.s010.zip › S2_imagesfolder/40/wakeCenter.UMean.X.png]

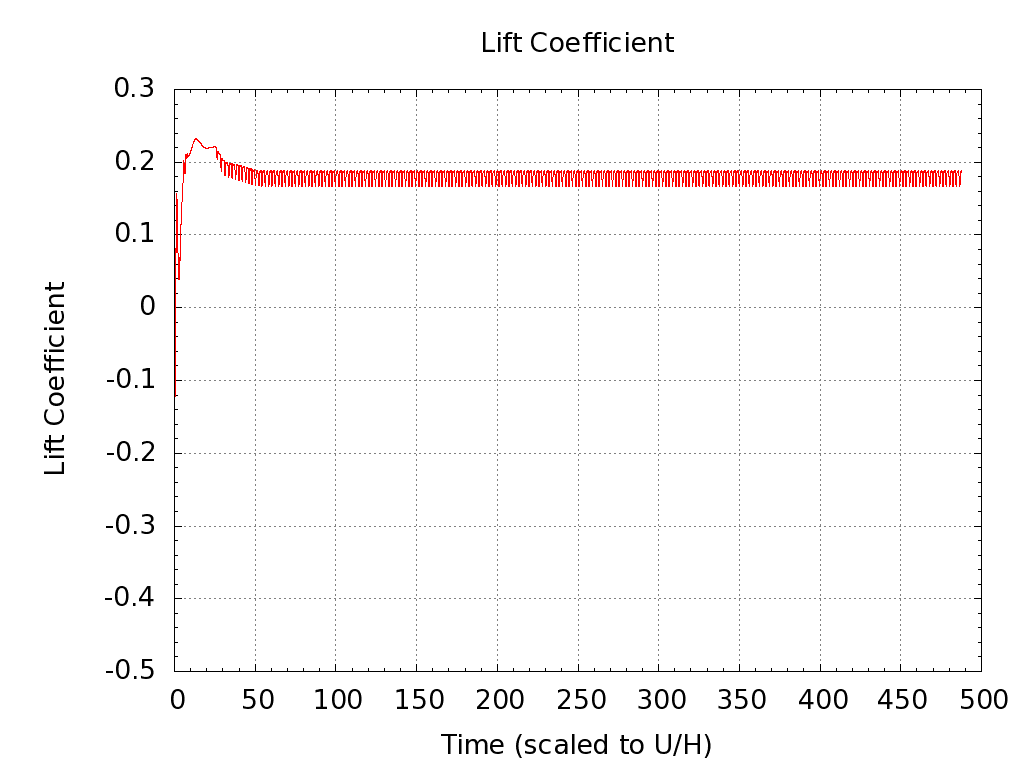

Supplement: S2 Images Folder — The numbers for each folder corresponds to the user number in phase2reports.txt. The Virtual Wind Tunnel does not generate images unless a user asks for a particular image. On a few occasions, users did not even look at certain graphs, so those graphs were not generated. Such non-inspected graphs are not present here. (Note: the x-axis label in the wake stream velocity graph, in Phase 2, due to a typo, indicated a scaling factor that was not actually applied.) (ZIP) [file pone.0134978.s010.zip › S2_imagesfolder/41/forceCoeffs.Cl.png]

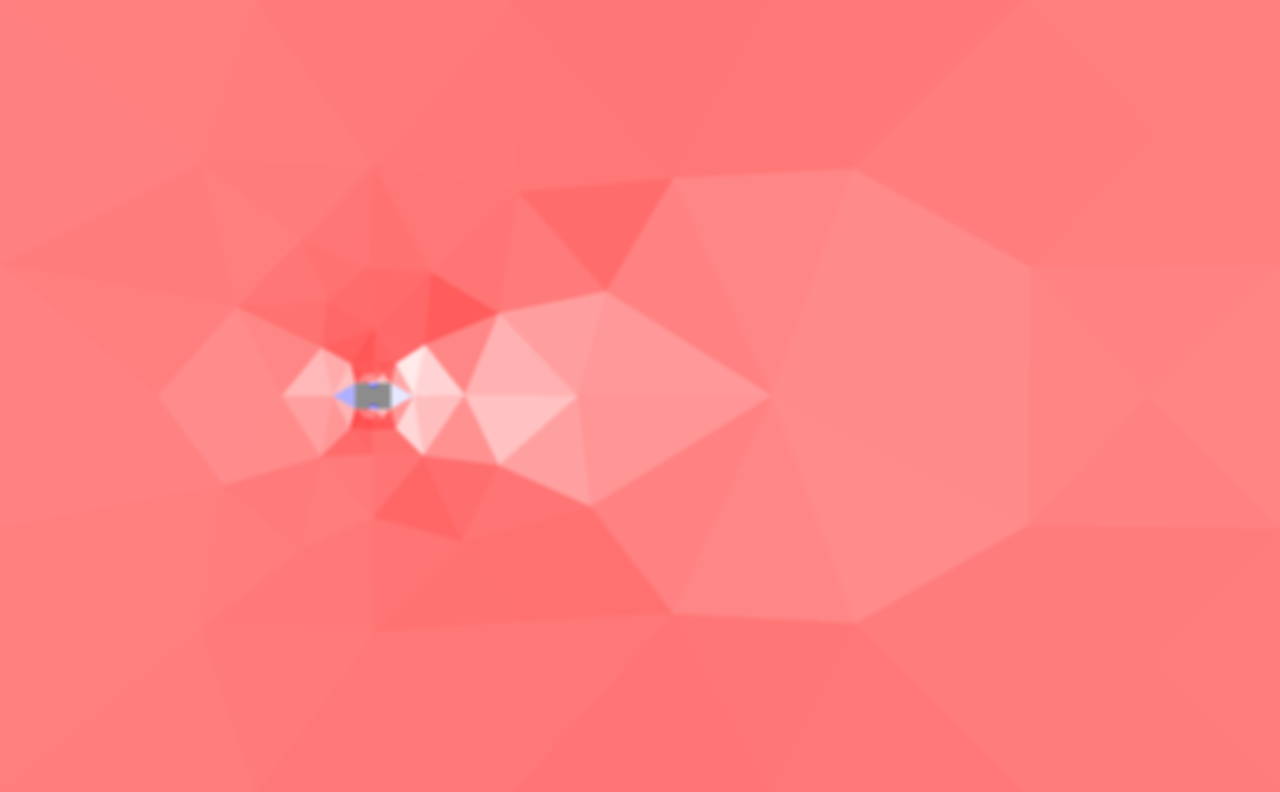

Supplement: S2 Images Folder — The numbers for each folder corresponds to the user number in phase2reports.txt. The Virtual Wind Tunnel does not generate images unless a user asks for a particular image. On a few occasions, users did not even look at certain graphs, so those graphs were not generated. Such non-inspected graphs are not present here. (Note: the x-axis label in the wake stream velocity graph, in Phase 2, due to a typo, indicated a scaling factor that was not actually applied.) (ZIP) [file pone.0134978.s010.zip › S2_imagesfolder/41/U.png]

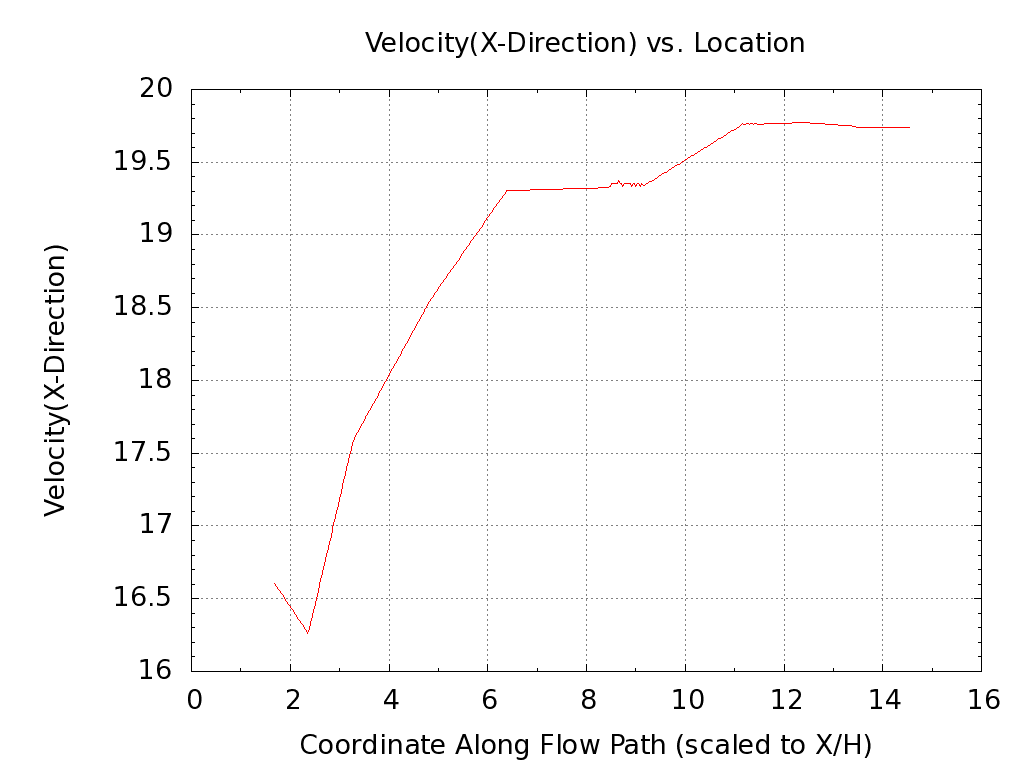

Supplement: S2 Images Folder — The numbers for each folder corresponds to the user number in phase2reports.txt. The Virtual Wind Tunnel does not generate images unless a user asks for a particular image. On a few occasions, users did not even look at certain graphs, so those graphs were not generated. Such non-inspected graphs are not present here. (Note: the x-axis label in the wake stream velocity graph, in Phase 2, due to a typo, indicated a scaling factor that was not actually applied.) (ZIP) [file pone.0134978.s010.zip › S2_imagesfolder/41/wakeCenter.UMean.X.png]

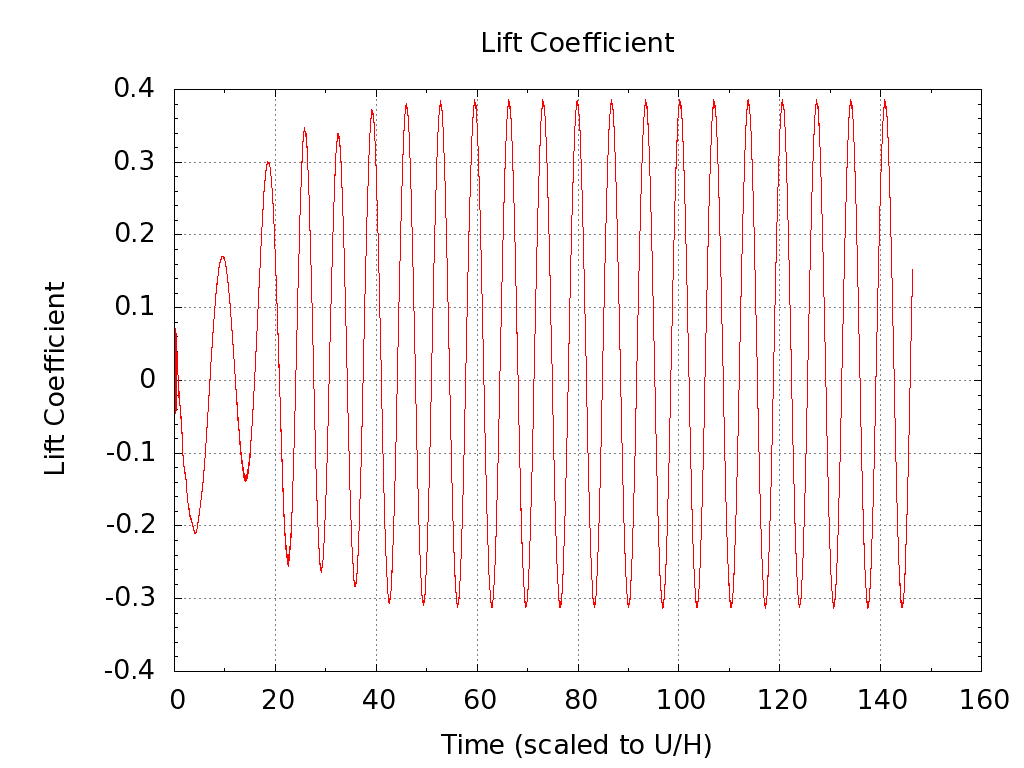

Supplement: S2 Images Folder — The numbers for each folder corresponds to the user number in phase2reports.txt. The Virtual Wind Tunnel does not generate images unless a user asks for a particular image. On a few occasions, users did not even look at certain graphs, so those graphs were not generated. Such non-inspected graphs are not present here. (Note: the x-axis label in the wake stream velocity graph, in Phase 2, due to a typo, indicated a scaling factor that was not actually applied.) (ZIP) [file pone.0134978.s010.zip › S2_imagesfolder/43/forceCoeffs.Cl.png]

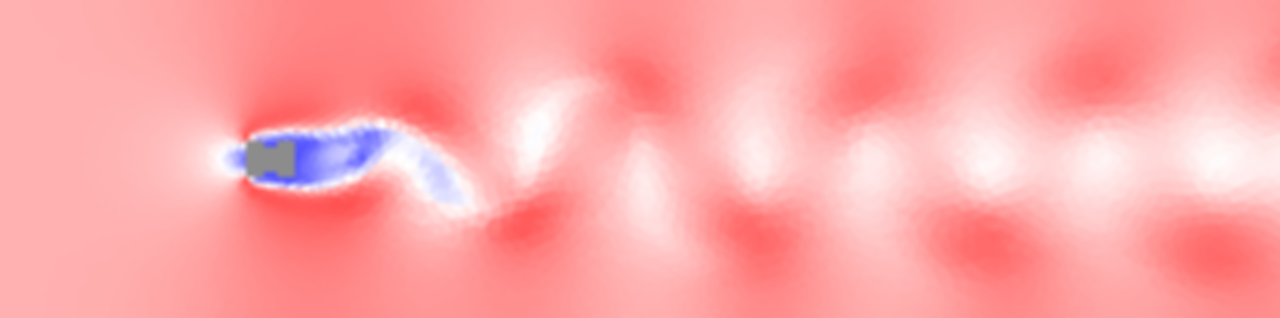

Supplement: S2 Images Folder — The numbers for each folder corresponds to the user number in phase2reports.txt. The Virtual Wind Tunnel does not generate images unless a user asks for a particular image. On a few occasions, users did not even look at certain graphs, so those graphs were not generated. Such non-inspected graphs are not present here. (Note: the x-axis label in the wake stream velocity graph, in Phase 2, due to a typo, indicated a scaling factor that was not actually applied.) (ZIP) [file pone.0134978.s010.zip › S2_imagesfolder/43/U.png]

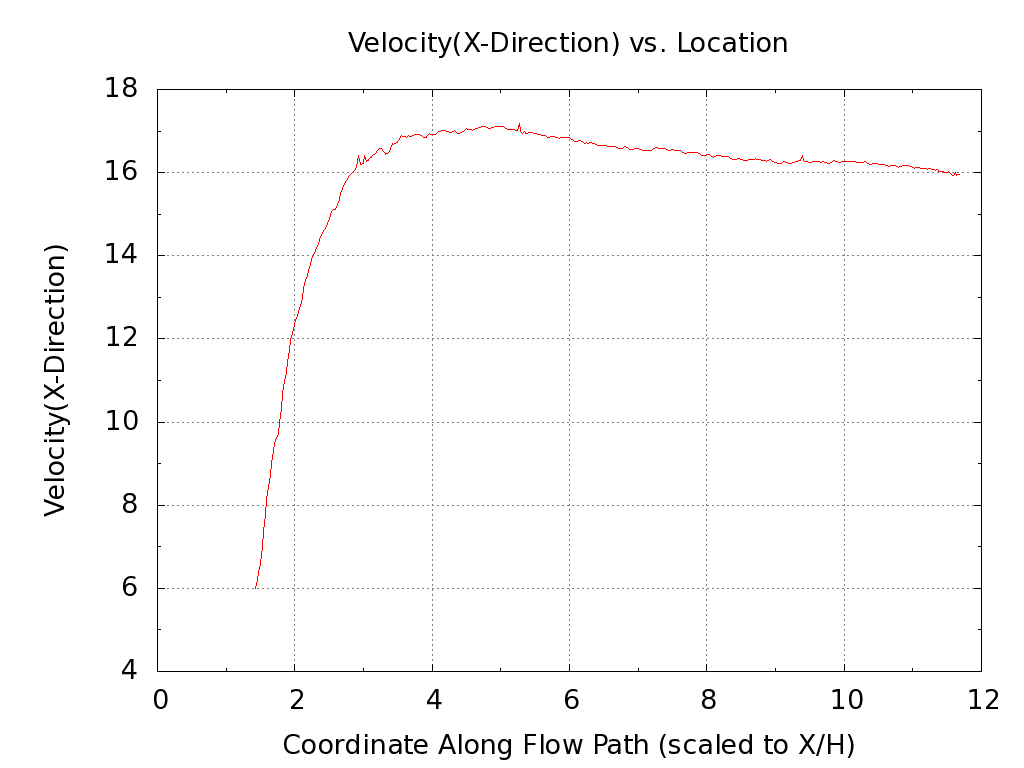

Supplement: S2 Images Folder — The numbers for each folder corresponds to the user number in phase2reports.txt. The Virtual Wind Tunnel does not generate images unless a user asks for a particular image. On a few occasions, users did not even look at certain graphs, so those graphs were not generated. Such non-inspected graphs are not present here. (Note: the x-axis label in the wake stream velocity graph, in Phase 2, due to a typo, indicated a scaling factor that was not actually applied.) (ZIP) [file pone.0134978.s010.zip › S2_imagesfolder/43/wakeCenter.UMean.X.png]

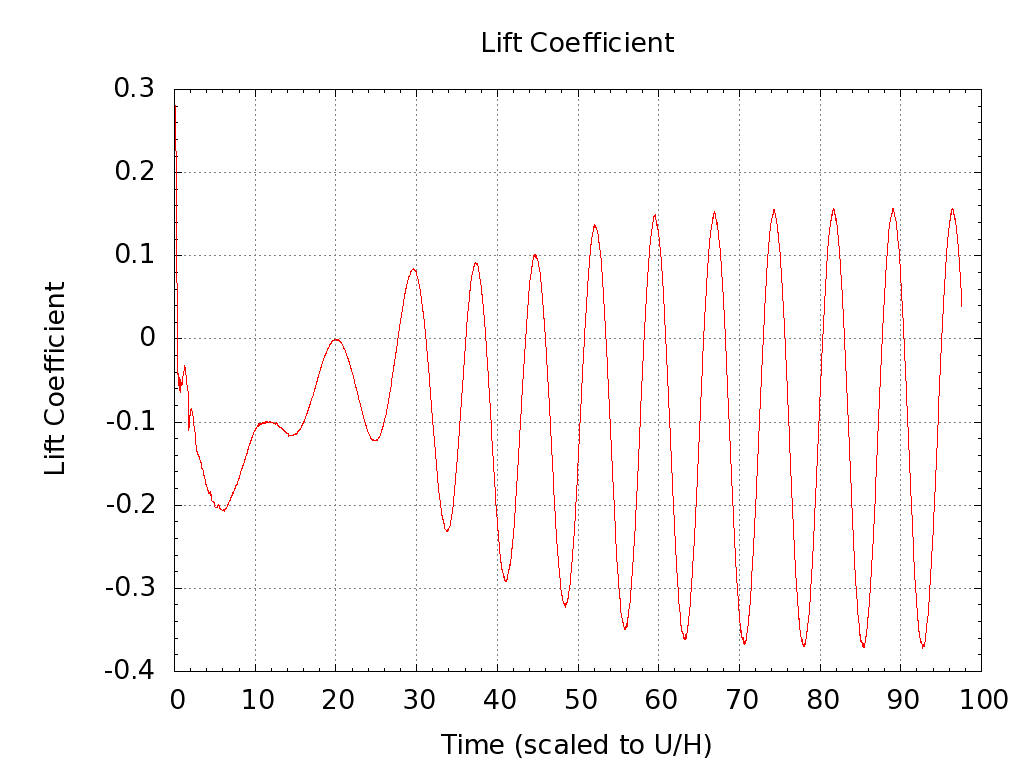

Supplement: S2 Images Folder — The numbers for each folder corresponds to the user number in phase2reports.txt. The Virtual Wind Tunnel does not generate images unless a user asks for a particular image. On a few occasions, users did not even look at certain graphs, so those graphs were not generated. Such non-inspected graphs are not present here. (Note: the x-axis label in the wake stream velocity graph, in Phase 2, due to a typo, indicated a scaling factor that was not actually applied.) (ZIP) [file pone.0134978.s010.zip › S2_imagesfolder/47/forceCoeffs.Cl.png]

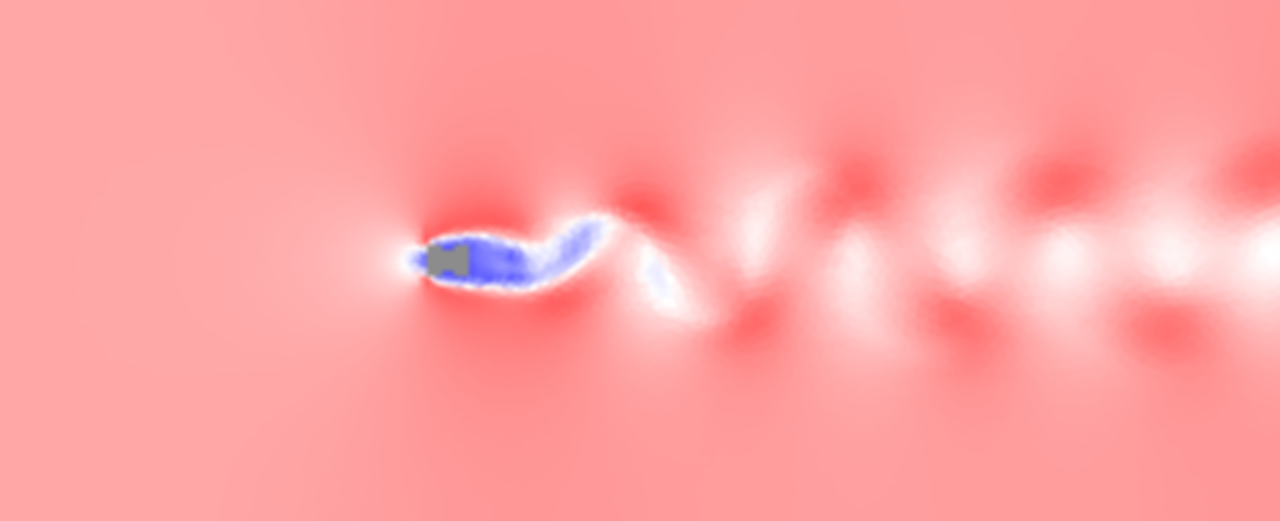

Supplement: S2 Images Folder — The numbers for each folder corresponds to the user number in phase2reports.txt. The Virtual Wind Tunnel does not generate images unless a user asks for a particular image. On a few occasions, users did not even look at certain graphs, so those graphs were not generated. Such non-inspected graphs are not present here. (Note: the x-axis label in the wake stream velocity graph, in Phase 2, due to a typo, indicated a scaling factor that was not actually applied.) (ZIP) [file pone.0134978.s010.zip › S2_imagesfolder/47/U.png]

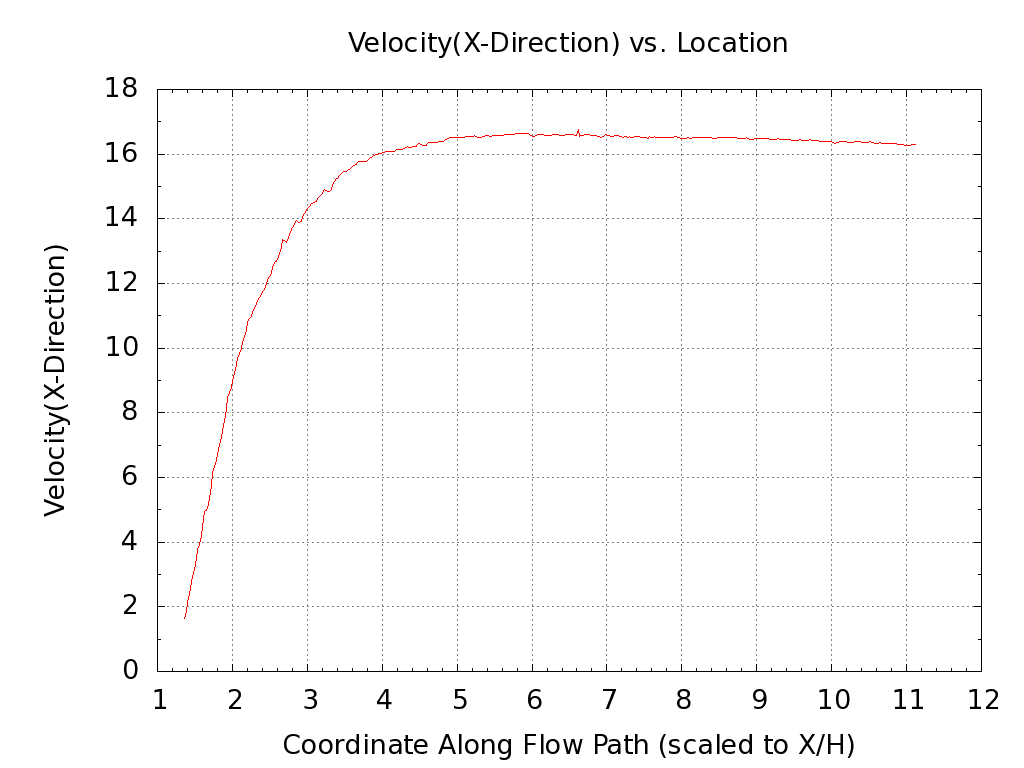

Supplement: S2 Images Folder — The numbers for each folder corresponds to the user number in phase2reports.txt. The Virtual Wind Tunnel does not generate images unless a user asks for a particular image. On a few occasions, users did not even look at certain graphs, so those graphs were not generated. Such non-inspected graphs are not present here. (Note: the x-axis label in the wake stream velocity graph, in Phase 2, due to a typo, indicated a scaling factor that was not actually applied.) (ZIP) [file pone.0134978.s010.zip › S2_imagesfolder/47/wakeCenter.UMean.X.png]

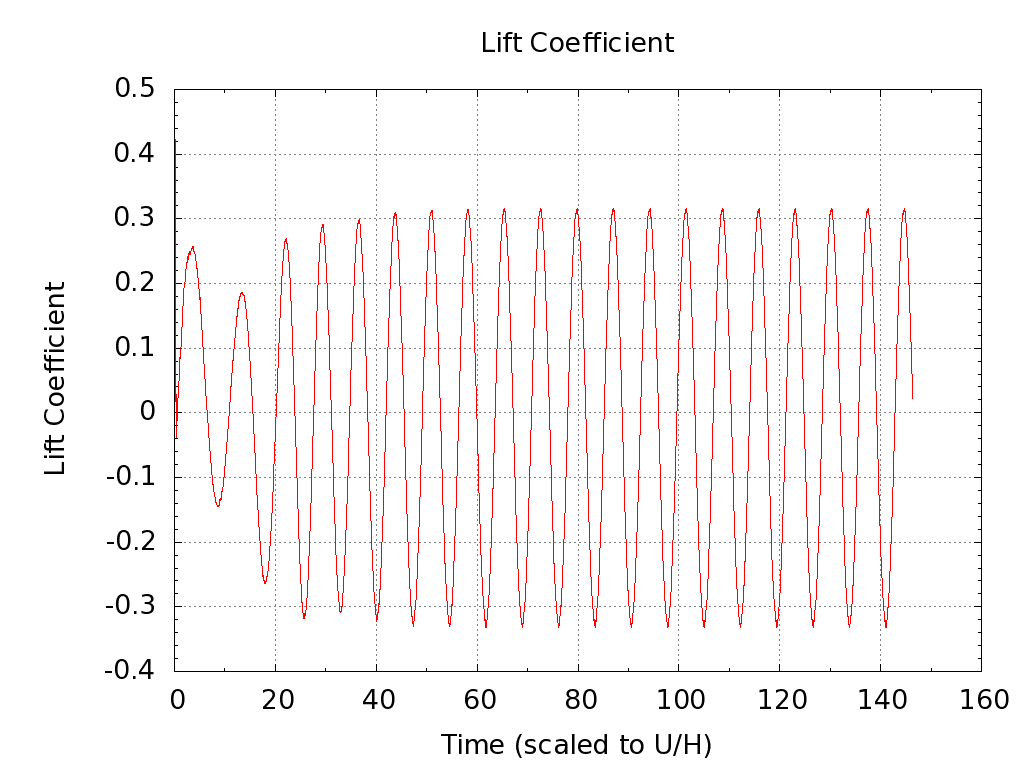

Supplement: S2 Images Folder — The numbers for each folder corresponds to the user number in phase2reports.txt. The Virtual Wind Tunnel does not generate images unless a user asks for a particular image. On a few occasions, users did not even look at certain graphs, so those graphs were not generated. Such non-inspected graphs are not present here. (Note: the x-axis label in the wake stream velocity graph, in Phase 2, due to a typo, indicated a scaling factor that was not actually applied.) (ZIP) [file pone.0134978.s010.zip › S2_imagesfolder/48/forceCoeffs.Cl.png]

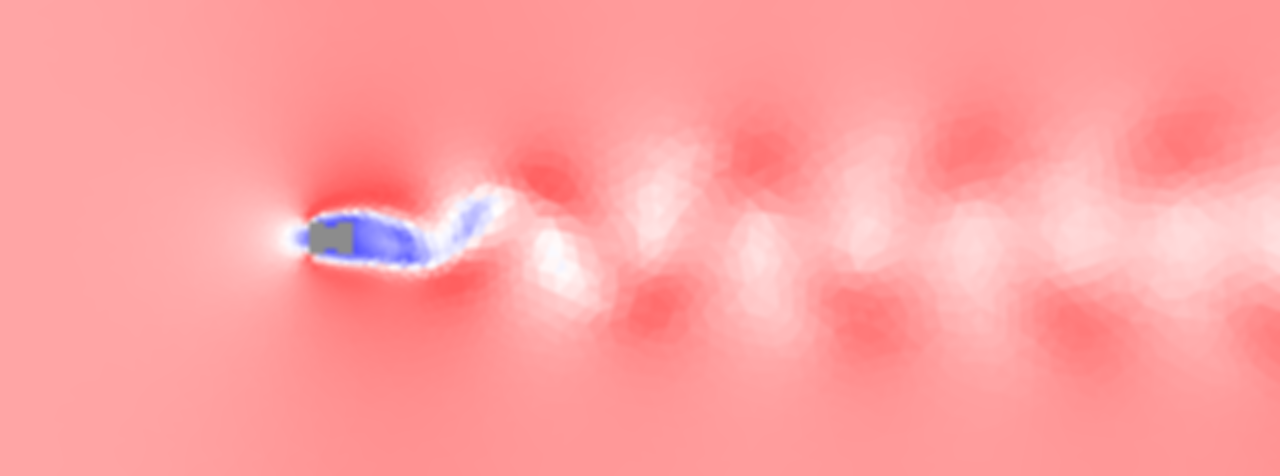

Supplement: S2 Images Folder — The numbers for each folder corresponds to the user number in phase2reports.txt. The Virtual Wind Tunnel does not generate images unless a user asks for a particular image. On a few occasions, users did not even look at certain graphs, so those graphs were not generated. Such non-inspected graphs are not present here. (Note: the x-axis label in the wake stream velocity graph, in Phase 2, due to a typo, indicated a scaling factor that was not actually applied.) (ZIP) [file pone.0134978.s010.zip › S2_imagesfolder/48/U.png]

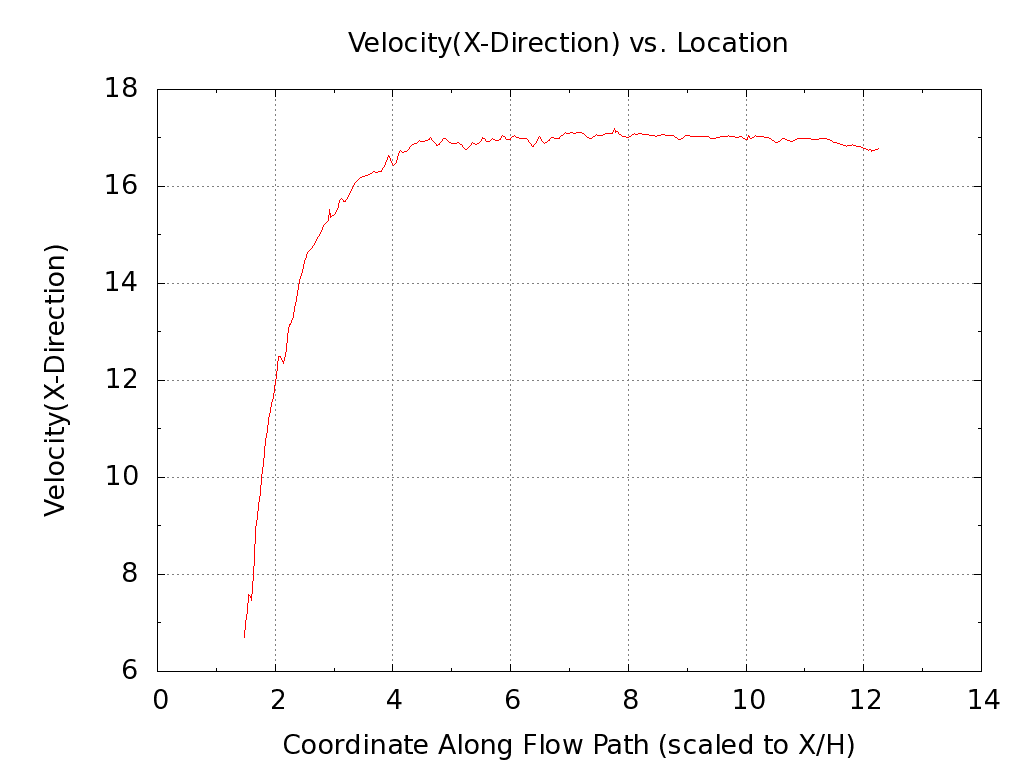

Supplement: S2 Images Folder — The numbers for each folder corresponds to the user number in phase2reports.txt. The Virtual Wind Tunnel does not generate images unless a user asks for a particular image. On a few occasions, users did not even look at certain graphs, so those graphs were not generated. Such non-inspected graphs are not present here. (Note: the x-axis label in the wake stream velocity graph, in Phase 2, due to a typo, indicated a scaling factor that was not actually applied.) (ZIP) [file pone.0134978.s010.zip › S2_imagesfolder/48/wakeCenter.UMean.X.png]

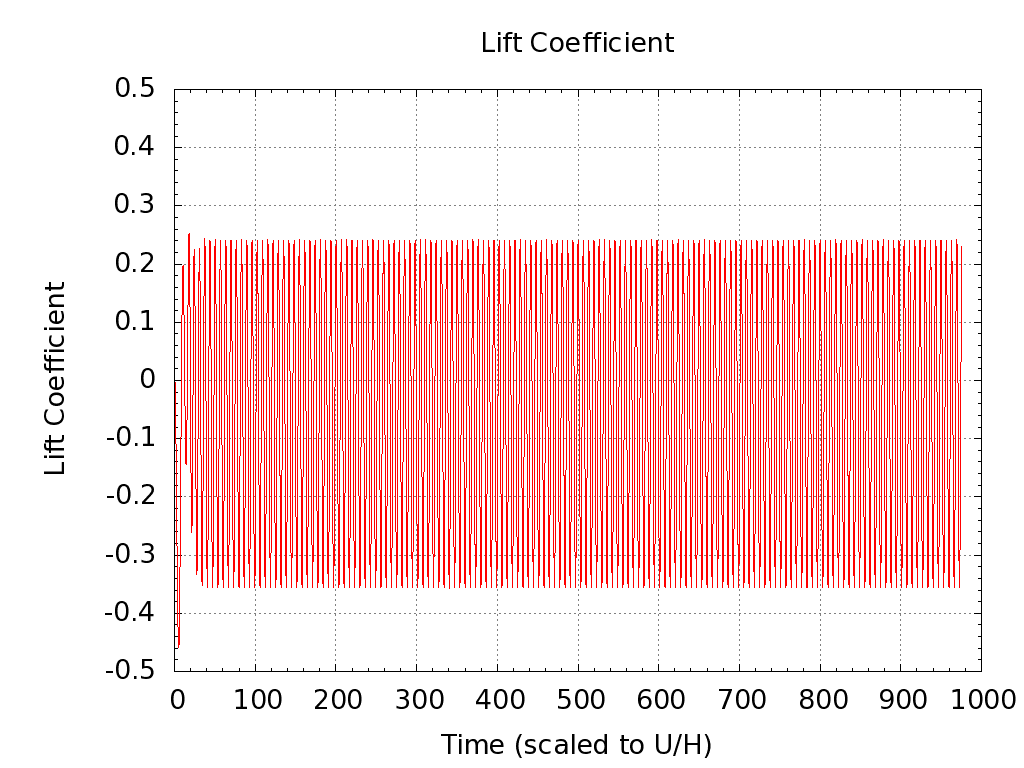

Supplement: S2 Images Folder — The numbers for each folder corresponds to the user number in phase2reports.txt. The Virtual Wind Tunnel does not generate images unless a user asks for a particular image. On a few occasions, users did not even look at certain graphs, so those graphs were not generated. Such non-inspected graphs are not present here. (Note: the x-axis label in the wake stream velocity graph, in Phase 2, due to a typo, indicated a scaling factor that was not actually applied.) (ZIP) [file pone.0134978.s010.zip › S2_imagesfolder/51/forceCoeffs.Cl.png]

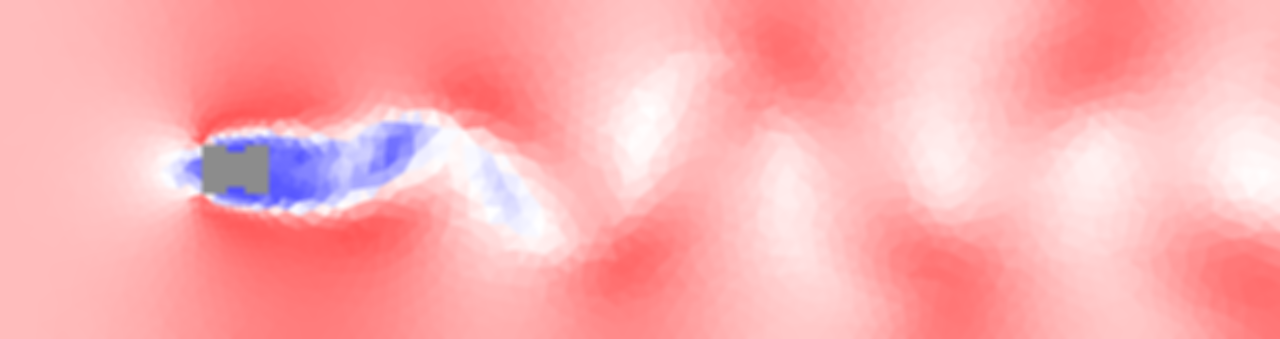

Supplement: S2 Images Folder — The numbers for each folder corresponds to the user number in phase2reports.txt. The Virtual Wind Tunnel does not generate images unless a user asks for a particular image. On a few occasions, users did not even look at certain graphs, so those graphs were not generated. Such non-inspected graphs are not present here. (Note: the x-axis label in the wake stream velocity graph, in Phase 2, due to a typo, indicated a scaling factor that was not actually applied.) (ZIP) [file pone.0134978.s010.zip › S2_imagesfolder/51/U.png]

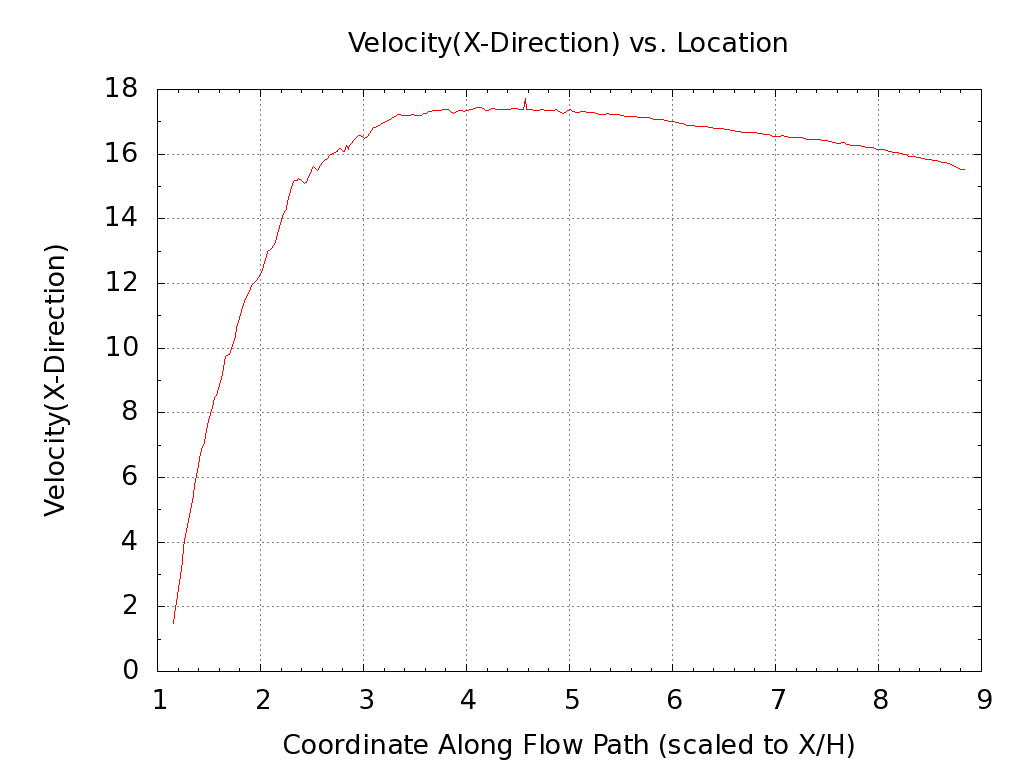

Supplement: S2 Images Folder — The numbers for each folder corresponds to the user number in phase2reports.txt. The Virtual Wind Tunnel does not generate images unless a user asks for a particular image. On a few occasions, users did not even look at certain graphs, so those graphs were not generated. Such non-inspected graphs are not present here. (Note: the x-axis label in the wake stream velocity graph, in Phase 2, due to a typo, indicated a scaling factor that was not actually applied.) (ZIP) [file pone.0134978.s010.zip › S2_imagesfolder/51/wakeCenter.UMean.X.png]

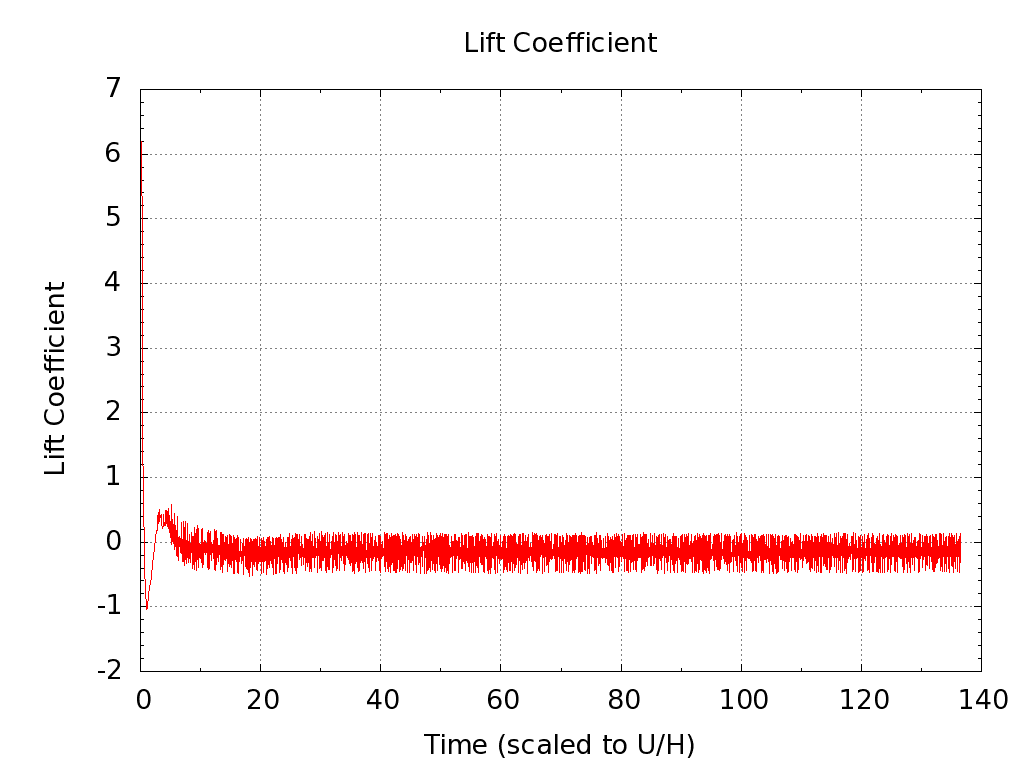

Supplement: S2 Images Folder — The numbers for each folder corresponds to the user number in phase2reports.txt. The Virtual Wind Tunnel does not generate images unless a user asks for a particular image. On a few occasions, users did not even look at certain graphs, so those graphs were not generated. Such non-inspected graphs are not present here. (Note: the x-axis label in the wake stream velocity graph, in Phase 2, due to a typo, indicated a scaling factor that was not actually applied.) (ZIP) [file pone.0134978.s010.zip › S2_imagesfolder/52/forceCoeffs.Cl.png]

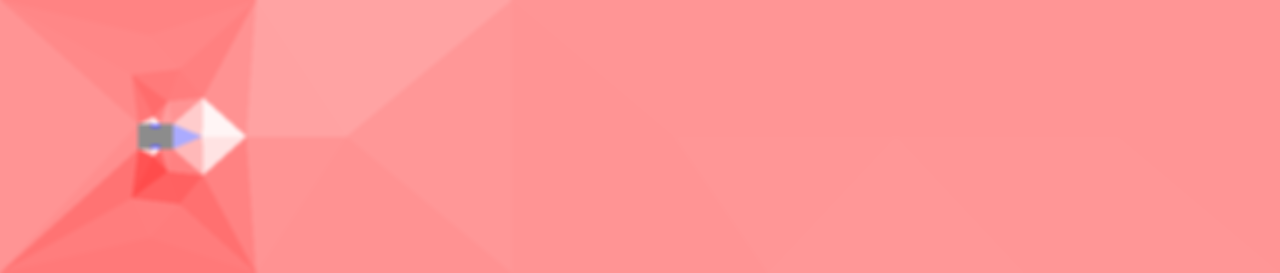

Supplement: S2 Images Folder — The numbers for each folder corresponds to the user number in phase2reports.txt. The Virtual Wind Tunnel does not generate images unless a user asks for a particular image. On a few occasions, users did not even look at certain graphs, so those graphs were not generated. Such non-inspected graphs are not present here. (Note: the x-axis label in the wake stream velocity graph, in Phase 2, due to a typo, indicated a scaling factor that was not actually applied.) (ZIP) [file pone.0134978.s010.zip › S2_imagesfolder/52/U.png]

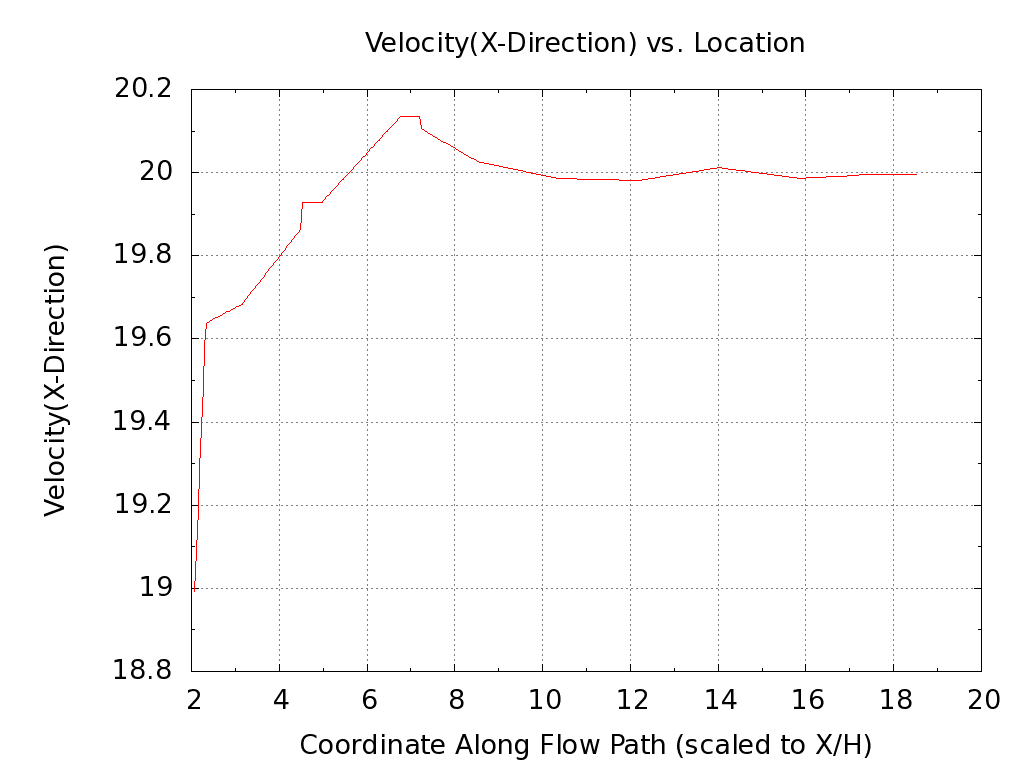

Supplement: S2 Images Folder — The numbers for each folder corresponds to the user number in phase2reports.txt. The Virtual Wind Tunnel does not generate images unless a user asks for a particular image. On a few occasions, users did not even look at certain graphs, so those graphs were not generated. Such non-inspected graphs are not present here. (Note: the x-axis label in the wake stream velocity graph, in Phase 2, due to a typo, indicated a scaling factor that was not actually applied.) (ZIP) [file pone.0134978.s010.zip › S2_imagesfolder/52/wakeCenter.UMean.X.png]

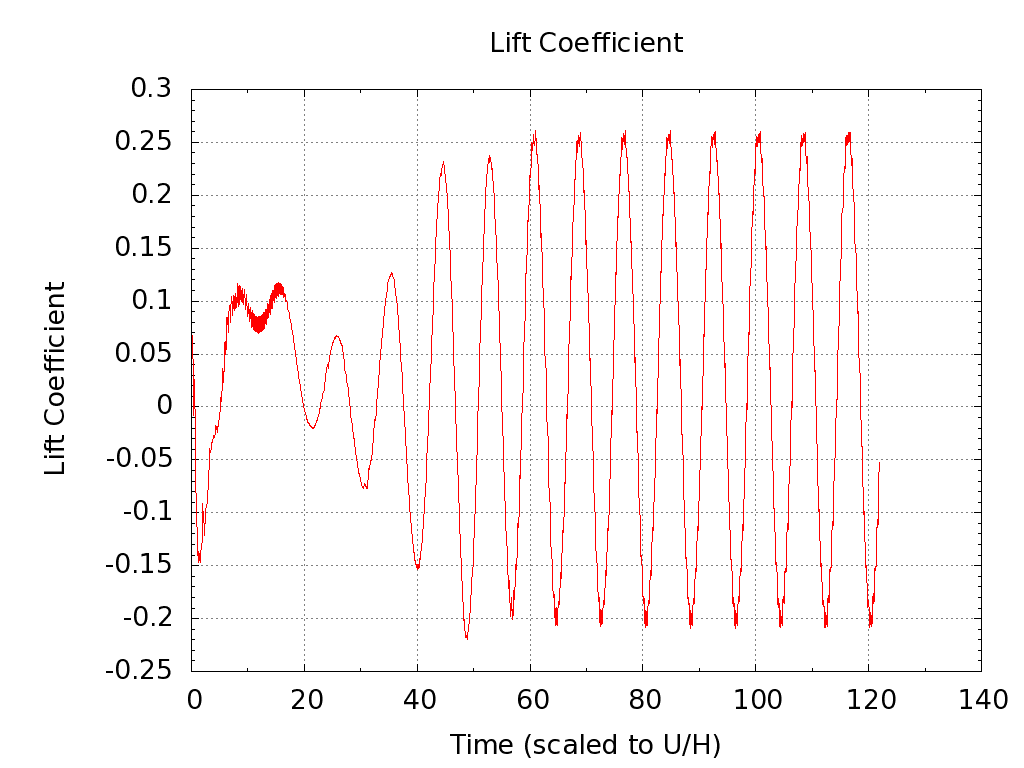

Supplement: S2 Images Folder — The numbers for each folder corresponds to the user number in phase2reports.txt. The Virtual Wind Tunnel does not generate images unless a user asks for a particular image. On a few occasions, users did not even look at certain graphs, so those graphs were not generated. Such non-inspected graphs are not present here. (Note: the x-axis label in the wake stream velocity graph, in Phase 2, due to a typo, indicated a scaling factor that was not actually applied.) (ZIP) [file pone.0134978.s010.zip › S2_imagesfolder/8/forceCoeffs.Cl.png]

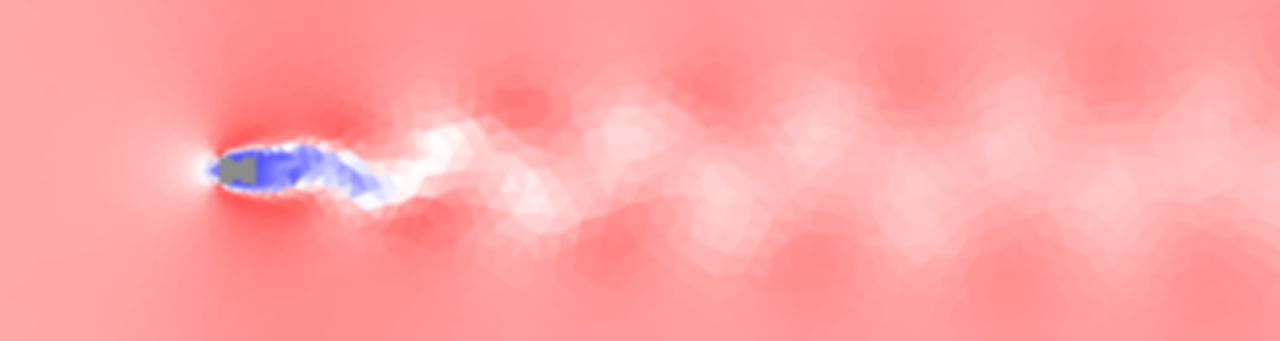

Supplement: S2 Images Folder — The numbers for each folder corresponds to the user number in phase2reports.txt. The Virtual Wind Tunnel does not generate images unless a user asks for a particular image. On a few occasions, users did not even look at certain graphs, so those graphs were not generated. Such non-inspected graphs are not present here. (Note: the x-axis label in the wake stream velocity graph, in Phase 2, due to a typo, indicated a scaling factor that was not actually applied.) (ZIP) [file pone.0134978.s010.zip › S2_imagesfolder/8/U.png]

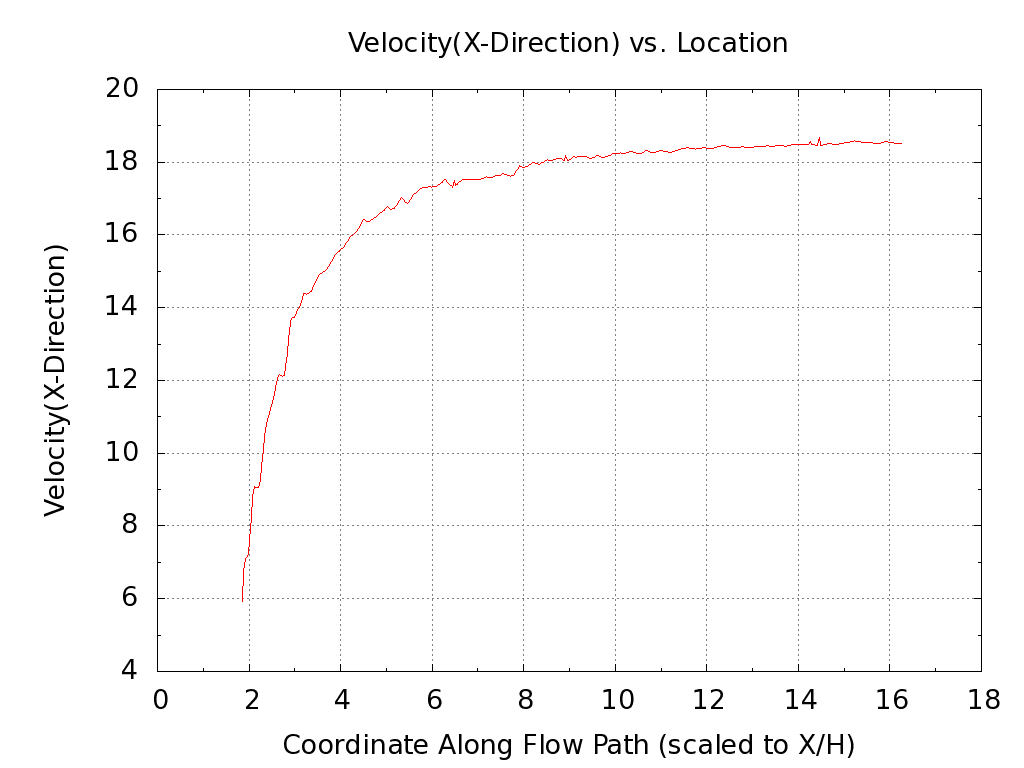

Supplement: S2 Images Folder — The numbers for each folder corresponds to the user number in phase2reports.txt. The Virtual Wind Tunnel does not generate images unless a user asks for a particular image. On a few occasions, users did not even look at certain graphs, so those graphs were not generated. Such non-inspected graphs are not present here. (Note: the x-axis label in the wake stream velocity graph, in Phase 2, due to a typo, indicated a scaling factor that was not actually applied.) (ZIP) [file pone.0134978.s010.zip › S2_imagesfolder/8/wakeCenter.UMean.X.png]

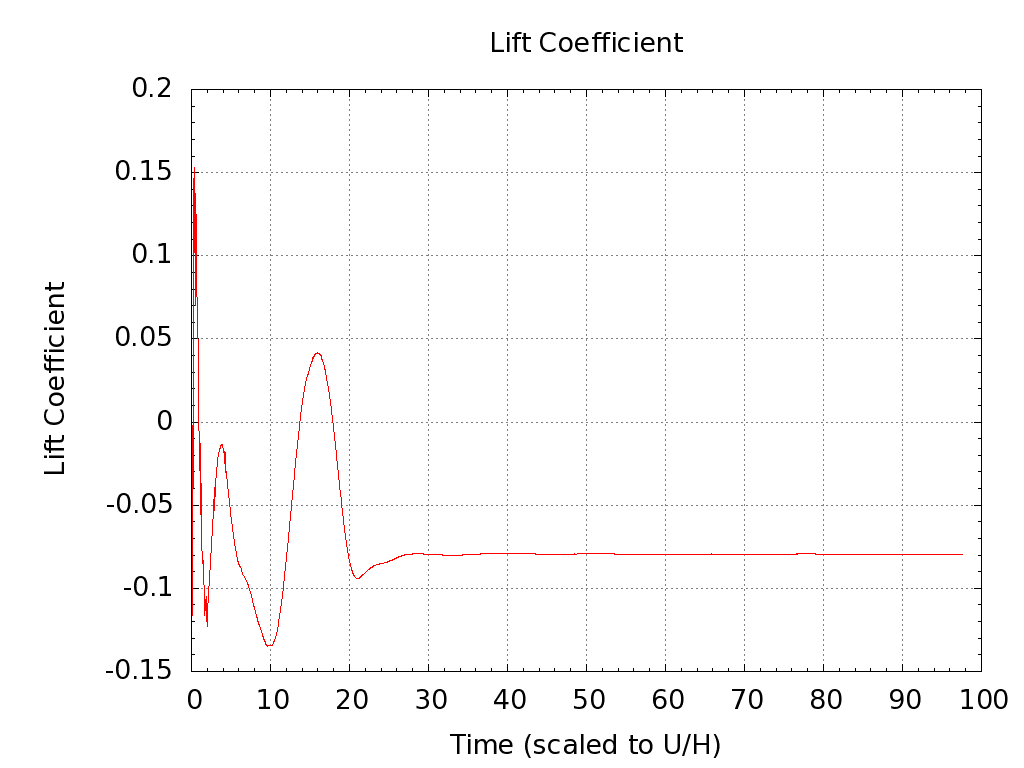

Supplement: S2 Images Folder — The numbers for each folder corresponds to the user number in phase2reports.txt. The Virtual Wind Tunnel does not generate images unless a user asks for a particular image. On a few occasions, users did not even look at certain graphs, so those graphs were not generated. Such non-inspected graphs are not present here. (Note: the x-axis label in the wake stream velocity graph, in Phase 2, due to a typo, indicated a scaling factor that was not actually applied.) (ZIP) [file pone.0134978.s010.zip › S2_imagesfolder/9/forceCoeffs.Cl.png]
