# Supplementary figures and images for: Lessons Learned from Crowdsourcing Complex Engineering Tasks (part 1 of 3)
Source: PLoS One. 2015 Sep 18;10(9):e0134978. doi: 10.1371/journal.pone.0134978 (PMC4575153; doi:10.1371/journal.pone.0134978)

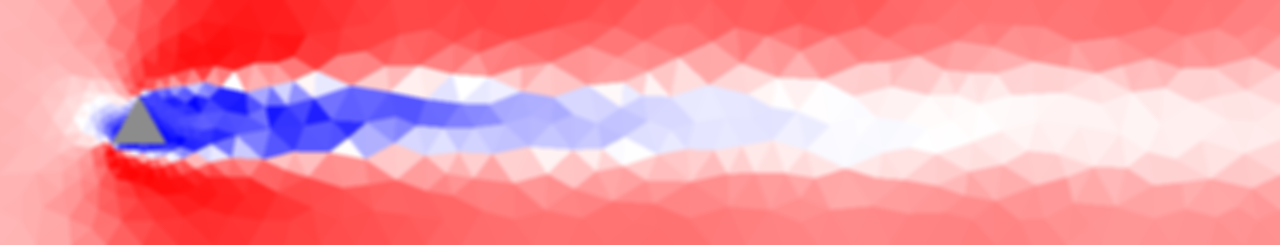

Supplement: S1 Images Folder — Image names are the column headings for and pertain to data in S1, S2, S4 and S5 Datasets. (ZIP) [file pone.0134978.s009.zip › S1_imagesfolder/AT1M1_B.png]

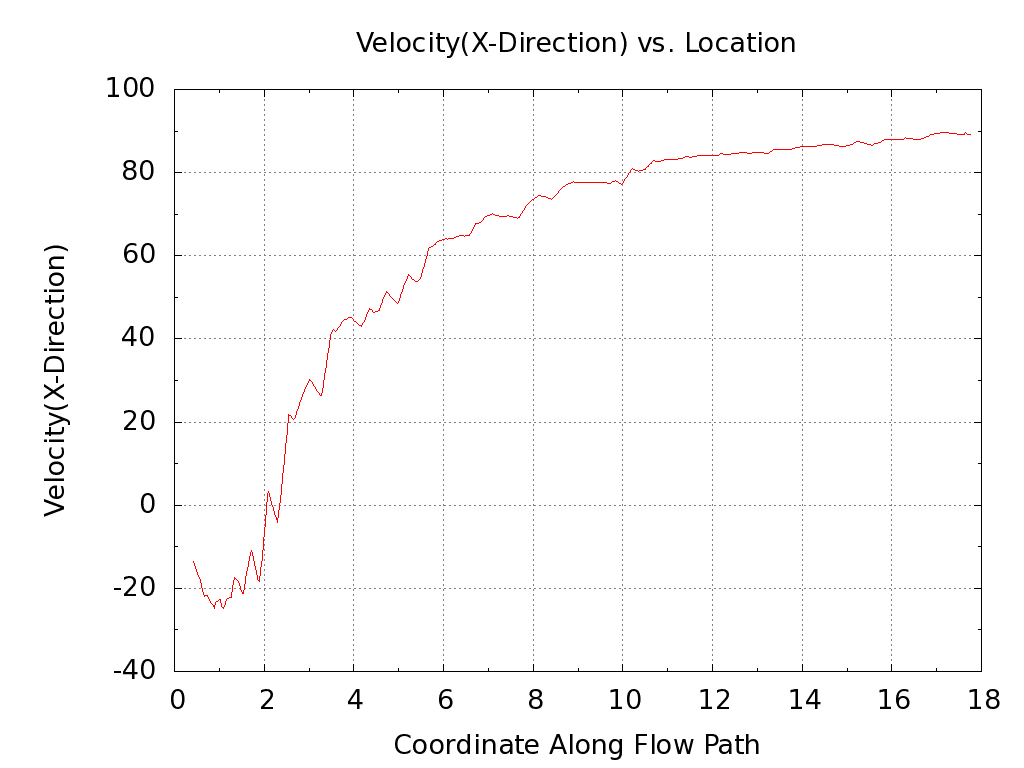

Supplement: S1 Images Folder — Image names are the column headings for and pertain to data in S1, S2, S4 and S5 Datasets. (ZIP) [file pone.0134978.s009.zip › S1_imagesfolder/AT1M1_C.png]

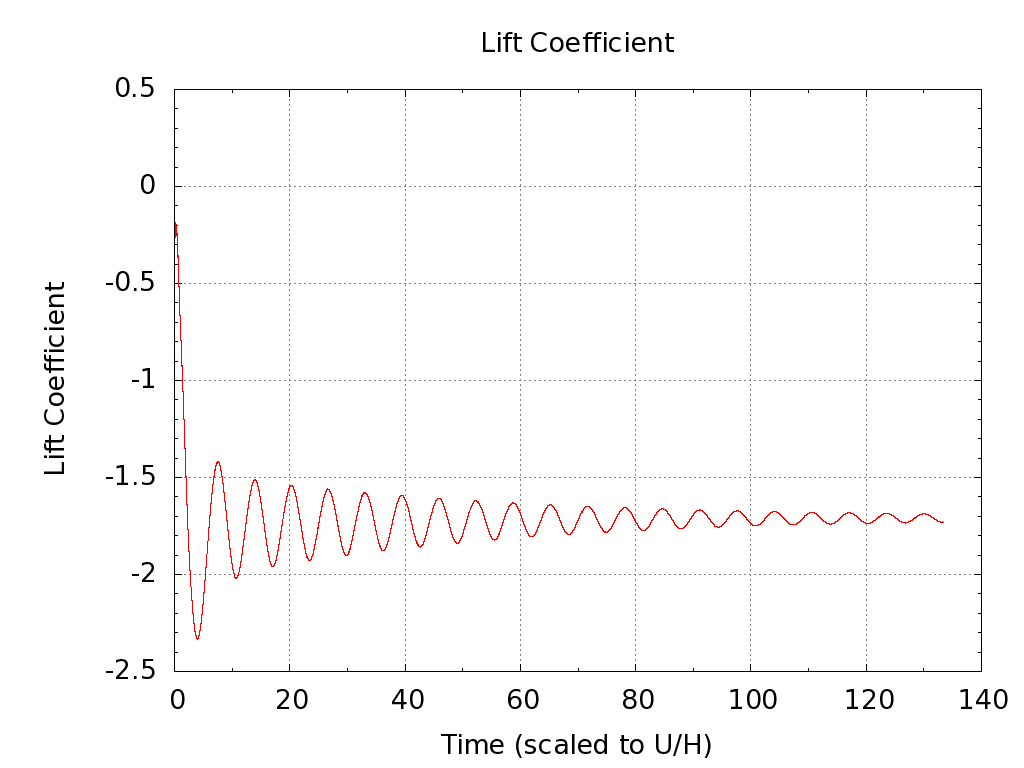

Supplement: S1 Images Folder — Image names are the column headings for and pertain to data in S1, S2, S4 and S5 Datasets. (ZIP) [file pone.0134978.s009.zip › S1_imagesfolder/AT1M1_D.png]

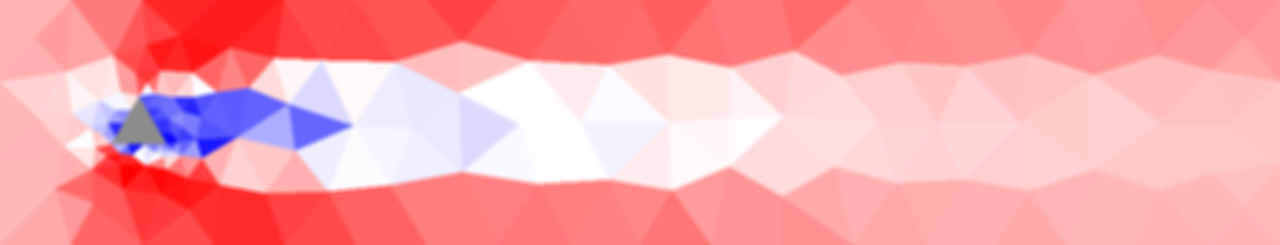

Supplement: S1 Images Folder — Image names are the column headings for and pertain to data in S1, S2, S4 and S5 Datasets. (ZIP) [file pone.0134978.s009.zip › S1_imagesfolder/AT1M2_B.png]

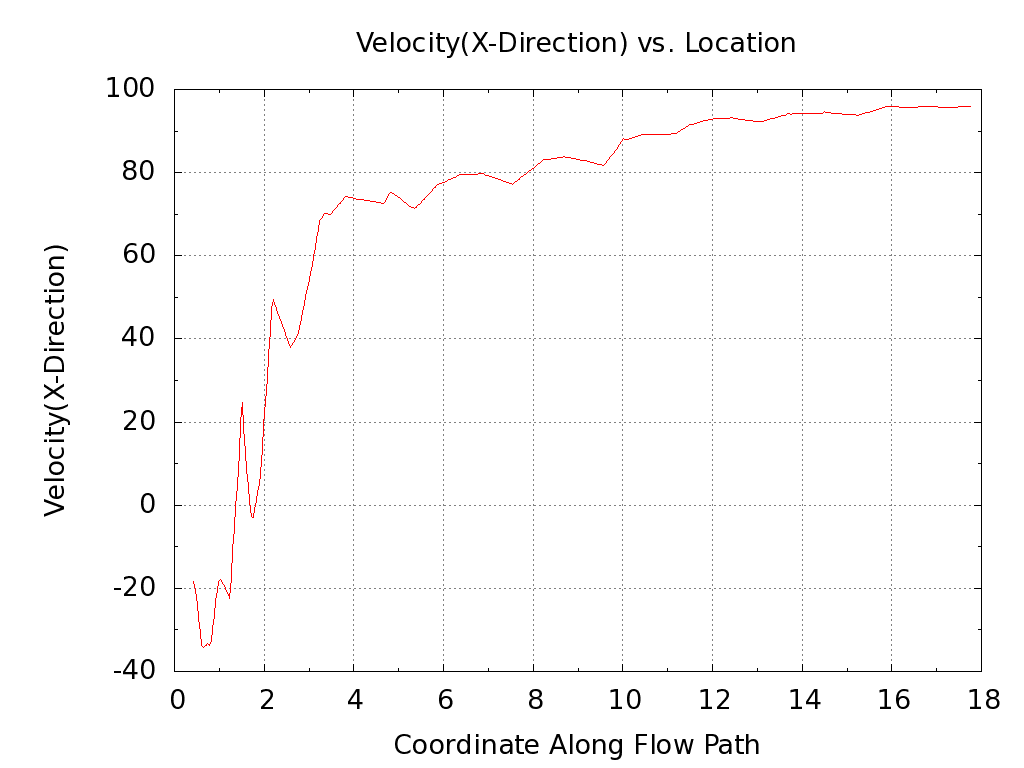

Supplement: S1 Images Folder — Image names are the column headings for and pertain to data in S1, S2, S4 and S5 Datasets. (ZIP) [file pone.0134978.s009.zip › S1_imagesfolder/AT1M2_C.png]

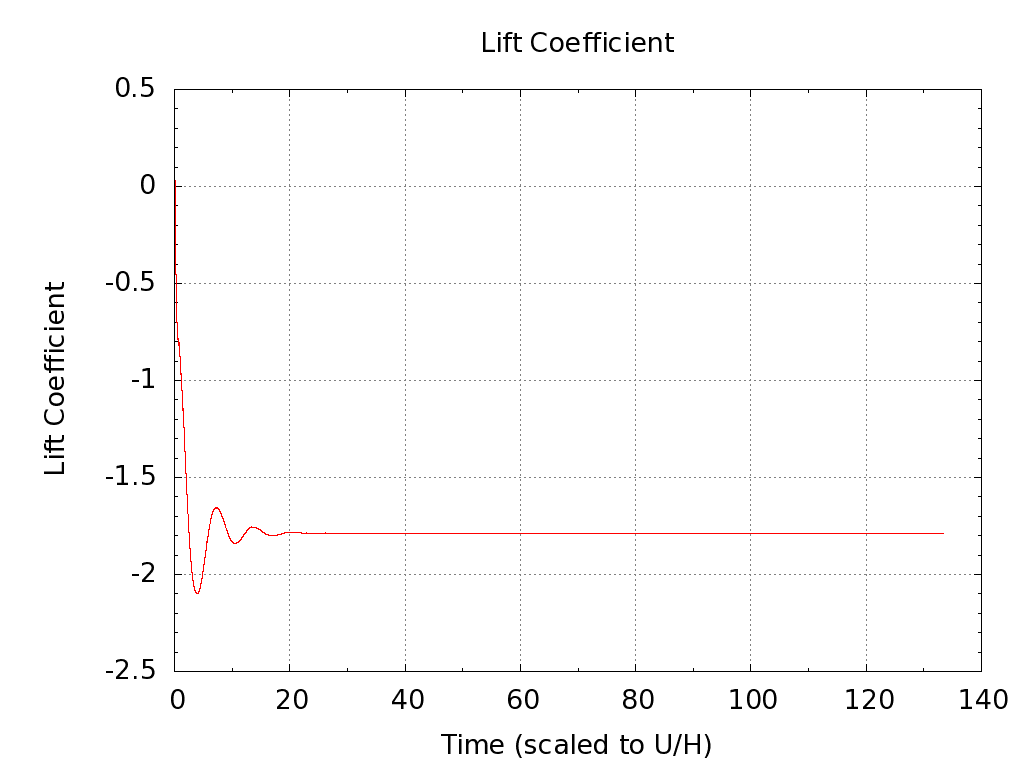

Supplement: S1 Images Folder — Image names are the column headings for and pertain to data in S1, S2, S4 and S5 Datasets. (ZIP) [file pone.0134978.s009.zip › S1_imagesfolder/AT1M2_D.png]

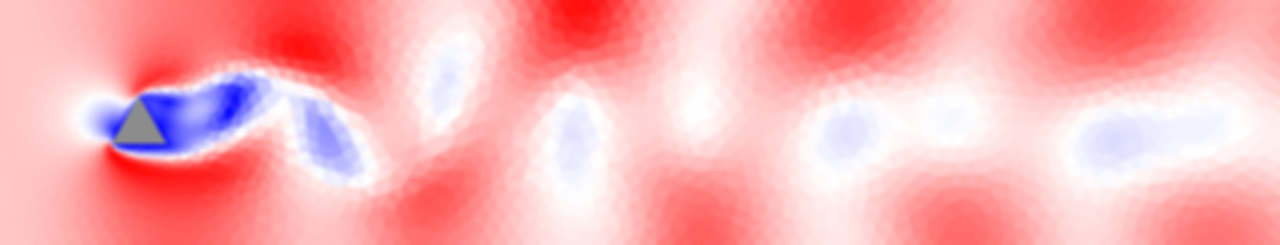

Supplement: S1 Images Folder — Image names are the column headings for and pertain to data in S1, S2, S4 and S5 Datasets. (ZIP) [file pone.0134978.s009.zip › S1_imagesfolder/AT1M3_B.png]

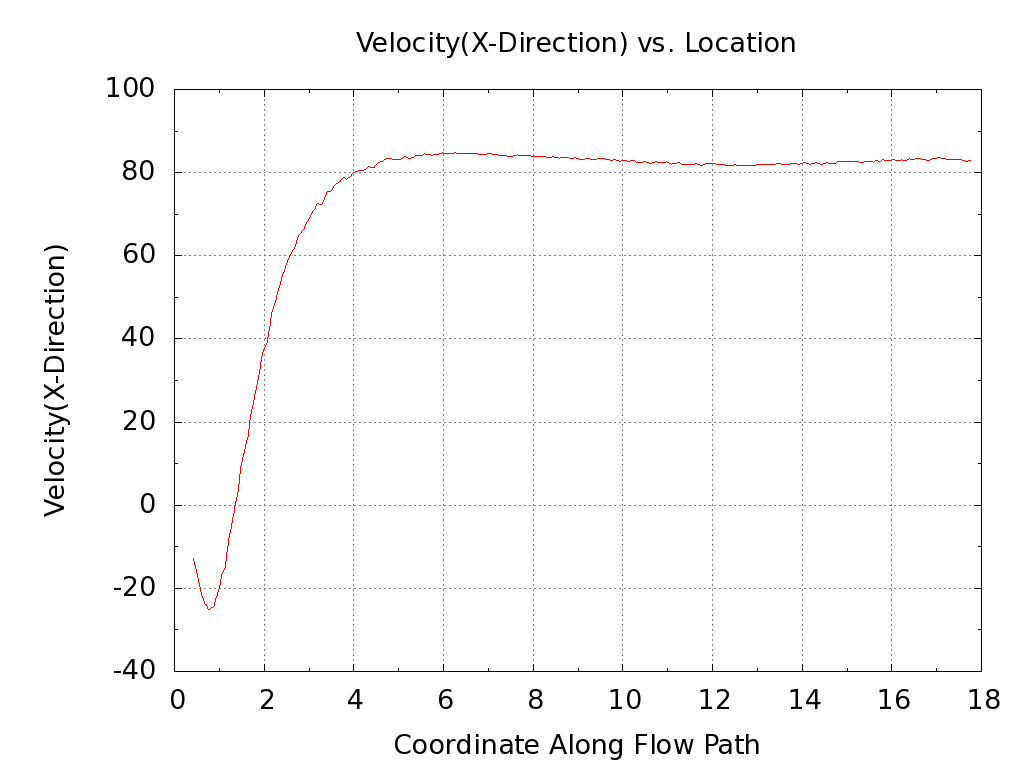

Supplement: S1 Images Folder — Image names are the column headings for and pertain to data in S1, S2, S4 and S5 Datasets. (ZIP) [file pone.0134978.s009.zip › S1_imagesfolder/AT1M3_C.png]

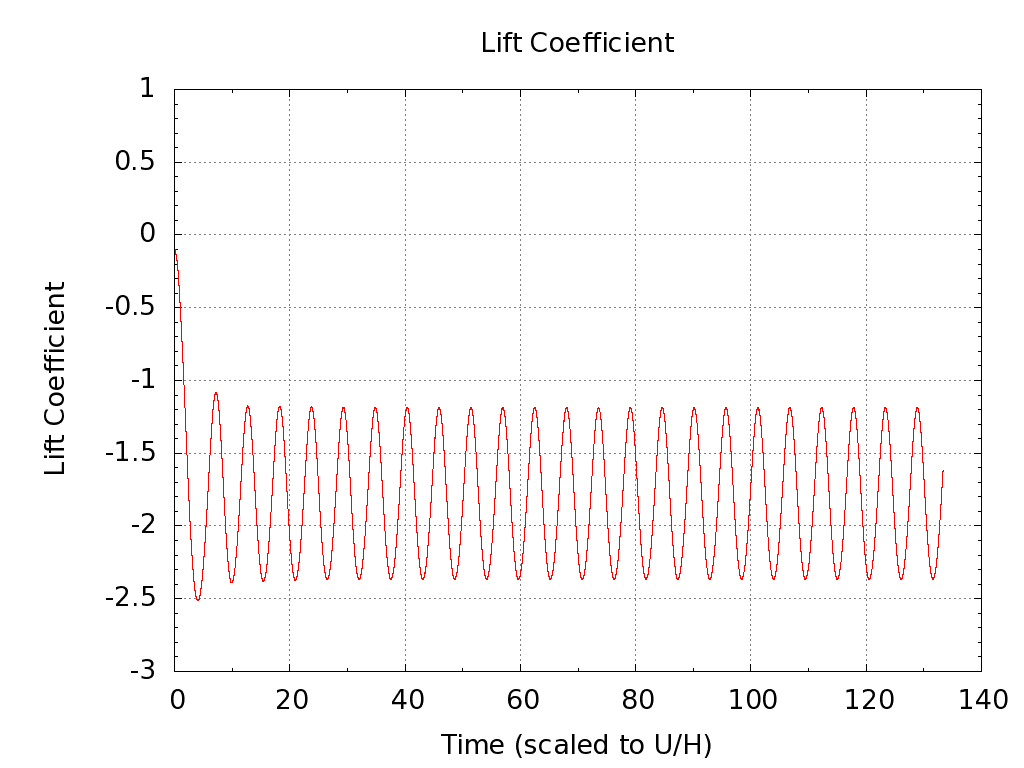

Supplement: S1 Images Folder — Image names are the column headings for and pertain to data in S1, S2, S4 and S5 Datasets. (ZIP) [file pone.0134978.s009.zip › S1_imagesfolder/AT1M3_D.png]

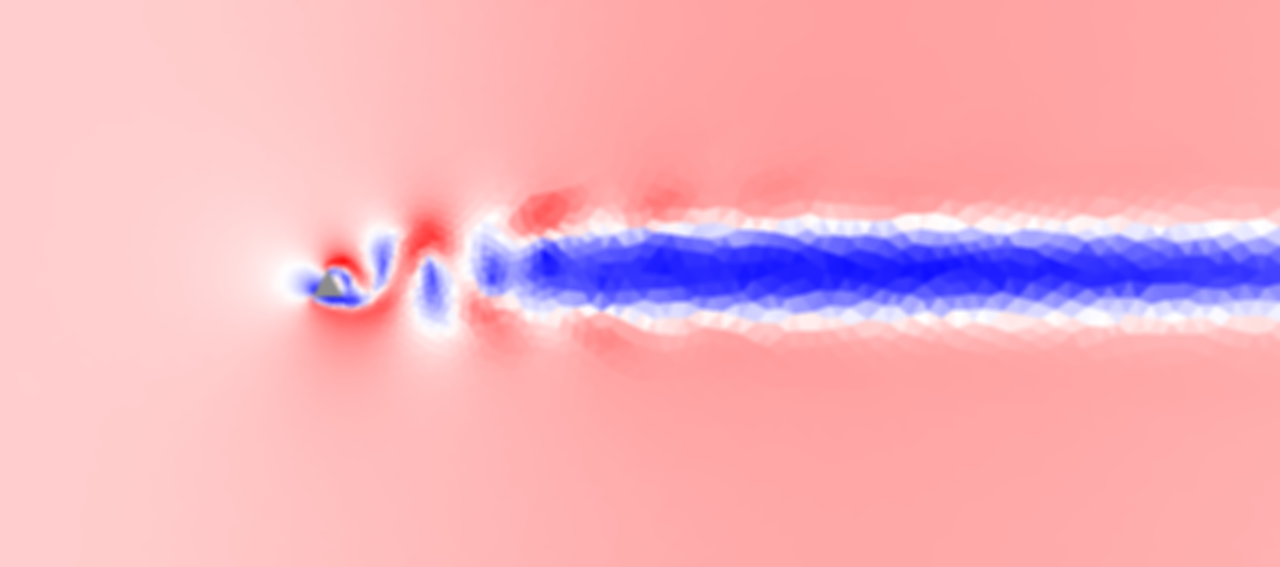

Supplement: S1 Images Folder — Image names are the column headings for and pertain to data in S1, S2, S4 and S5 Datasets. (ZIP) [file pone.0134978.s009.zip › S1_imagesfolder/AT2M1_B.png]

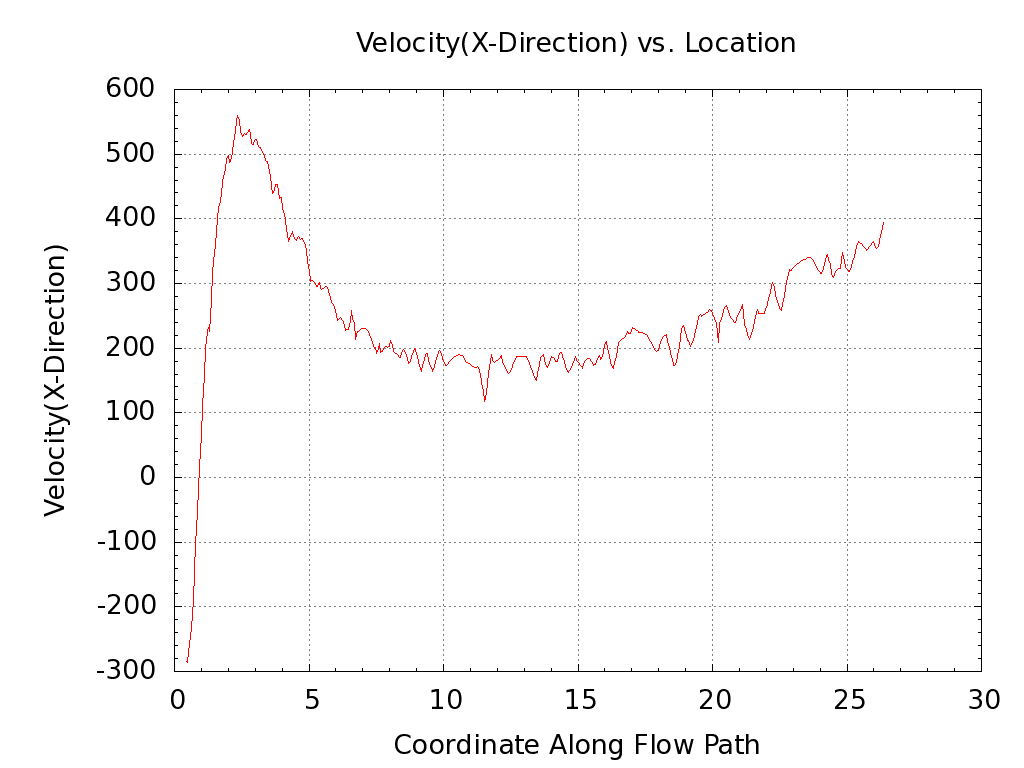

Supplement: S1 Images Folder — Image names are the column headings for and pertain to data in S1, S2, S4 and S5 Datasets. (ZIP) [file pone.0134978.s009.zip › S1_imagesfolder/AT2M1_C.png]

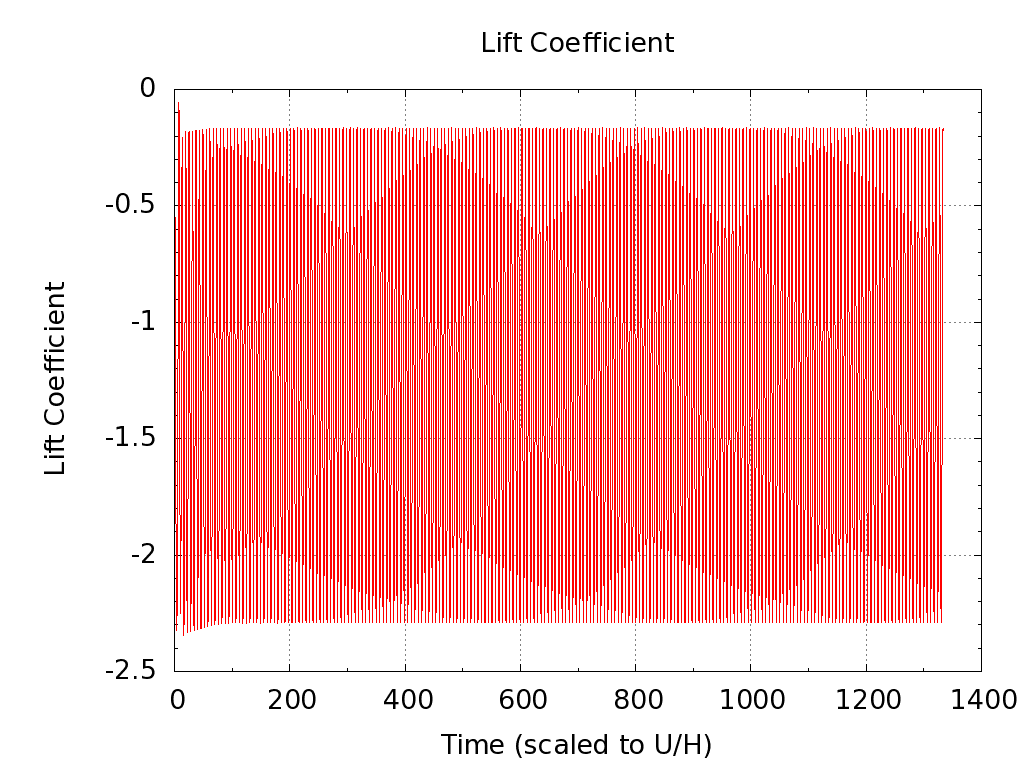

Supplement: S1 Images Folder — Image names are the column headings for and pertain to data in S1, S2, S4 and S5 Datasets. (ZIP) [file pone.0134978.s009.zip › S1_imagesfolder/AT2M1_D.png]

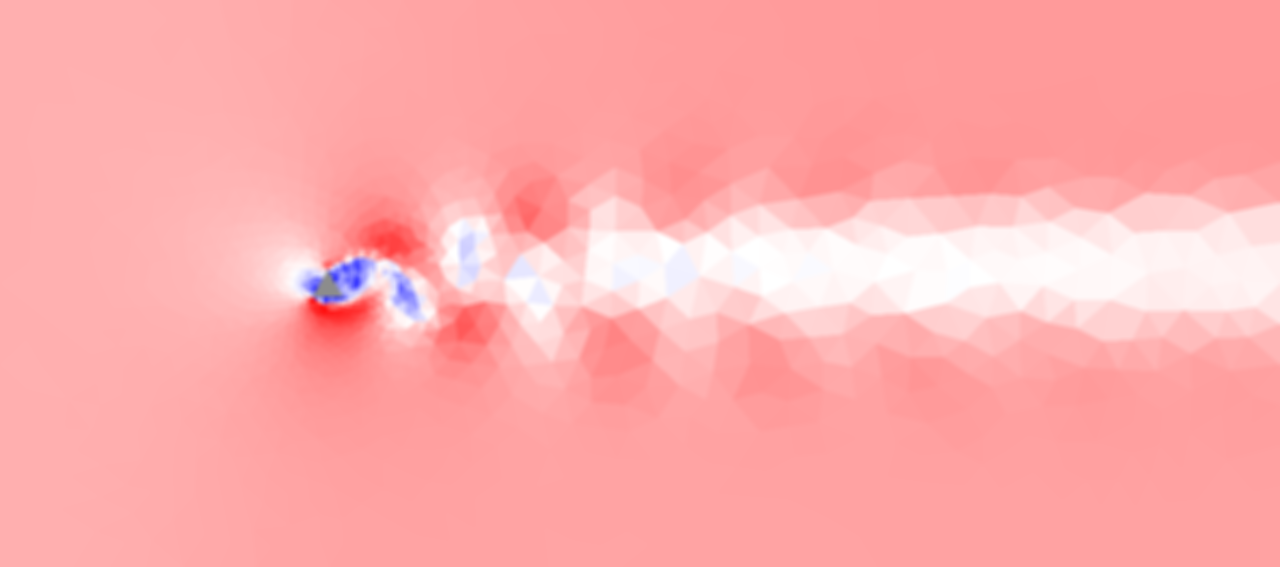

Supplement: S1 Images Folder — Image names are the column headings for and pertain to data in S1, S2, S4 and S5 Datasets. (ZIP) [file pone.0134978.s009.zip › S1_imagesfolder/AT2M2_B.png]

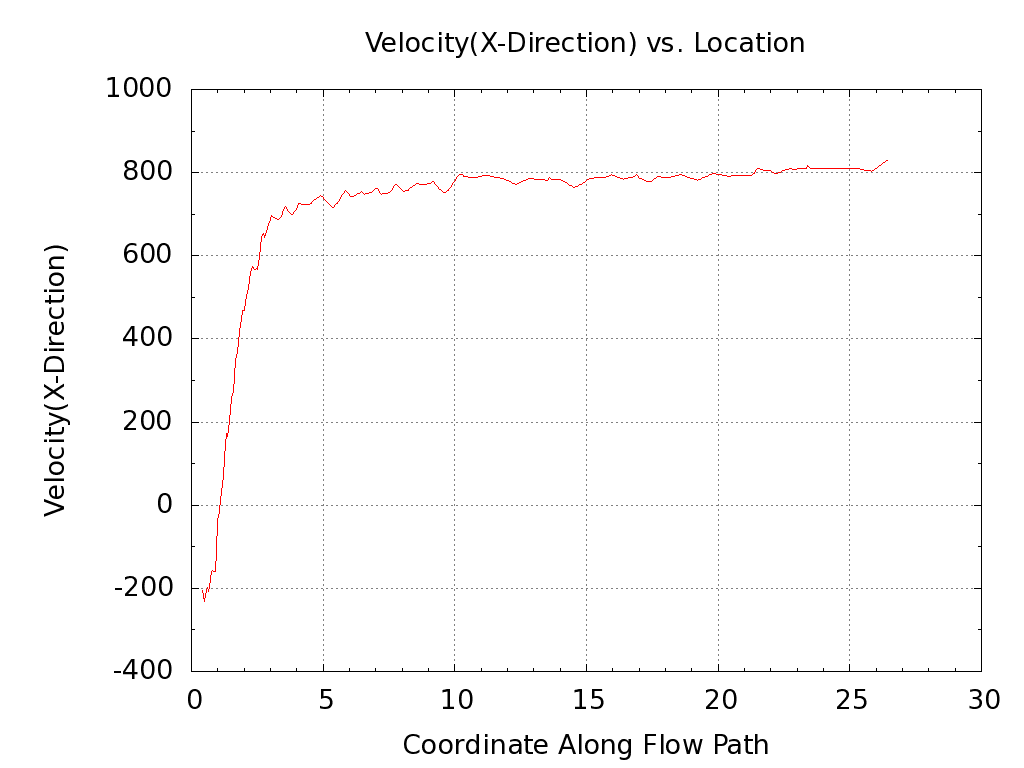

Supplement: S1 Images Folder — Image names are the column headings for and pertain to data in S1, S2, S4 and S5 Datasets. (ZIP) [file pone.0134978.s009.zip › S1_imagesfolder/AT2M2_C.png]

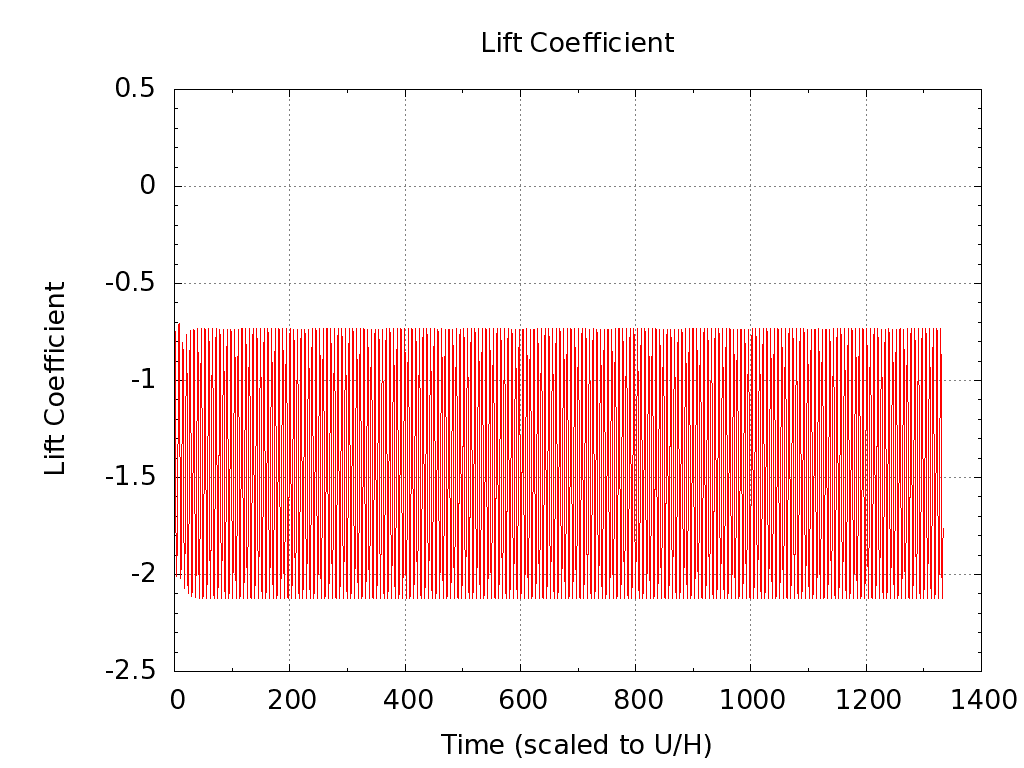

Supplement: S1 Images Folder — Image names are the column headings for and pertain to data in S1, S2, S4 and S5 Datasets. (ZIP) [file pone.0134978.s009.zip › S1_imagesfolder/AT2M2_D.png]

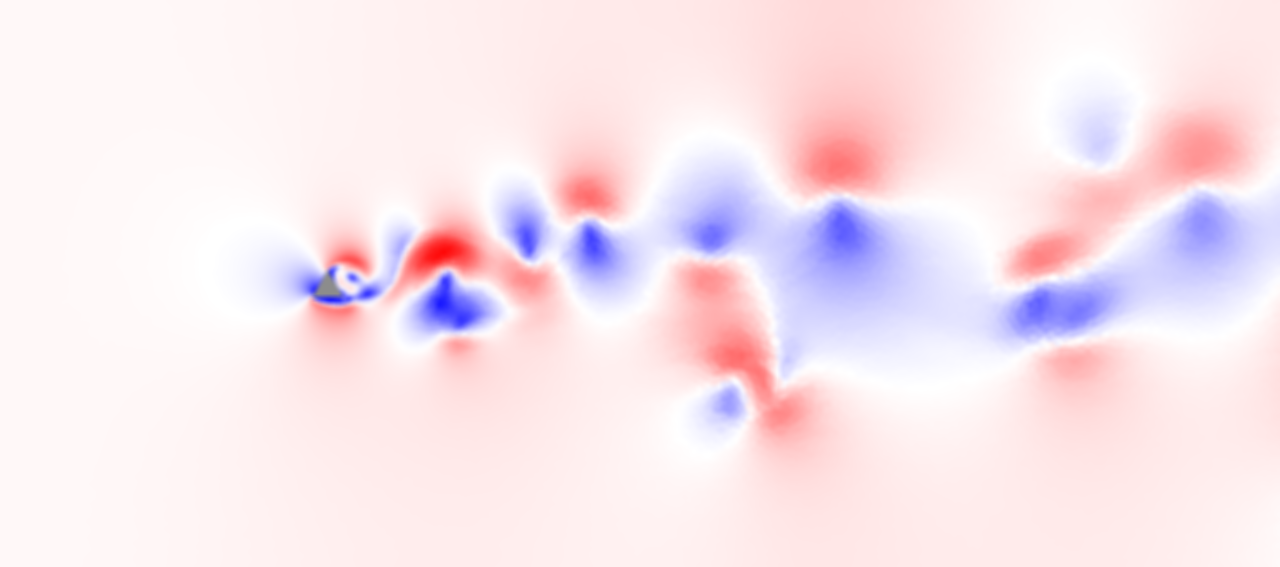

Supplement: S1 Images Folder — Image names are the column headings for and pertain to data in S1, S2, S4 and S5 Datasets. (ZIP) [file pone.0134978.s009.zip › S1_imagesfolder/AT2M3_B.png]

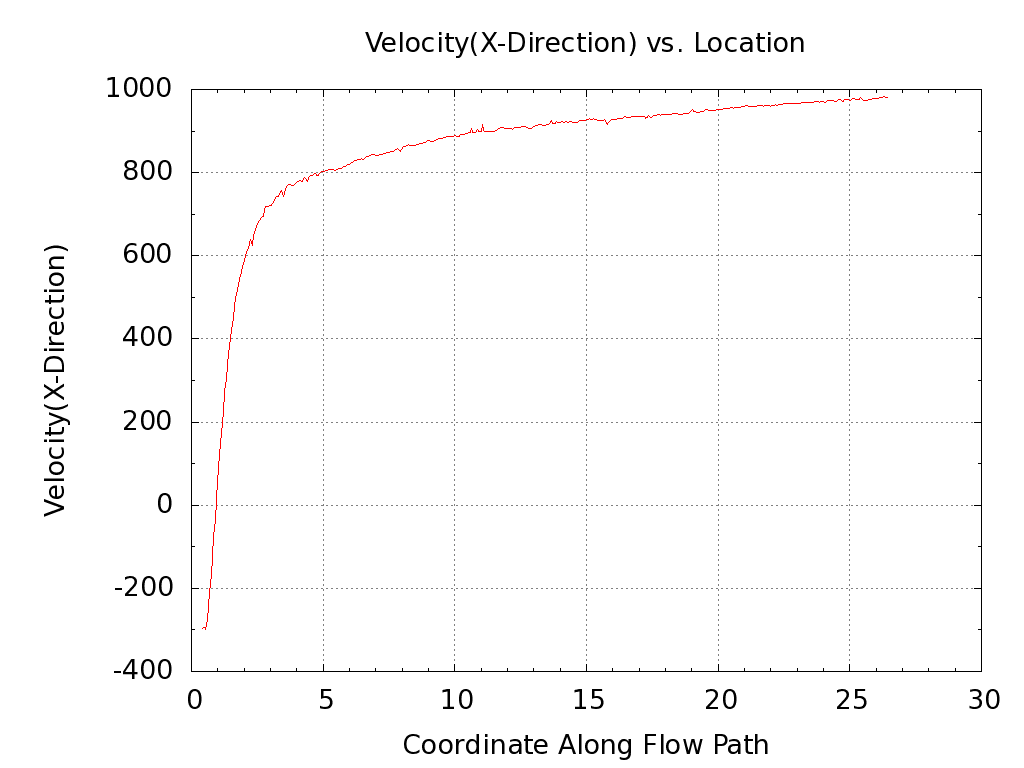

Supplement: S1 Images Folder — Image names are the column headings for and pertain to data in S1, S2, S4 and S5 Datasets. (ZIP) [file pone.0134978.s009.zip › S1_imagesfolder/AT2M3_C.png]

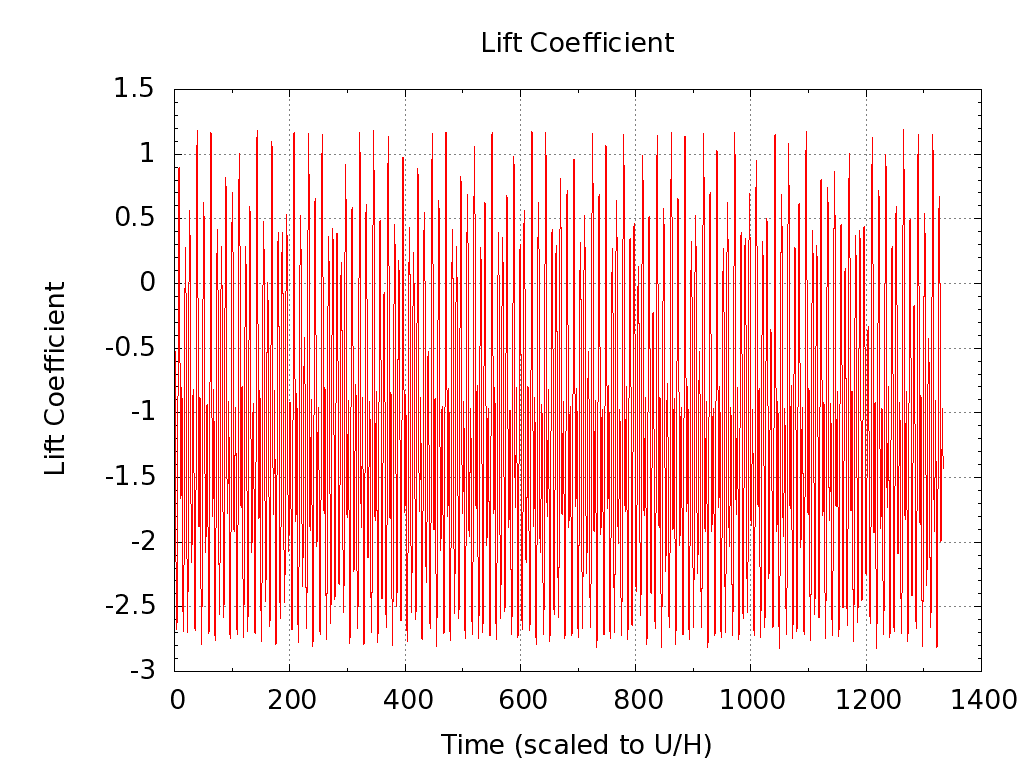

Supplement: S1 Images Folder — Image names are the column headings for and pertain to data in S1, S2, S4 and S5 Datasets. (ZIP) [file pone.0134978.s009.zip › S1_imagesfolder/AT2M3_D.png]

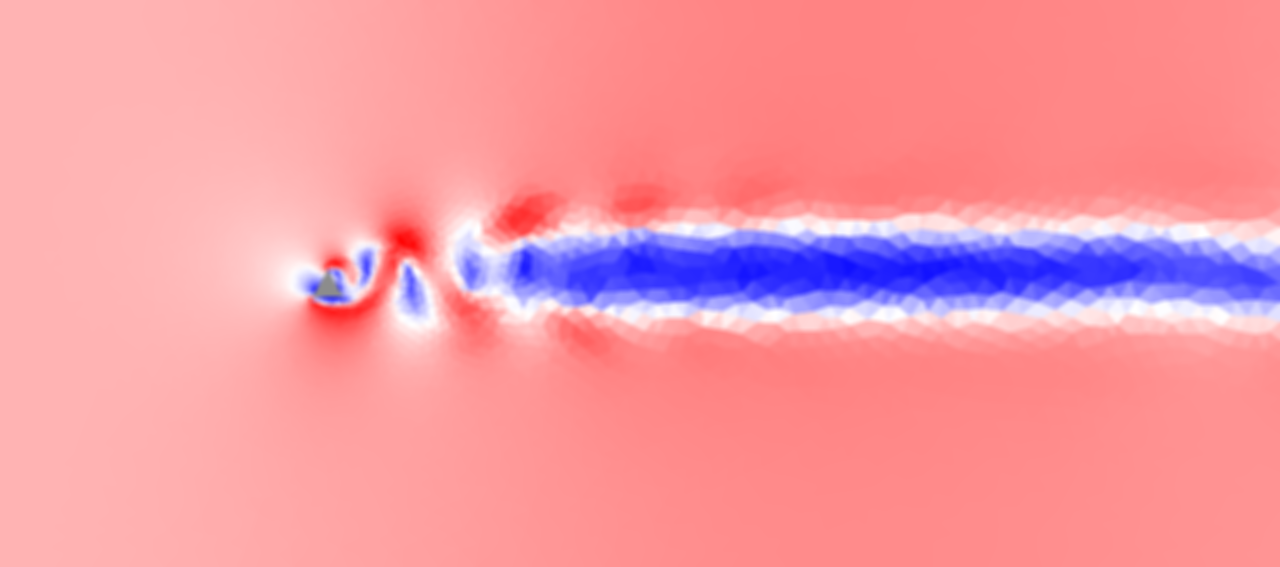

Supplement: S1 Images Folder — Image names are the column headings for and pertain to data in S1, S2, S4 and S5 Datasets. (ZIP) [file pone.0134978.s009.zip › S1_imagesfolder/AT3M1_B.png]

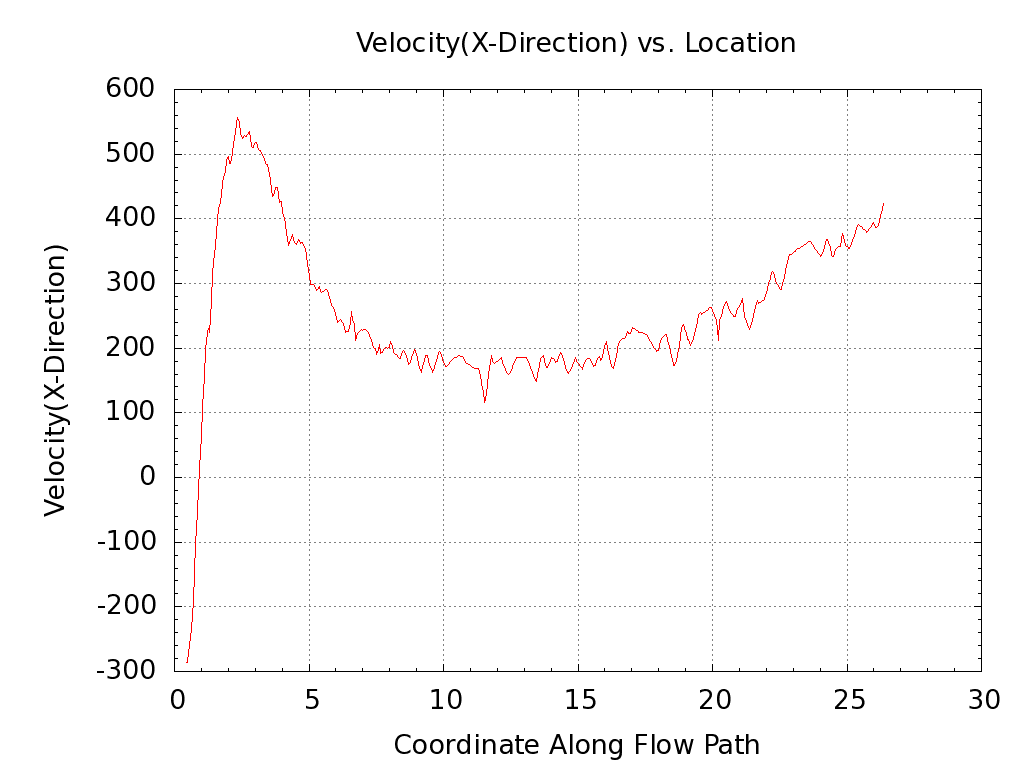

Supplement: S1 Images Folder — Image names are the column headings for and pertain to data in S1, S2, S4 and S5 Datasets. (ZIP) [file pone.0134978.s009.zip › S1_imagesfolder/AT3M1_C.png]

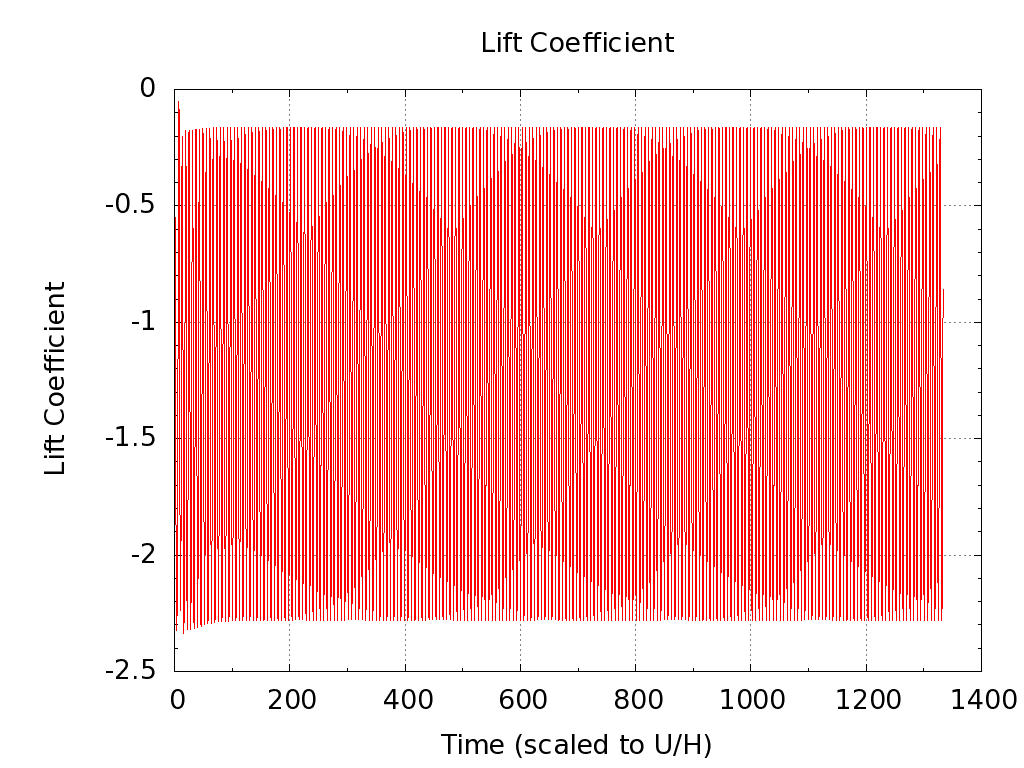

Supplement: S1 Images Folder — Image names are the column headings for and pertain to data in S1, S2, S4 and S5 Datasets. (ZIP) [file pone.0134978.s009.zip › S1_imagesfolder/AT3M1_D.png]

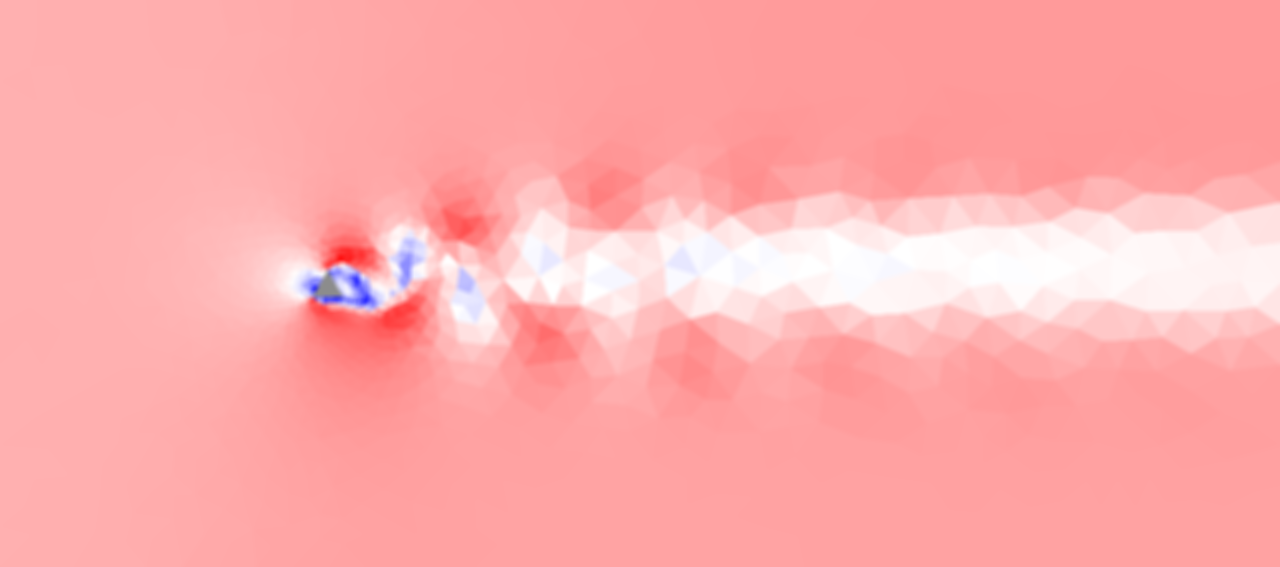

Supplement: S1 Images Folder — Image names are the column headings for and pertain to data in S1, S2, S4 and S5 Datasets. (ZIP) [file pone.0134978.s009.zip › S1_imagesfolder/AT3M2_B.png]

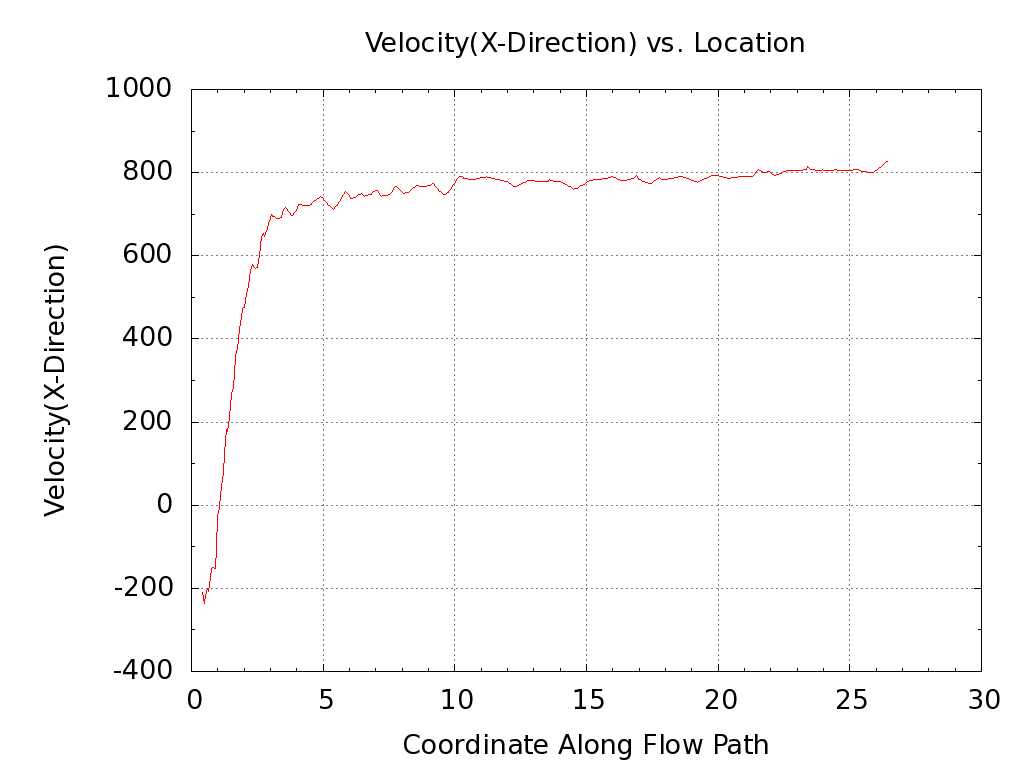

Supplement: S1 Images Folder — Image names are the column headings for and pertain to data in S1, S2, S4 and S5 Datasets. (ZIP) [file pone.0134978.s009.zip › S1_imagesfolder/AT3M2_C.png]

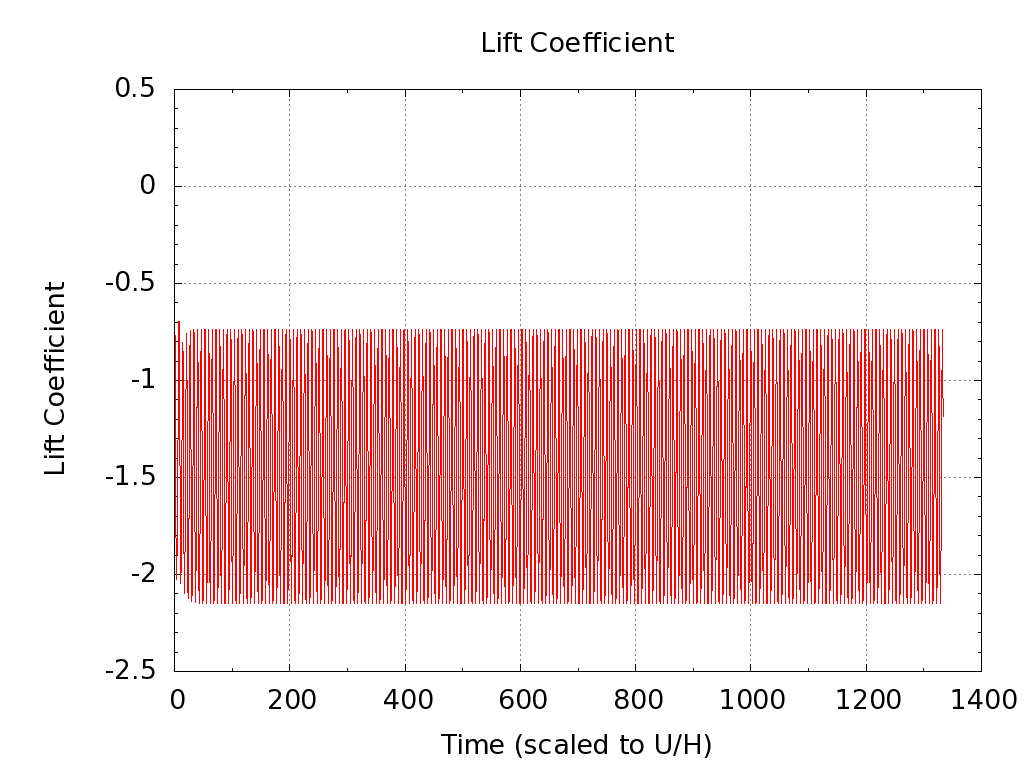

Supplement: S1 Images Folder — Image names are the column headings for and pertain to data in S1, S2, S4 and S5 Datasets. (ZIP) [file pone.0134978.s009.zip › S1_imagesfolder/AT3M2_D.png]

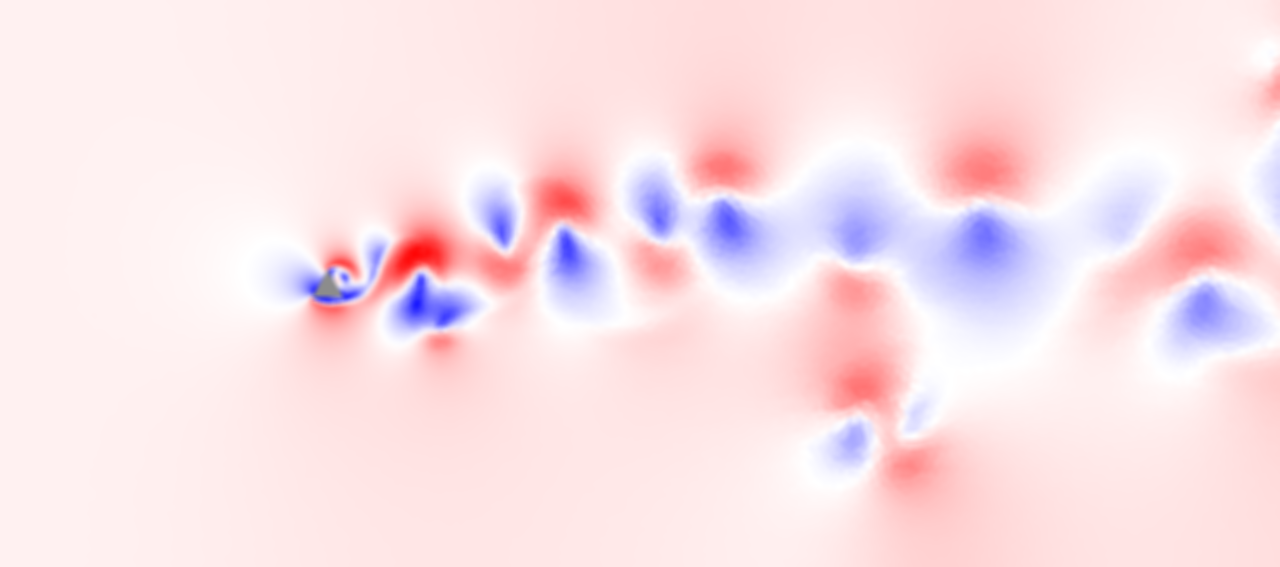

Supplement: S1 Images Folder — Image names are the column headings for and pertain to data in S1, S2, S4 and S5 Datasets. (ZIP) [file pone.0134978.s009.zip › S1_imagesfolder/AT3M3_B.png]

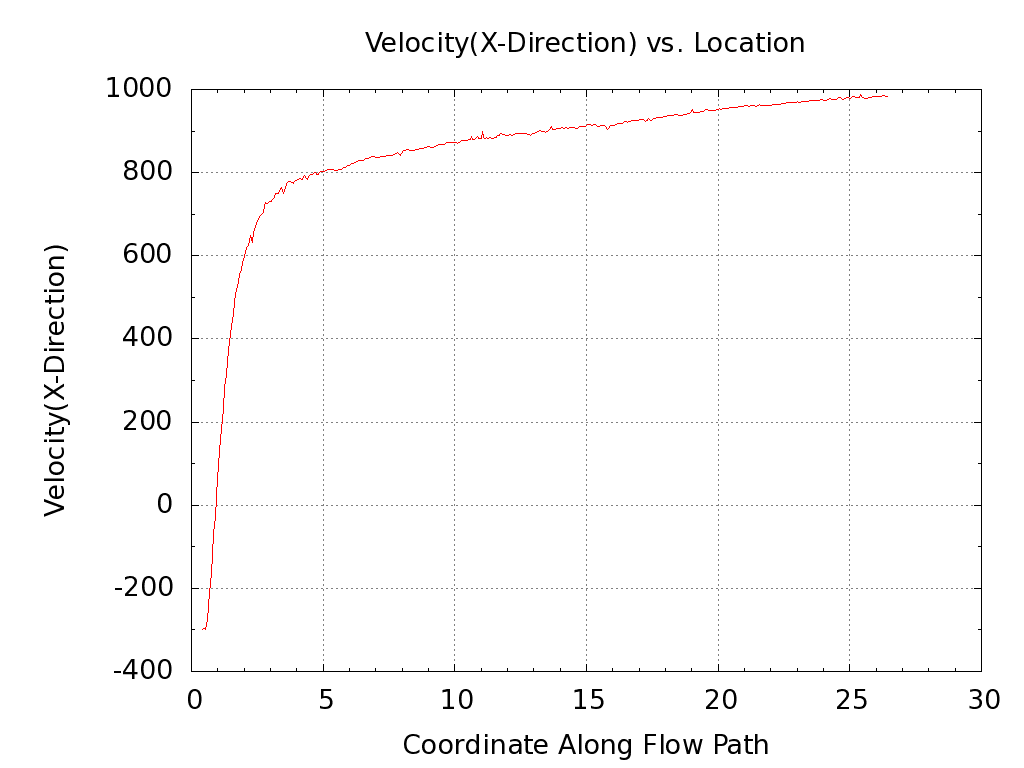

Supplement: S1 Images Folder — Image names are the column headings for and pertain to data in S1, S2, S4 and S5 Datasets. (ZIP) [file pone.0134978.s009.zip › S1_imagesfolder/AT3M3_C.png]

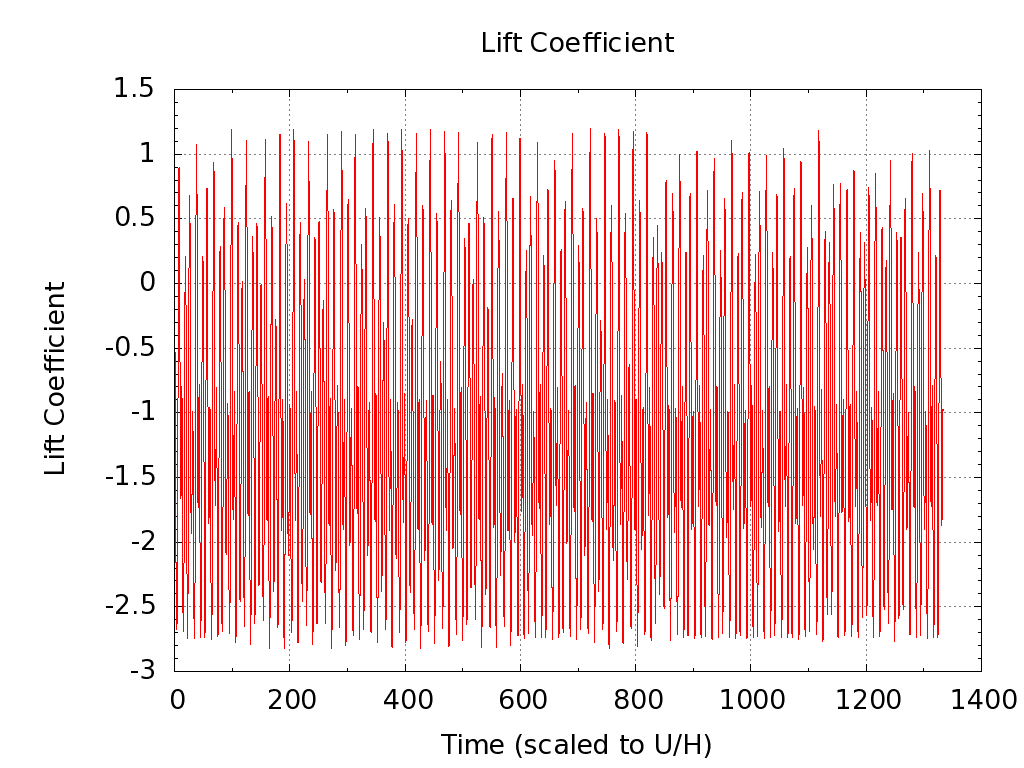

Supplement: S1 Images Folder — Image names are the column headings for and pertain to data in S1, S2, S4 and S5 Datasets. (ZIP) [file pone.0134978.s009.zip › S1_imagesfolder/AT3M3_D.png]

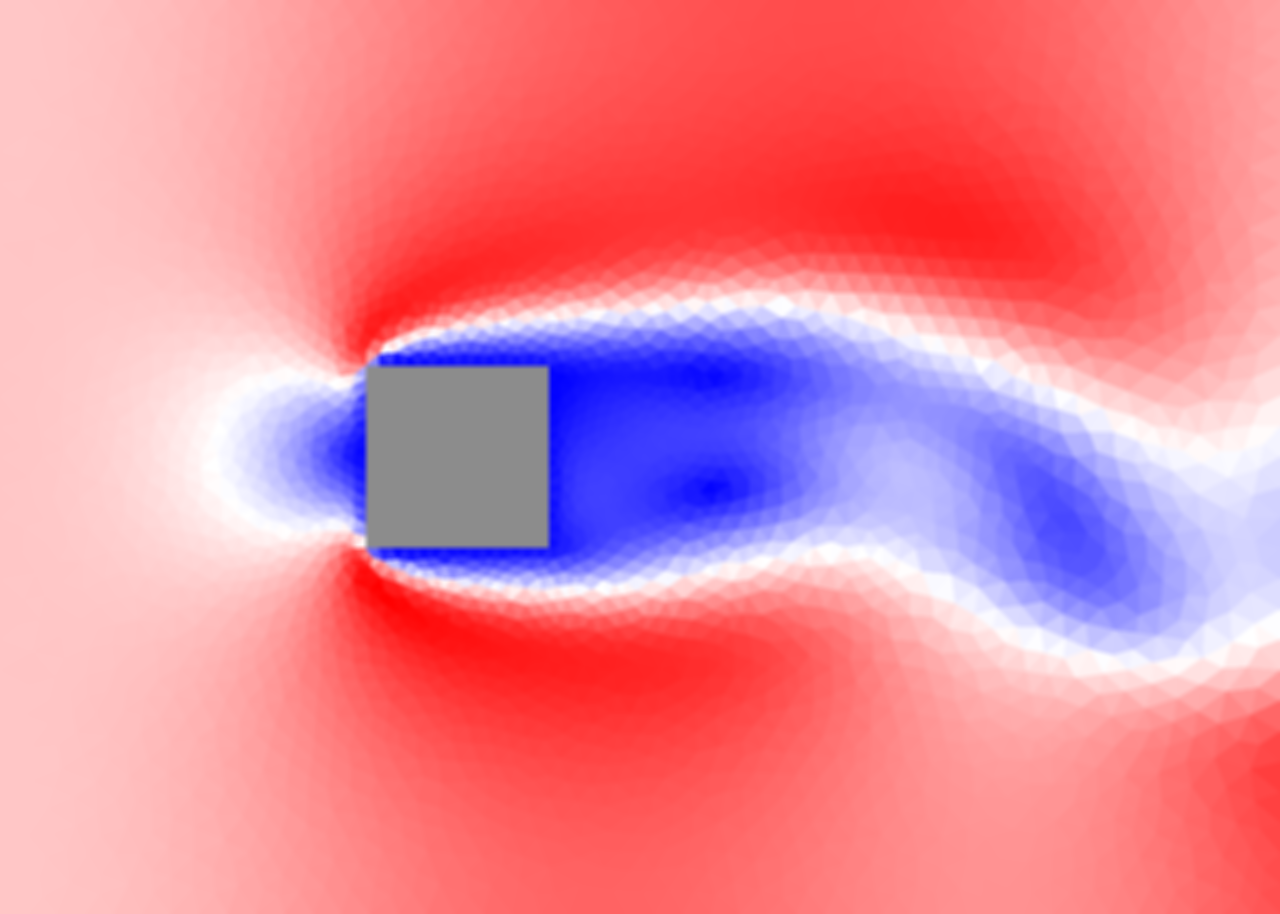

Supplement: S1 Images Folder — Image names are the column headings for and pertain to data in S1, S2, S4 and S5 Datasets. (ZIP) [file pone.0134978.s009.zip › S1_imagesfolder/BDom1_B.png]

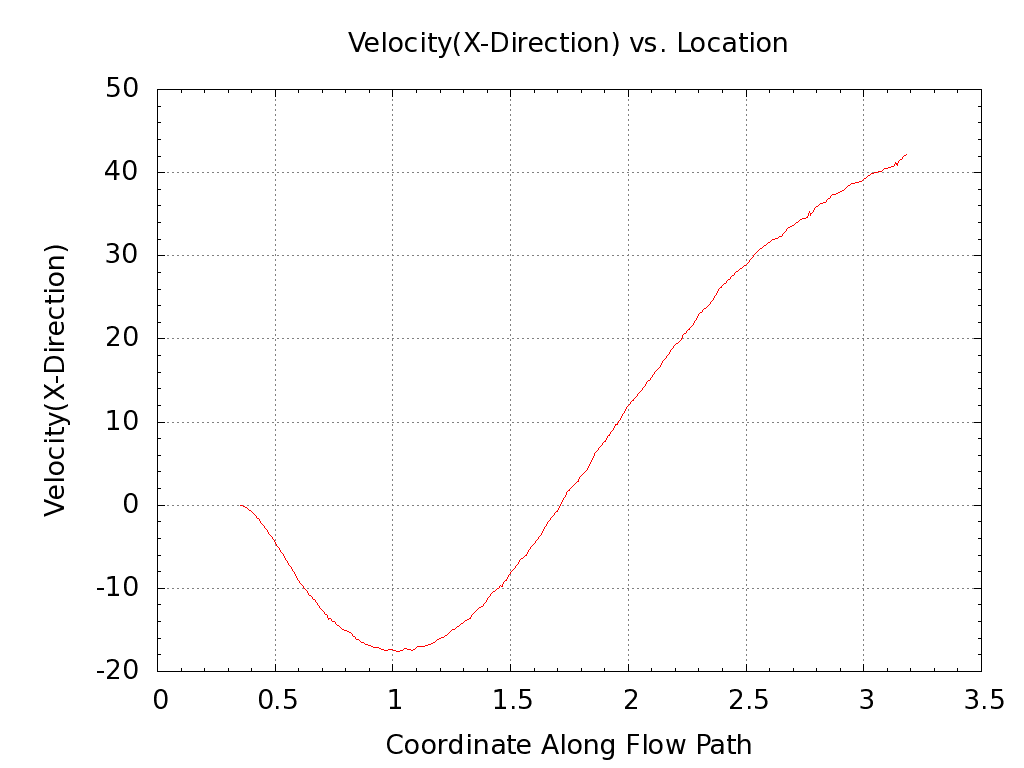

Supplement: S1 Images Folder — Image names are the column headings for and pertain to data in S1, S2, S4 and S5 Datasets. (ZIP) [file pone.0134978.s009.zip › S1_imagesfolder/BDom1_C.png]

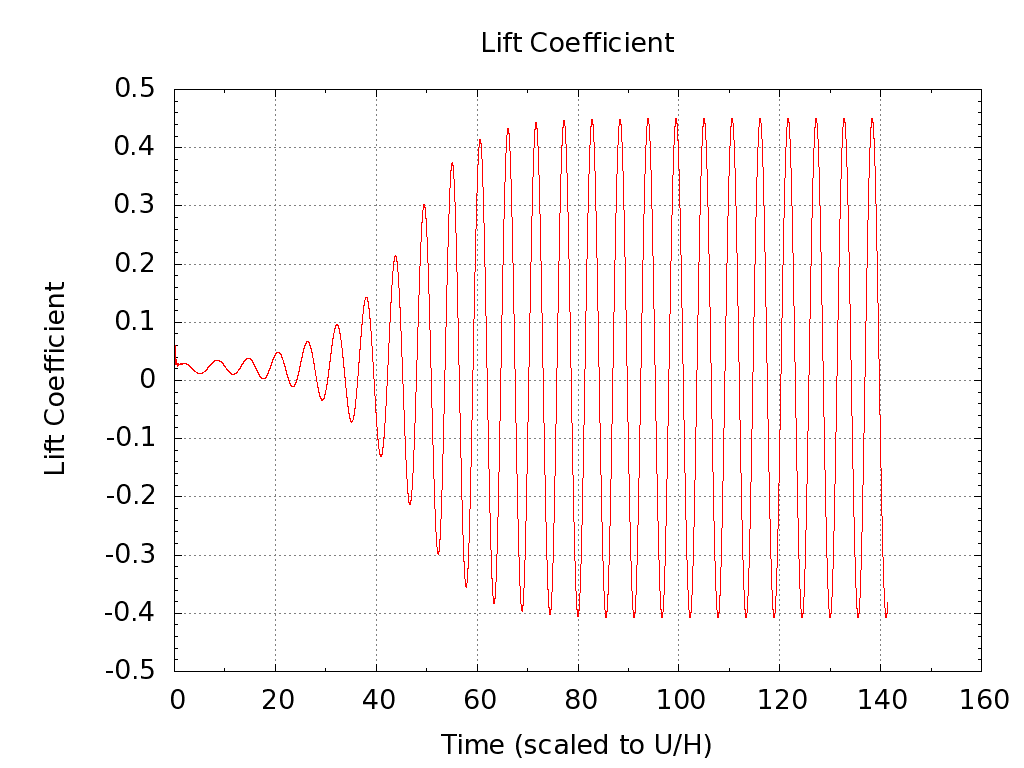

Supplement: S1 Images Folder — Image names are the column headings for and pertain to data in S1, S2, S4 and S5 Datasets. (ZIP) [file pone.0134978.s009.zip › S1_imagesfolder/BDom1_D.png]

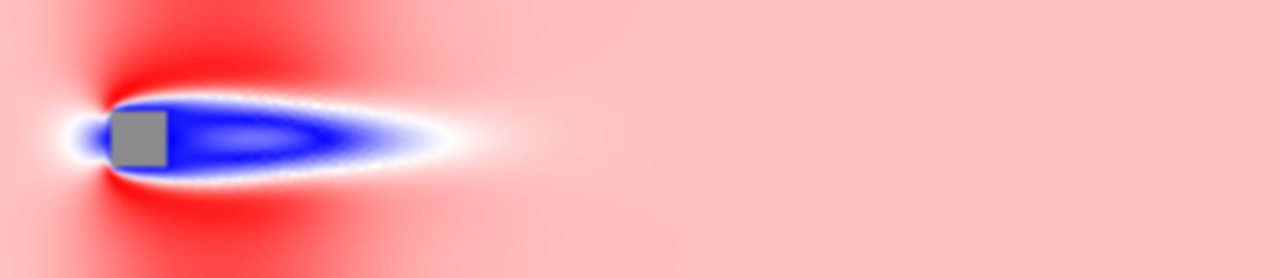

Supplement: S1 Images Folder — Image names are the column headings for and pertain to data in S1, S2, S4 and S5 Datasets. (ZIP) [file pone.0134978.s009.zip › S1_imagesfolder/BDur1_B.png]

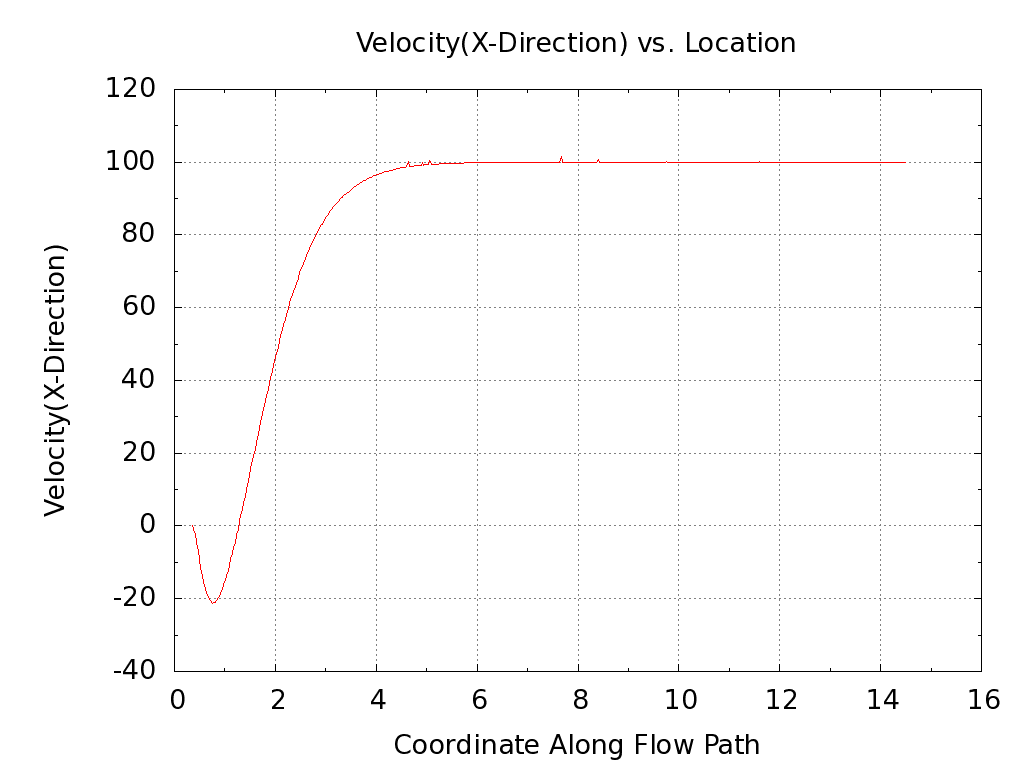

Supplement: S1 Images Folder — Image names are the column headings for and pertain to data in S1, S2, S4 and S5 Datasets. (ZIP) [file pone.0134978.s009.zip › S1_imagesfolder/BDur1_C.png]

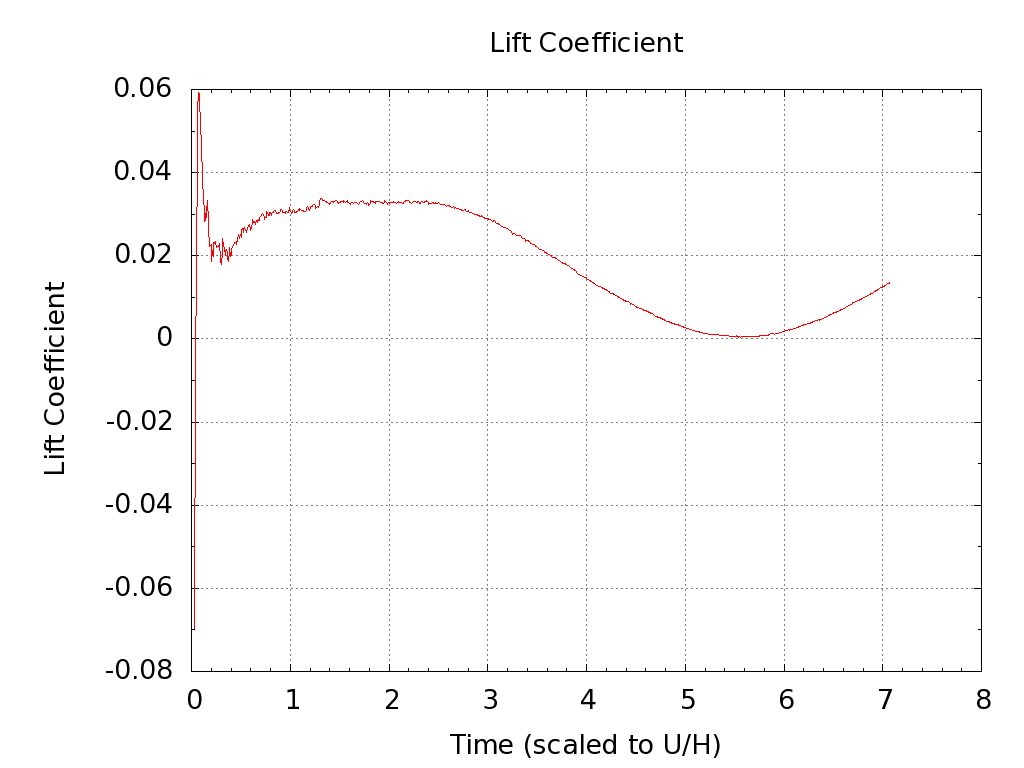

Supplement: S1 Images Folder — Image names are the column headings for and pertain to data in S1, S2, S4 and S5 Datasets. (ZIP) [file pone.0134978.s009.zip › S1_imagesfolder/BDur1_D.png]

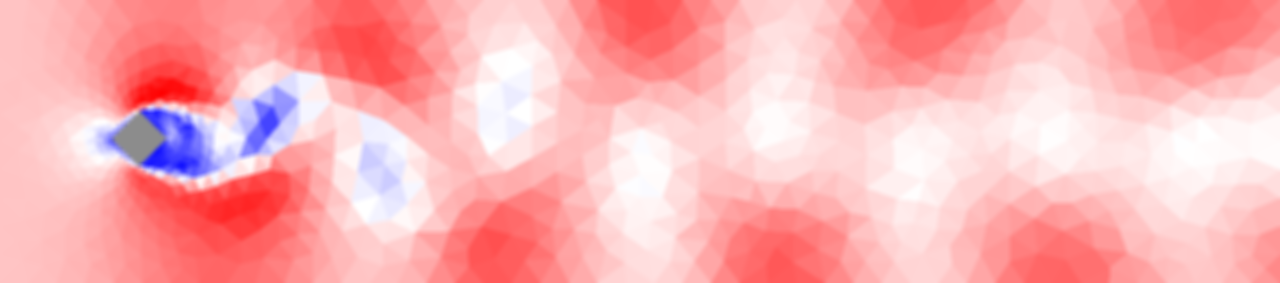

Supplement: S1 Images Folder — Image names are the column headings for and pertain to data in S1, S2, S4 and S5 Datasets. (ZIP) [file pone.0134978.s009.zip › S1_imagesfolder/BT1M1_B.png]

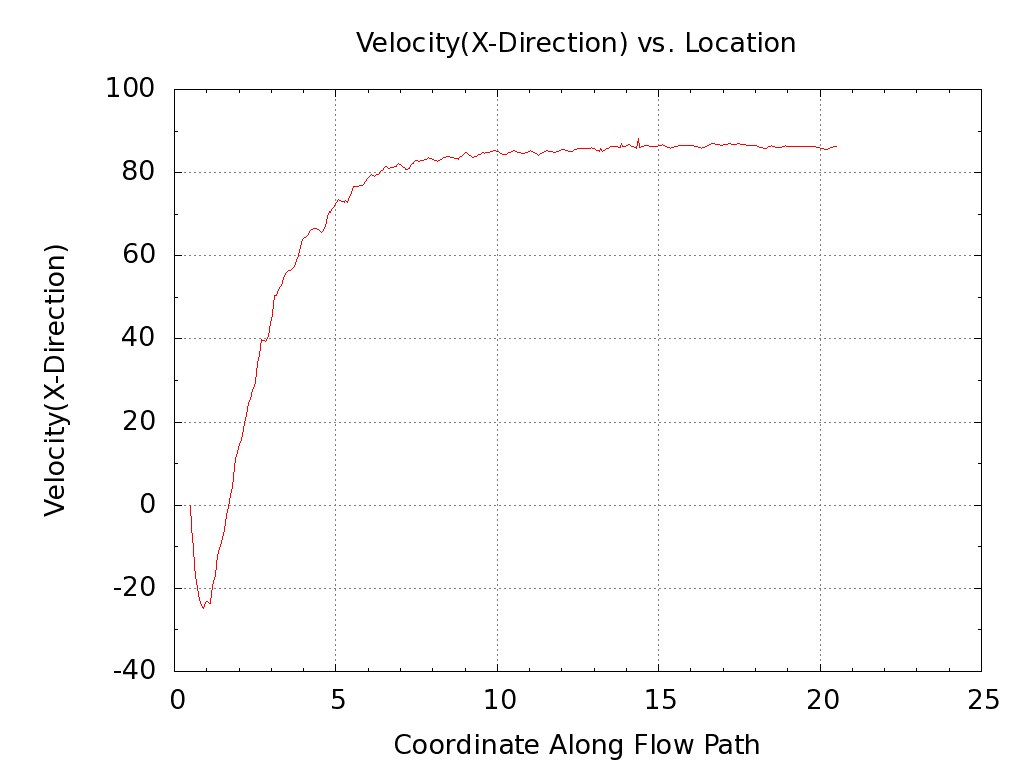

Supplement: S1 Images Folder — Image names are the column headings for and pertain to data in S1, S2, S4 and S5 Datasets. (ZIP) [file pone.0134978.s009.zip › S1_imagesfolder/BT1M1_C.png]

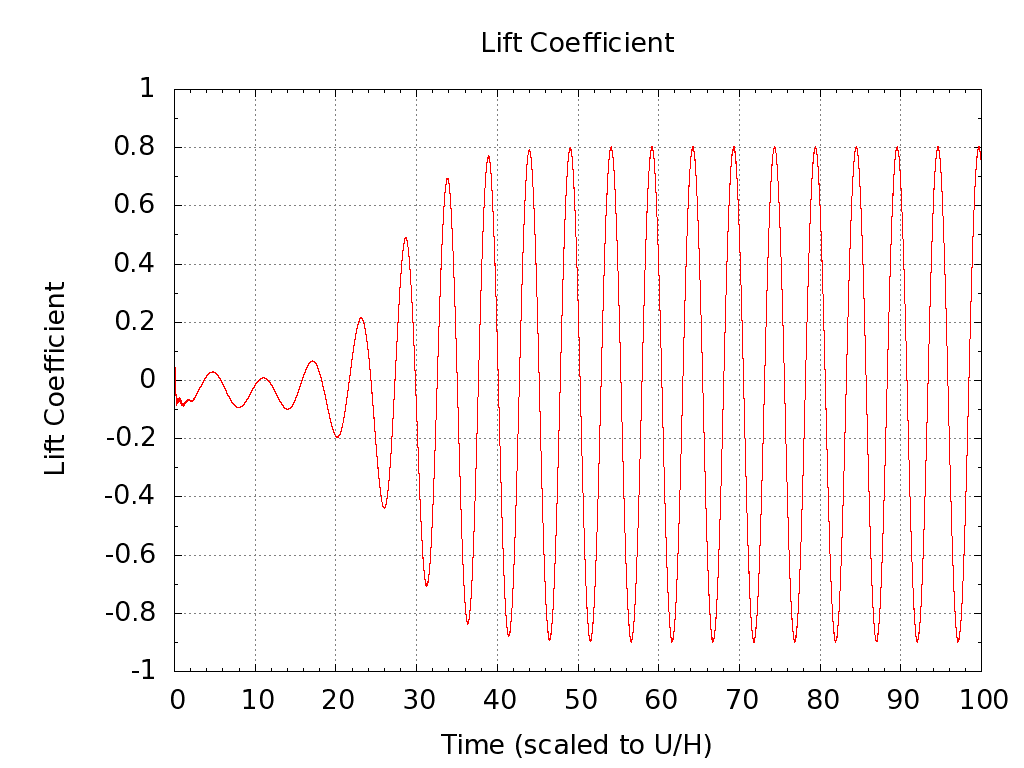

Supplement: S1 Images Folder — Image names are the column headings for and pertain to data in S1, S2, S4 and S5 Datasets. (ZIP) [file pone.0134978.s009.zip › S1_imagesfolder/BT1M1_D.png]

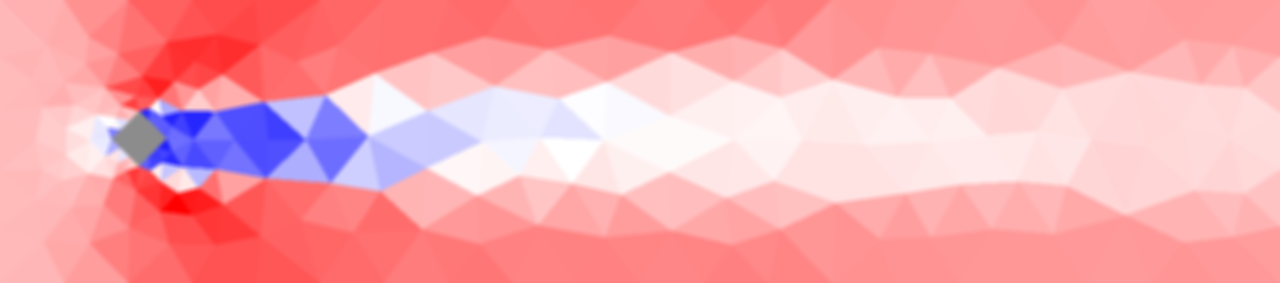

Supplement: S1 Images Folder — Image names are the column headings for and pertain to data in S1, S2, S4 and S5 Datasets. (ZIP) [file pone.0134978.s009.zip › S1_imagesfolder/BT1M2_B.png]

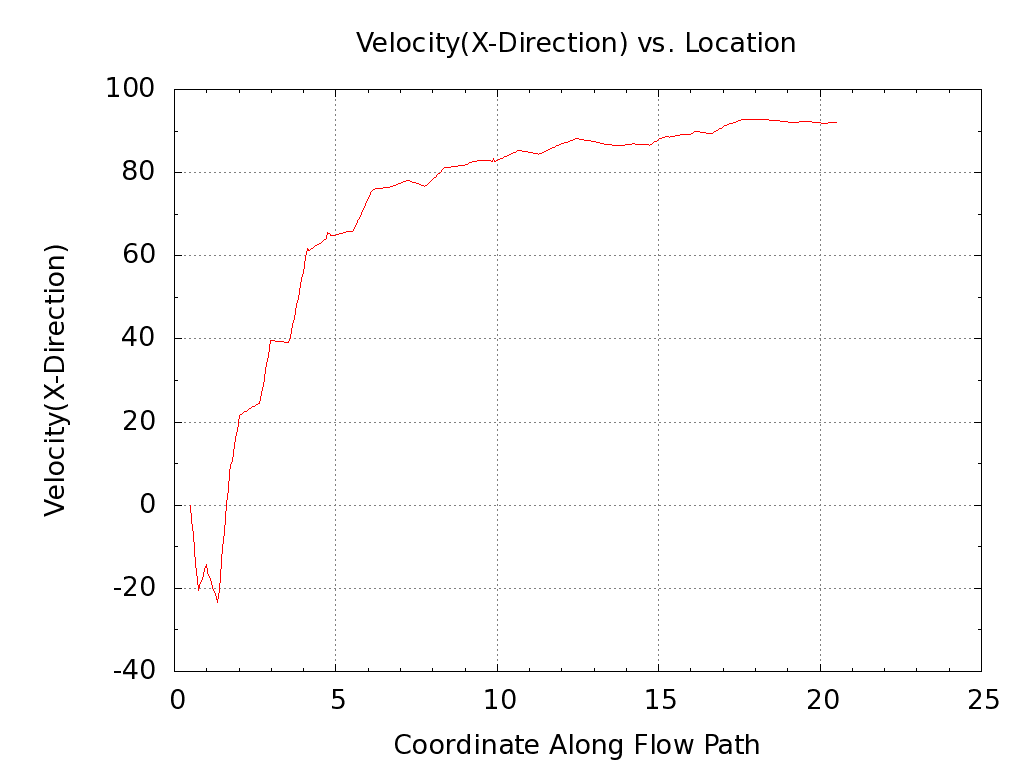

Supplement: S1 Images Folder — Image names are the column headings for and pertain to data in S1, S2, S4 and S5 Datasets. (ZIP) [file pone.0134978.s009.zip › S1_imagesfolder/BT1M2_C.png]

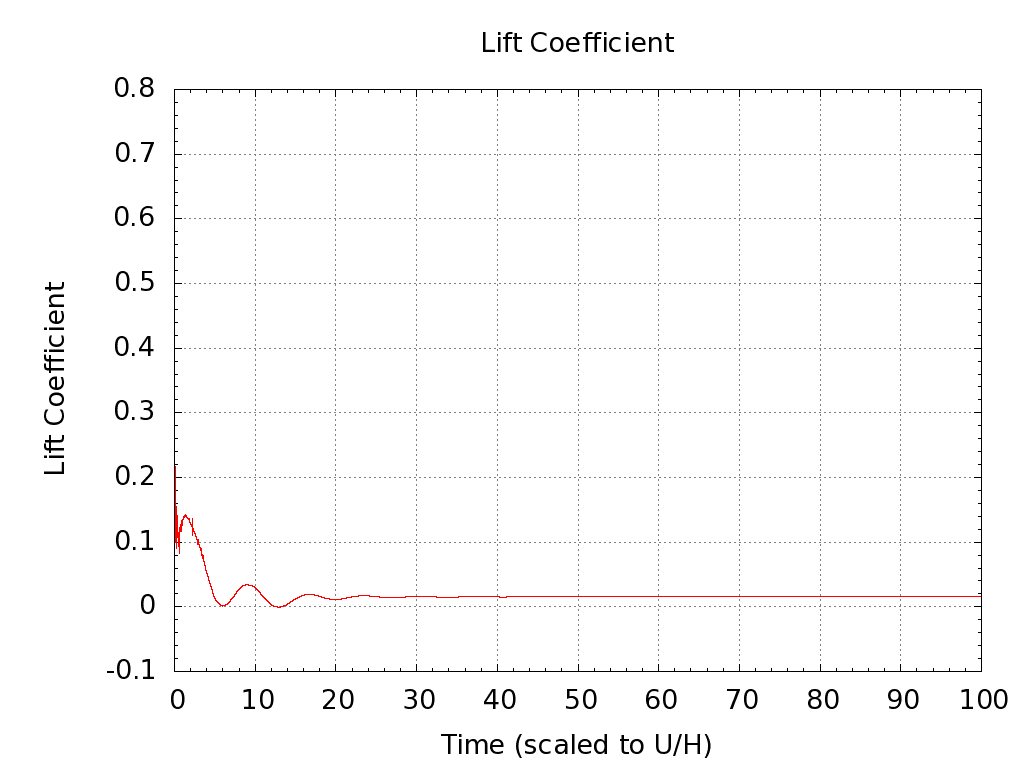

Supplement: S1 Images Folder — Image names are the column headings for and pertain to data in S1, S2, S4 and S5 Datasets. (ZIP) [file pone.0134978.s009.zip › S1_imagesfolder/BT1M2_D.png]

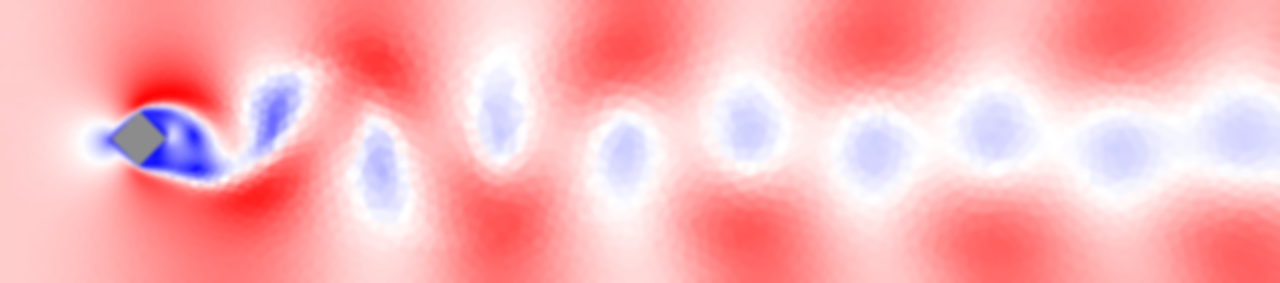

Supplement: S1 Images Folder — Image names are the column headings for and pertain to data in S1, S2, S4 and S5 Datasets. (ZIP) [file pone.0134978.s009.zip › S1_imagesfolder/BT1M3_B.png]

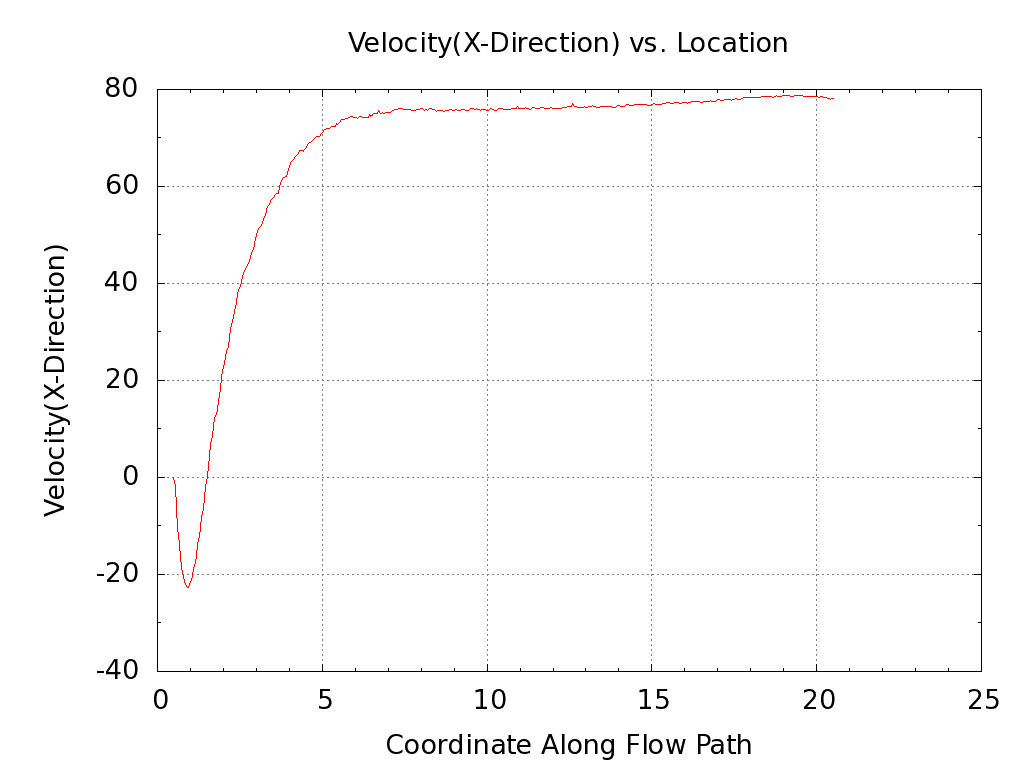

Supplement: S1 Images Folder — Image names are the column headings for and pertain to data in S1, S2, S4 and S5 Datasets. (ZIP) [file pone.0134978.s009.zip › S1_imagesfolder/BT1M3_C.png]

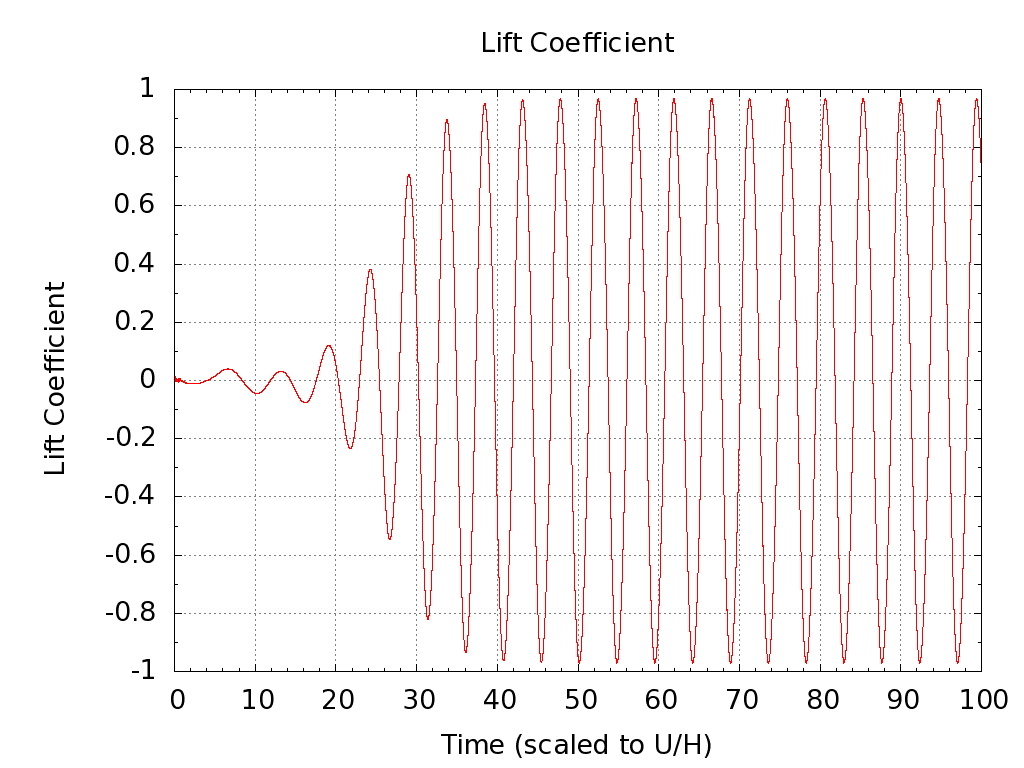

Supplement: S1 Images Folder — Image names are the column headings for and pertain to data in S1, S2, S4 and S5 Datasets. (ZIP) [file pone.0134978.s009.zip › S1_imagesfolder/BT1M3_D.png]

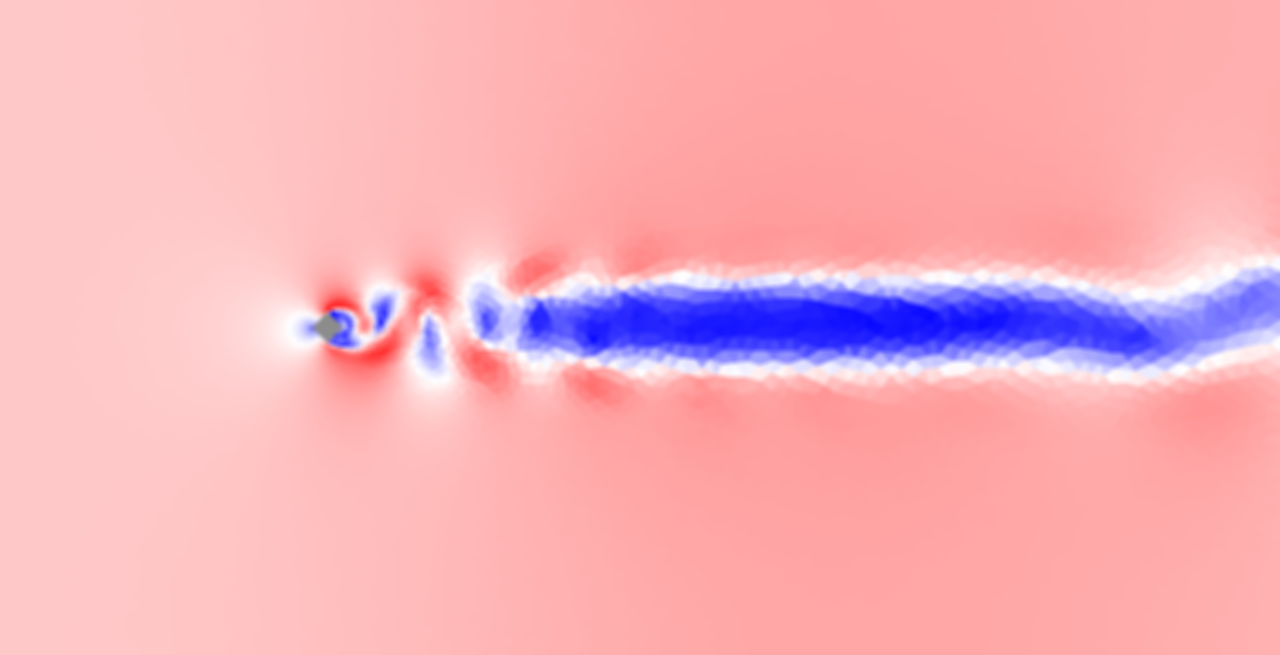

Supplement: S1 Images Folder — Image names are the column headings for and pertain to data in S1, S2, S4 and S5 Datasets. (ZIP) [file pone.0134978.s009.zip › S1_imagesfolder/BT2M1_B.png]

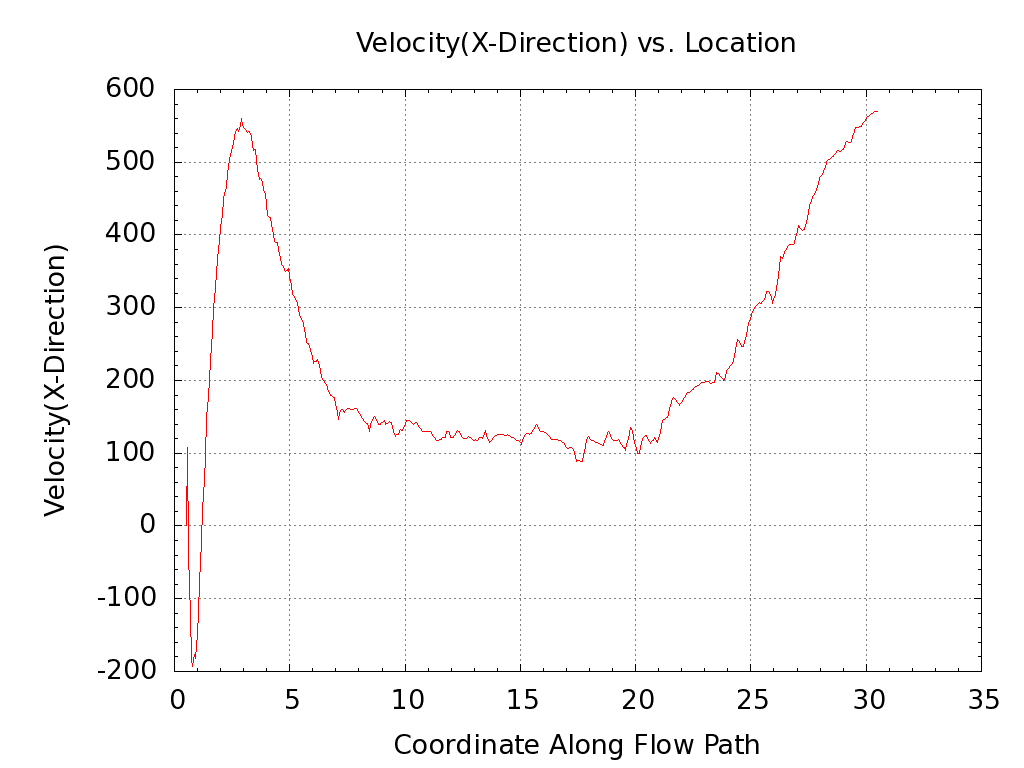

Supplement: S1 Images Folder — Image names are the column headings for and pertain to data in S1, S2, S4 and S5 Datasets. (ZIP) [file pone.0134978.s009.zip › S1_imagesfolder/BT2M1_C.png]

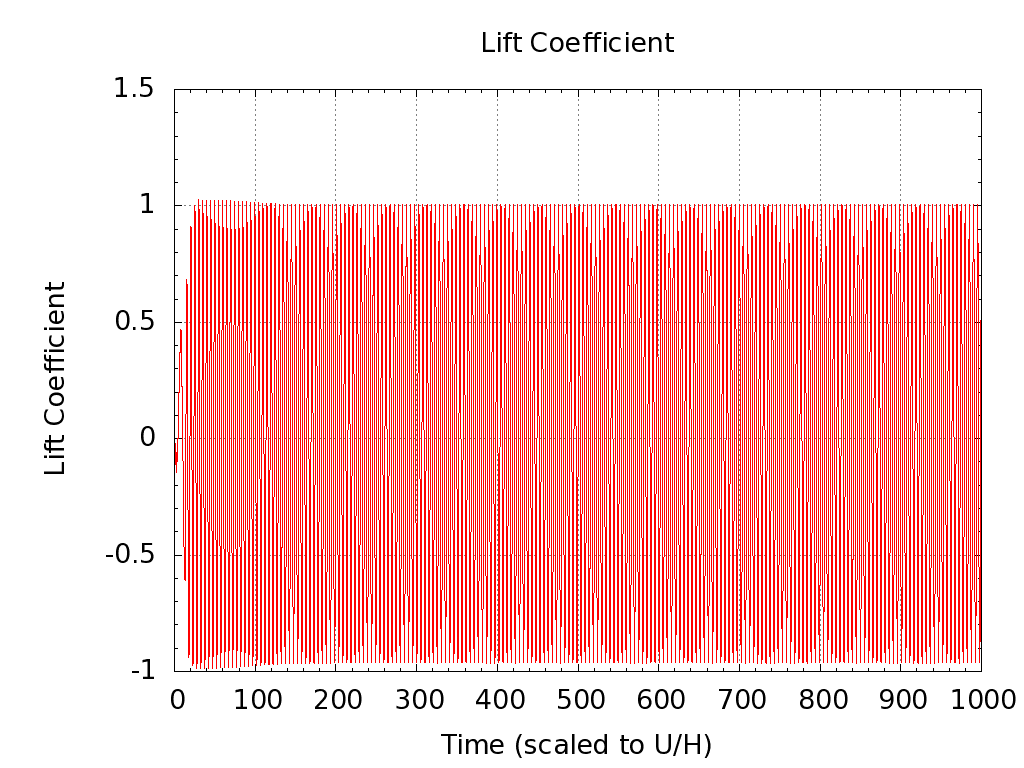

Supplement: S1 Images Folder — Image names are the column headings for and pertain to data in S1, S2, S4 and S5 Datasets. (ZIP) [file pone.0134978.s009.zip › S1_imagesfolder/BT2M1_D.png]

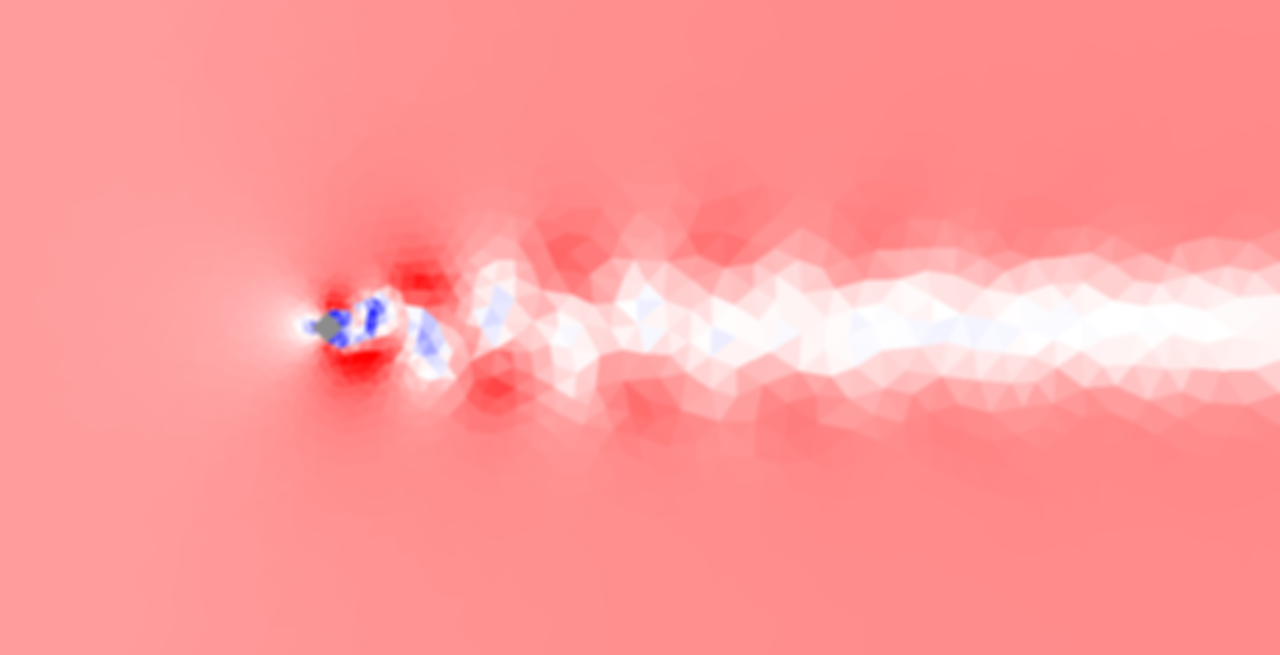

Supplement: S1 Images Folder — Image names are the column headings for and pertain to data in S1, S2, S4 and S5 Datasets. (ZIP) [file pone.0134978.s009.zip › S1_imagesfolder/BT2M2_B.png]

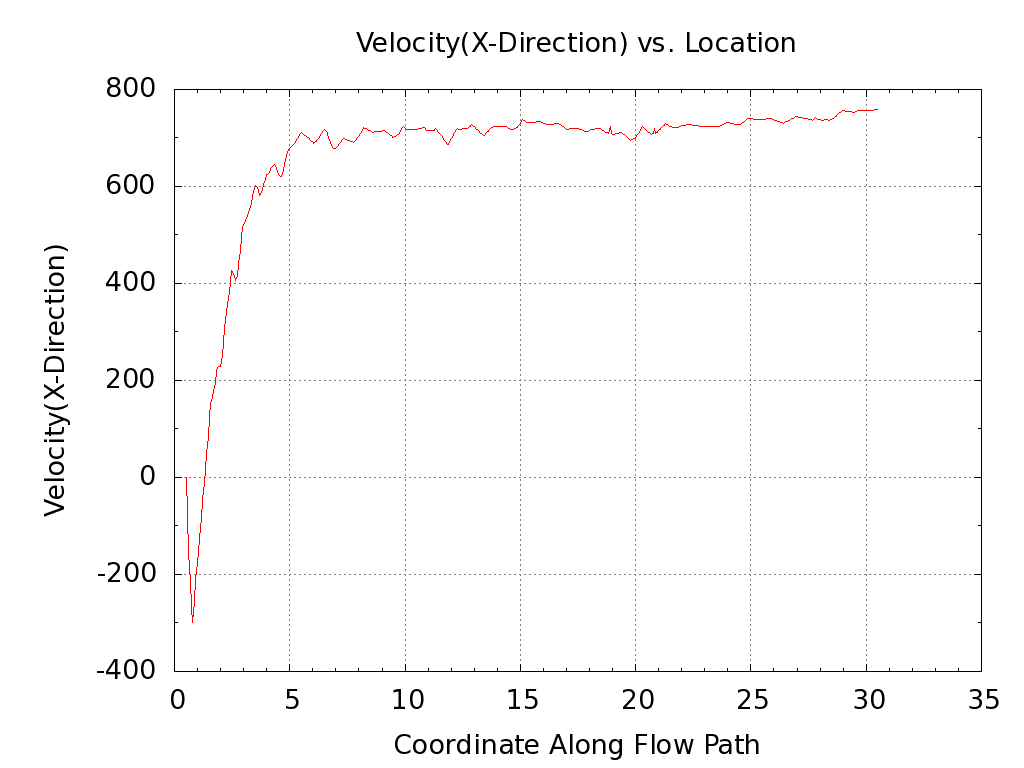

Supplement: S1 Images Folder — Image names are the column headings for and pertain to data in S1, S2, S4 and S5 Datasets. (ZIP) [file pone.0134978.s009.zip › S1_imagesfolder/BT2M2_C.png]

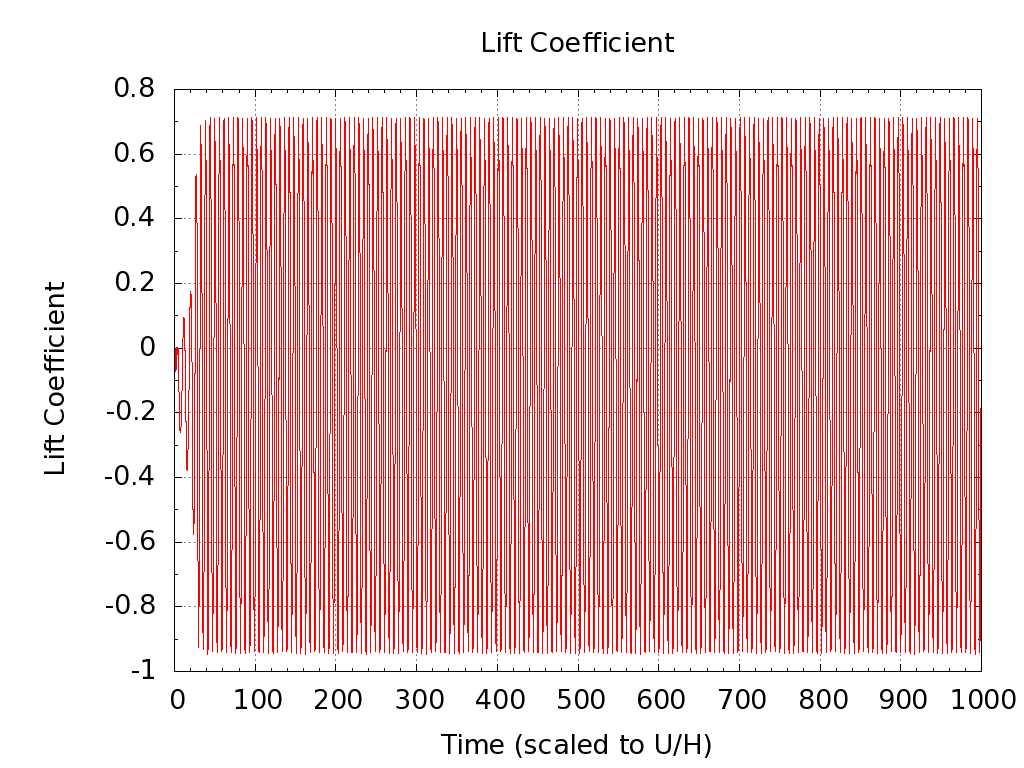

Supplement: S1 Images Folder — Image names are the column headings for and pertain to data in S1, S2, S4 and S5 Datasets. (ZIP) [file pone.0134978.s009.zip › S1_imagesfolder/BT2M2_D.png]

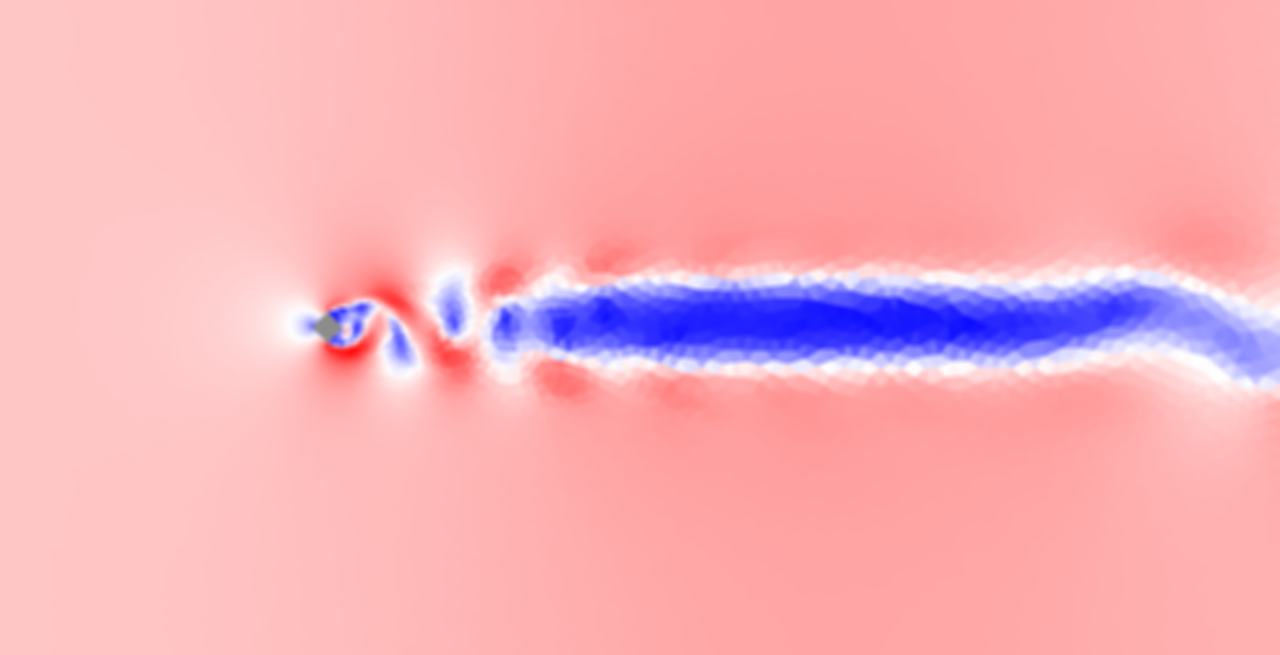

Supplement: S1 Images Folder — Image names are the column headings for and pertain to data in S1, S2, S4 and S5 Datasets. (ZIP) [file pone.0134978.s009.zip › S1_imagesfolder/BT3M1_B.png]

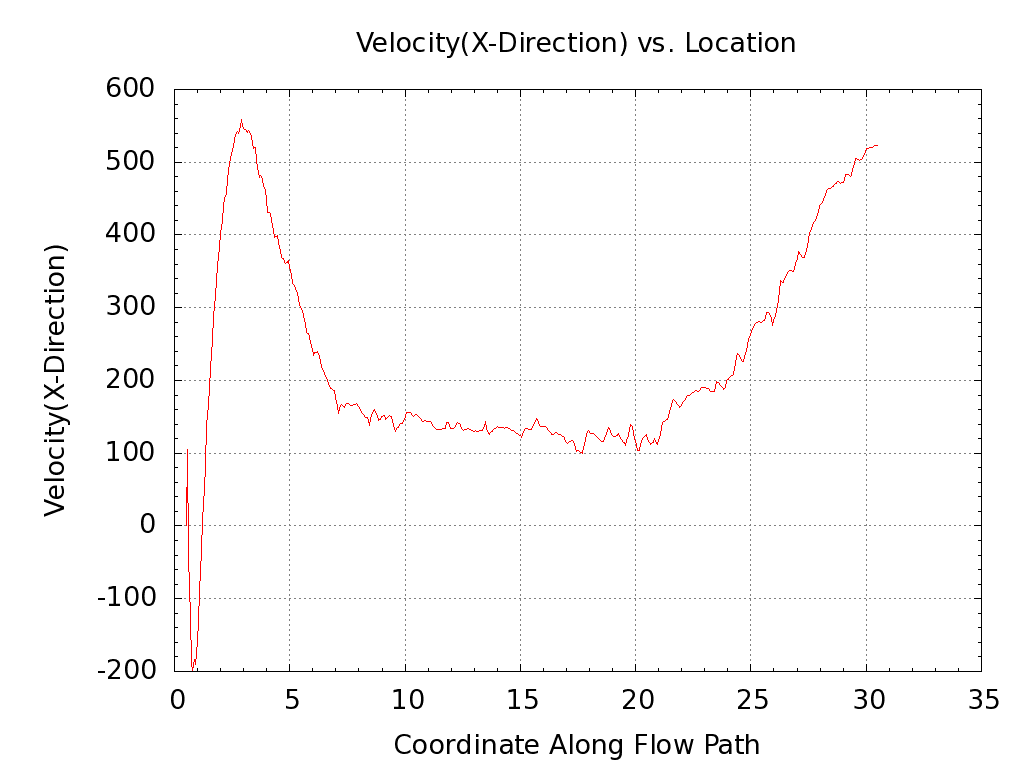

Supplement: S1 Images Folder — Image names are the column headings for and pertain to data in S1, S2, S4 and S5 Datasets. (ZIP) [file pone.0134978.s009.zip › S1_imagesfolder/BT3M1_C.png]

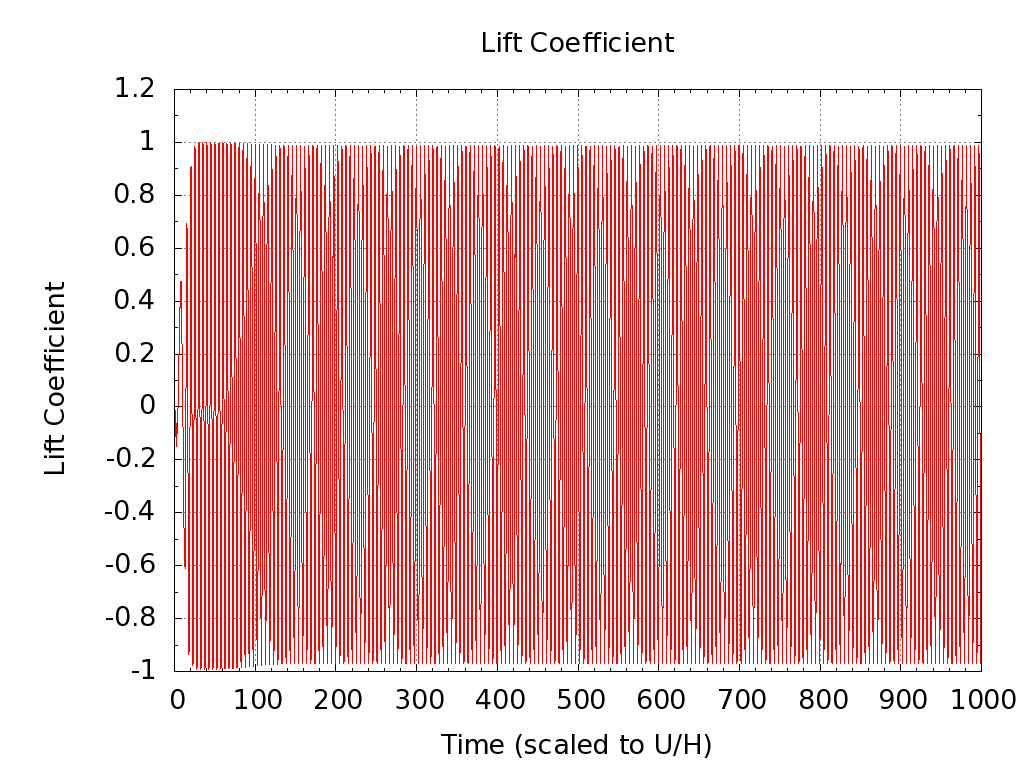

Supplement: S1 Images Folder — Image names are the column headings for and pertain to data in S1, S2, S4 and S5 Datasets. (ZIP) [file pone.0134978.s009.zip › S1_imagesfolder/BT3M1_D.png]

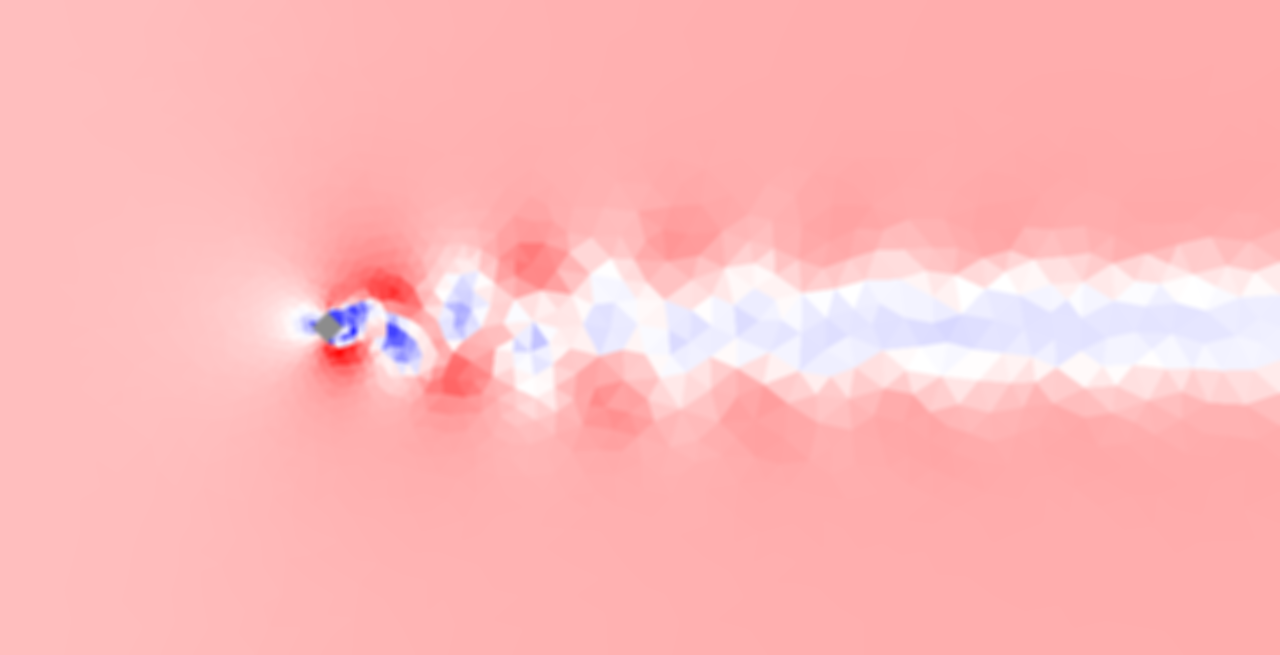

Supplement: S1 Images Folder — Image names are the column headings for and pertain to data in S1, S2, S4 and S5 Datasets. (ZIP) [file pone.0134978.s009.zip › S1_imagesfolder/BT3M2_B.png]

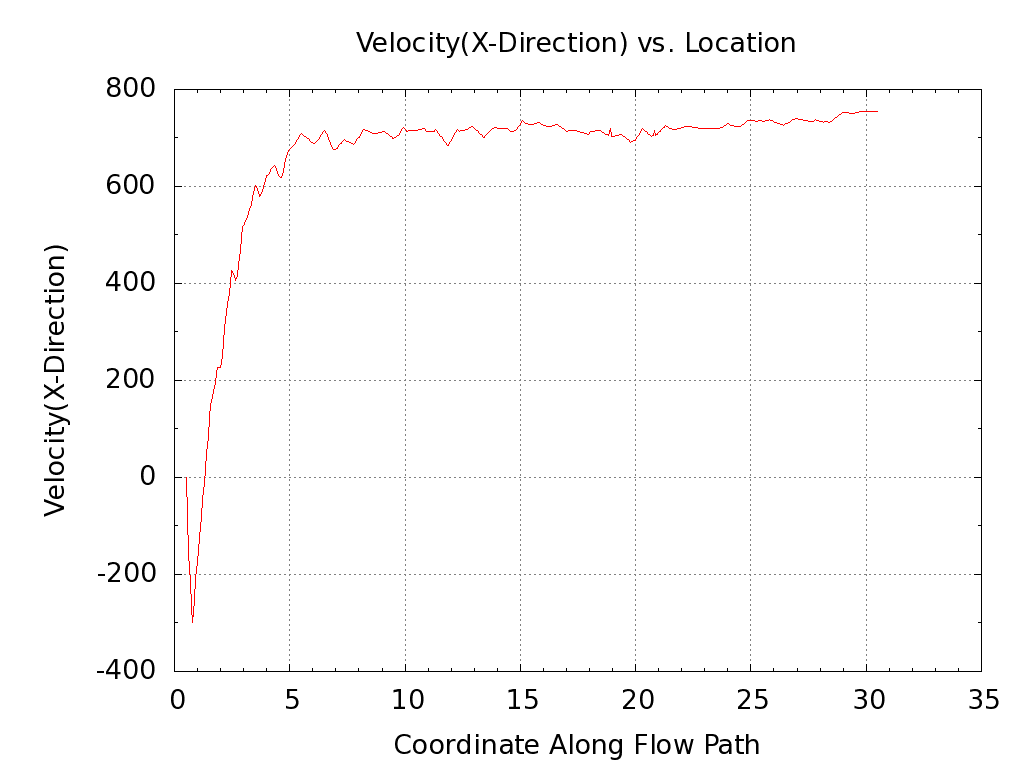

Supplement: S1 Images Folder — Image names are the column headings for and pertain to data in S1, S2, S4 and S5 Datasets. (ZIP) [file pone.0134978.s009.zip › S1_imagesfolder/BT3M2_C.png]

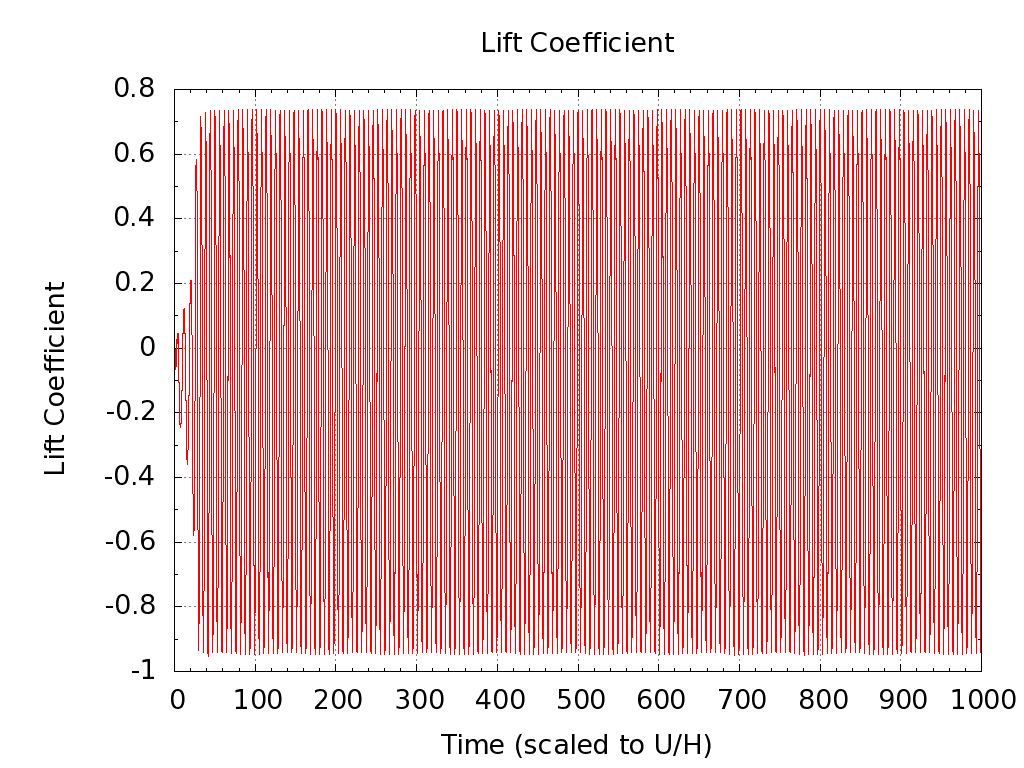

Supplement: S1 Images Folder — Image names are the column headings for and pertain to data in S1, S2, S4 and S5 Datasets. (ZIP) [file pone.0134978.s009.zip › S1_imagesfolder/BT3M2_D.png]

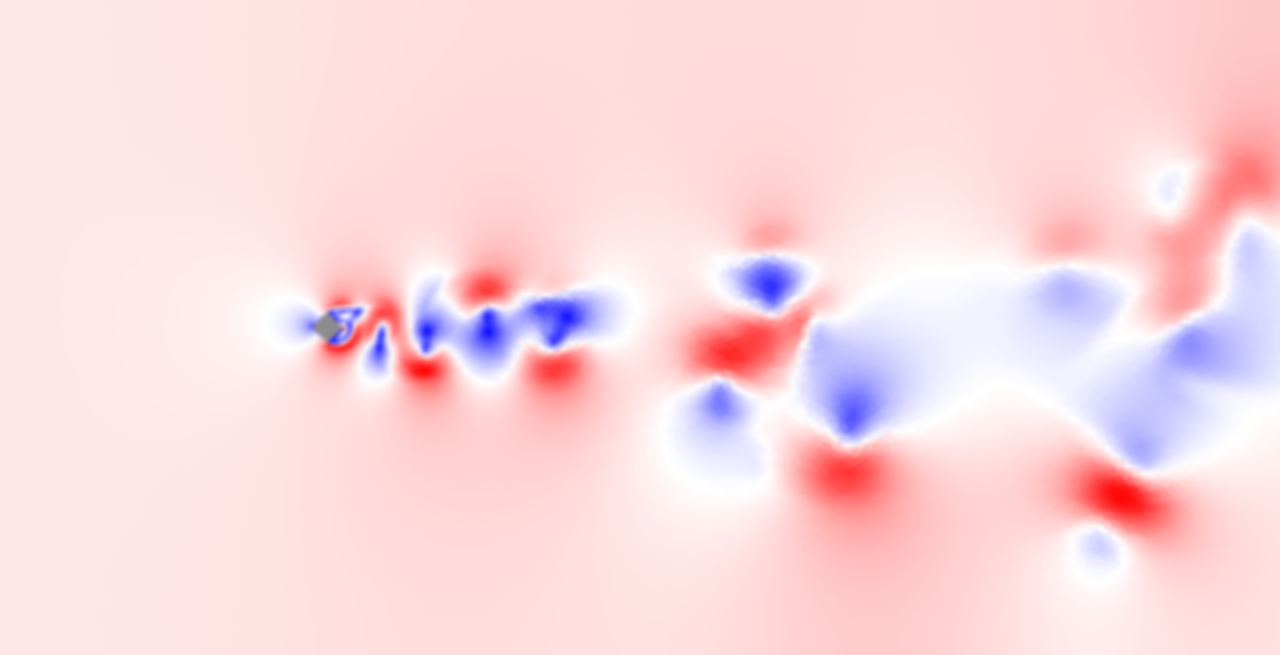

Supplement: S1 Images Folder — Image names are the column headings for and pertain to data in S1, S2, S4 and S5 Datasets. (ZIP) [file pone.0134978.s009.zip › S1_imagesfolder/BT3M3_B.png]

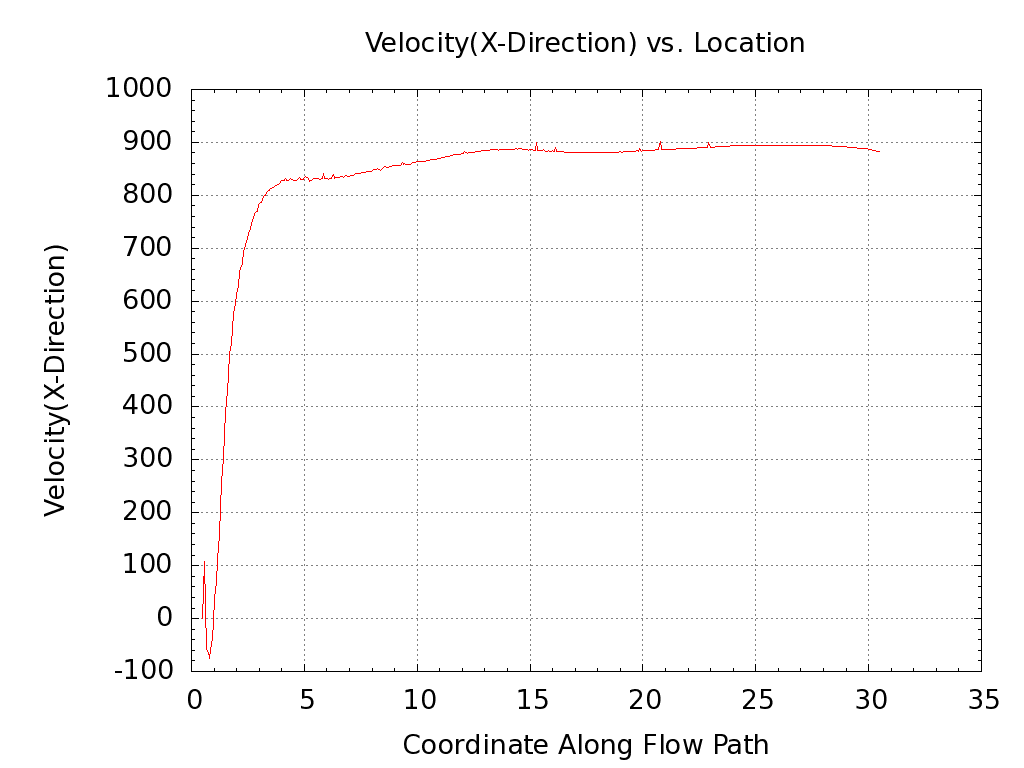

Supplement: S1 Images Folder — Image names are the column headings for and pertain to data in S1, S2, S4 and S5 Datasets. (ZIP) [file pone.0134978.s009.zip › S1_imagesfolder/BT3M3_C.png]

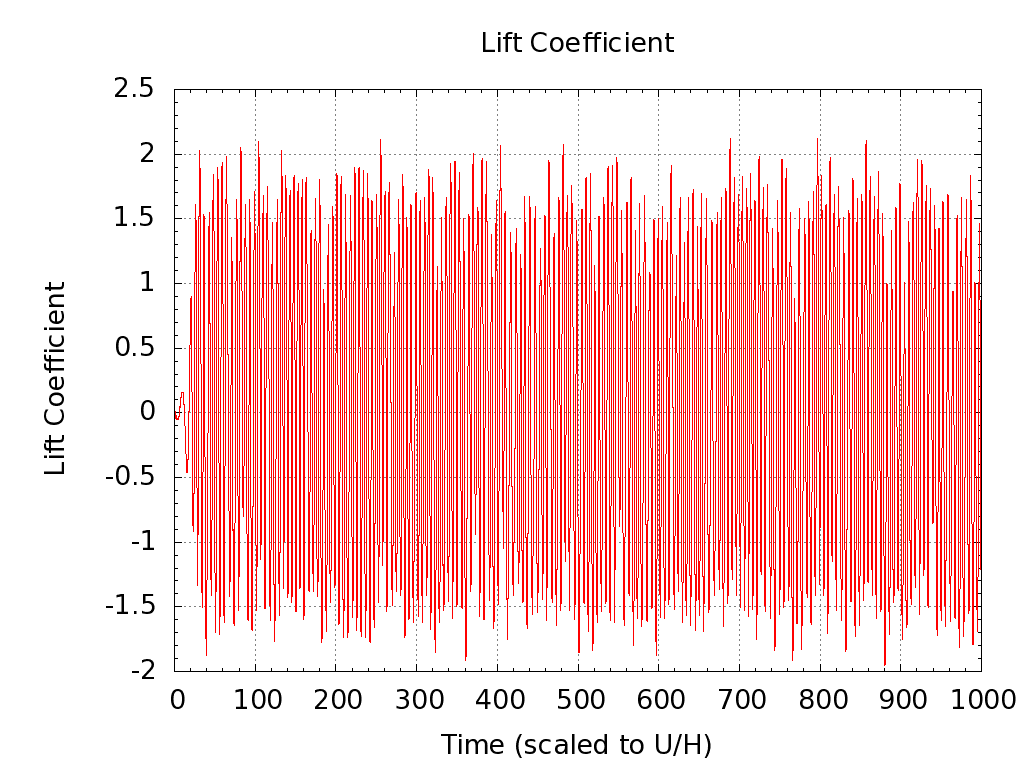

Supplement: S1 Images Folder — Image names are the column headings for and pertain to data in S1, S2, S4 and S5 Datasets. (ZIP) [file pone.0134978.s009.zip › S1_imagesfolder/BT3M3_D.png]

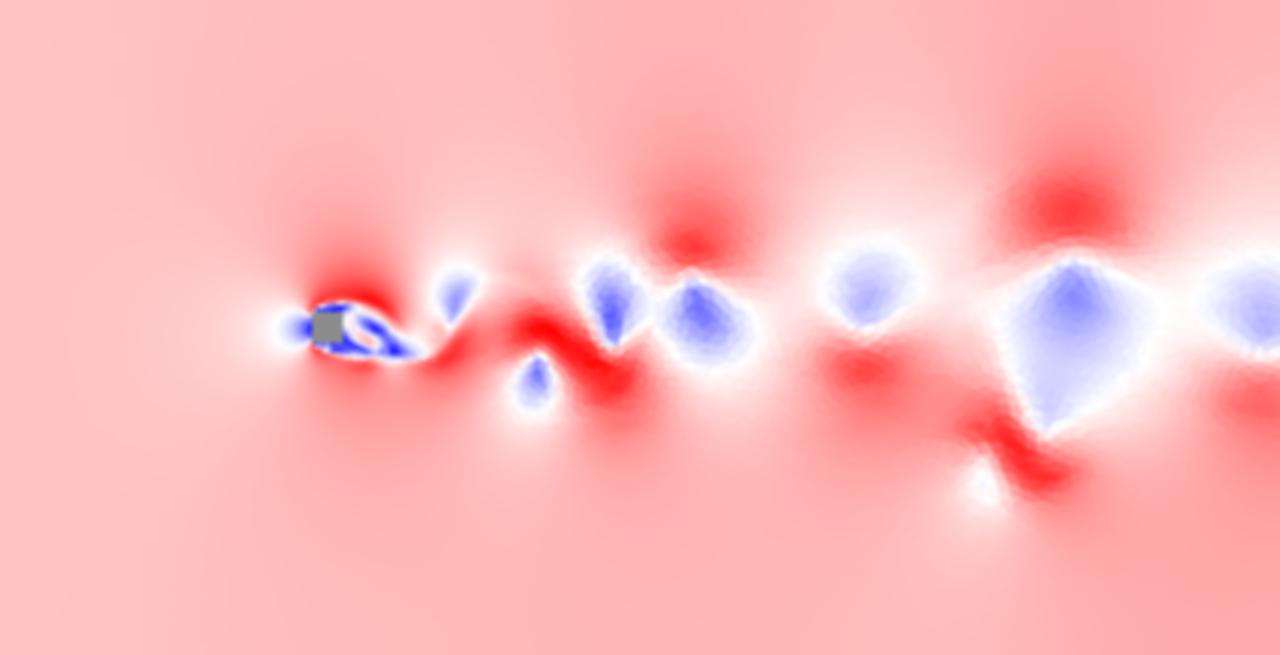

Supplement: S1 Images Folder — Image names are the column headings for and pertain to data in S1, S2, S4 and S5 Datasets. (ZIP) [file pone.0134978.s009.zip › S1_imagesfolder/BTurb1_B.png]

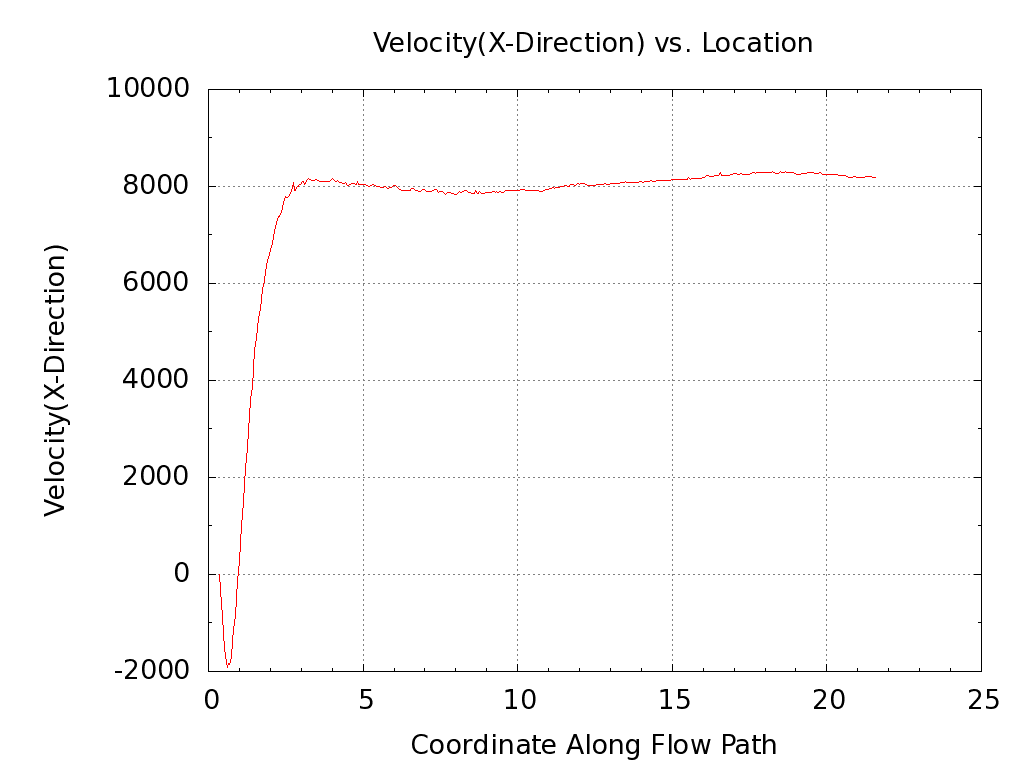

Supplement: S1 Images Folder — Image names are the column headings for and pertain to data in S1, S2, S4 and S5 Datasets. (ZIP) [file pone.0134978.s009.zip › S1_imagesfolder/BTurb1_C.png]

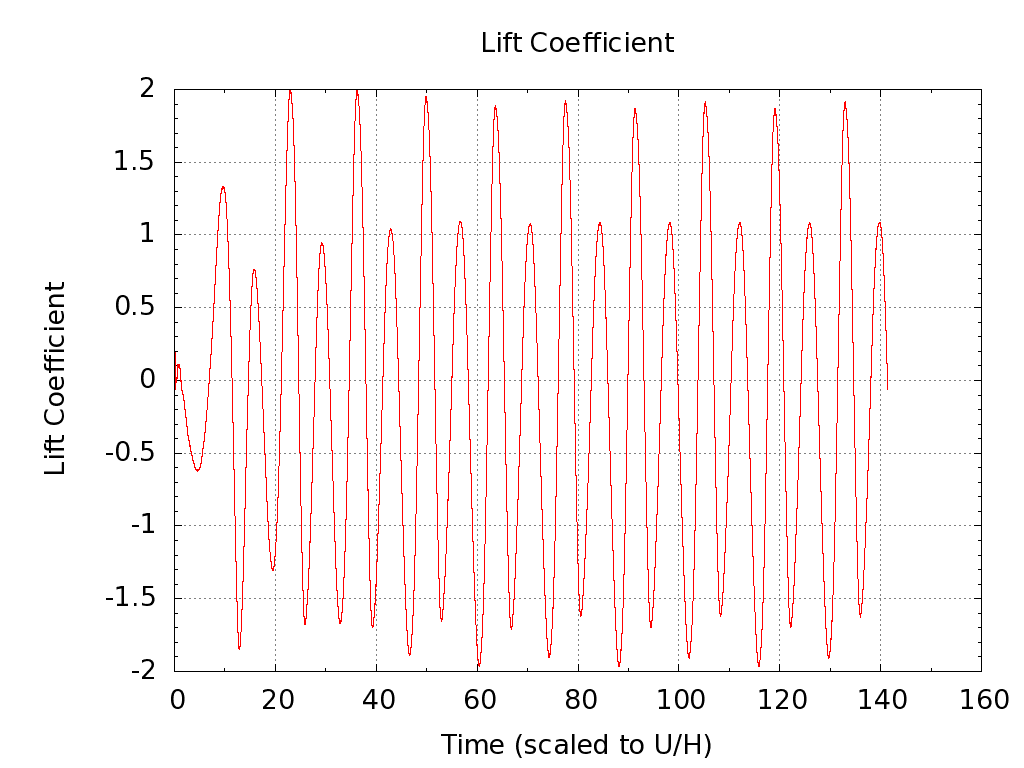

Supplement: S1 Images Folder — Image names are the column headings for and pertain to data in S1, S2, S4 and S5 Datasets. (ZIP) [file pone.0134978.s009.zip › S1_imagesfolder/BTurb1_D.png]

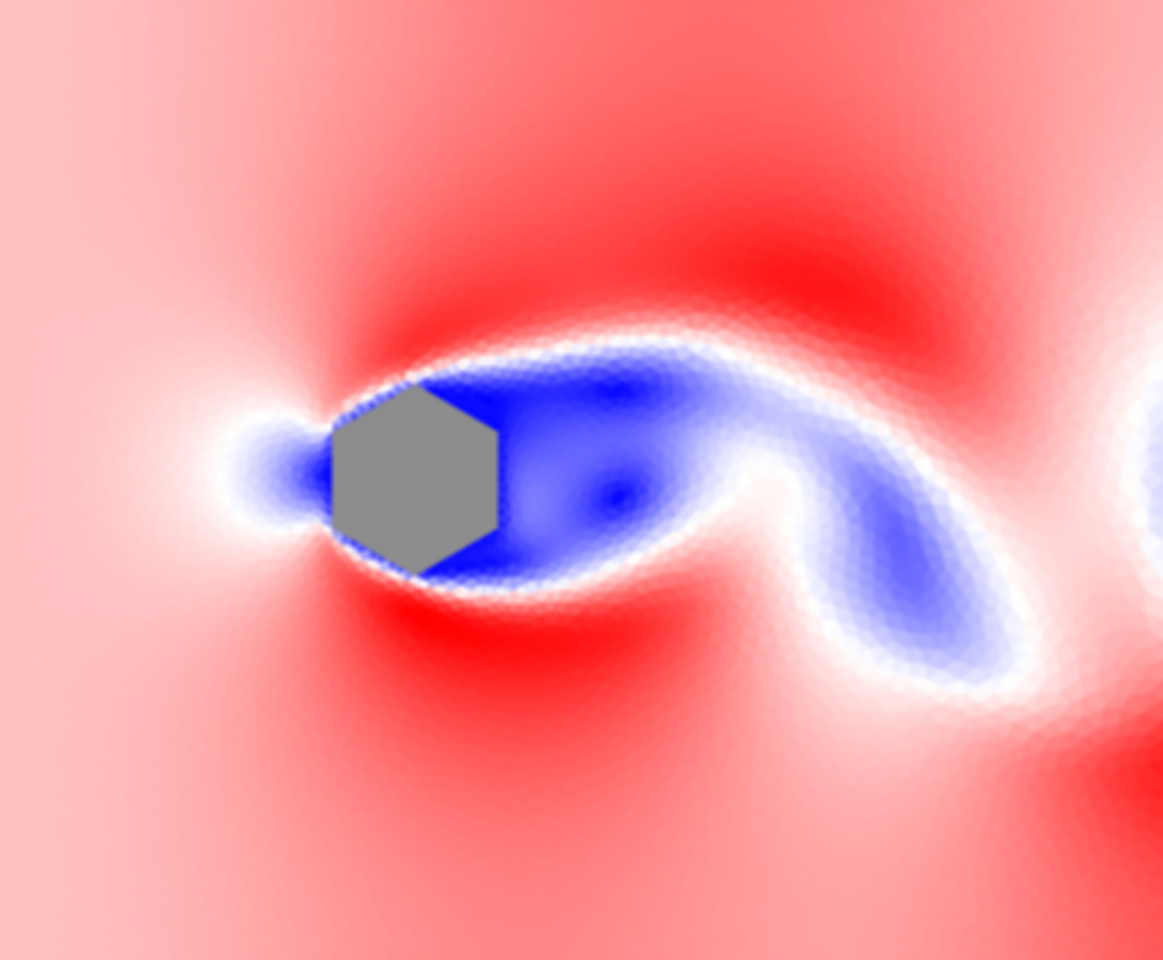

Supplement: S1 Images Folder — Image names are the column headings for and pertain to data in S1, S2, S4 and S5 Datasets. (ZIP) [file pone.0134978.s009.zip › S1_imagesfolder/CDom1_B.png]

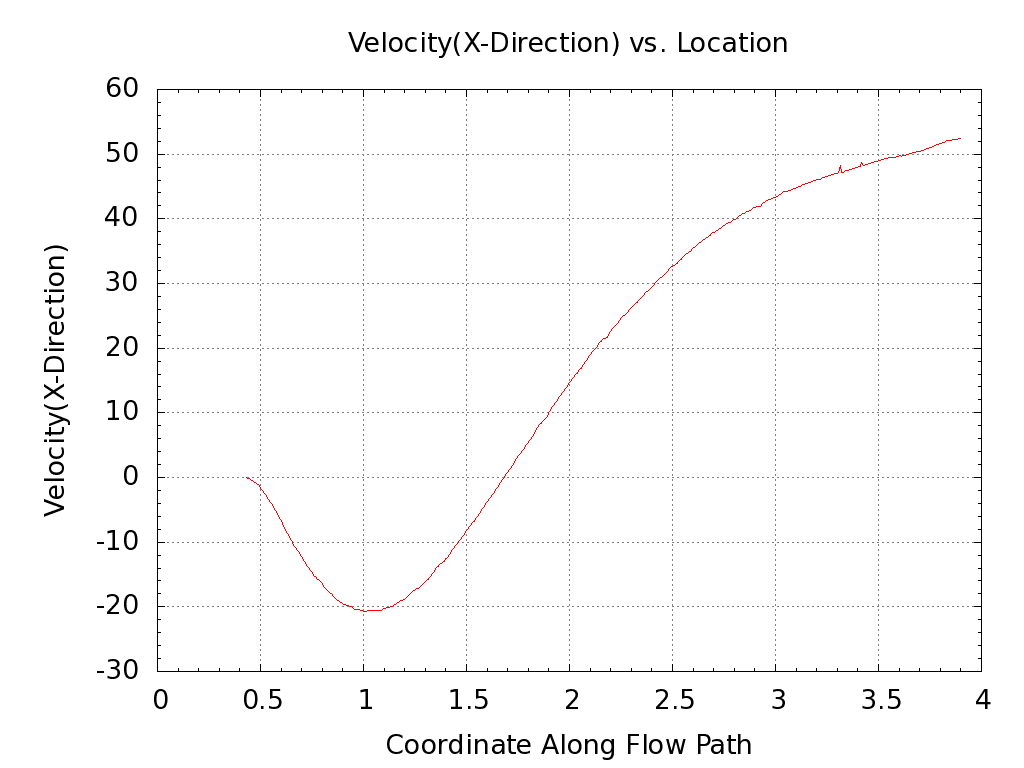

Supplement: S1 Images Folder — Image names are the column headings for and pertain to data in S1, S2, S4 and S5 Datasets. (ZIP) [file pone.0134978.s009.zip › S1_imagesfolder/CDom1_C.png]

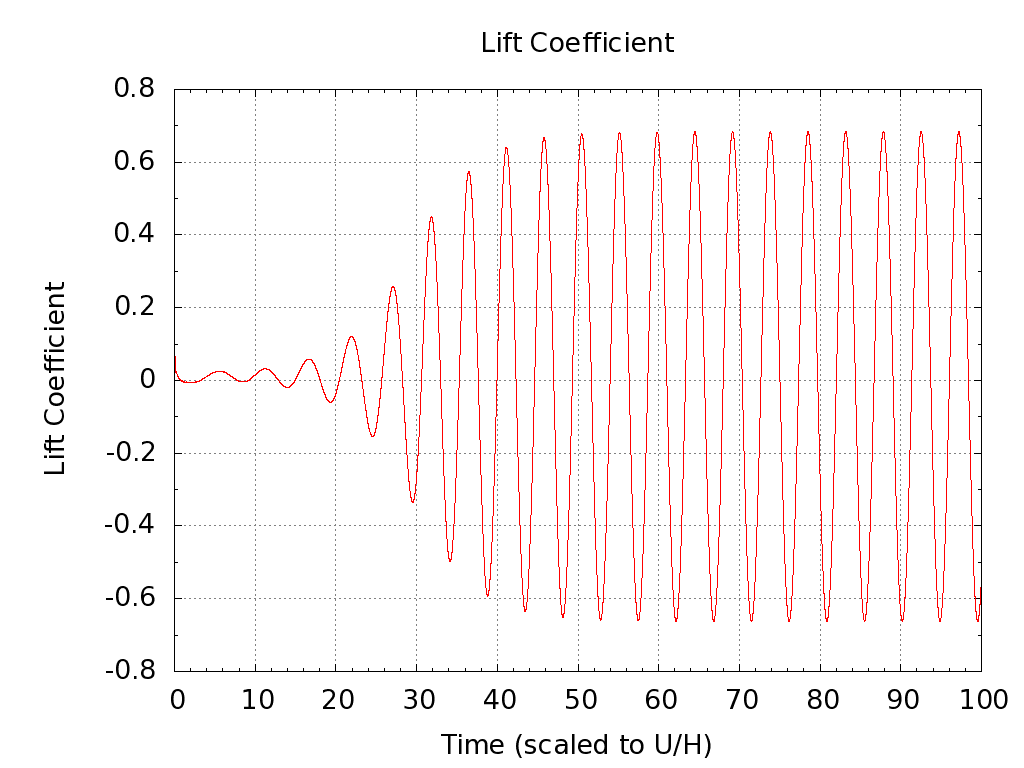

Supplement: S1 Images Folder — Image names are the column headings for and pertain to data in S1, S2, S4 and S5 Datasets. (ZIP) [file pone.0134978.s009.zip › S1_imagesfolder/CDom1_D.png]

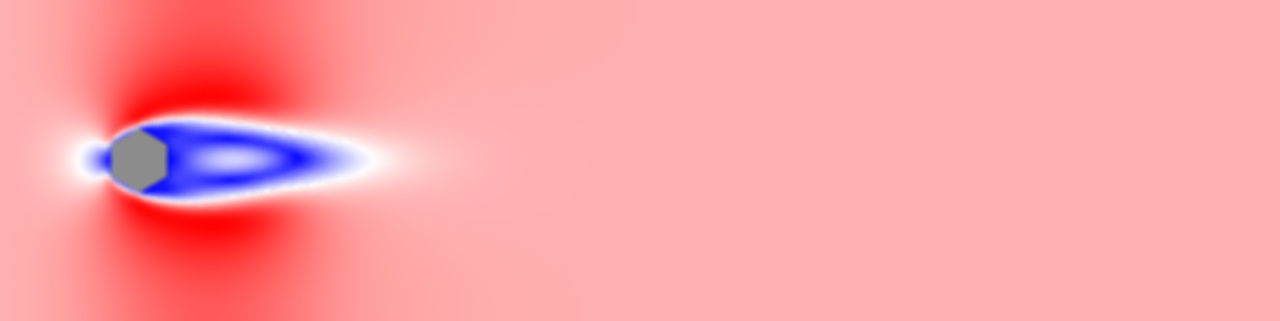

Supplement: S1 Images Folder — Image names are the column headings for and pertain to data in S1, S2, S4 and S5 Datasets. (ZIP) [file pone.0134978.s009.zip › S1_imagesfolder/CDur1_B.png]

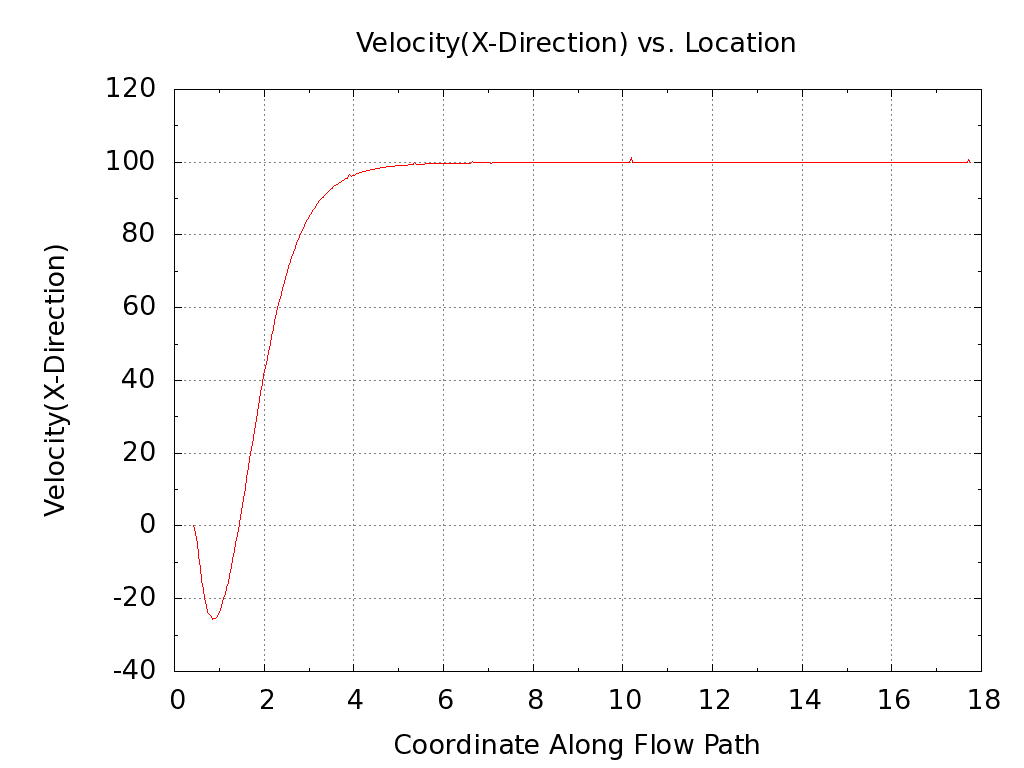

Supplement: S1 Images Folder — Image names are the column headings for and pertain to data in S1, S2, S4 and S5 Datasets. (ZIP) [file pone.0134978.s009.zip › S1_imagesfolder/CDur1_C.png]

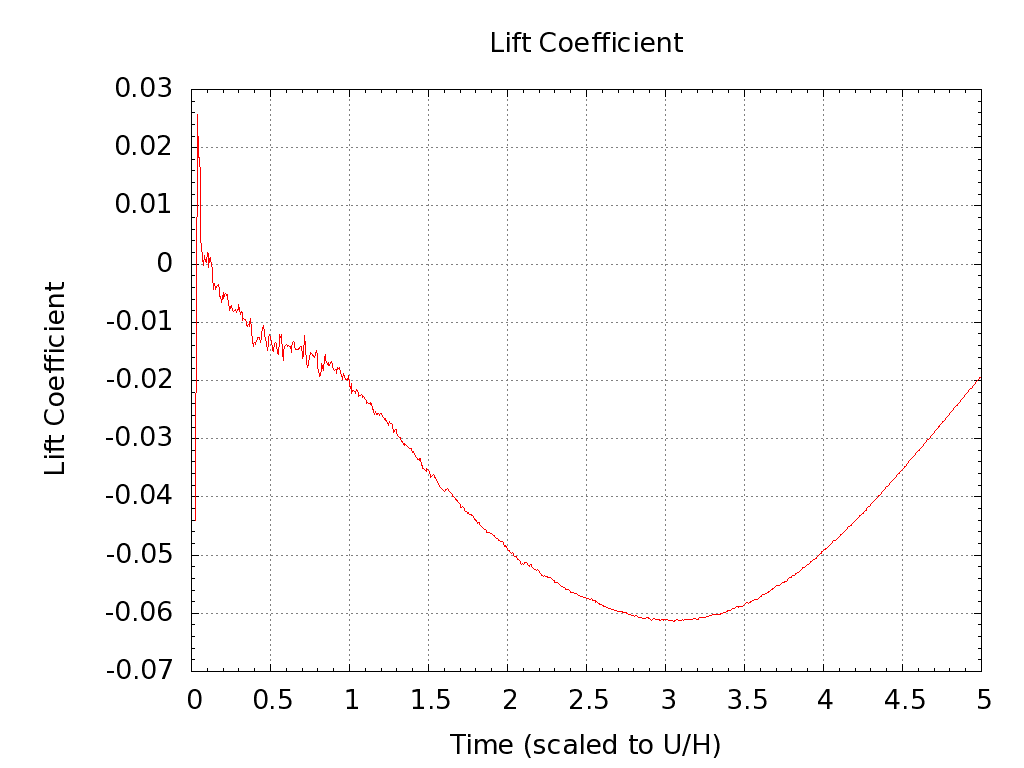

Supplement: S1 Images Folder — Image names are the column headings for and pertain to data in S1, S2, S4 and S5 Datasets. (ZIP) [file pone.0134978.s009.zip › S1_imagesfolder/CDur1_D.png]

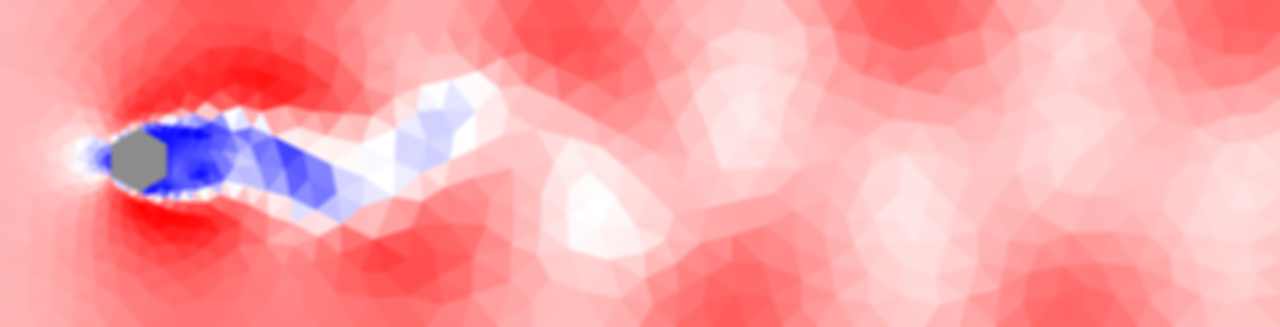

Supplement: S1 Images Folder — Image names are the column headings for and pertain to data in S1, S2, S4 and S5 Datasets. (ZIP) [file pone.0134978.s009.zip › S1_imagesfolder/CT1M1_B.png]

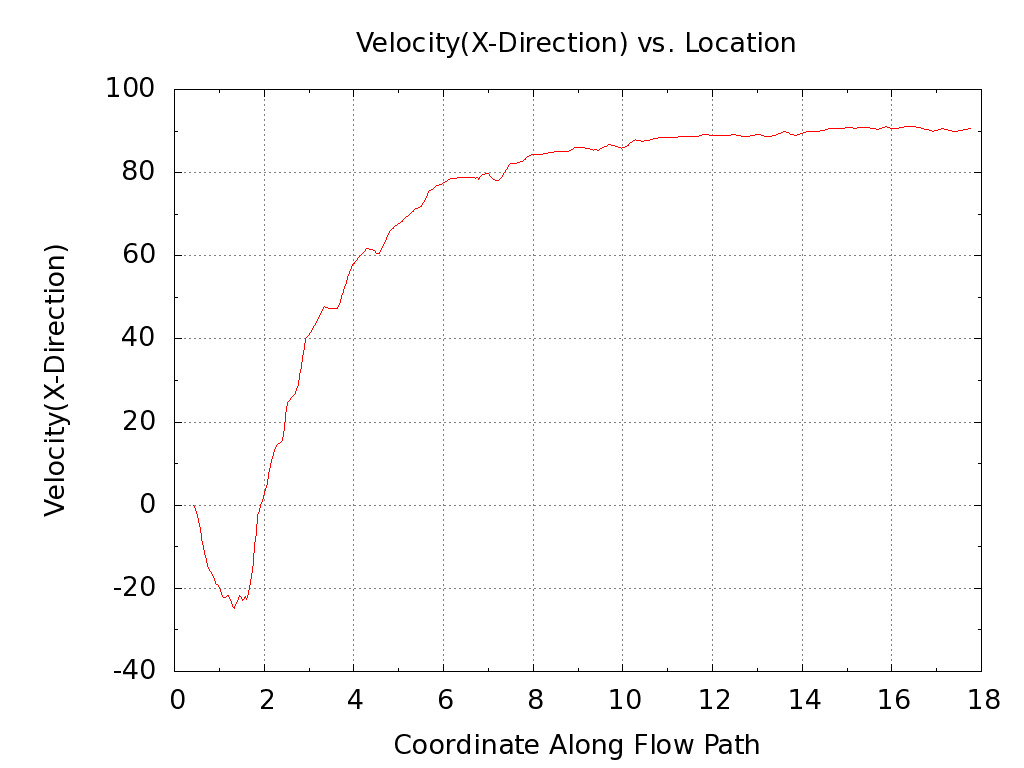

Supplement: S1 Images Folder — Image names are the column headings for and pertain to data in S1, S2, S4 and S5 Datasets. (ZIP) [file pone.0134978.s009.zip › S1_imagesfolder/CT1M1_C.png]

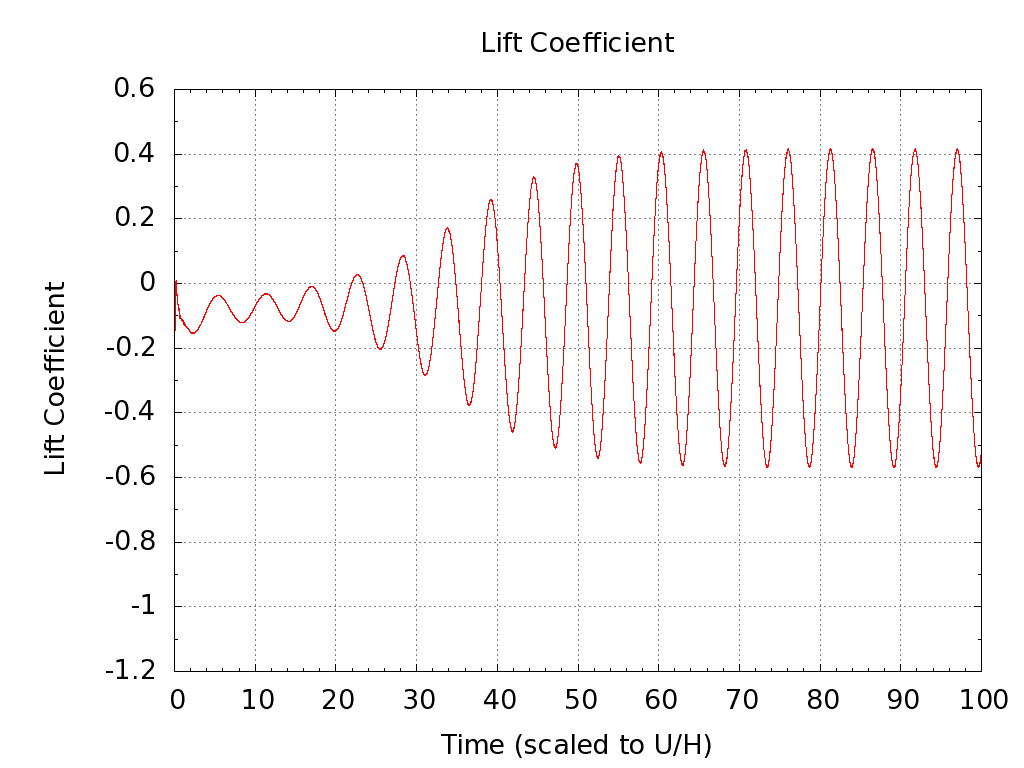

Supplement: S1 Images Folder — Image names are the column headings for and pertain to data in S1, S2, S4 and S5 Datasets. (ZIP) [file pone.0134978.s009.zip › S1_imagesfolder/CT1M1_D.png]

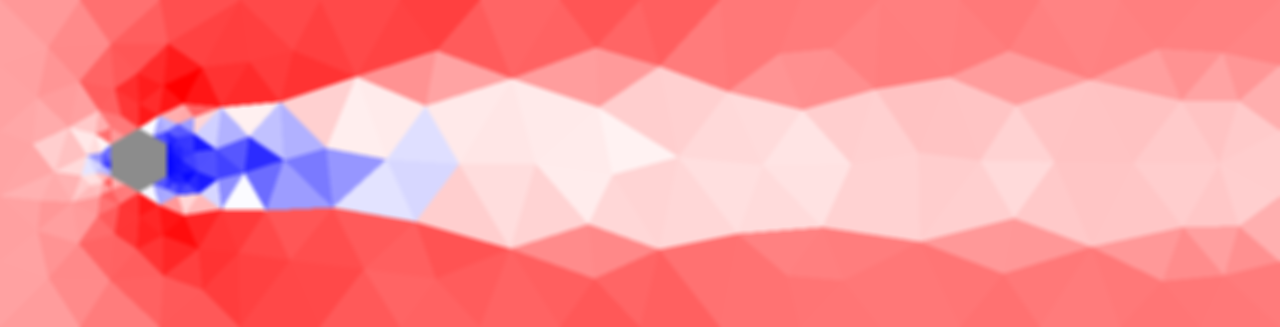

Supplement: S1 Images Folder — Image names are the column headings for and pertain to data in S1, S2, S4 and S5 Datasets. (ZIP) [file pone.0134978.s009.zip › S1_imagesfolder/CT1M2_B.png]

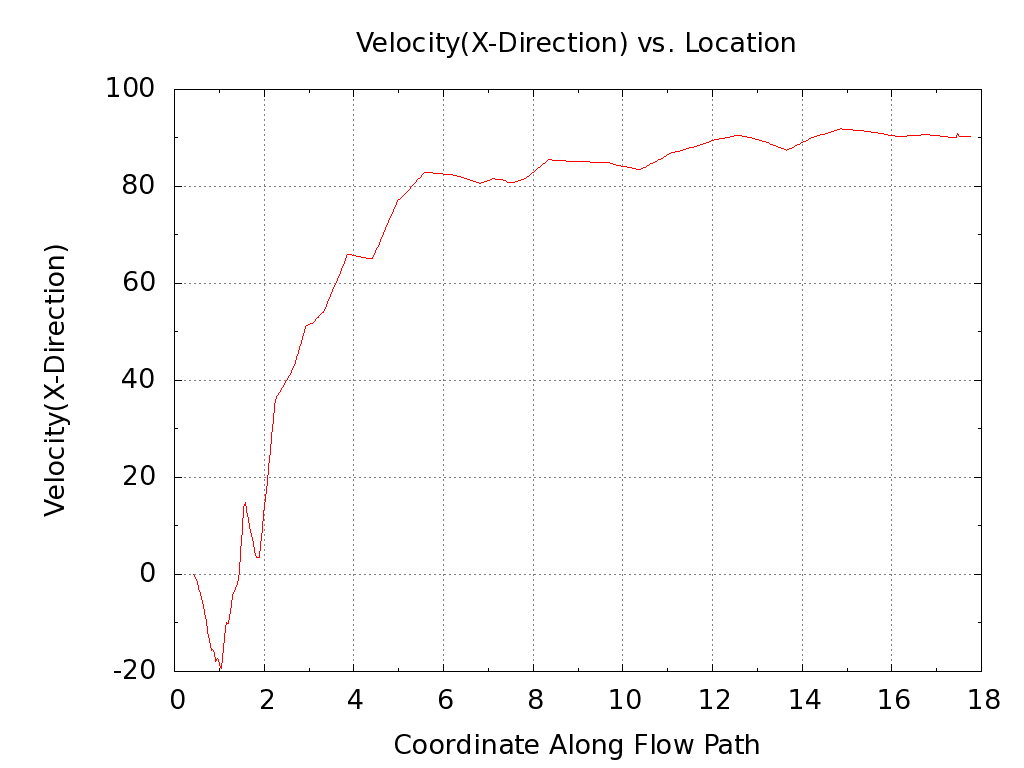

Supplement: S1 Images Folder — Image names are the column headings for and pertain to data in S1, S2, S4 and S5 Datasets. (ZIP) [file pone.0134978.s009.zip › S1_imagesfolder/CT1M2_C.png]

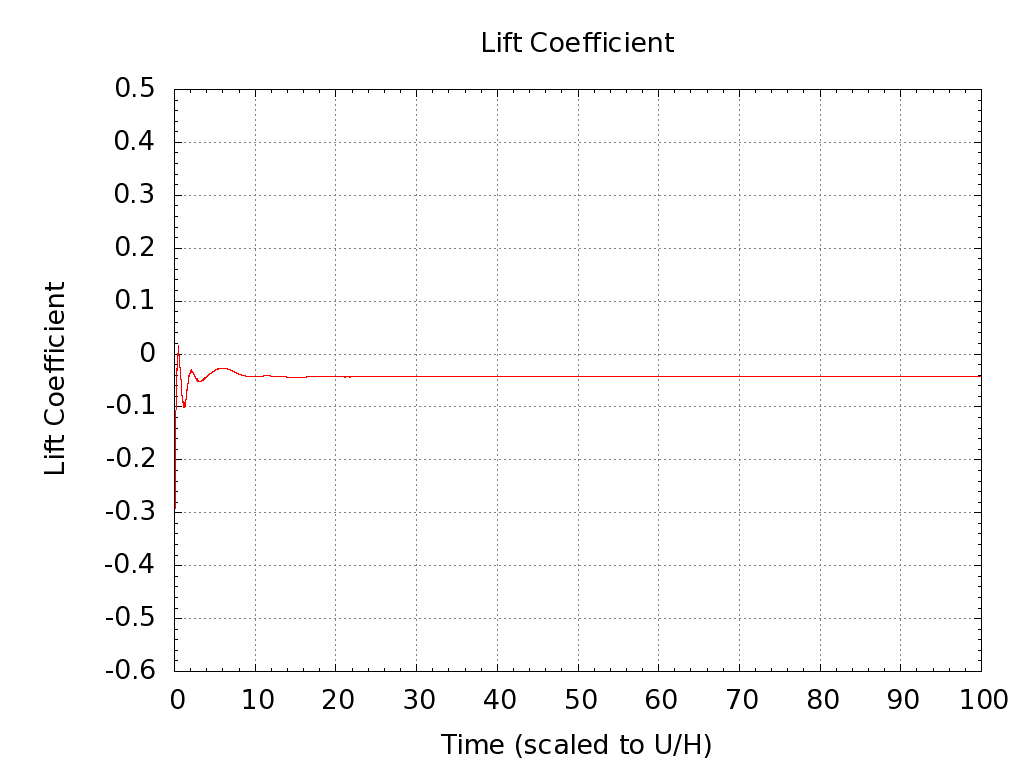

Supplement: S1 Images Folder — Image names are the column headings for and pertain to data in S1, S2, S4 and S5 Datasets. (ZIP) [file pone.0134978.s009.zip › S1_imagesfolder/CT1M2_D.png]

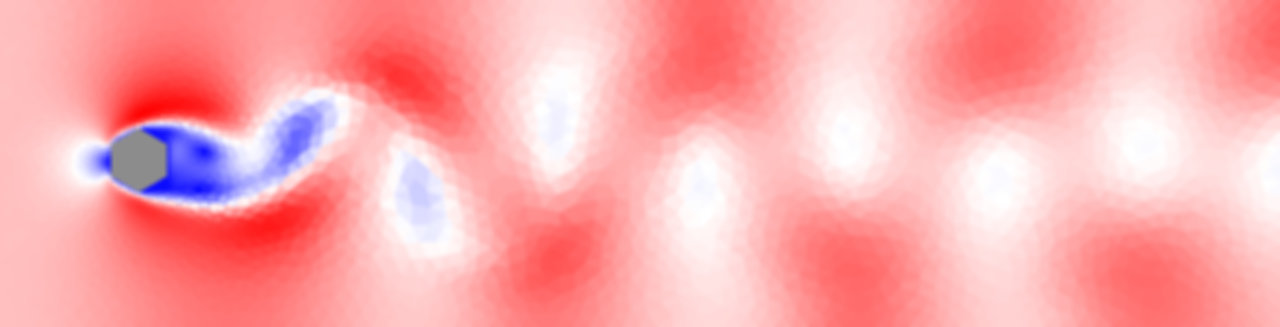

Supplement: S1 Images Folder — Image names are the column headings for and pertain to data in S1, S2, S4 and S5 Datasets. (ZIP) [file pone.0134978.s009.zip › S1_imagesfolder/CT1M3_B.png]

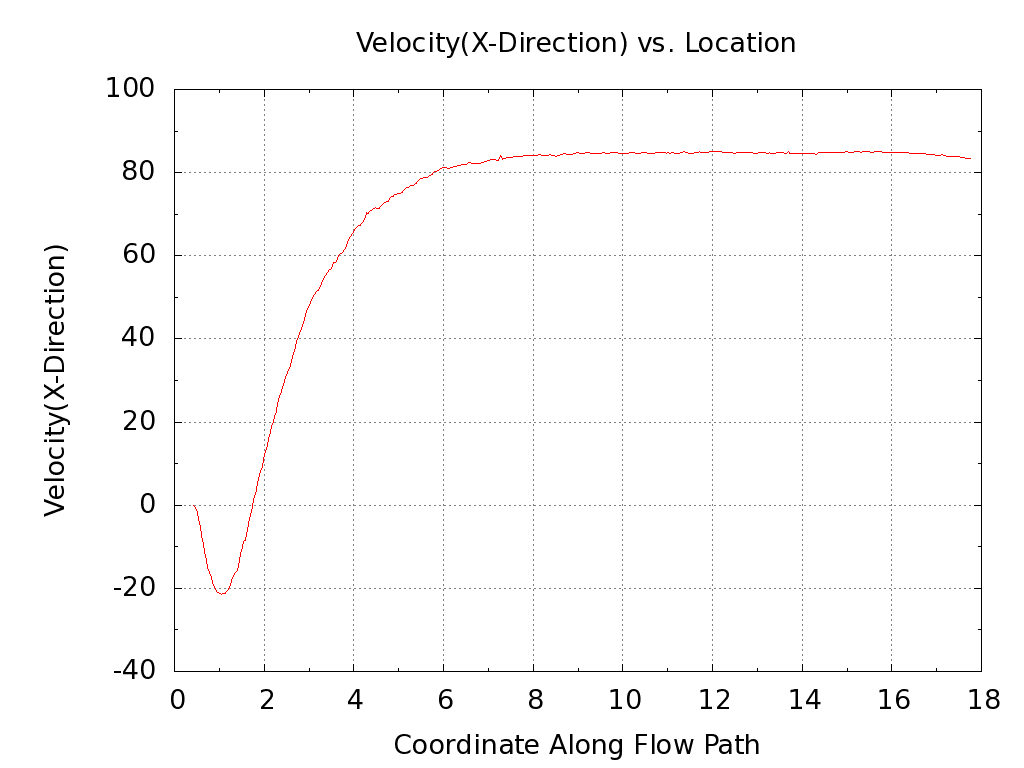

Supplement: S1 Images Folder — Image names are the column headings for and pertain to data in S1, S2, S4 and S5 Datasets. (ZIP) [file pone.0134978.s009.zip › S1_imagesfolder/CT1M3_C.png]

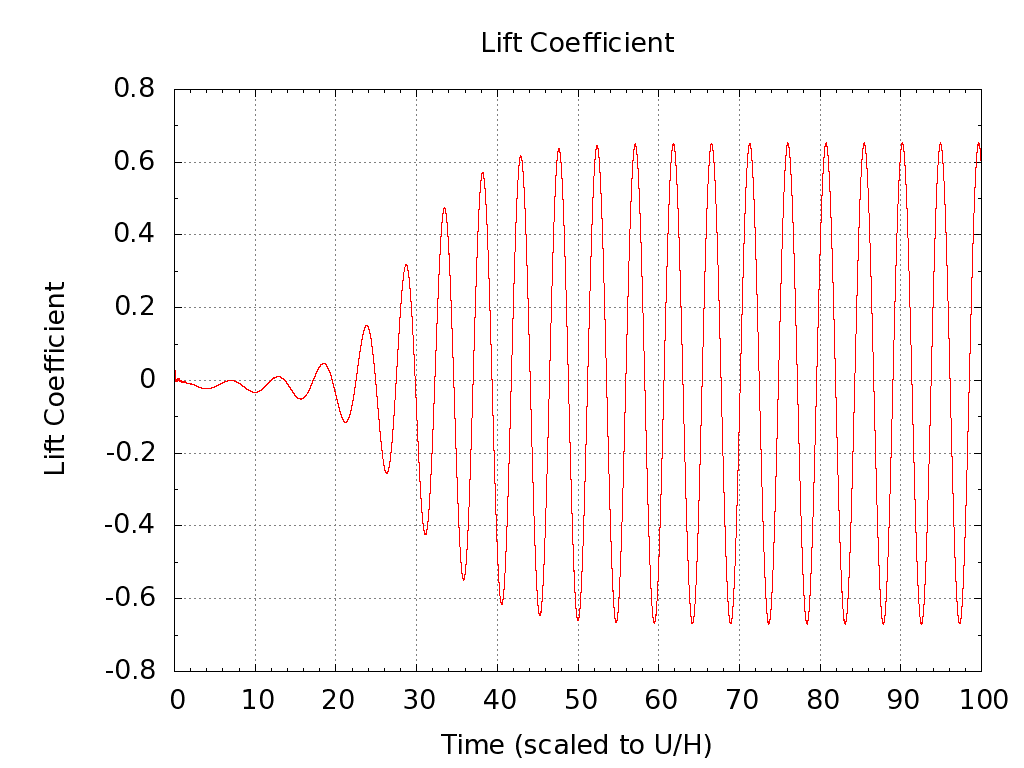

Supplement: S1 Images Folder — Image names are the column headings for and pertain to data in S1, S2, S4 and S5 Datasets. (ZIP) [file pone.0134978.s009.zip › S1_imagesfolder/CT1M3_D.png]

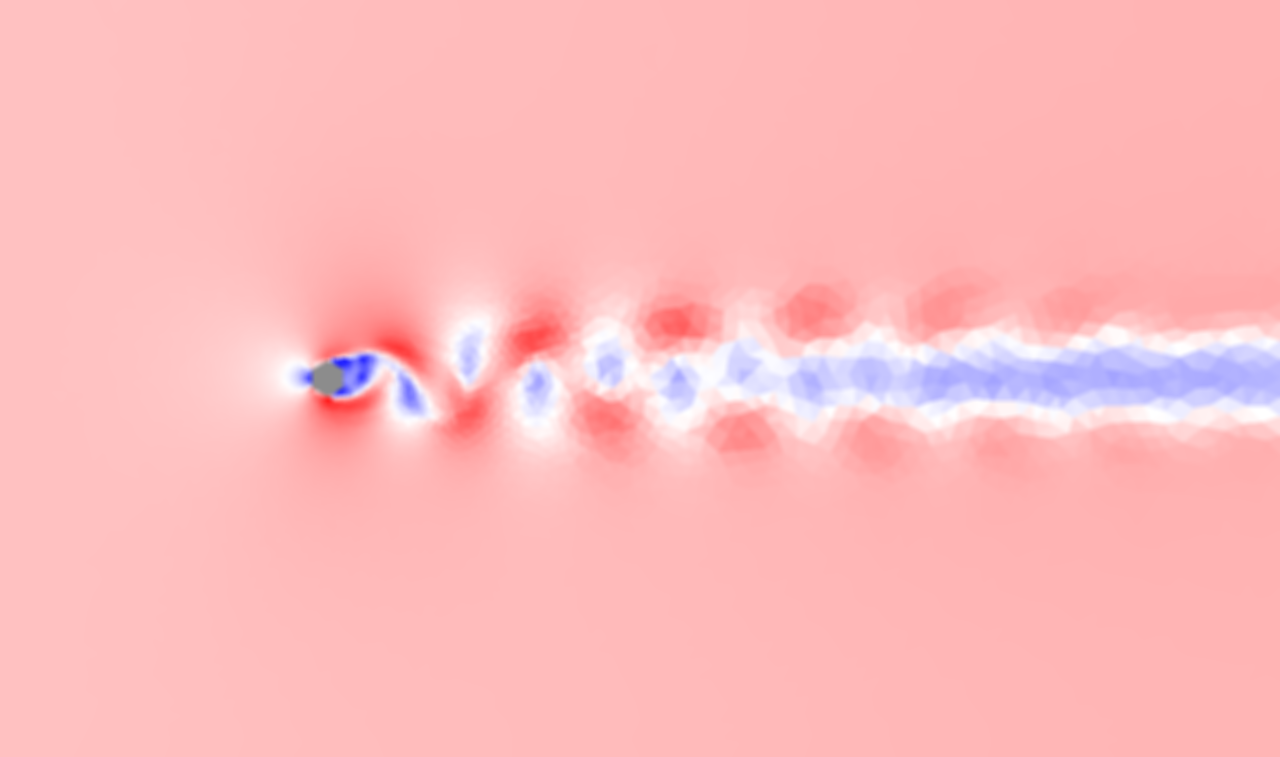

Supplement: S1 Images Folder — Image names are the column headings for and pertain to data in S1, S2, S4 and S5 Datasets. (ZIP) [file pone.0134978.s009.zip › S1_imagesfolder/CT2M1_B.png]

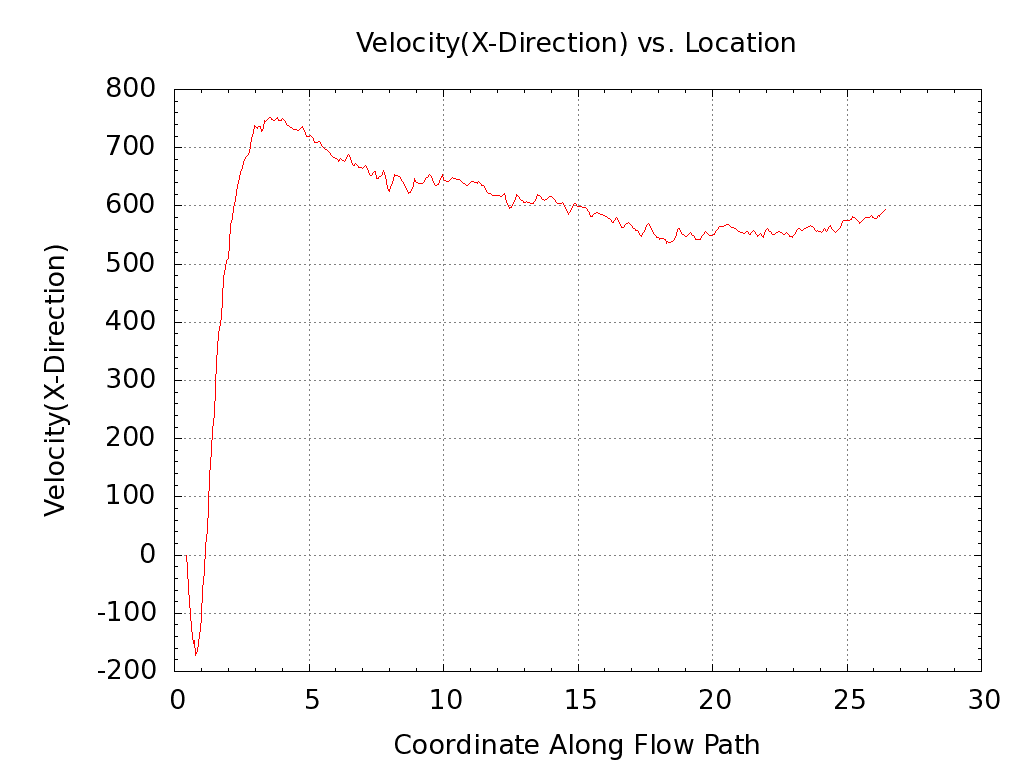

Supplement: S1 Images Folder — Image names are the column headings for and pertain to data in S1, S2, S4 and S5 Datasets. (ZIP) [file pone.0134978.s009.zip › S1_imagesfolder/CT2M1_C.png]

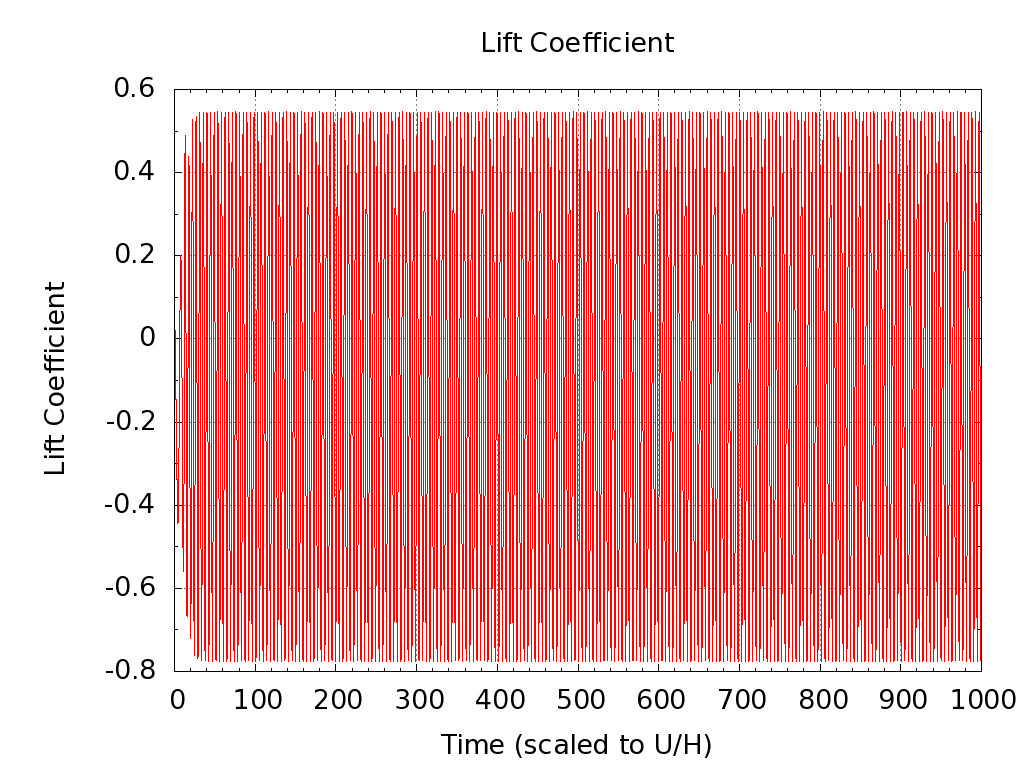

Supplement: S1 Images Folder — Image names are the column headings for and pertain to data in S1, S2, S4 and S5 Datasets. (ZIP) [file pone.0134978.s009.zip › S1_imagesfolder/CT2M1_D.png]

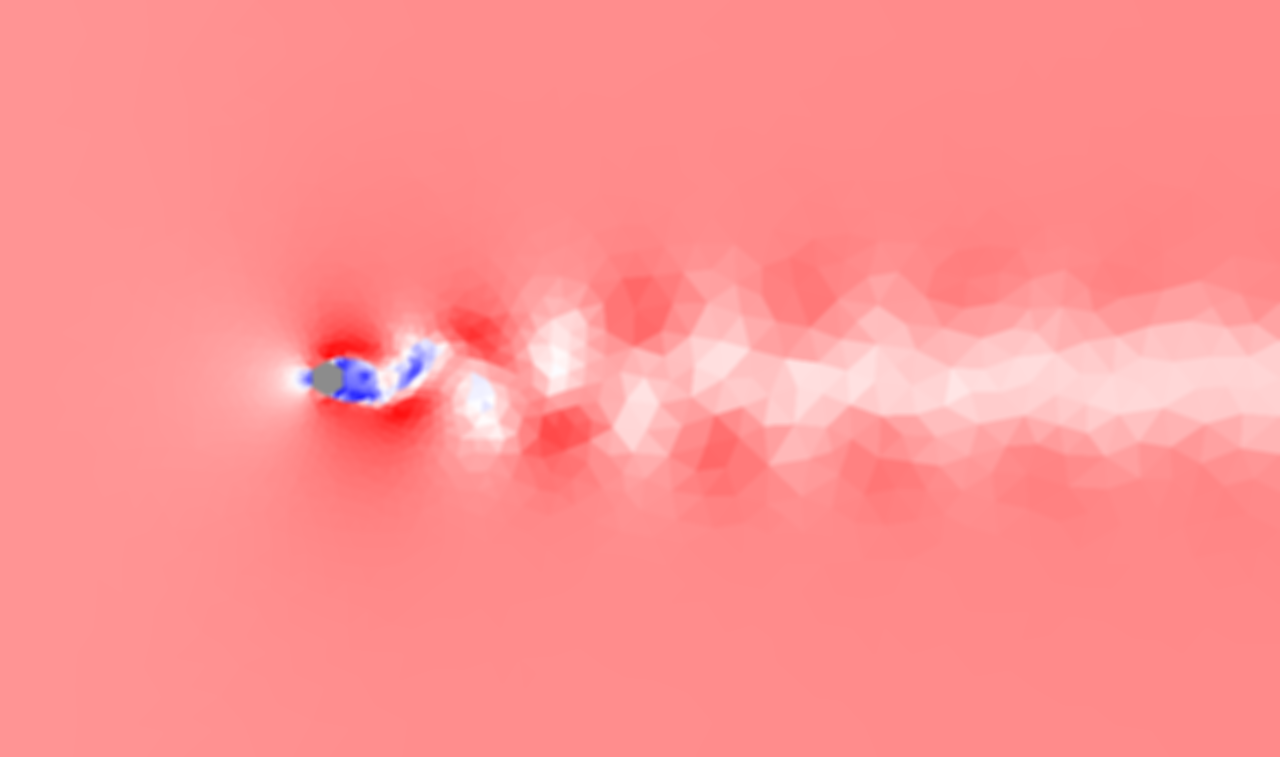

Supplement: S1 Images Folder — Image names are the column headings for and pertain to data in S1, S2, S4 and S5 Datasets. (ZIP) [file pone.0134978.s009.zip › S1_imagesfolder/CT2M2_B.png]

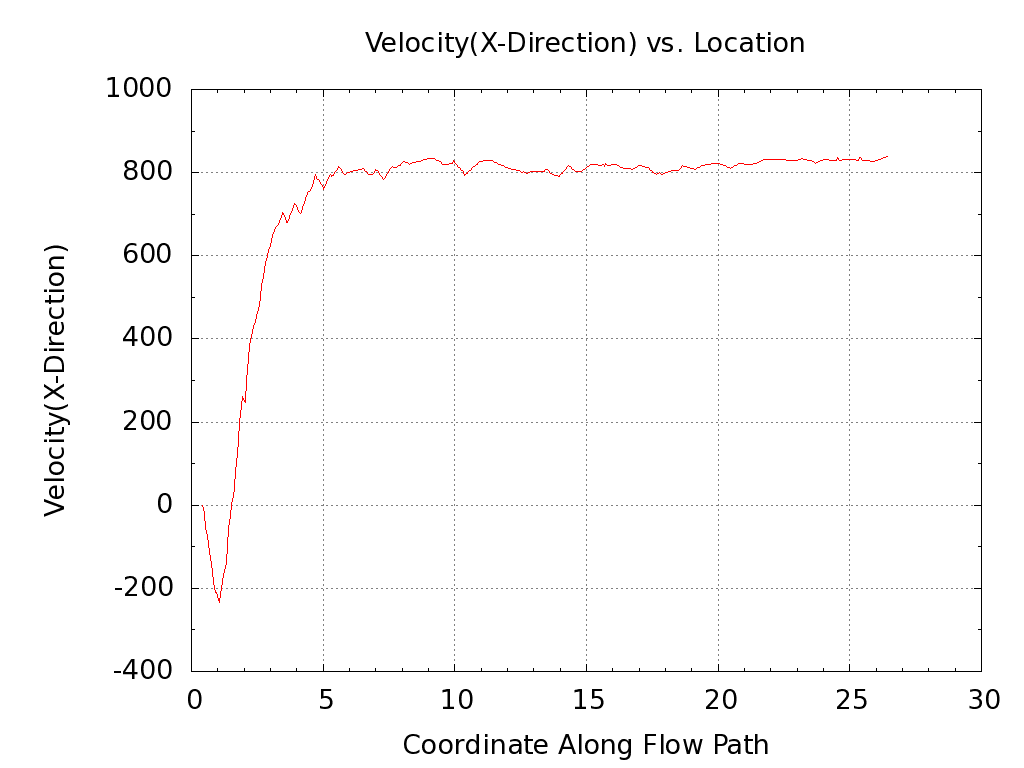

Supplement: S1 Images Folder — Image names are the column headings for and pertain to data in S1, S2, S4 and S5 Datasets. (ZIP) [file pone.0134978.s009.zip › S1_imagesfolder/CT2M2_C.png]

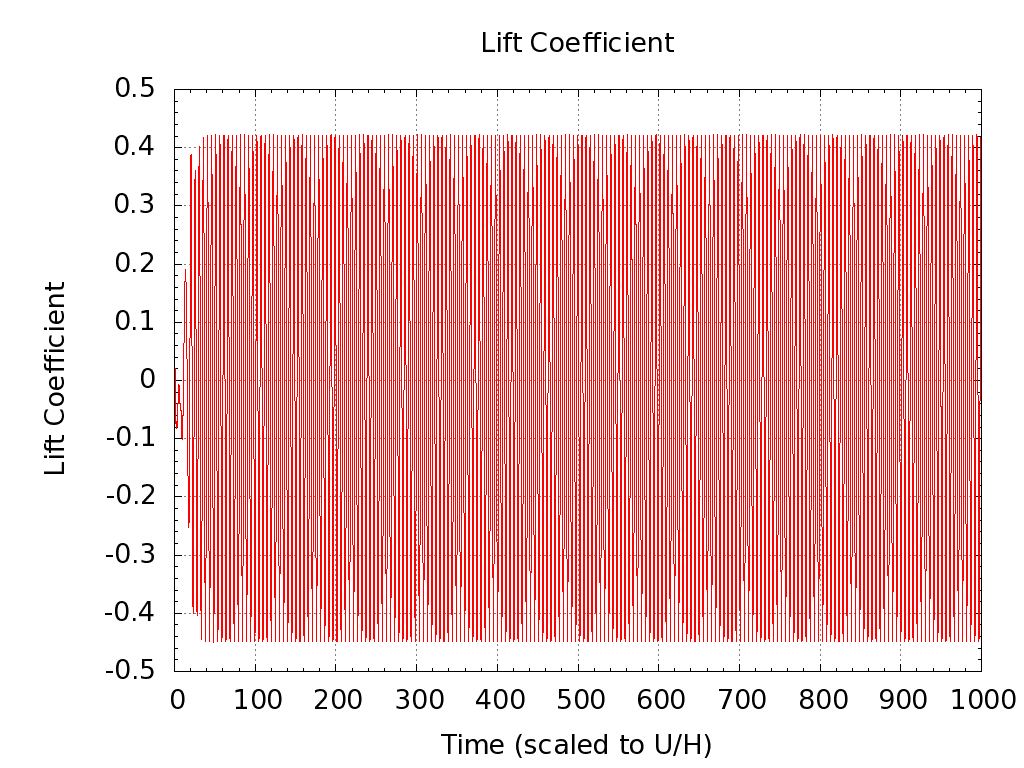

Supplement: S1 Images Folder — Image names are the column headings for and pertain to data in S1, S2, S4 and S5 Datasets. (ZIP) [file pone.0134978.s009.zip › S1_imagesfolder/CT2M2_D.png]

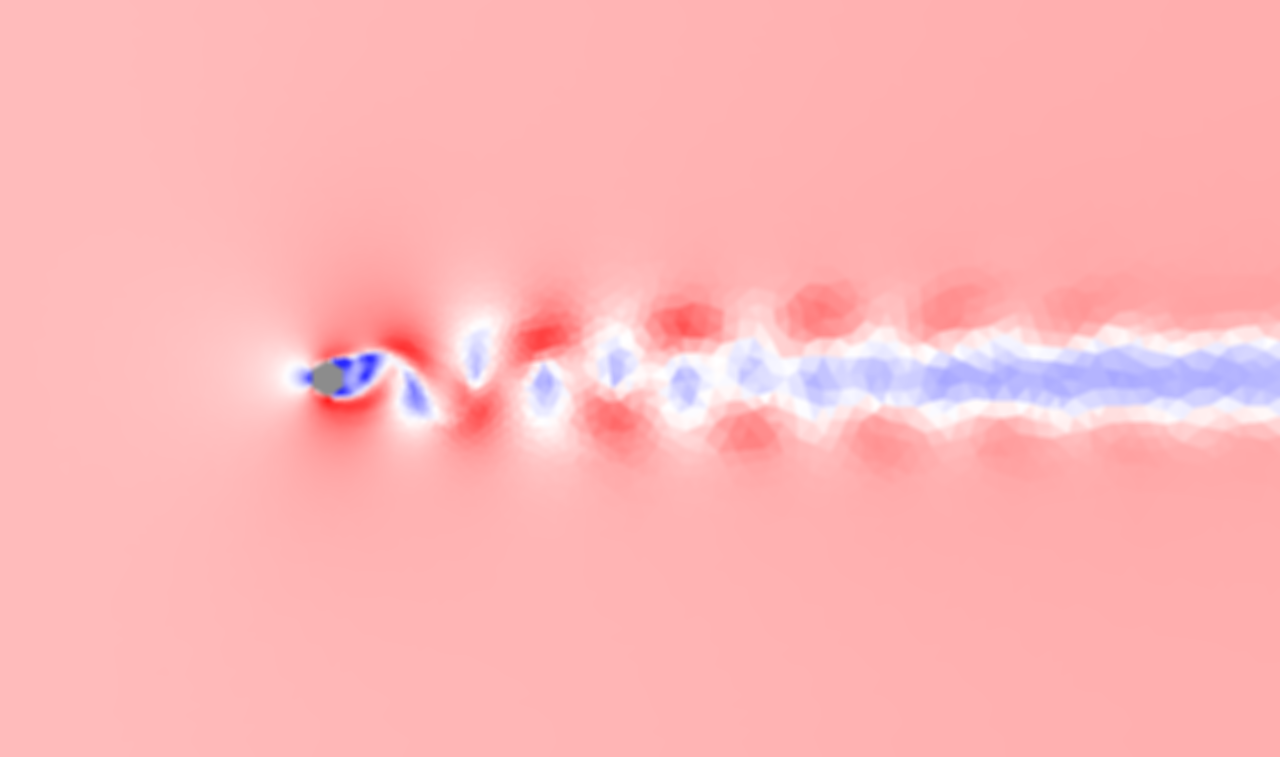

Supplement: S1 Images Folder — Image names are the column headings for and pertain to data in S1, S2, S4 and S5 Datasets. (ZIP) [file pone.0134978.s009.zip › S1_imagesfolder/CT3M1_B.png]

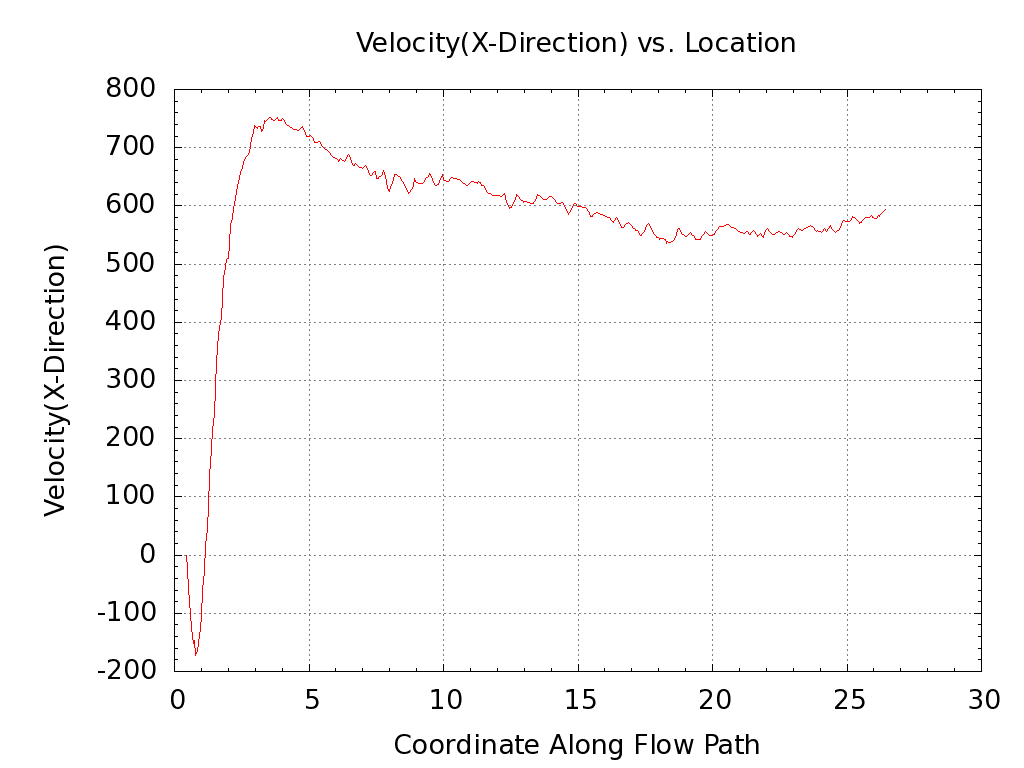

Supplement: S1 Images Folder — Image names are the column headings for and pertain to data in S1, S2, S4 and S5 Datasets. (ZIP) [file pone.0134978.s009.zip › S1_imagesfolder/CT3M1_C.png]

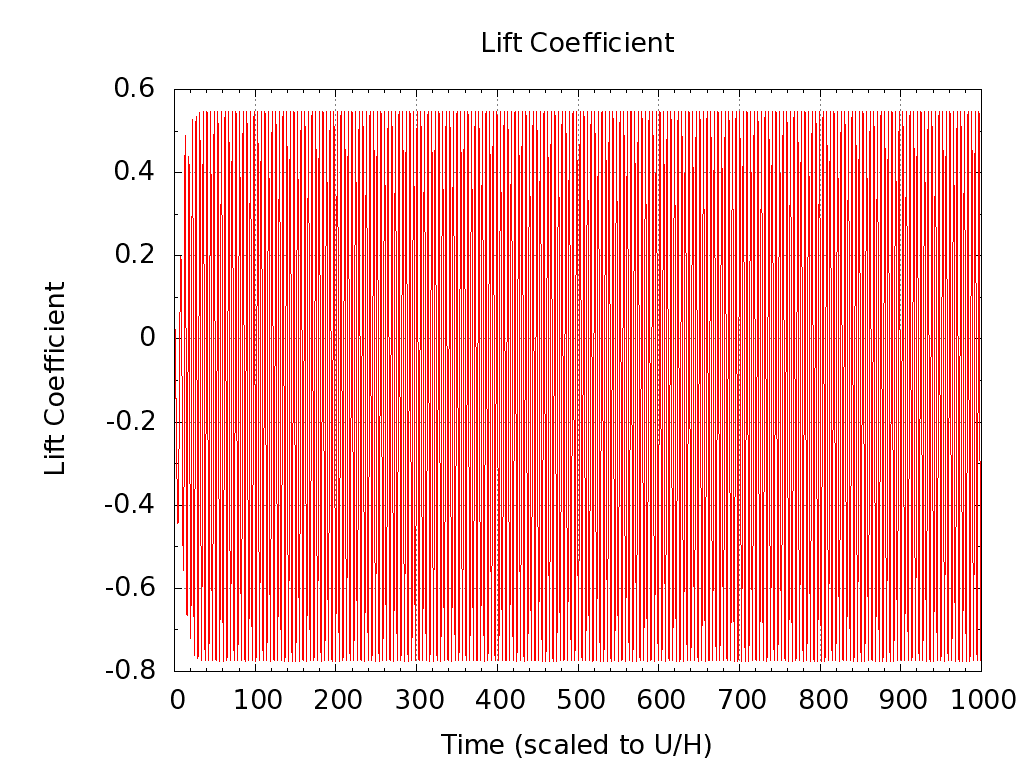

Supplement: S1 Images Folder — Image names are the column headings for and pertain to data in S1, S2, S4 and S5 Datasets. (ZIP) [file pone.0134978.s009.zip › S1_imagesfolder/CT3M1_D.png]

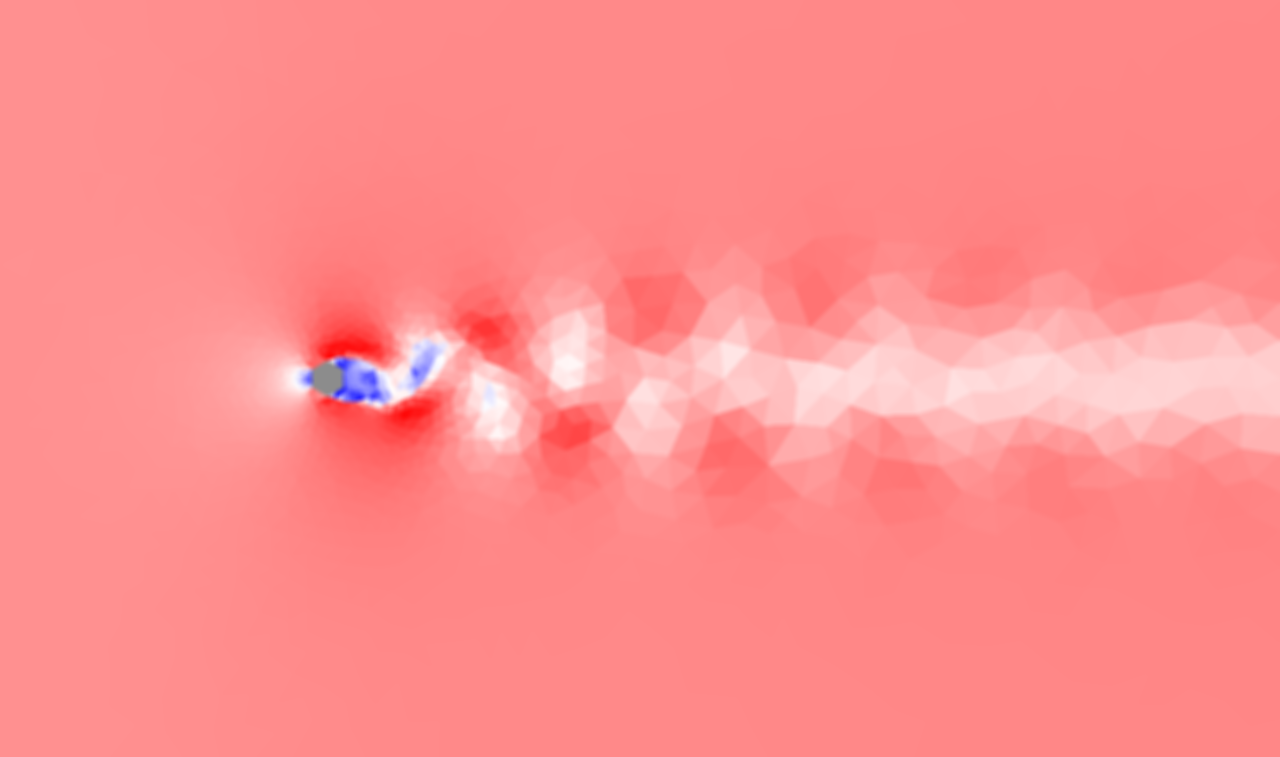

Supplement: S1 Images Folder — Image names are the column headings for and pertain to data in S1, S2, S4 and S5 Datasets. (ZIP) [file pone.0134978.s009.zip › S1_imagesfolder/CT3M2_B.png]

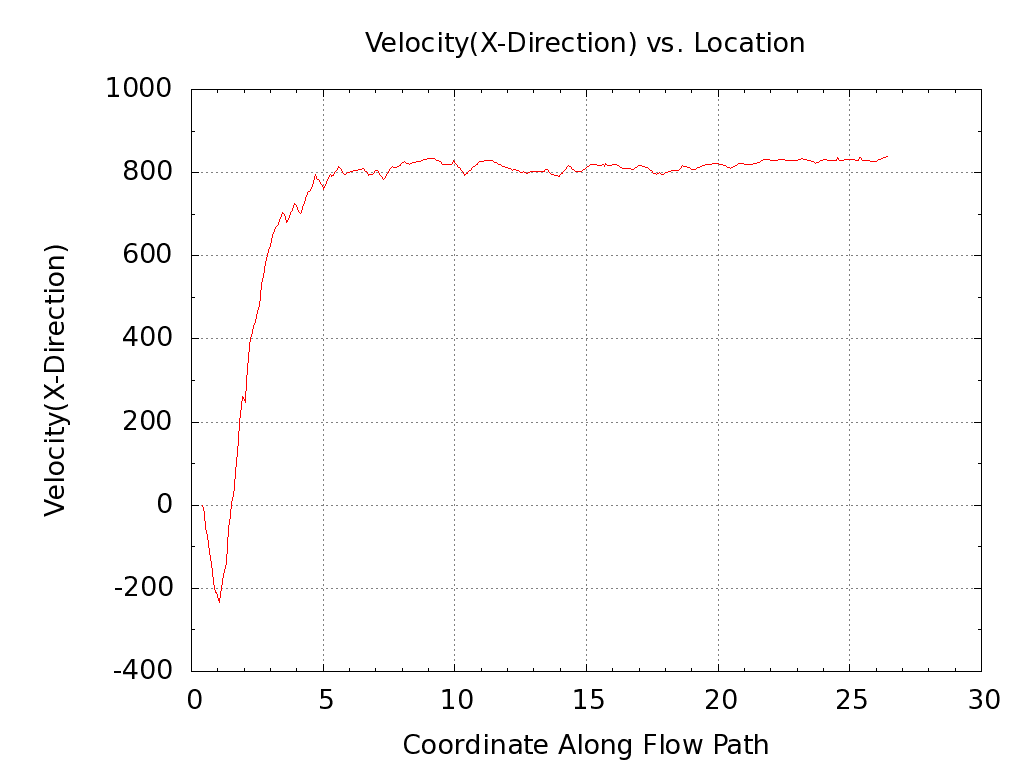

Supplement: S1 Images Folder — Image names are the column headings for and pertain to data in S1, S2, S4 and S5 Datasets. (ZIP) [file pone.0134978.s009.zip › S1_imagesfolder/CT3M2_C.png]

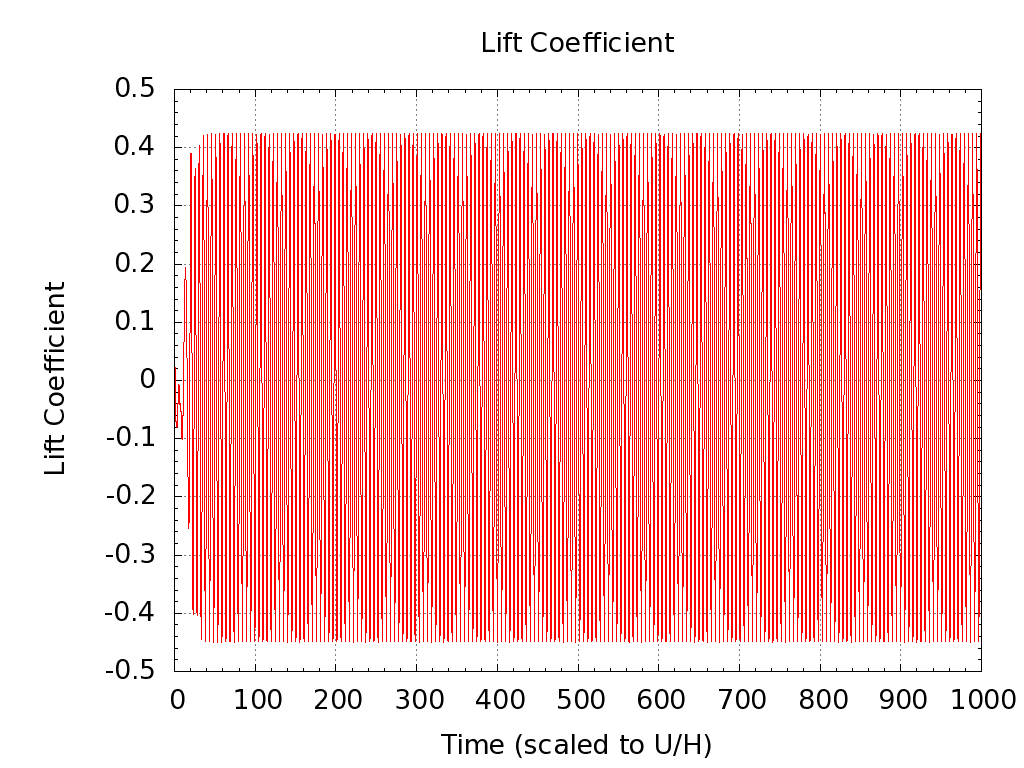

Supplement: S1 Images Folder — Image names are the column headings for and pertain to data in S1, S2, S4 and S5 Datasets. (ZIP) [file pone.0134978.s009.zip › S1_imagesfolder/CT3M2_D.png]

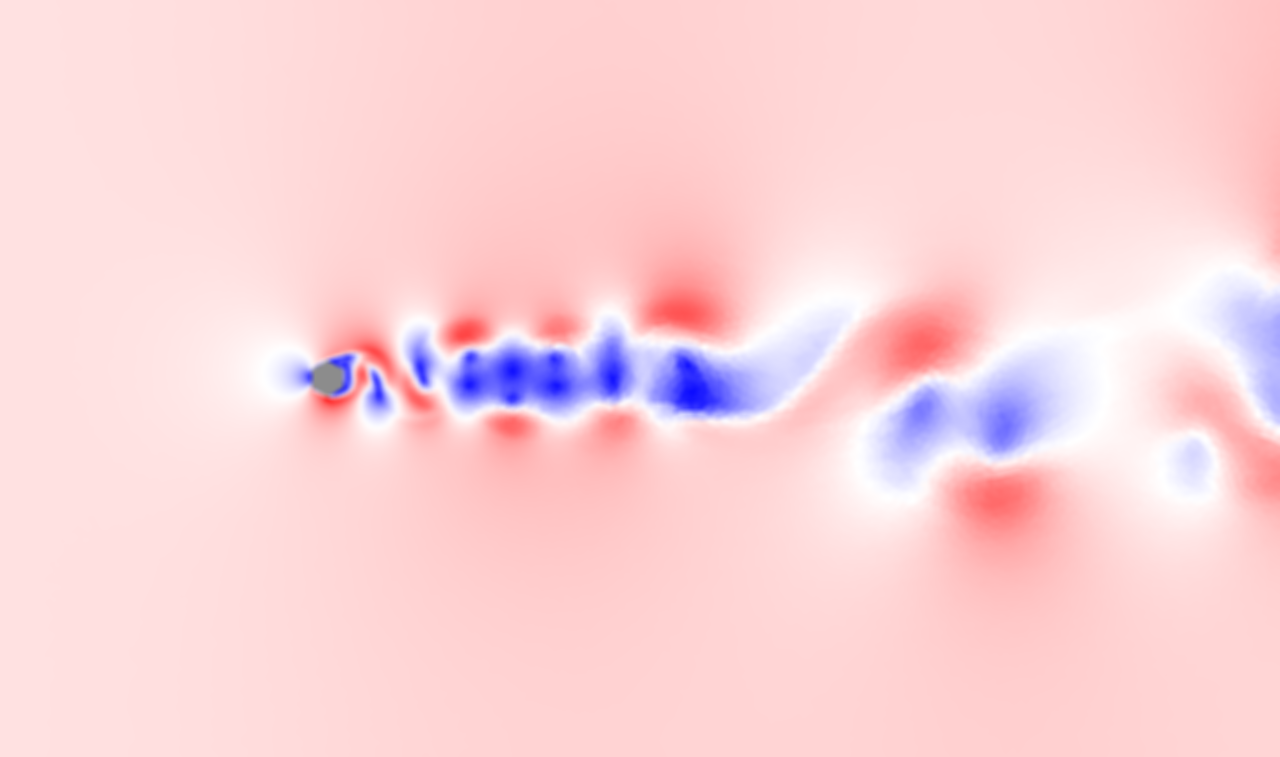

Supplement: S1 Images Folder — Image names are the column headings for and pertain to data in S1, S2, S4 and S5 Datasets. (ZIP) [file pone.0134978.s009.zip › S1_imagesfolder/CT3M3_B.png]

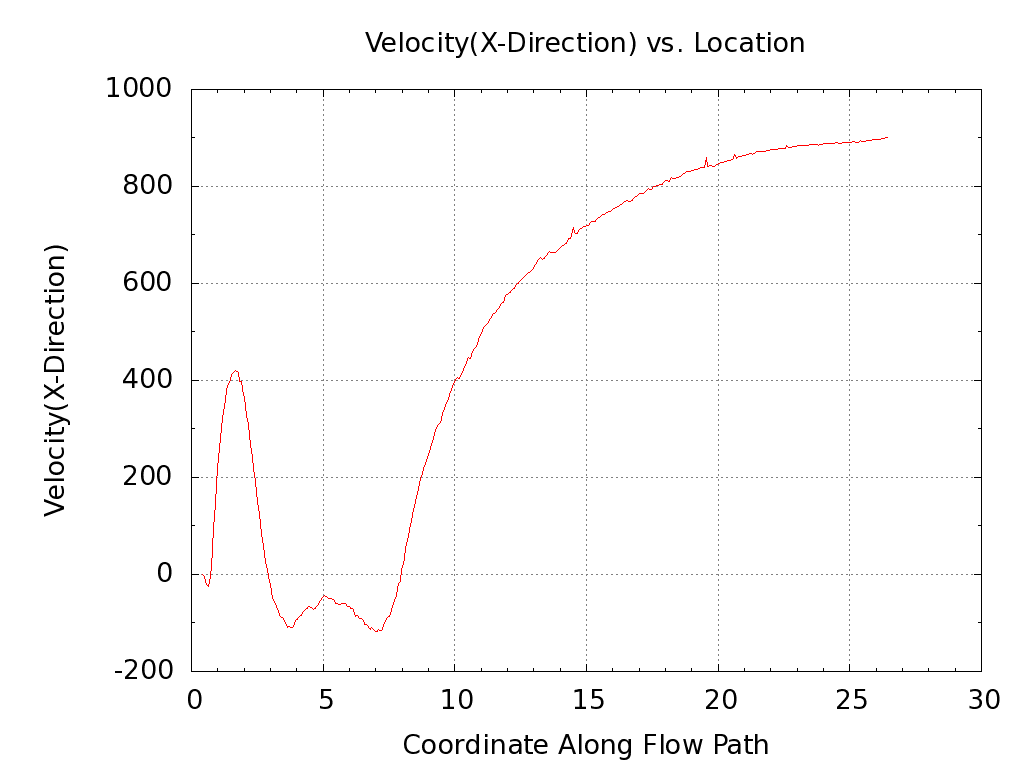

Supplement: S1 Images Folder — Image names are the column headings for and pertain to data in S1, S2, S4 and S5 Datasets. (ZIP) [file pone.0134978.s009.zip › S1_imagesfolder/CT3M3_C.png]

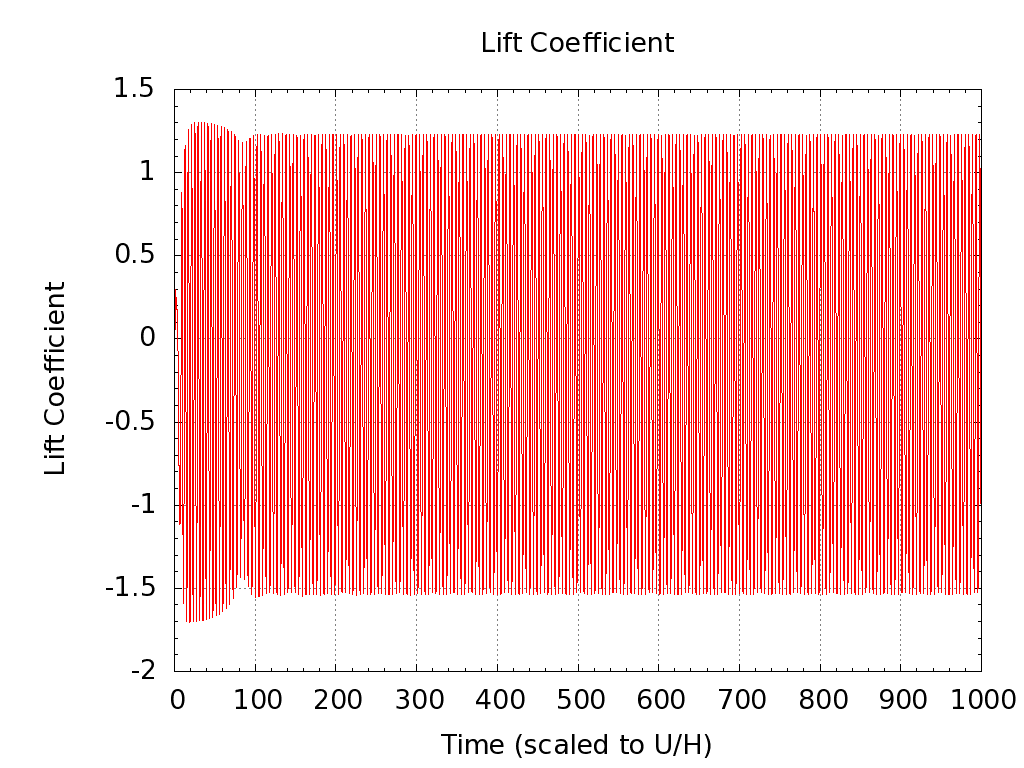

Supplement: S1 Images Folder — Image names are the column headings for and pertain to data in S1, S2, S4 and S5 Datasets. (ZIP) [file pone.0134978.s009.zip › S1_imagesfolder/CT3M3_D.png]

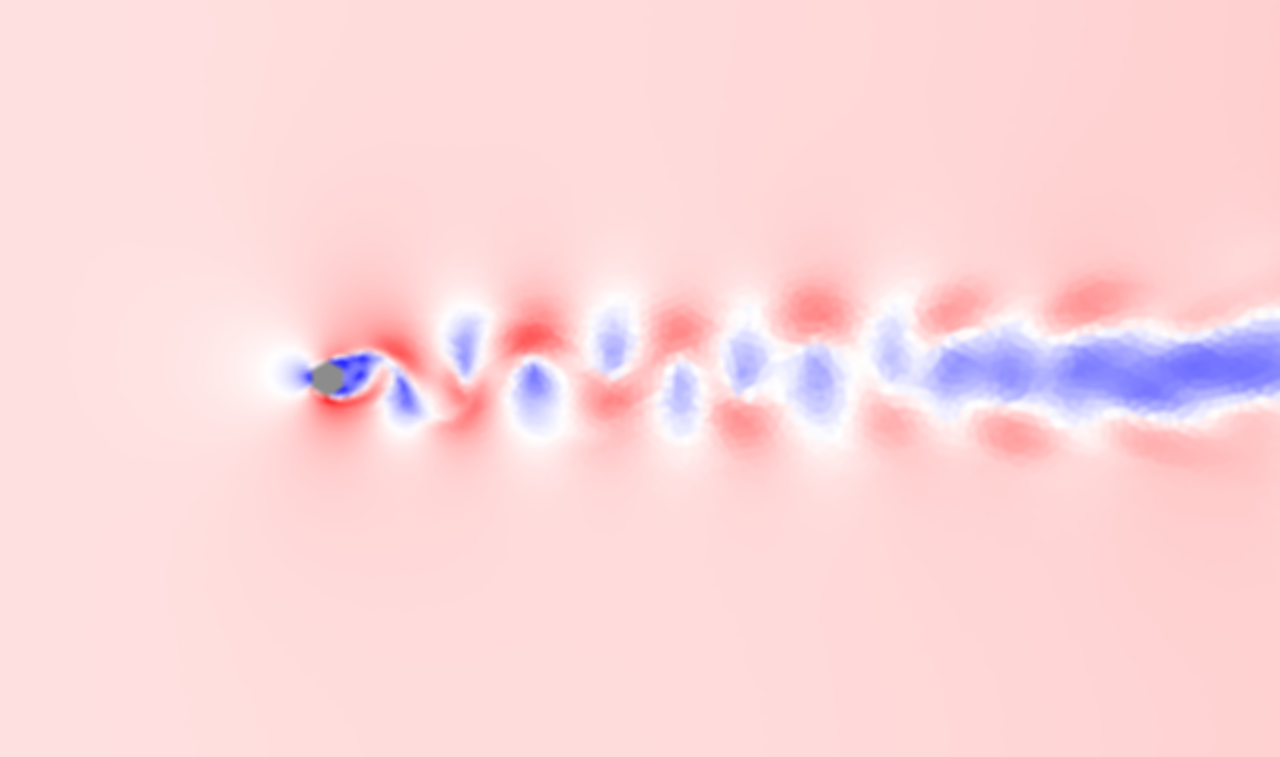

Supplement: S1 Images Folder — Image names are the column headings for and pertain to data in S1, S2, S4 and S5 Datasets. (ZIP) [file pone.0134978.s009.zip › S1_imagesfolder/CTurb1_B.png]

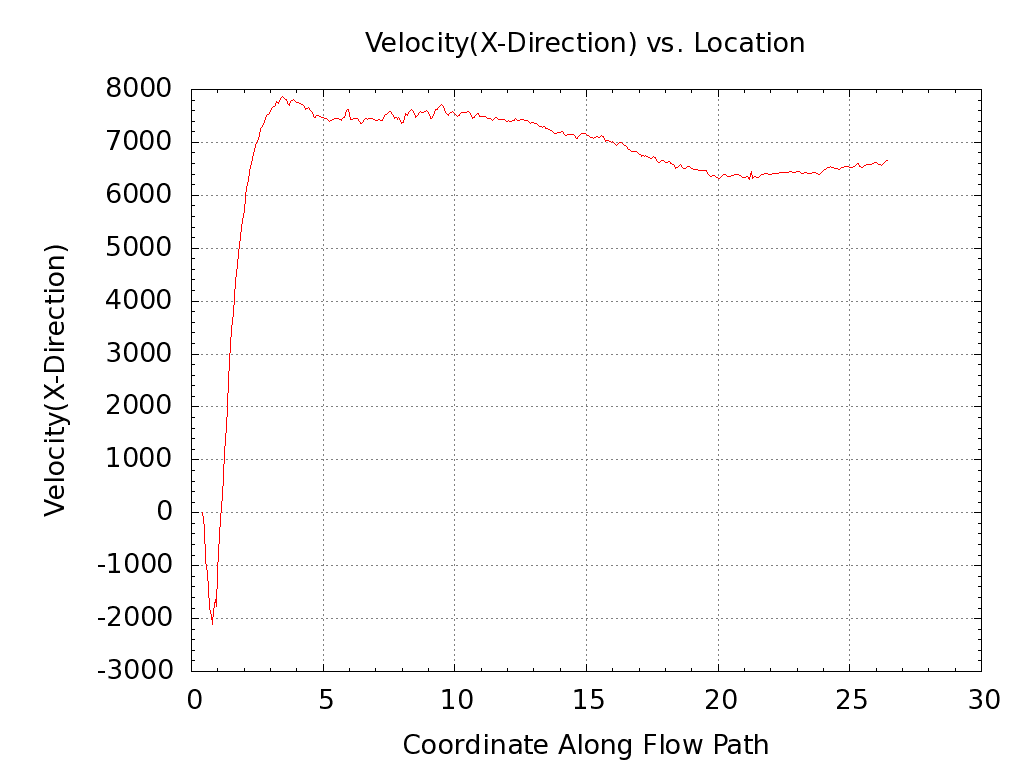

Supplement: S1 Images Folder — Image names are the column headings for and pertain to data in S1, S2, S4 and S5 Datasets. (ZIP) [file pone.0134978.s009.zip › S1_imagesfolder/CTurb1_C.png]

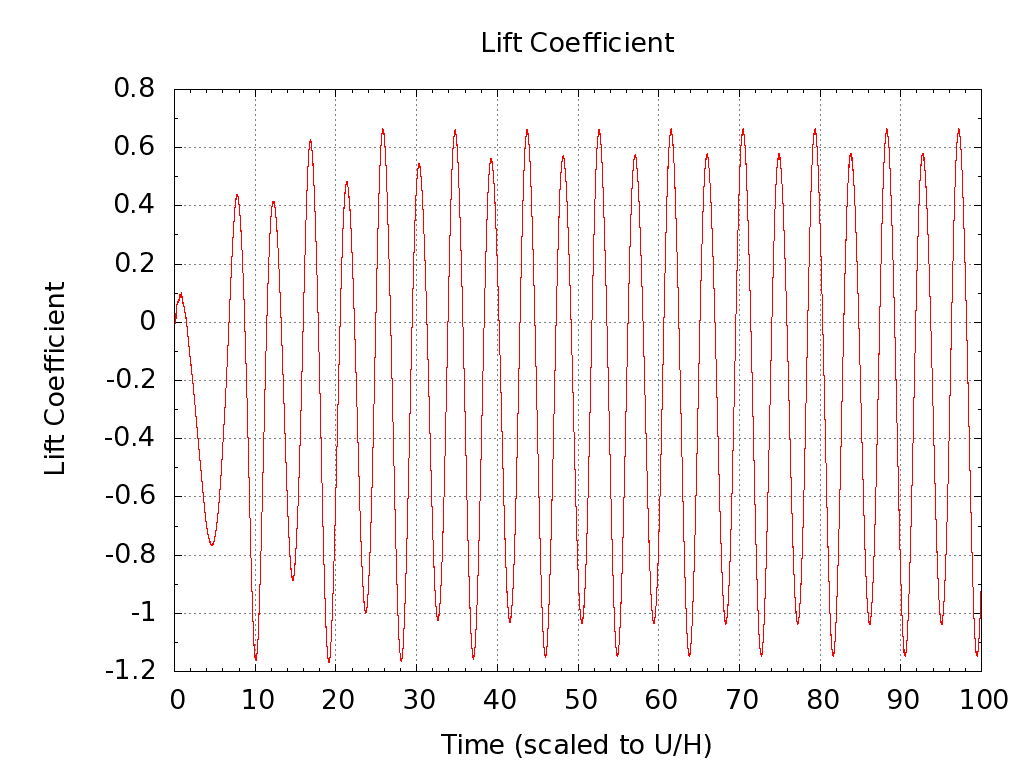

Supplement: S1 Images Folder — Image names are the column headings for and pertain to data in S1, S2, S4 and S5 Datasets. (ZIP) [file pone.0134978.s009.zip › S1_imagesfolder/CTurb1_D.png]

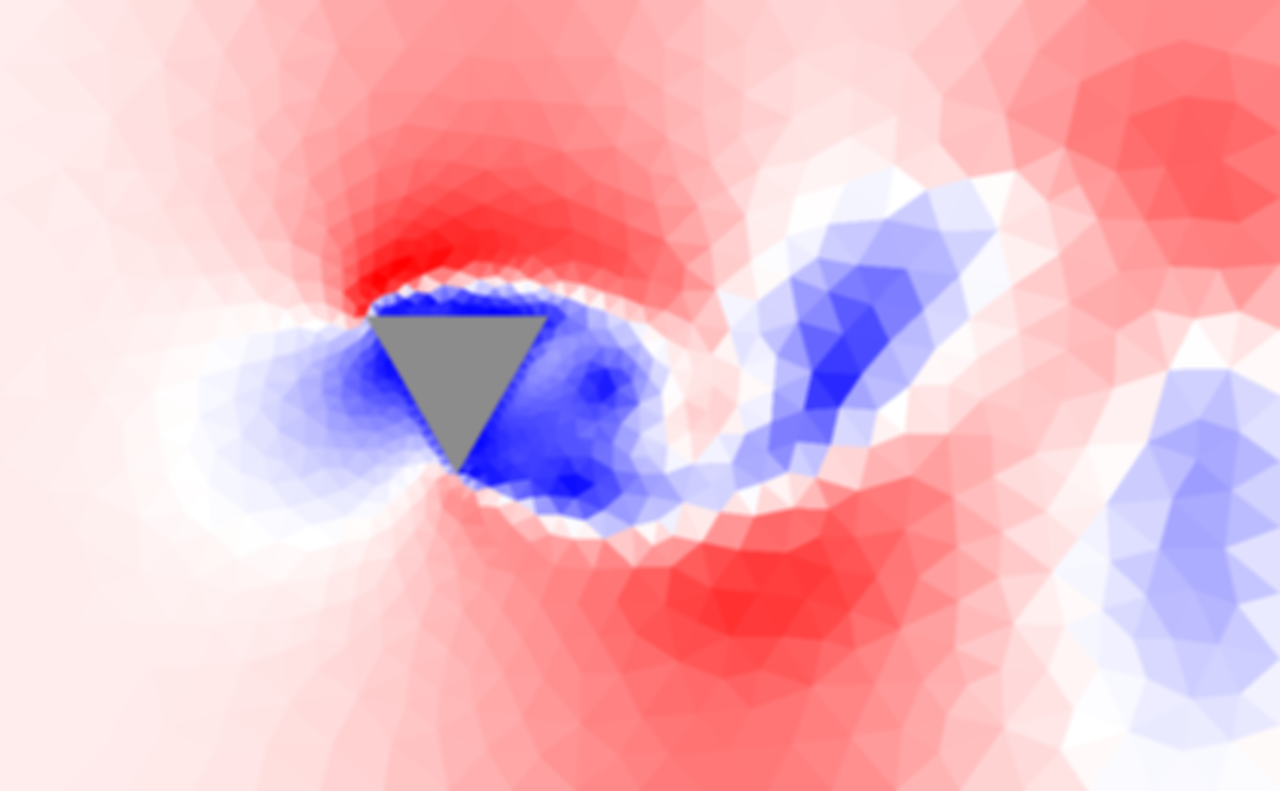

Supplement: S1 Images Folder — Image names are the column headings for and pertain to data in S1, S2, S4 and S5 Datasets. (ZIP) [file pone.0134978.s009.zip › S1_imagesfolder/Dom1_B.png]

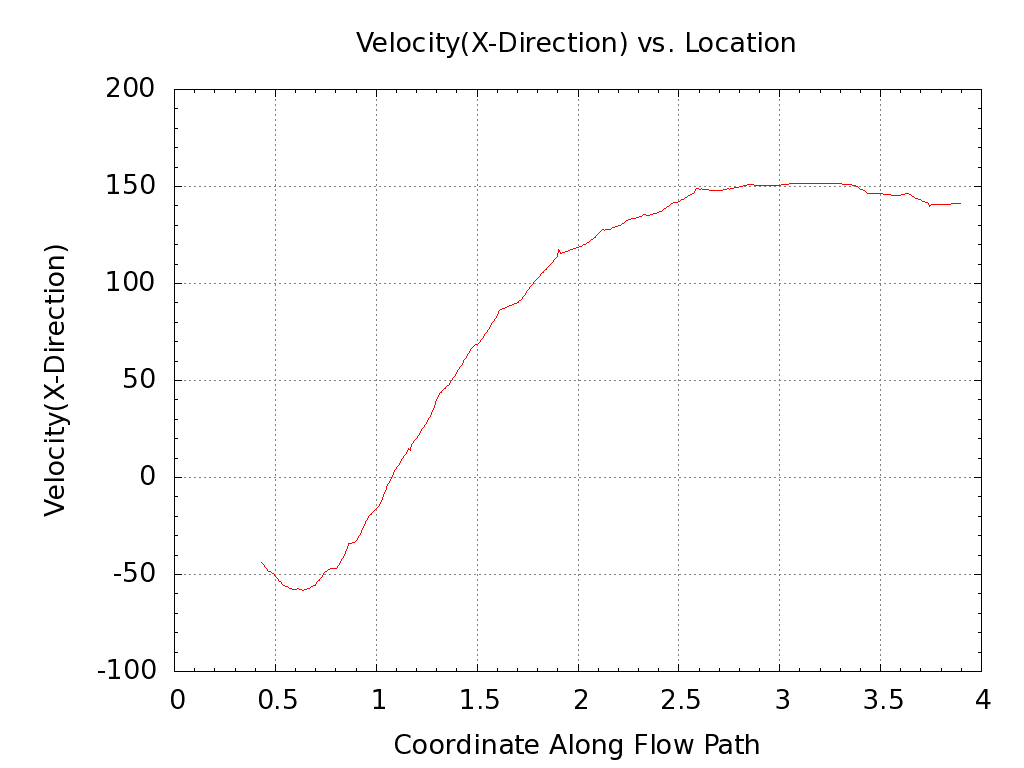

Supplement: S1 Images Folder — Image names are the column headings for and pertain to data in S1, S2, S4 and S5 Datasets. (ZIP) [file pone.0134978.s009.zip › S1_imagesfolder/Dom1_C.png]

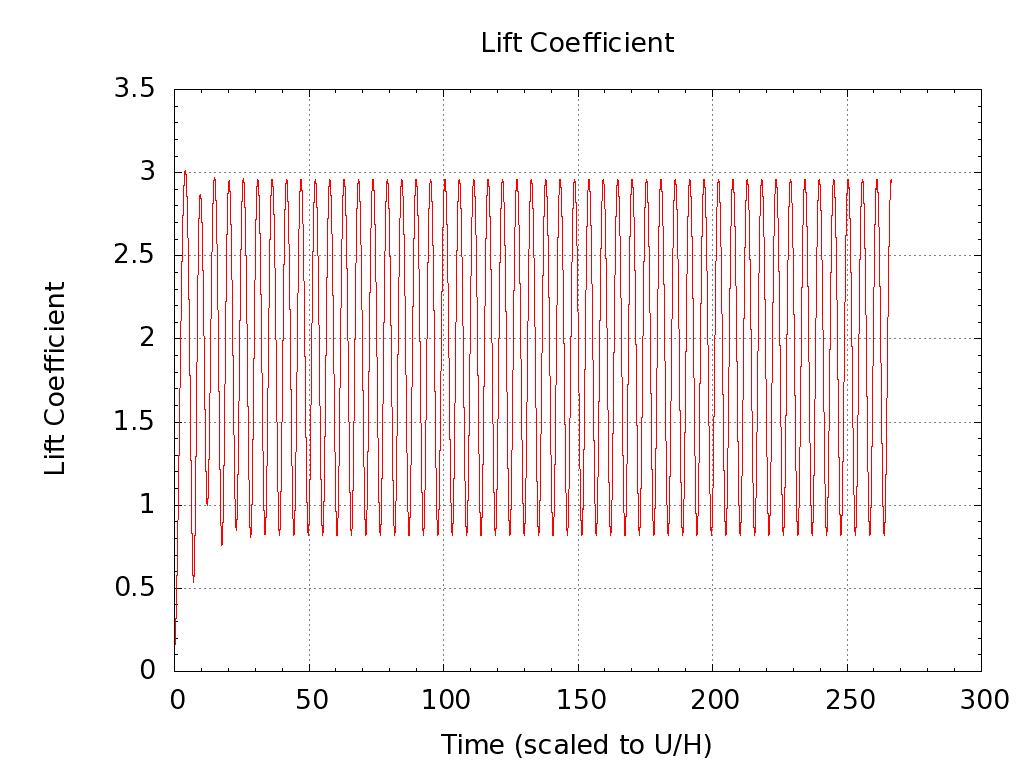

Supplement: S1 Images Folder — Image names are the column headings for and pertain to data in S1, S2, S4 and S5 Datasets. (ZIP) [file pone.0134978.s009.zip › S1_imagesfolder/Dom1_D.png]

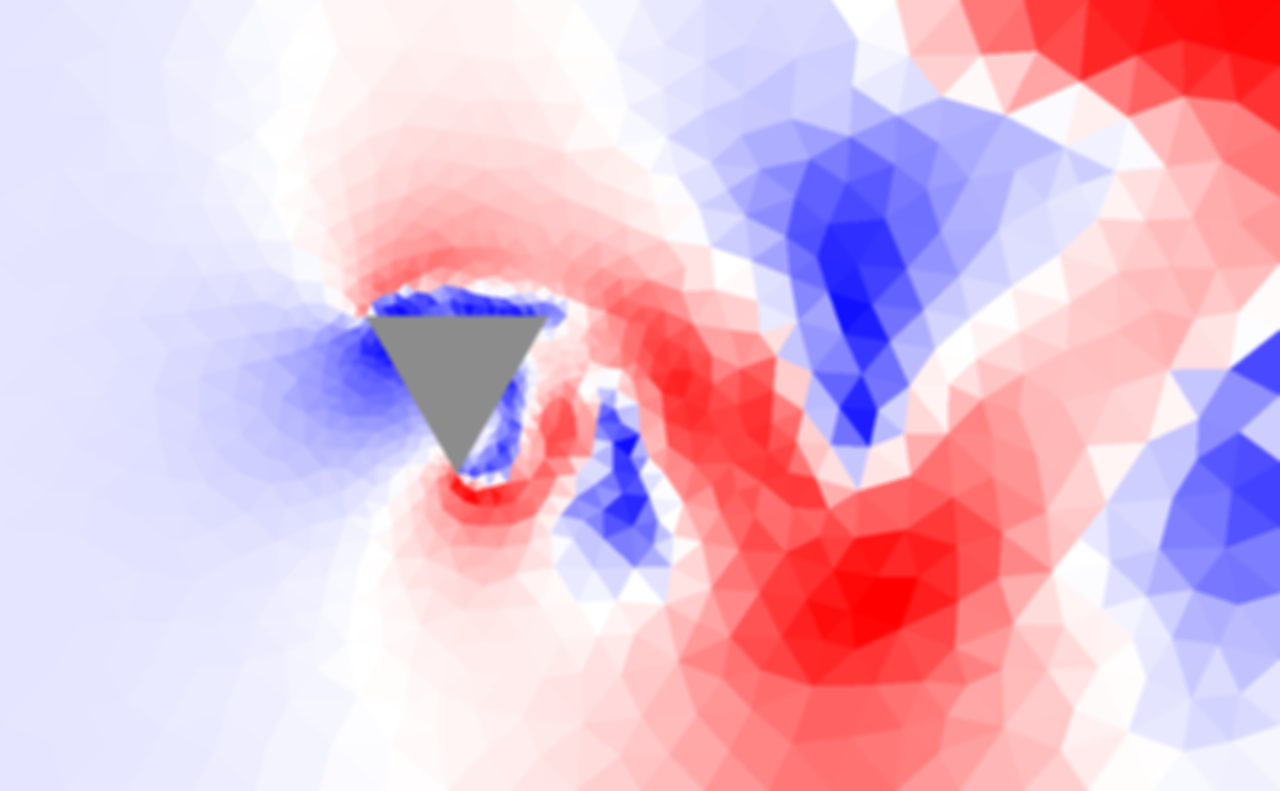

Supplement: S1 Images Folder — Image names are the column headings for and pertain to data in S1, S2, S4 and S5 Datasets. (ZIP) [file pone.0134978.s009.zip › S1_imagesfolder/Dom2_B.png]

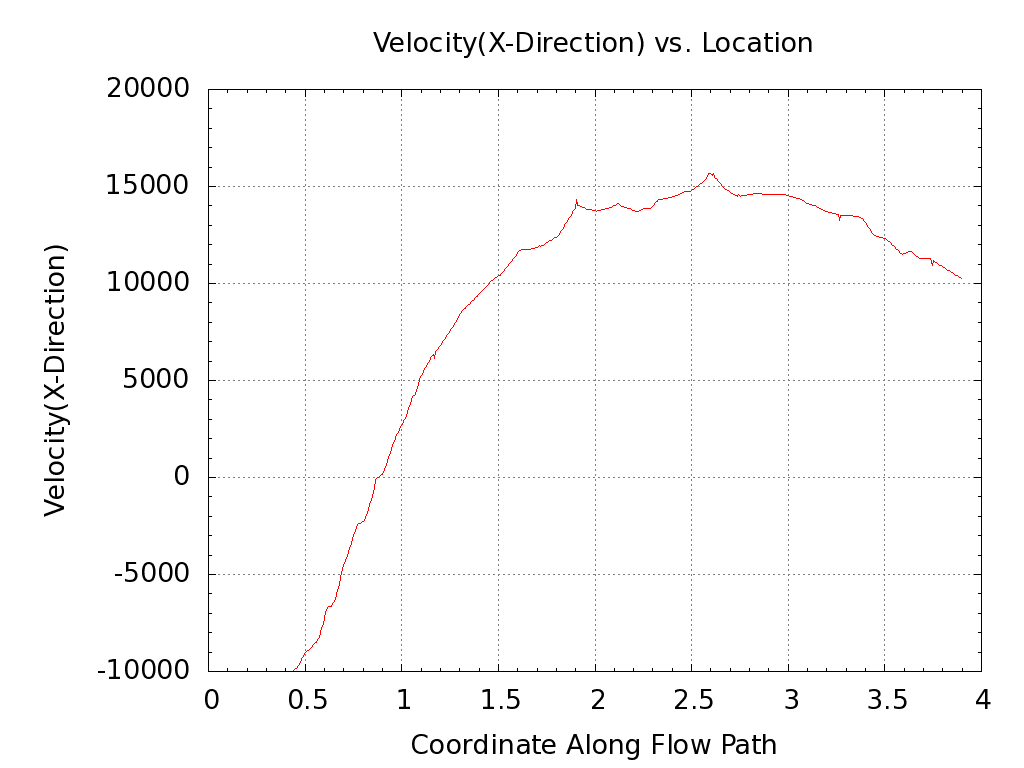

Supplement: S1 Images Folder — Image names are the column headings for and pertain to data in S1, S2, S4 and S5 Datasets. (ZIP) [file pone.0134978.s009.zip › S1_imagesfolder/Dom2_C.png]

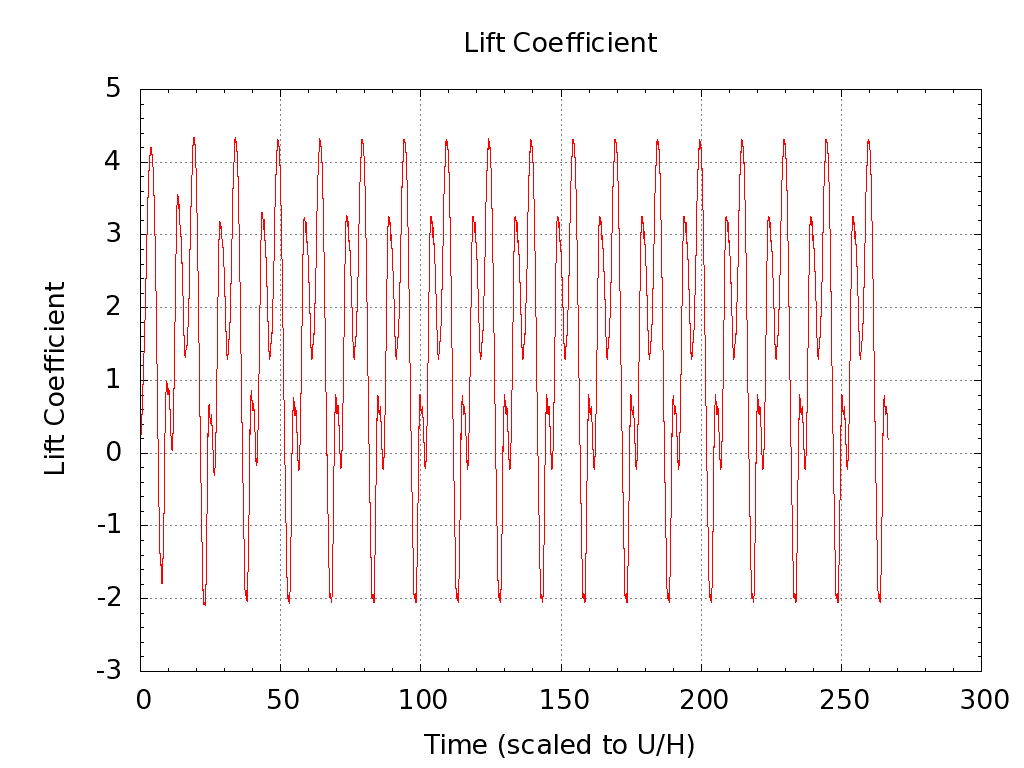

Supplement: S1 Images Folder — Image names are the column headings for and pertain to data in S1, S2, S4 and S5 Datasets. (ZIP) [file pone.0134978.s009.zip › S1_imagesfolder/Dom2_D.png]

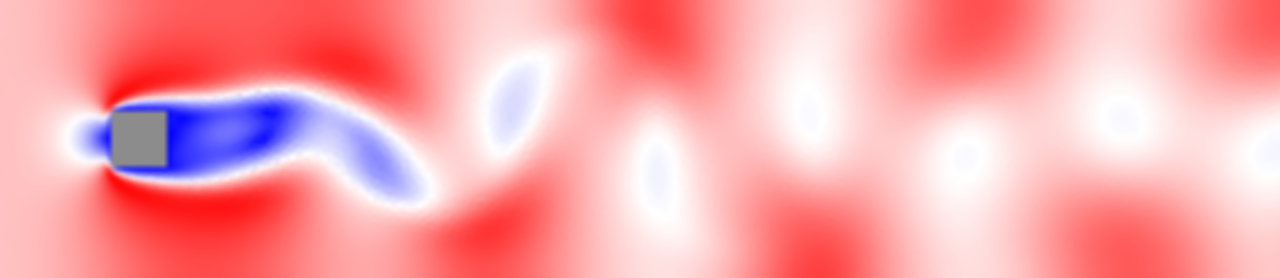

Supplement: S1 Images Folder — Image names are the column headings for and pertain to data in S1, S2, S4 and S5 Datasets. (ZIP) [file pone.0134978.s009.zip › S1_imagesfolder/Dur1_B.png]
