# Supplementary figures and images for: Lessons Learned from Crowdsourcing Complex Engineering Tasks (part 3 of 3)
Source: PLoS One. 2015 Sep 18;10(9):e0134978. doi: 10.1371/journal.pone.0134978 (PMC4575153; doi:10.1371/journal.pone.0134978)

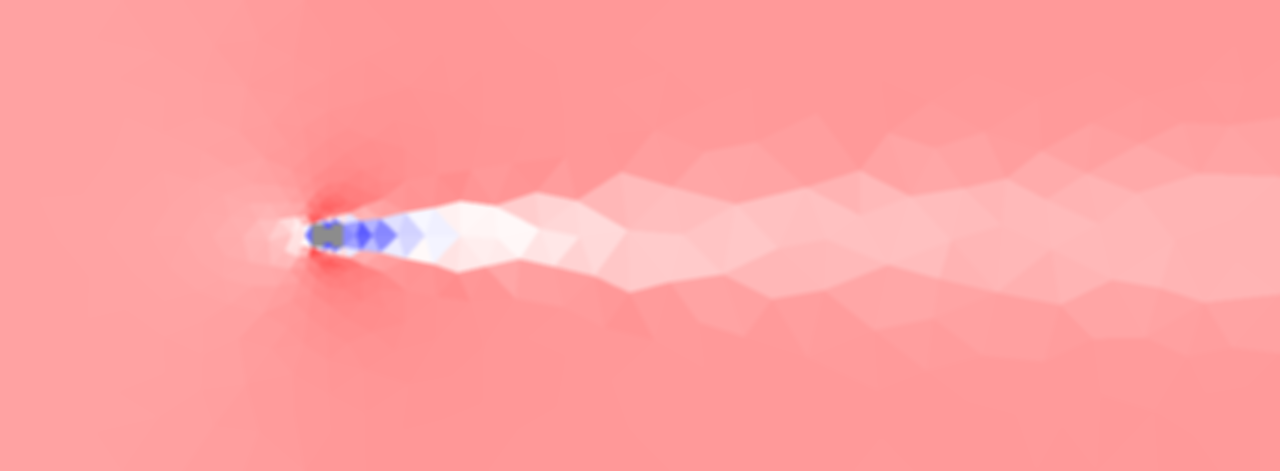

Supplement: S2 Images Folder — The numbers for each folder corresponds to the user number in phase2reports.txt. The Virtual Wind Tunnel does not generate images unless a user asks for a particular image. On a few occasions, users did not even look at certain graphs, so those graphs were not generated. Such non-inspected graphs are not present here. (Note: the x-axis label in the wake stream velocity graph, in Phase 2, due to a typo, indicated a scaling factor that was not actually applied.) (ZIP) [file pone.0134978.s010.zip › S2_imagesfolder/9/U.png]

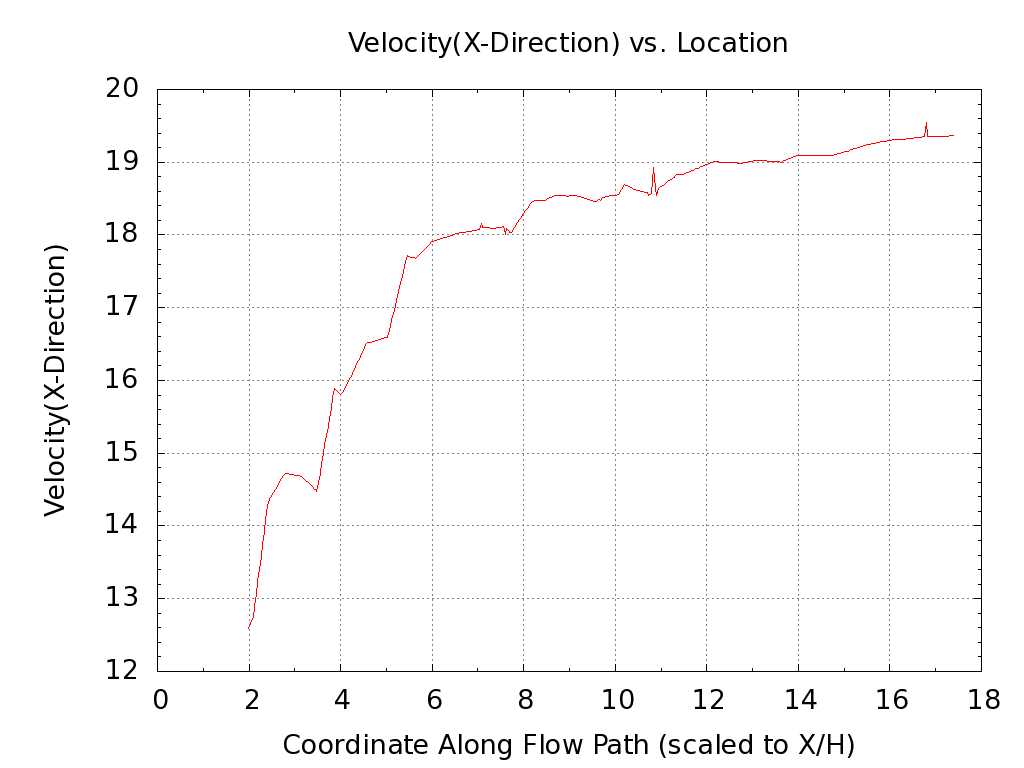

Supplement: S2 Images Folder — The numbers for each folder corresponds to the user number in phase2reports.txt. The Virtual Wind Tunnel does not generate images unless a user asks for a particular image. On a few occasions, users did not even look at certain graphs, so those graphs were not generated. Such non-inspected graphs are not present here. (Note: the x-axis label in the wake stream velocity graph, in Phase 2, due to a typo, indicated a scaling factor that was not actually applied.) (ZIP) [file pone.0134978.s010.zip › S2_imagesfolder/9/wakeCenter.UMean.X.png]
